# Fragment-based design, synthesis and biological evaluation of theophylline derivatives as ATAD2 inhibitors in BT-549 cells

**DOI:** 10.1080/14756366.2023.2242601

**Published:** 2023-08-03

**Authors:** Dahong Yao, Jieshu You, Xuetao Yang, Jin Zhang, Xiaojun Yao

**Affiliations:** a State Key Laboratory of Quality Research in Chinese Medicines, Macau University of Science and Technology, Macau, China; b School of Pharmaceutical Sciences, Shenzhen Technology University, Shenzhen, China; c School of Pharmaceutical Sciences, Medical School, Shenzhen University, Shenzhen, China

**Keywords:** ATAD2, fragment-based, TNBC, apoptosis, migration

## Abstract

ATPase family AAA domain-containing protein 2 (ATAD2) has been emerging as a hot anti-cancer drugable target due to its oncogenic epigenetic modification closely associated with cancer cells proliferation, apoptosis, migration and drug resistance. In this study, we design a series of theophylline derivatives as novel ATAD2 inhibitors through fragment-based screening and scaffold growth strategy. A novel potent ATAD2 inhibitor (compound **19f)** is discovered with an IC_50_ value of 0.27 μM against ATAD2, which adopts a combination of classic and atypical binding mode. Additionally, compound **19f** could impede ATAD2 activity and c-Myc activation, induced significant apoptosis, and illustrated an anti-migration effect in BT-549 cells. Collectively, these results provide new enlightenment for the development of novel potent ATAD2 inhibitors for triple-negative breast cancer (TNBC) treatment.

## Introduction

ATPase family AAA domain-containing protein 2 (ATAD2) contains a bromodomain and an ATPase domain, is an important epigenetic regulator in histone modification, identified as a coactivator of several oncogenic transcription factors, including c-Myc and E2F transcription factor 2 (E2F2) et al.^1^^,^^2^ In our previous review, ATAD2 plays an essential role in cancer chromatin remodelling, DNA replication, DNA damage, and DNA repair, and is associated with cancer cell proliferation, migration, autophagy, and cell cycle regulation^3^. ATAD2 is recruited to the promoter of *BRCA1*, enhancing the efficacy of DNA-damaging chemotherapy agents and radiation^4^. ATAD2 could directly bind to the histone acetylation site of chromatin, elevating chromatin accessibility and histone dynamics which is beneficial to oncogenic gene expression^5^. ATAD2 forms a complex with SOX10 to promote melanoma phenotypes through chromatin remodeling^6^. Additionally, ATAD2 is proven to be a marker and driver of cell proliferation in ovarian cancer, and MYBL2-ATAD2 proliferative signalling axis is confirmed by Liu et al.^7^ Due to its critical and extensive regulatory role, ATAD2 has emerged as a promising drug target for cancer treatment.

**Figure 1.**
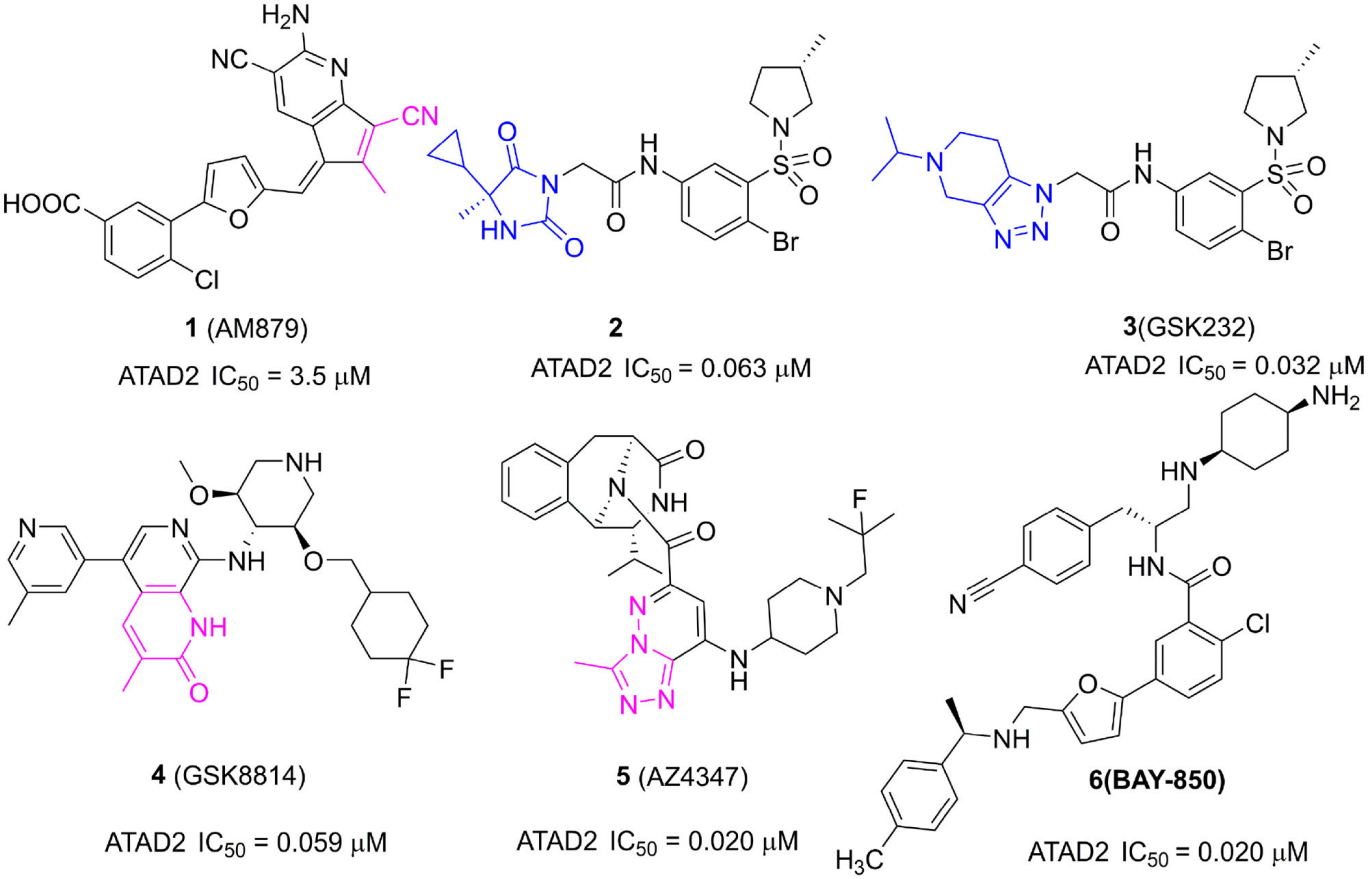
Several representative ATAD2 inhibitors. The pink group indicates the classical acetyl group analog unit initiating hydrogen with ASN1064 and the blue groups indicate non-classical ZA loop region binding units.

Some ATAD2 inhibitors have been discovered over the past few years for cancer treatment (Figure 1). In our previous work, a novel ATAD2 inhibitor (**1**, **AM879**) was discovered by a structure-based virtual screening synergy with a biochemical analysis strategy. Structurally, the cyano group of AM879 is very important for the affinity of ATAD2/AM879 by initiating a hydrogen network with key conserved Asn1064 and Tyr1021. AM879 shows potent inhibitory activity against ATAD2 with an IC_50_ value of 3.5 μM and acceptable antiproliferative activity with an IC_50_ value of 2.43 μM in MDA-MB-231 cells. AM879 could suppress the expression of c-Myc, and induce significant apoptosis and autophagy^8^. Another new ATAD2 inhibitor (**2**) is reported by Robert J. Watson, which is identified by high-throughput screening and hit qualification. A novel binding mode is observed in the X-ray structure of ATAD2 with **2,** there is no hydrogen interaction initiated by Asn1064. By contrast, three key hydrogen interactions are initiated by residues Arg1007, Lys1011, and Asp1014 from the ZA loop^9^. A structural derivative (**3**, **GSK232**) of **2** displays an improvement activity against ATAD2 with an IC_50_ value of 0.032 μM in an atypical binding mode, but no more cellular activity was observed^10^. 3-methylquinolinone derivatives are identified as potent ATAD2 inhibitors, the first reported 2 digits nanomolar selective ATAD2 inhibitor is **GSK8814** (**4**) with an IC_50_ value of 0.059 μM, which is used as a cell-permeable chemical probe for ATAD2 Bromodomain^11^. **AZ4347** (**5**) is a recently reported potent ATAD2 inhibitor with antiproliferative activity in a range of breast cancer models^12^. Another potent ATAD2 inhibitor is **BAY-850** (**6**) identified by a DNA-encoded library screen, which could specifically induce ATAD2 bromodomain dimerisation and prevented interactions with acetylated histones^13^. Although several potent and selective ATAD2 inhibitors have been reported, most lack appreciable cellular activity and druggability, especially in inhibiting tumour growth, which limits the clinical application of ATAD2 inhibitors. The discovery of new chemical frameworks is very important for improving cell penetration and antitumor activity of ATAD2 inhibitors.

In this study, a series of theophylline derivatives are designed and synthesised through fragment-based screening and scaffold growth strategy. Compound **19f** is identified as a potent ATAD2 inhibitor with an IC_50_ value of 0.27 μM, which could significantly inhibit BT-549 cells proliferation with an IC_50_ value of 5.43 μM. In addition, compound **19f** was substantiated to impede ATAD2 activity and c-Myc activation, induced significant apoptosis, and showed anti-migration ability in BT-549 cells. Taken together, these findings demonstrate that compound **19f** is a novel ATAD2 inhibitor, which may provide a candidate lead for future TNBC drug development.

## Results and discussion

### Fragment-based design novel ATAD2 inhibitors

**Figure 2.**
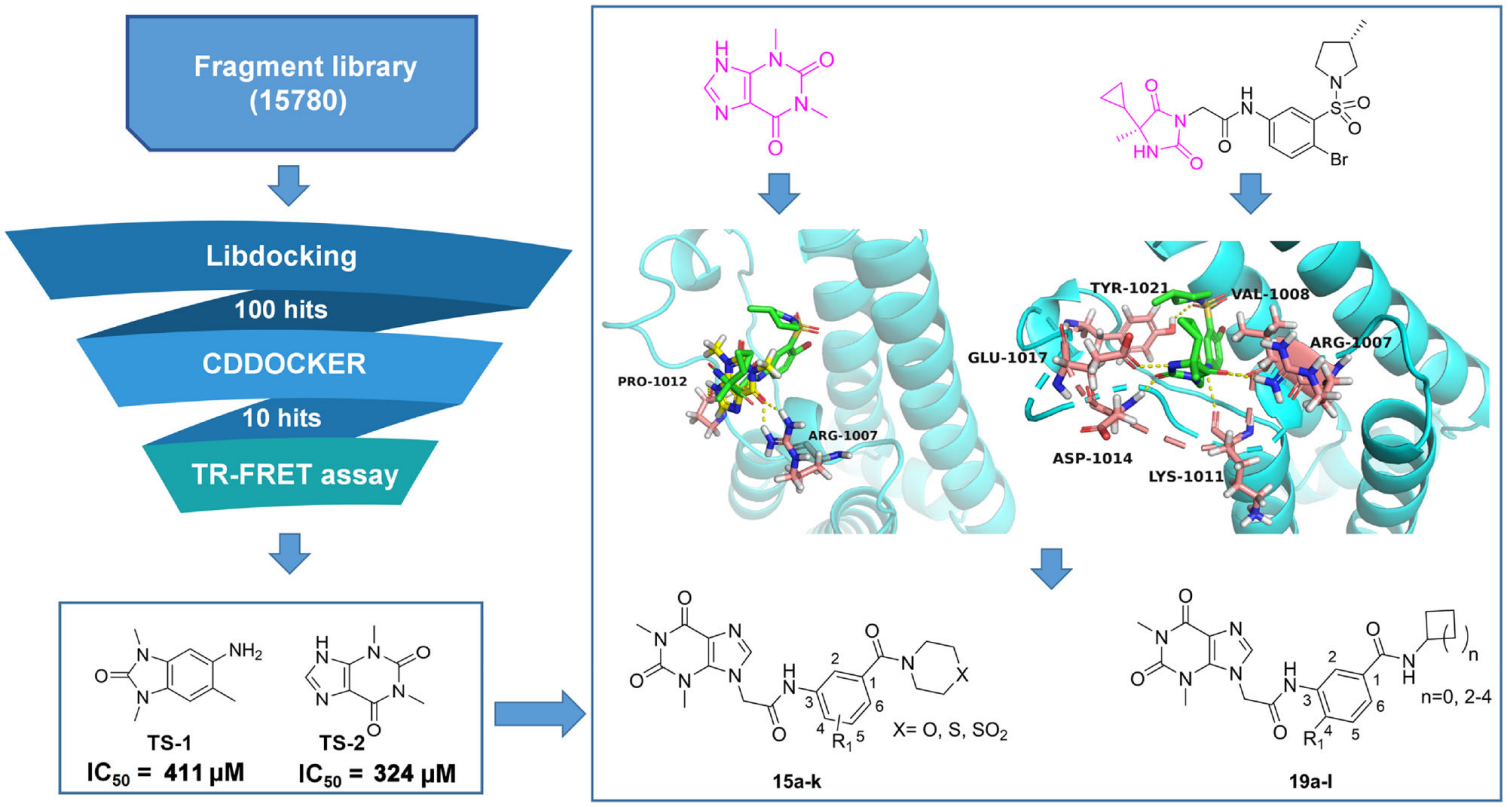
Fragment-Based design of novel ATAD2 inhibitors.

To discover potent ATAD2 inhibitors with novel scaffolders, a fragment-based screening strategy is performed (Figure 2). First, we screened a small molecule fragment library built by limiting molecular weight among 100–250 Da through the Libdocking protocol^14^. The top 100 hits were retained to access to next processing, and the CDOCKER interaction energy was calculated^15^. Subsequently, we detected the affinity of the top 10 hits with ATAD2 by TR-FRET assay. The results showed that only 2 hits (TS-1, TS-2) presented weak affinity (Figure 2(B)) with IC_50_ values of 411 μM and 324 μM respectively. Structurally, TS-2 (theophylline) is a novel scaffold for ATAD2 inhibitors and may have a non-classical binding mode similar to that of **2**, while TS-1 has been reported in the previous study^16^. The molecular docking result suggests that theophylline binds to the ZA loop of ATAD2 bromodomain, initiating three hydrogen interactions with residues Arg1007 and Pro1012, sharing a similar conformation of **2**, which indicates that theophylline is a promising start point to optimise to be novel ATAD2 inhibitors. Compared to the structure of **2**, we induced a chain of two carbon atoms as the linker to expand into the classical active centre (Asn1064). Compound **2** doesn’t form a hydrogen interaction with Asn1064, which may limit its affinity with ATAD2. Hence, we designed a series of benzamide derivatives, expecting that the amide bond could initiate a hydrogen bond with Asn1064 to enhance the Affinity. Additionally, a small size hydrophobic group (usually methyl) occupying a hydrophobic site was observed next to the hydrogen bond receptor, which would heighten the binding of ATAD2 and Ligand. Methyl or methoxyl was incorporated into the 4-site of the benzene ring to mimic the “methyl” of the substrate acetyl group. Collectively, we designed a series of theophylline derivatives as novel ATAD2 inhibitors through fragment-based screening and scaffold growth strategy.

### Chemistry

**Scheme 1.**
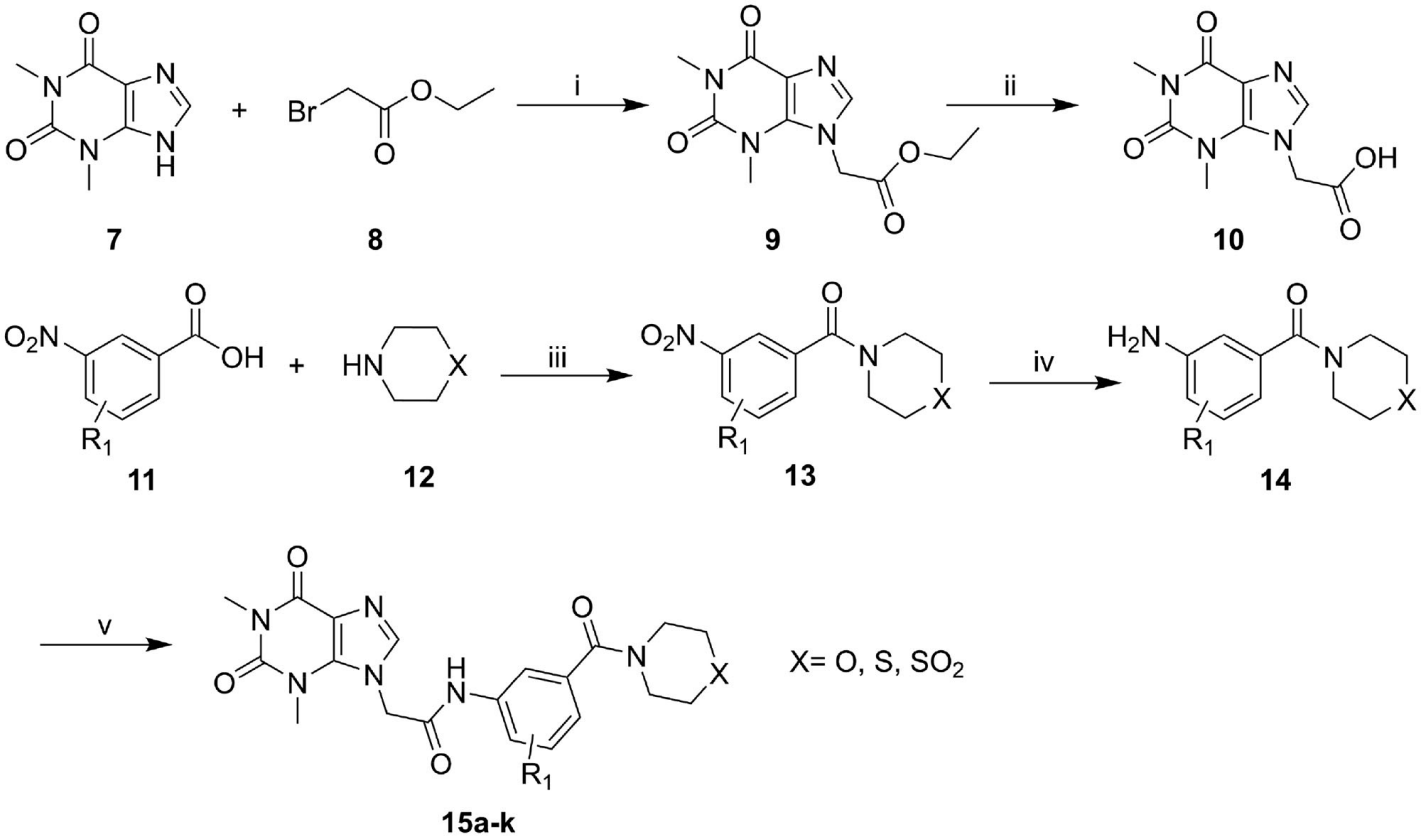
General synthesis of compounds **15a–k**. Reagents and conditions: (i) K_2_CO_3_, DMF, 60 °C, 6 h; (ii) methanol, LiOH, r.t., 8 h; (iii) HATU, DIEA, r.t., 24 h; (iv) Fe, NH_4_Cl, 90 °C refluxed, 3h; (v) HATU, DIEA, r.t., 24 h.

**Scheme 2.**
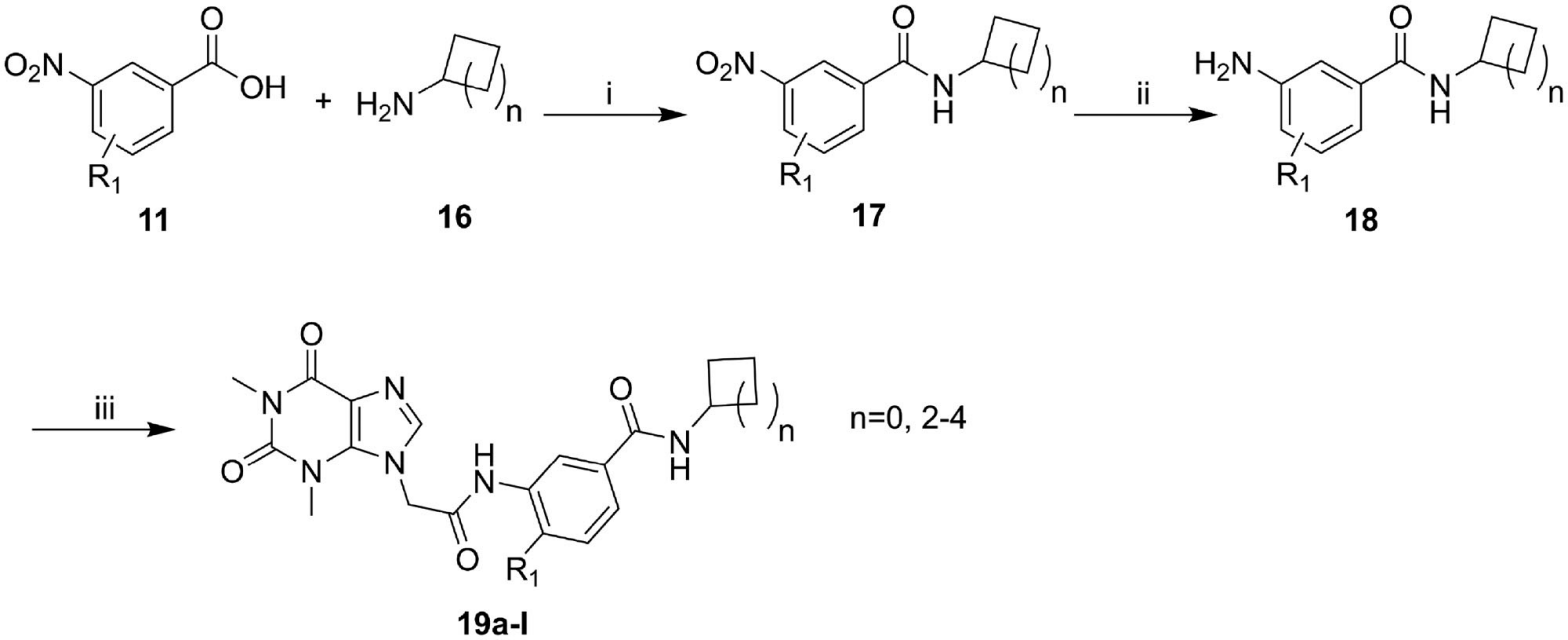
General synthesis of compounds **19a–i**. Reagents and conditions: (i) HATU, DIEA, r.t., 24 h; (ii) Fe, NH_4_Cl, 90 °C refluxed, 3h; (iii) HATU, DIEA, r.t., 24 h.

The synthesis of compounds **15a–k** and **19a–l** derivatives was carried out (Schemes 1–2). Intermediate **9** was prepared by a substitution reaction of **7** with ethyl bromoacetate (**2**), and intermediate **9** was hydrolysed to give intermediate **10**. The reaction of intermediate **11** with secondary amine derivatives (**12**) yielded intermediate **13** and further reduced to obtain intermediate **14**. Compounds **15** were prepared through an amide condensation of intermediate **10** and **14.** Additionally, Compounds **19a–l** were synthesised by a similar route as compounds **15**.

### Analysis of the structure-activity relationship

To obtain novel potent ATAD2 inhibitors by optimising from theophylline based on scaffold growth strategy, according to the docking results of theophylline-ATAD2, the thiomorpholine group was incorporated into the middle benzene ring via an amine bond to yield **15a** without ATAD2 inhibition activity (Table 1). Furthermore, we induced methyl and methoxy to the 4-site of the middle benzene ring (**15b**, **15c**), displaying weak inhibitory activities against ATAD2. We hypothesised that hydrogen bond receptors could enhance the affinity of ATAD2, so the thiomorpholine group was substituted with the thiomorpholine 1,1-dioxide group (**15d–15f**), resulting in a complete loss of activity against ATAD2. Next, the replacement of the S atom with an oxygen atom yielded **15 g**, displaying a weak inhibition potency. Different substituents were induced into the AROMATIC ring to yield **15h–15k**, the 4-site methoxyl substituted (**15i**) presented the most potent activity against ATAD2 with an IC_50_ value of 11.32 μM. On the whole, the round of structural optimisation is not successful. We rechecked the binding configuration of ligand and ATAD2 and speculated that the secondary amide failed to form the active configuration due to its rigidity. Next, the secondary amide was replaced by the primary amide to improve the flexibility of the ligand. First, the cyclopropylamine substituted (**19a–19c**) display significant improvement in ATAD2 inhibitory activity, a similar potency (IC_50_ ≈ 9 μM) was observed between **19b** and **19c** (Table 2). Encouraged by the results, cyclopentyl, a bigger group, was induced to yield **19d** with an improved IC_50_ value of 5.13 μM. The methyl (**19e**) exhibited about a three-fold increase in activity compared to **19d**. The methoxy (**19f**) presented the most potent activity against ATAD2 with an IC_50_ value of 0.27 μM. Further increasing the size of the substituent resulted in a significant decrease in activity (**19 g–19i**) or even complete loss (**19j–19l**). *In vitro* anti-proliferation assays, these compounds only presented a low micromole antiproliferatory potency in BT-549 cells. The most potent compound **19f** displayed an IC_50_ value of 5.43 μM against BT-549 cells for 24 h. Although the antiproliferative activity of **19f** is about one-fold weaker than that of the positive drug (**BAY-850**), it is the first compound with the particular binding mode inhibitor to show moderate inhibitory activity, which still has positive implications for the design of novel ATAD2 inhibitors.

**Table 1.** The ATAD2 inhibitory activity of compounds **15a–k**.

	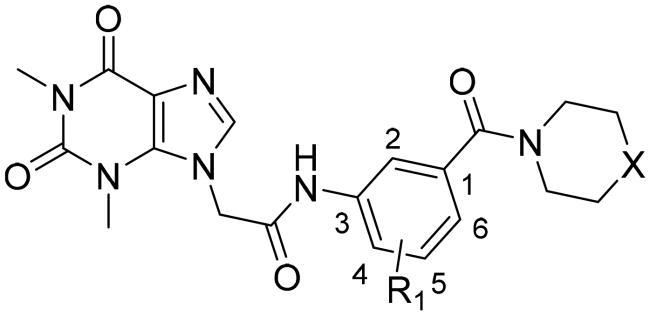			
Compound	*R*_1_	X	Kinase inhibitory activity (IC_50_, μM)^a^	Anti-proliferative activity (IC_50_, μM)^a^
			ATAD2	BT-549 cells
**15a**	4-H	S	>25	>30
**15b**	4-CH_3_	S	19.34 ± 2.35	>30
**15c**	4-OCH_3_	S	14.78 ± 1.78	>30
**15d**	4-H	SO_2_	>25	>30
**15e**	4-CH_3_	SO_2_	>25	>30
**15f**	4-OCH_3_	SO_2_	>25	>30
**15g**	4-H	O	21.23 ± 3.17	>30
**15h**	4-CH_3_	O	>25	>30
**15i**	4-OCH_3_	O	11.32 ± 2.79	27.98 ± 3.61
**15j**	4-Cl	O	15.24 ± 3.63	26.07 ± 3.77
**15k**	2-F	O	>25	>30

^a^Each compound was tested in triplicate; the data are presented as the mean ± SD.

**Table 2.** the ATAD2 inhibitory activity of compounds **19a–l**.

	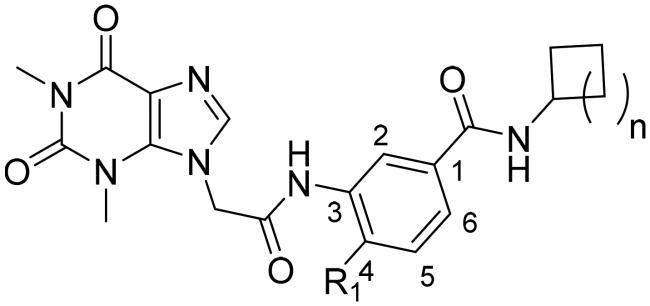			
Compound	R_1_	n	Kinase inhibitory activity (IC_50_, μM)^a^	Anti-proliferative activity (IC_50_, μM)^a^
			ATAD2	BT-549 cells
**19a**	4-H	0	13.83 ± 2.57	>30
**19b**	4-CH_3_	0	9.06 ± 1.81	18.72 ± 3.06
**19c**	4-OCH_3_	0	9.19 ± 1.90	21.89 ± 4.03
**19d**	4-H	2	5.13 ± 0.72	17.91 ± 3.54
**19e**	4-CH_3_	2	1.78 ± 0.39	11.32 ± 2.70
**19f**	4-OCH_3_	2	0.27 ± 0.05	5.43 ± 0.71
**19g**	4-H	3	17.26 ± 3.92	25.08 ± 4.27
**19h**	4-CH_3_	3	19.71 ± 4.38	11.72 ± 2.46
**19i**	4-OCH_3_	3	12.33 ± 3.06	22.01 ± 3.35
**19j**	4-H	4	>25	>30
**19k**	4-CH_3_	4	>25	>30
**19l**	4-OCH_3_	4	>25	>30
**BAY-850**	–	–	0.16 ± 0.04	3.45 ± 0.59
**AM879**	–	–	3.49 ± 0.77	4.79 ± 0.52

^a^Each compound was tested in triplicate; the data are presented as the mean ± SD.

### Molecular docking and molecular dynamic simulation

**Figure 3.**
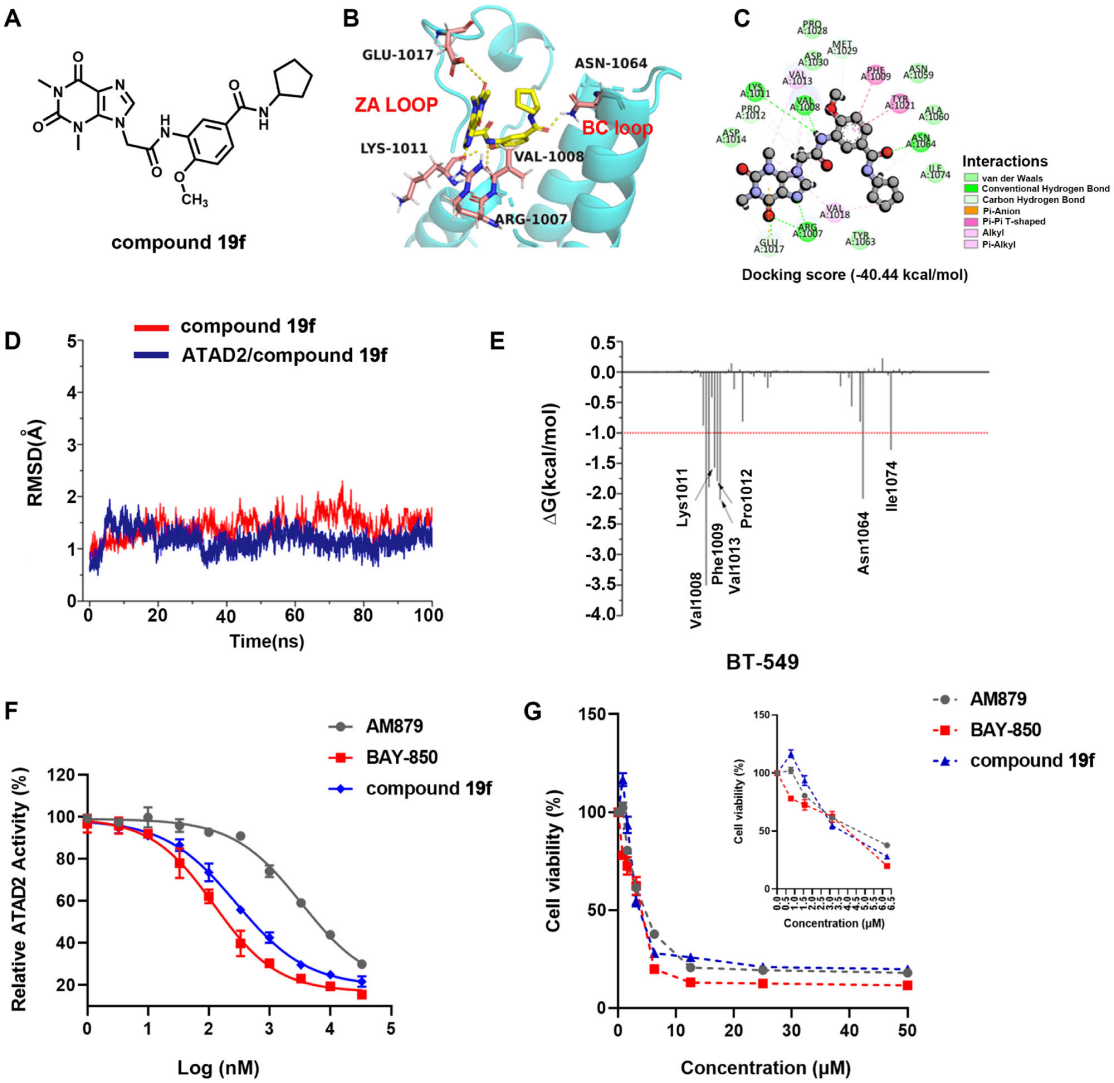
The docking of compound **19f** and ATAD2 bromodomain. (A) The structure of compound **19f**; (B) The hydrogen interactions of compound **19f** and ATAD2 bromodomain in the cartoon; (C) The detailed interactions between compound **19f** and ATAD2 bromodomain; (D) The RMSD of ATAD2 atoms around 5 Å of compound **19f**; (E) the residues energy decomposition of compound **19f** and ATAD2; (F) The effect of AM879, BAY-850 and compound **19f** on ATAD2 activity detected by TR-FRET technology; (G) The effect of AM879, BAY-850, and compound **19f** on the cell viability of BT-549 cells.

To explore the binding mode of compound **19f** (Figure 3(A)) and ATAD2, molecular docking was performed (Figure 3(B,C)). The results show that compound **19f** could be combined with ATAD2 active site with an interaction energy value of −40.44 kcal/mol and the theophylline group initiates two key hydrogen interactions with the conserved residue Arg1007 from the ZA loop of ATAD2. Additional three hydrogen bonds were observed between the amide group of the linker and residues Val1008, Lys1011, and Glu1017, and the benzene ring interacts with residues Phe1009 and Tyr1021 through two Pi-Pi interactions. Furthermore, the methoxy group at the 4-site occupies the hydrophobic pocket near the active centre (Asn1064), mimicking the “methyl” of the acetyl group from substrates. Surprisingly, an important hydrogen interaction is formed between the terminal amide group and residue Asn1064, which is rigidly conservative for the classic ATAD2 inhibitors. The cyclopentyl positions to the solvent-accessible area as the previously reported atypical binding mode of **2** and ATAD2. Furthermore, the root-mean-square deviation (RMSD) of ATAD2 atoms around 5 Å of compound **19f** fluctuated between 0.5 and 2.1 in 100 ns MD simulation (Figure 3(D)), which suggested the bindings of compound **19f** and ATAD2 presented fairly stable active profiles. In addition, the binding free energy of the system revealed that compound **19f** showed the strongest binding affinity against ATAD2, which further confirmed the strong enzyme inhibition activity (Table 3). Subsequently, the residues energy decomposition indicated that residues Lys1011, Nal1008, Phe1009, Val1013, Asn1064, and Ile1074 made a great contribution to the binding of compound **19f** and ATAD2 (Figure 3(E)). In addition, compound **19f** showed a weaker ATAD2 inhibitory activity compared with BAY-850, but a stronger ATAD2 inhibitory activity than AM879, which was previously found by us (Figure 3(F)). These results demonstrated that compound **19f** is a potent ATAD2 inhibitor adopting a combination of classic and atypical binding mode. Intriguingly, compound **19f** showed anti-proliferative activity comparable to BAY-850 and AM879 in BT-549 cells (Figure 3(G)). In another TNBC cell line MDA-MB-231 cells, compound **19f** also illustrated good anti-proliferative activity, but did not show obvious cytotoxicity to normal breast MCF-10A cells (Figure S1). Taken together, compound **19f** might be a promising candidate for TNBC treatment.

**Table 3.** The binding free energies were calculated by MM-GBSA method (kcal/mol).

Compd.	Δ*E*_ele_	Δ*E*_vdw_	Δ*G*_sol-np_	Δ*G*_sol-ele_	Δ*G*_polar_^a^	Δ*G*_nonpolar_^b^	Δ*H*_bind_	*T*Δ*S*	Δ*G*_bind_
**19f**	−30.48	−42.96	−5.84	42.76	12.28	−48.80	−36.52	−23.82	−12.70

^a^ΔGpolar = ΔEele + ΔEvdw.

^b^ΔGnonpolar = ΔGvdw + ΔGsol-np.

### Compound 19f inhibits ATAD2-Myc activation in BT-549 cells

**Figure 4.**
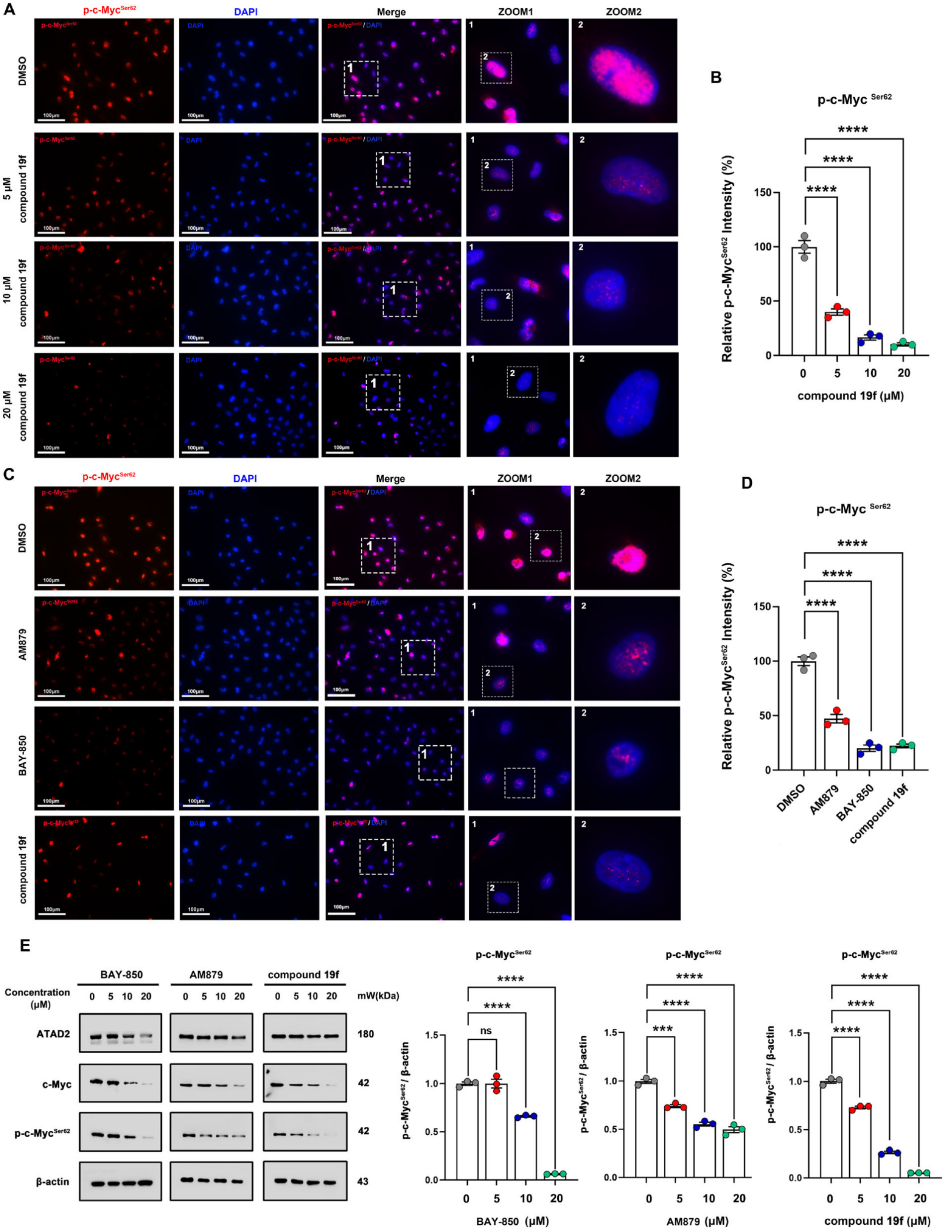
compound **19f** inhibits ATAD2-Myc activation in TNBC cells. (A) BT-549 cells were treated with 5, 10, 20 μM compound **19f** for 48h, and the expression level of p-c-Myc^Ser62^ was detected by immunofluorescence. The nuclei were labelled with DAPI. Scale bar = 100 μm. (B) Relative p-c-Myc^Ser62^ intensity was quantified with Image J, and the mean intensity value of the control group was 100%, ****p < 0.0001. (C) BT-549 cells were treated with 10 μM AM879, BAY-850 or compound **19f** for 48h, the expression level of p-c-Myc Ser62 was detected by immunofluorescence. The nuclei were labelled with DAPI. Scale bar = 100 μm. (D) Relative p-c-Myc^Ser62^ intensity was quantified with Image J, and the mean intensity value of the control group was 100%. ****, *p* < 0.0001, compared with the control group. ^##^, *p* < 0.01, compared with the AM879 treated group. (E,F) BT-549 cells were treated with 5, 10, 20 μM AM879, BAY-850, or compound **19f** for 48h, the expression level of ATAD2, c-Myc, and p-c-Myc^Ser62^ were detected by western blot. ns, no significance. ***, *p* < 0.001; ****, *p* < 0.0001, compared with the control group.

Next, we investigated the effect of 19f on the biological function of ATAD2 in BT-549 cells. Considering that ATAD2 plays a tumour-promoting role mainly through activation of c-Myc (lack of sex hormone in TNBC)^3^, we first detected the phosphorylation level of c-Myc at Ser62 in BT-549 cells after compound **19f** treatment. Of note, *MYC* is a well-known proto-oncogene involved in the development and progression of many cancers^17^^,^^18^, including breast cancer^19^. The phosphorylation of c-Myc at ser62 can promote the stabilisation of c-Myc and enhance the *MYC*-mediated cancer-promoting pathway^18^^,^^20^. ATAD2 is a cofactor of *MYC* that binds to *MYC* and stimulates its transcriptional activity^21^^,^^22^. By examining the expression and phosphorylation of c-Myc, it was possible to determine whether the ATAD2 function was affected by compound **19f**. As shown in Figure 4(A), the immunofluorescence results showed that compound **19f** could reduce the fluorescence intensity of p-c-Myc^Ser62^ in a dose-dependent manner (Figure 4(A,B)). Compared to the ATAD2 inhibitor **BAY-850**, **AM879** we previously discovered, **19f** has a more significant impact on p-c-Myc^Ser62^ level than AM879, and a similar result to the **BAY-850** (Figure 4(C,D)). Subsequently, immunoblotting was used to detect the expression levels of ATAD2, c-Myc, and p-c-Myc in BT-549 cells treated with **BAY-850**, **AM879,** and compound **19f**, respectively. The results demonstrated that **AM879** and compound **19f** both have no significant effect on the expression of ATAD2, but inhibited the expression levels of c-Myc and p-c-Myc^Ser62^ in a dose-dependent manner, and compound **19f** had a more significant effect (Figure 4(E,F)). Interestingly, **BAY-850** inhibited the expression of ATAD2, which is consistent with previous reports in other literature that **BAY-850** reduced ATAD2 expression^23^. Similarly, **BAY-850** also reduced c-Myc expression and ser62 the phosphorylation at ser62 in a dose-dependent manner (Figure 4(E,F)). Taken together, compound **19f** impeded ATAD2 activity and c-Myc activation in TNBC cells.

### Compound 19f inhibits the proliferation of BT-549 cells

**Figure 5.**
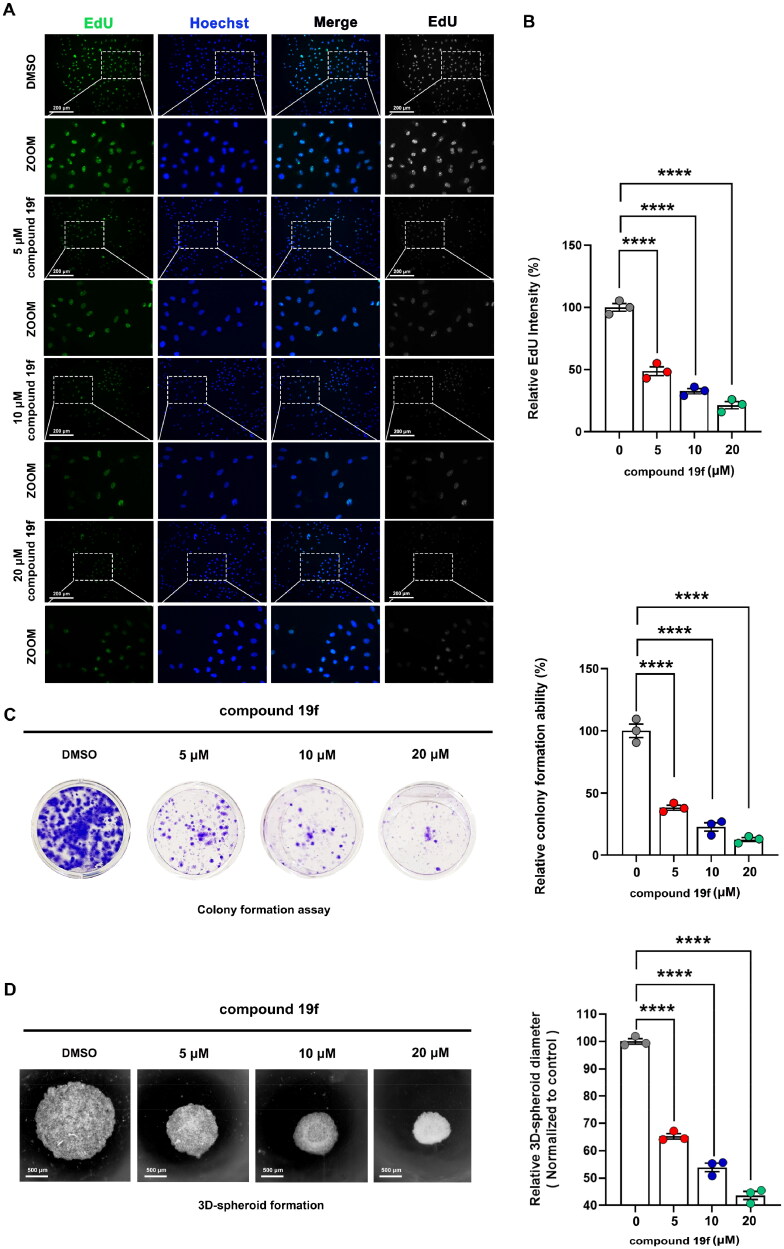
compound **19f** inhibits the proliferation of TNBC cells. (A) BT-549 cells were treated with 5, 10, and 20 μM compound 19f for 48h, the newly synthesised DNA was detected using EdU-488, the nuclei were labelled with Hoechst 33258. Scale bar = 200 μm. (B) Relative EdU-488 intensity was quantified with Image J, and the mean intensity value of the control group was 100%. ****, *p* < 0.0001. (C) Colony formation assays were used to detect the Long-term cell proliferation ability after compound 19f treatment. Relative colony formation ability was quantified with Image J, and the mean value of the control group was 100%. ****p < 0.0001, compared with the control group. (D) 3D spherical plates were used to investigate the effect of compounds on the formation of cell spheres. Relative 3D cell spheres diameter was quantified with Image J, and the mean value of the control group was 100%. ****p < 0.0001, compared with the control group.

**Figure 6.**
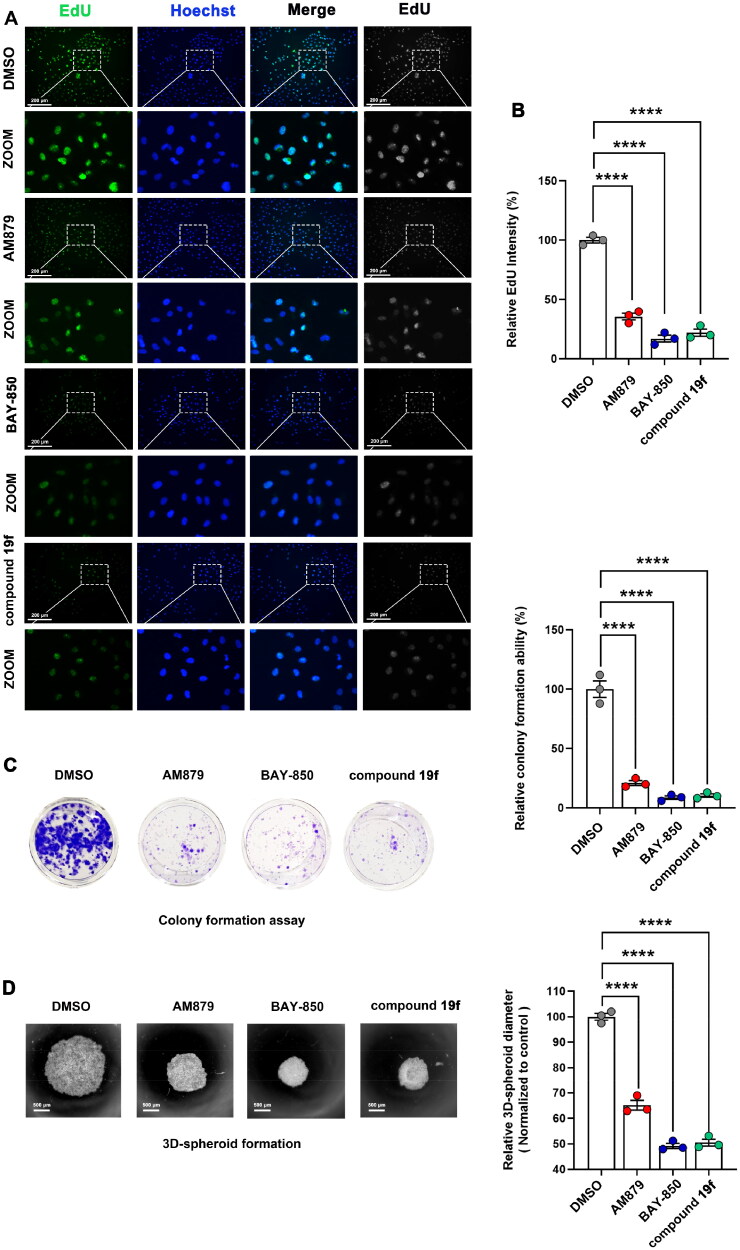
Comparison of the antiproliferative ability of compound **19f**, BAY-850 and AM879 on BT-549 cells. (A) BT-549 cells were treated with 10 μM compound **19f**, BAY-850 and AM879, the newly synthesised DNA was detected using EdU-488, and the nuclei were labelled with Hoechst 33258. Scale bar = 200 μm. (B) Relative EdU-488 intensity was quantified with Image J, and the mean intensity value of the control group was 100%. ****p < 0.0001, compared with the control group. (C) Colony formation assay were used to detect the long-term cell proliferation ability after compound **19f**, BAY-850 and AM879 treatment. Relative colony formation ability was quantified with Image J, and the mean value of the control group was 100%. ****p < 0.0001, compared with the control group. (D) 3D spherical plates were used to investigate the effect of compounds on the formation of cell spheres. Relative 3D cell spheres diameter was quantified with Image J, and the mean value of the control group was 100%. ****p < 0.0001, compared with the control group.

Next, we continue to examine the effect of compound **19f** on the proliferation ability of BT-549 cells. As shown in Figure 5(A), compound **19f** can reduce the fluorescence intensity of EdU-488 in a dose-dependent manner, indicating that compound **19f** can inhibit the synthesis of new DNA in BT-549 cells in a dose-dependent way, proving that compound **19f** can inhibit TNBC cell proliferation (Figure 5(B)). Next, we detected the effect of compound **19f** on the long-term cell proliferation ability of BT-549 cells. Compared with the control group, compound **19f** can inhibit the colony formation ability of BT-549 cells in a concentration-dependent manner (Figure 5(C)), suggesting that compound **19f** can also inhibit the long-term proliferation of BT-549 cells. In addition, we used 3D cell culture to investigate the effect of compound **19f** on the formation of cell spheres. It is worth noting that 3D cell culture technology can better simulate the *in vivo* state of the tumor^24^. The results indicated that compound **19f** dose-dependently reduce the diameter of the 3D tumour cell spheres (Figure 5(D)). Subsequently, we compared the effects of **BAY-850**, **AM879,** and compound **19f** on the proliferative ability of BT-549 cells. The results demonstrated that compound **19f** showed similar anti-proliferation ability to **BAY-850**, slightly better than **AM879** (Figure 6). Collectively, compound **19f** presents good anti-proliferative activity in BT-549 cells.

### 2.7. Compound 19f induces apoptosis in BT-549 cells

**Figure 7.**
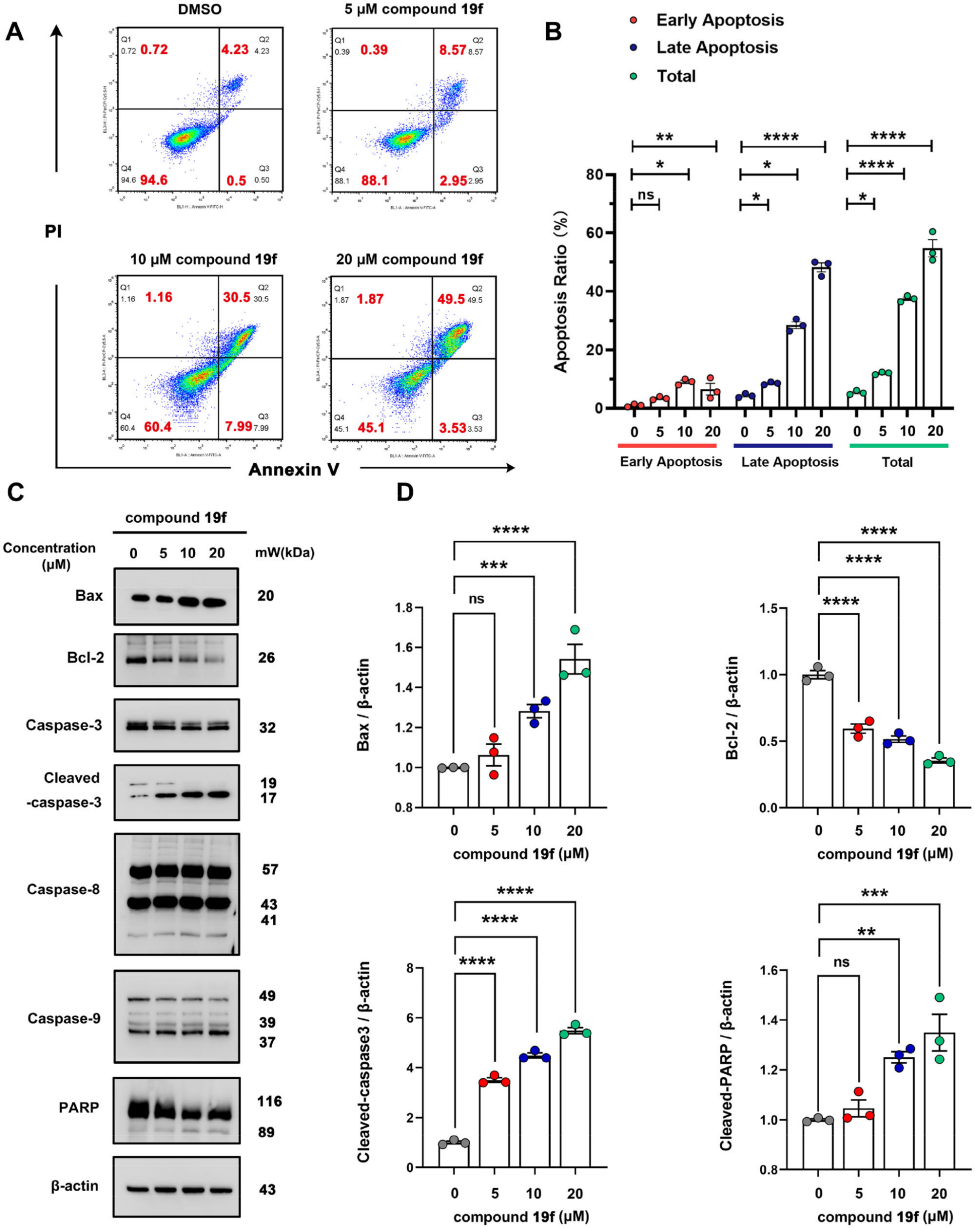
Compound **19f** induces apoptosis in BT-549 cells. (A) BT-549 cells were treated with 5, 10, 20 μM compound **19f** for 48h, the apoptosis ratio was measured by Annexin V-PI staining. (B) Relative apoptosis ratio was quantified with GraphPad Prism 8. *p < 0.05; **p < 0.01; ****, *p* < 0.0001, ns, no significance, compared with the control group. (C-D) BT-549 cells were treated with 5, 10, 20 μM compound **19f** for 48h, the expression level of Bax, Bcl-2, Caspase-3, Caspase-8, Caspase-9, and PARP were detected by western blot. ns, no significance; *p < 0.05; **p < 0.01; ***p < 0.001; ****p < 0.0001, compared with the control group.

Due to the close relationship between ATAD2 and apoptosis, inhibition of ATAD2 can induce apoptosis^8^. Therefore, we next tested whether compound **19f** can induce apoptosis in BT-549 cells. Interestingly, compound **19f** could induce obvious apoptosis in a dose-dependent manner, with an increase in early apoptosis and late apoptosis ratio (Figure 7(A,B)). Then, we investigated the expression of classic apoptotic marker protein. The results revealed that compound **19f** promoted the expression of Bax, the cleavage of caspase-3, caspase-9 and PARP. Meanwhile, the expression of Bcl-2 was significantly decreased after compound **19f** treatment. Intriguingly, the cleavage of caspase-8 was not changed significantly, indicating that death receptor-mediated apoptosis^25^ may be not involved in the regulation of BT-549 cell apoptosis by compound **19f** (Figure 7(C,D)). Thus, the inhibition of ATAD2 by compound **19f** induces apoptosis in BT-549 cells.

### Compound 19f inhibits the migration of BT-549 cells

**Figure 8.**
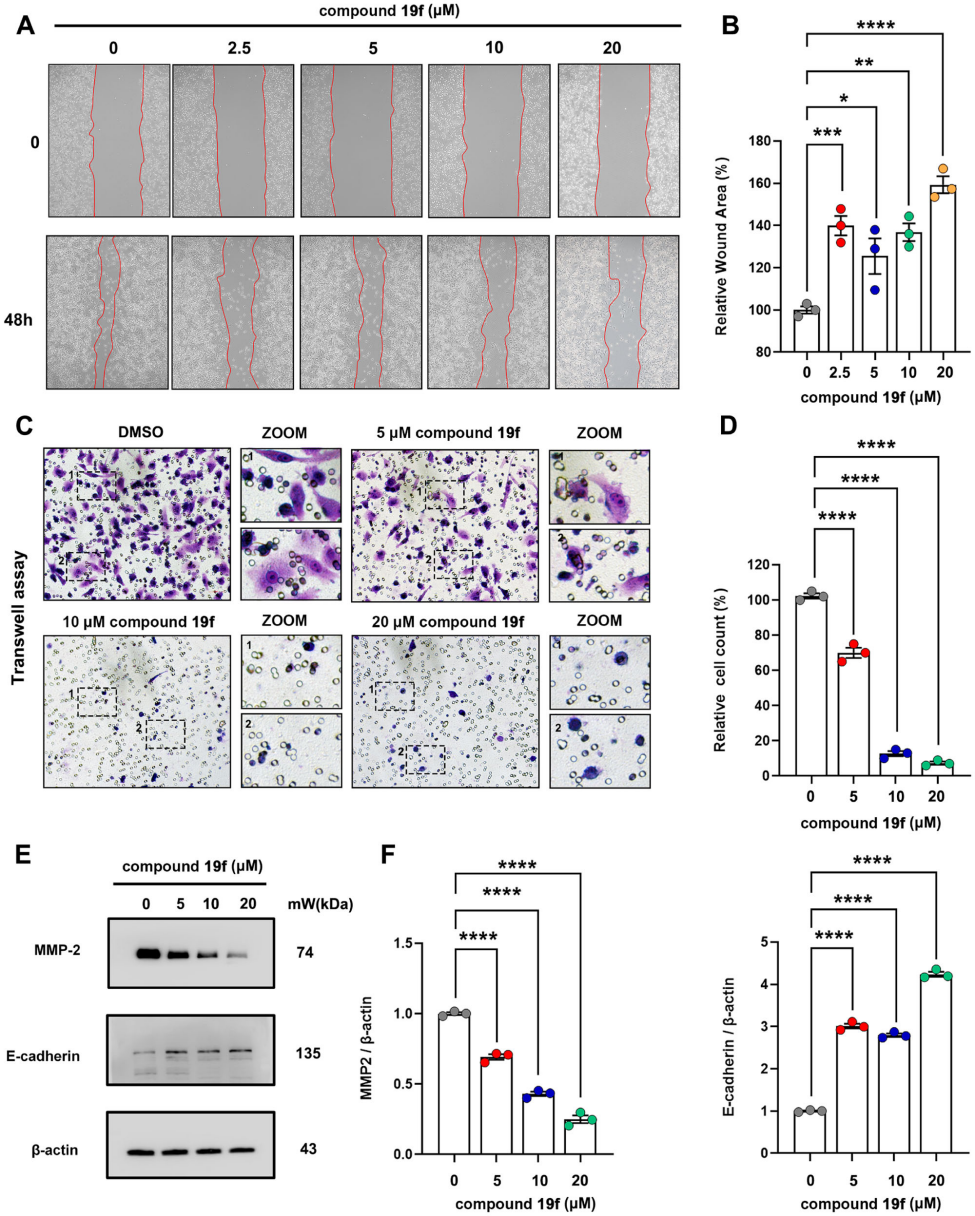
Compound **19f** inhibits BT-549 cells migration. (A) Wound healing assay of BT-549 cells treated with 2.5, 5, 10, 20 μM compound **19f** for 48h. (B) Relative wound areas were quantified with Image J, and the mean value of the control group was 100%. *p < 0.05; **p < 0.01; ***p < 0.001; ****, *p* < 0.0001, compared with the control group. (C) Transwell assay of BT-549 cells treated with 5, 10, 20 μM compound **19f** for 48h. (D) Relative migrated cell count was quantified with Image J, and the mean value of the control group was 100%. ****p < 0.0001, compared with the control group. (E–F) BT-549 cells were treated with 5, 10, 20 μM compound **19f** for 48h, the expression level of MMP-2 and E-cadherin were detected by western blot. ****p < 0.0001, compared with the control group.

To further evaluate the potential application of compound **19f** in TNBC, we subsequently examined its impact on the migration of TNBC cells. Not surprisingly, the migration ability of BT-549 cells was significantly decreased after compound **19f** treatment. The wound healing assay revealed that compound **19f** increased the relative wound area in a dose-dependent manner (Figure 8(A,B)). Meanwhile, compound **19f** also decreased the migrated cell number in a dose-dependent manner (Figure 8(C,D)). Next, we checked the expression of MMP-2 and E-cadherin, which are two key migration markers, and found that compound **19f** up-regulated the expression of E-cadherin and down-regulated MMP-2 (Figure 8(C,D)). Taken together, compound **19f** exhibits good anti-migration activity *in vitro*.

## Conclusion

In summary, we have successfully designed and synthesised a series of theophylline derivatives as novel ATAD2 inhibitors through fragment-based screening and scaffold growth strategy. Compound **19f** is identified as a potent ATAD2 inhibitor with an IC_50_ value of 0.27 μM, presenting a significant elevation of 87 folds in affinity for ATAD2 bromodomain compared to the lead theophylline. Additionally, compound **19f** may adopt a combination of the classic and atypical binding mode for ATAD2. Compound **19f** significantly inhibited BT-549 cells proliferation with an IC_50_ value of 5.43 μM, although the antiproliferative activity of compound **19f** is slightly weaker than that of the positive drug (**BAY-850**), it is the first compound with the particular binding mode inhibitor to show cellular antiproliferative potency. In addition, compound **19f** could impede ATAD2 activity and c-Myc activation, and induce significant apoptosis, and anti-migration in BT-549 cells. Collectively, compound **19f** is a promising candidate lead for the development of novel potent ATAD2 inhibitors.

## Experimental

### Chemistry

All commercially purchased reagents/solvents are used directly without any further processing. The NMR spectra were detected by AVANCE NEO 600 (Bruker Switzerland AG). ESI-MS spectra were collected by TSQ Altis™ Plus. The purities (HPLC) of all compounds were more than 95%. HPLC device: Agilent 1260, Column: Phenomenex Luna 5 μm C18 (2) 100 A, 250 × 4.6 mm S/N: H19-293790. The mobile phase is different concentrations of methanol in water with a flow rate of 1.0 ml/min.

### Synthesis

#### Synthesis of intermediate 9

To a solution of theophylline (**7**, 10 mmol) in DMF (15 ml), K_2_CO_3_ (30 mmol), and the resulting mixture was allowed to warm up to 60 °C for 30 min, and ethyl bromoacetate (**8**, 12 mmol) were added. Upon completion the reaction, the residue was added 50 ml water, and extracted with ethyl acetate (3 × 50 ml). The combined organic layers were washed with saturated aqueous sodium bicarbonate and brine and then dried over anhydrous sodium sulphate. After removing the solvent under reduced pressure to give the colourless oil. The crude oil was purified by silica sel flash chromatography (dichloromethane/methanol 50:1) as a colourless oil, yield 89%. *Ethyl 2–(1,3-dimethyl-2,6-dioxo-1,2,3,6-tetrahydro-9H-purin-9-yl) acetate* (***9***) ^1^H-NMR (600 MHz, DMSO-*d6*), δ(ppm): 8.06 (1H, s), 5.17 (2H, s), 4.17 (2H, q, *J* = 7.1 Hz), 3.44 (3H, s), 3.20 (3H, s), 1.22 (3H, t, *J* = 7.1 Hz); ^13^C-NMR (150 MHz, DMSO-*d6*), δ(ppm): 168.1, 154.8, 151.4, 148.4, 143.6, 106.7, 61.8, 47.6, 29.9, 27.9, 14.4.

#### Synthesis of intermediate 10

To a solution of **9** (10 mmol) in 75% MeOH (15 ml), LiOH (30 mmol), and the resulting mixture was stirred for 8 h. Upon completion, the residue was evaporated to remove excess MeOH and acidified with 1 M HCl to pH = 3. The resulting precipitate was collected by filtration to yield the intermediate **10** as a white solid and used directly for the next reaction without purification, yield 89%. *2–(1,3-dimethyl-2,6-dioxo-1,2,3,6-tetrahydro-9H-purin-9-yl)acetic acid* (***9***) ^1^H-NMR (600 MHz, DMSO-*d6*), δ(ppm): 13.29 (1H, br. s), 8.04 (1H, br. s), 5.08 (2H, s), 3.44 (3H, s), 3.20 (3H, s); ^13^C-NMR (150 MHz, DMSO-*d6*), δ(ppm): 169.4, 154.8, 151.4, 148.3, 143.6, 106.8, 47.6, 29.9, 27.9.

#### General procedure synthesis of intermediates 13

To a solution of intermediate **11** (5 mmol), secondary amine derivatives (**12**, 5 mmol) and DIEA (10 mmol) in DMF (10 ml), and HATU was added at room temperature for 24 h. The mixture was added water (50 ml), extracted with ethyl acetate (30 ml) three times. The combined organic layers were washed with water, saturated aqueous sodium bicarbonate and brine, and then dried over anhydrous sodium sulphate. After removing the solvent under reduced pressure, the crude product was purified by flash chromatography on silica gel, eluting with dichloromethane/methanol (1–2%), yield 83–95%.

##### *(3-nitrophenyl)(thiomorpholino)methanone (13a)* light yellow solid, yield 83%

^1^H-NMR (600 MHz, CDCl_3_), δ (ppm): 8.30 (1H, d, *J* = 8.1 Hz), 8.25 (1H, m), 7.73 (1H, dt, *J* = 7.6, 1.3 Hz), 7.64 (1H, t, *J* = 7.6 Hz), 4.05 (2H, br. s), 3.68 (2H, br. s), 2.76 (2H, br. s), 2.61 (2H, br. s); ^13^C-NMR (100 MHz, CDCl_3_), δ (ppm): 168.0, 148.1, 137.3, 132.8, 129.9, 124.5, 122.0, 50.1, 44.8, 27.8.

##### *(4-methyl-3-nitrophenyl)(thiomorpholino)methanone (13b)* light yellow solid, yield 89%

^1^H-NMR (600 MHz, CDCl_3_), δ (ppm): 8.01 (1H, d, *J* = 1.5 Hz), 7.54 (1H, dd, *J* = 7.8, 1.5 Hz), 7.42 (1H, d, *J* = 7.8 Hz), 4.02 (2H, br. s), 3.71 (2H, br. s), 2.69 (4H, br. s), 2.64 (3H, s); ^13^C-NMR (150 MHz, CDCl_3_), δ (ppm): 168.1, 149.0, 135.3, 134.7, 133.3, 131.2, 123.3, 50.2, 44.7, 27.7, 20.3.

##### *(4-methoxy-3-nitrophenyl)(thiomorpholino)methanone (13c)* light yellow solid, yield 92%

^1^H-NMR (600 MHz, CDCl_3_), δ (ppm): 7.93 (1H, d, *J* = 2.1 Hz), 7.65 (1H, dd, *J* = 8.6, 2.1 Hz), 7.16 (1H, d, *J* = 8.6 Hz), 4.01 (3H, s), 3.91 (4H, br. s), 2.68 (4H, br. s); ^13^C-NMR (150 MHz, CDCl_3_), δ (ppm): 168.1, 154.0,139.1, 133.2, 127.6, 124.9, 113.7, 56.8, 50.3, 45.1, 27.6.

##### *(1,1-dioxidothiomorpholino)(3-nitrophenyl)methanone (13d)* light yellow solid, yield 87%

^1^H-NMR (600 MHz, CDCl_3_), δ (ppm): 8.41 (1H, br. s), 8.32 (1H, dd, *J* = 8.2, 1.4 Hz), 7.93 (1H, d, *J* = 7.7 Hz), 7.77 (1H, d, *J* = 8.0 Hz), 4.03 (4H, br. s), 3.67 (4H, br. s), 3.26 (4H, br. s); ^13^C-NMR (150 MHz, CDCl_3_), δ (ppm): 167.7, 148.2, 137.2, 133.6, 130.7, 124.9, 122.4, 51.0, 46.0.

##### *(1,1-dioxidothiomorpholino)(4-methyl-3-nitrophenyl)methanone (13e)* light yellow solid, yield 94%

^1^H-NMR (600 MHz, CDCl_3_), δ (ppm): 8.01 (1H, d, *J* = 1.5 Hz), 7.54 (1H, dd, *J* = 7.8, 1.5 Hz), 7.42 (1H, d, *J* = 7.8 Hz), 3.86 (4H, br. s), 3.26 (4H, br. s), 2.30 (3H, s); ^13^C-NMR (150 MHz, CDCl_3_), δ (ppm): 167.8, 153.3, 139.2, 133.6, 127.5, 124.6, 114.9, 57.4, 51.1, 20.3.

##### *(1,1-dioxidothiomorpholino)(4-methoxy-3-nitrophenyl)methanone (13f)* light yellow solid, yield 86%

^1^H-NMR (600 MHz, CDCl_3_), δ (ppm): 8.07 (1H, d, *J* = 2.1 Hz), 7.79 (1H, dd, *J* = 8.6, 2.1 Hz), 7.45 (1H, d, *J* = 8.6 Hz), 3.97 (3H, s), 3.86 (4H, br. s), 3.26 (4H, br. s); ^13^C-NMR (150 MHz, CDCl_3_), δ (ppm): 167.8, 153.3, 139.2, 133.6, 127.5, 124.6, 114.9, 57.4, 51.1.

##### *Morpholino(3-nitrophenyl)methanone (13 g)* light yellow solid, yield 89%

^1^H-NMR (600 MHz, CDCl_3_), δ (ppm): 7.90 (1H, d, *J* = 1.9 Hz), 7.82 (1H, d, *J* = 8.2 Hz), 7.49 (1H, dd, *J* = 8.2, 1.9 Hz), 3.74 (6H, br. s), 3.48 (2H, br. s); ^13^C-NMR (150 MHz, CDCl_3_), δ (ppm): 166.7, 149.7, 135.7, 135.5, 131.6, 124.5, 116.2, 66.6, 48.2, 42.7.

##### *(4-methyl-3-nitrophenyl)(morpholino)methanone (13h)* light yellow solid, yield 86%

^1^H-NMR (600 MHz, CDCl_3_), δ (ppm): 8.04 (1H, d, *J* = 1.5 Hz), 7.58 (1H, dd, *J* = 7.8, 1.5 Hz), 7.43 (1H, d, *J* = 7.8 Hz), 3.74 (6H, br. s), 3.50 (2H, br. s), 2.65 (3H, s); ^13^C-NMR (150 MHz, CDCl_3_), δ (ppm): 167.8, 149.0, 135.4, 134.3, 133.2, 131.5, 123.6, 66.7, 48.2, 42.7, 20.3.

##### *(4-methoxy-3-nitrophenyl)(morpholino)methanone (13i)* light yellow solid, yield 86%

^1^H-NMR (600 MHz, CDCl_3_), δ (ppm): 7.96 (1H, d, *J* = 1.9 Hz), 7.68 (1H, dd, *J* = 8.6, 1.9 Hz), 7.16 (1H, d, *J* = 8.6 Hz), 4.02 (3H, s), 3.73 (6H, br. s), 3.66 (2H, br. s); ^13^C-NMR (150 MHz, CDCl_3_), δ (ppm): 167.7, 154.0, 139.1, 133.5, 127.2, 125.1, 113.6, 66.7, 56.8, 48.2, 42.7.

##### *(4-chloro-3-nitrophenyl)(morpholino)methanone (13j)* light yellow solid, yield 78%

^1^H-NMR (600 MHz, CDCl_3_), δ (ppm): 7.95 (1H, s), 7.64 (1H, d, *J* = 8.3 Hz), 7.58 (1H, d, *J* = 8.3 Hz), 3.77 (2H, br. s), 3.69 (2H, br. s), 3.46 (2H, br. s); ^13^C-NMR (150 MHz, CDCl_3_), δ (ppm): 166.7, 147.8, 135.0, 132.3, 131.8, 128.7, 124.6, 66.7, 48.2, 42.7.

##### *(2-fluoro-3-nitrophenyl)(morpholino)methanone (13k)* light yellow solid, yield 90%

^1^H-NMR (600 MHz, CDCl_3_), δ (ppm): 8.12 (1H, t, *J* = 7.6 Hz), 7.70 (1H, t, *J* = 6.7 Hz), 7.40 (1H, t, *J* = 7.9 Hz), 3.83 (2H, br. s), 3.80 (2H, br. s), 3.68 (2H, br. s), 3.35 (2H, br. s); ^13^C-NMR (150 MHz, CDCl_3_), δ (ppm): 162.7, 137.5, 134.6, 134.6, 127.2, 127.2, 126.9, 126.7, 125.2, 125.1, 66.6, 47.4, 42.6.

#### Synthesis of compounds 14

To a solution of intermediate **13** (3 mmol), iron powder (12 mmol) and ammonium chloride (NH_4_Cl, 15 mmol) in 95% ethanol (20 ml), and a catalytic amount of glacial acetic acid were added under strenuous stirring, and then the mixture was allowed to warm up to reflux for 4 h. The additional iron powder was removed by filtration, and the filtrate was concentrated to dryness to give the crude product and used directly for the next reaction without purification, yield 87–97%.

##### *(3-aminophenyl)(thiomorpholino)methanone (14a)* light brown solid, yield 88%

^1^H-NMR (600 MHz, CDCl_3_), δ(ppm): 7.17 (1H, t, *J* = 7.7 Hz), 6.71 (1H, t, *J* = 7.2 Hz), 6.67 (2H, s), 3.99 (2H, brs), 3.84 (2H, brs), 3.67 (2H, brs), 2.72 (2H, brs), 2.55 (2H, brs); ^13^C-NMR (150 MHz, CDCl_3_), δ(ppm): 170.9, 146.7, 136.9, 129.5, 116.4, 116.2, 113.1, 50.0, 44.4, 28.1, 27.4.

##### *(3-amino-4-methylphenyl)(thiomorpholino)methanone (14b)* light brown solid, yield 94%

^1^H-NMR (600 MHz, CDCl_3_), δ(ppm): 7.05 (1H, d, *J* = 7.5 Hz), 6.68 (1H, brs Hz), 6.65 (1H, d, *J* = 7.5 Hz), 3.96 (2H, brs), 3.84 (2H, brs), 3.70 (2H, brs), 2.66 (4H, brs); ^13^C-NMR (150 MHz, CDCl_3_), δ(ppm): 171.0, 144.8, 134.5, 130.4, 123.9, 116.6, 113.1, 50.1, 44.5, 29.6, 27.7, 17.2.

##### *(3-amino-4-methoxyphenyl)(thiomorpholino)methanone (14c)* light brown solid, yield 91%

^1^H-NMR (600 MHz, CDCl_3_), δ(ppm): 6.77 − 6.73 (3H, m), 3.89 (2H, br. s), 3.88 (4H, br. s), 3.87 (3H, s), 2.64 (4H, br. s); ^13^C-NMR (150 MHz, CDCl_3_), δ(ppm): 171.1, 148.3, 136.3, 128.3, 117.3, 113.5, 109.8, 55.5, 49.8, 44.9, 27.8, 27.8.

##### *(3-aminophenyl)(1,1-dioxidothiomorpholino)methanone (14d)* light brown solid, yield 96%

^1^H-NMR (600 MHz, CDCl_3_), δ(ppm): 7.22 (1H, t, *J* = 7.8 Hz), 6.73 (1H, s), 6.78 (1H, d, *J* = 7.8 Hz), 6.75 (1H, d, *J* = 7.5 Hz), 6.72 (1H, br. s), 4.10 (4H, br. s), 3.06 (4H, br. s); ^13^C-NMR (150 MHz, CDCl_3_), δ(ppm): 171.2, 147.0, 135.0, 129.8, 117.0, 116.3, 113.0, 52.1, 45.8, 29.7.

##### *(3-amino-4-methylphenyl)(1,1-dioxidothiomorpholino)methanone (14e)* light brown solid, yield 85%

^1^H-NMR (600 MHz, CDCl_3_), δ(ppm): 7.10 (1H, d, *J* = 7.6 Hz), 6.73 (1H, s), 6.71 (1H, d, *J* = 7.6 Hz), 4.9 (4H, br. s), 3.06 (4H, br. s), 2.19 (3H, s); ^13^C-NMR (150 MHz, CDCl_3_), δ(ppm): 171.4, 145.2, 132.6, 130.7, 124.9, 116.5, 113.0, 52.0, 45.9, 41.1, 17.3.

##### *(3-amino-4-methoxyphenyl)(1,1-dioxidothiomorpholino)methanone (14f)* light brown solid, yield 81%

^1^H-NMR (600 MHz, CDCl_3_), δ(ppm): 6.80 (3H, s), 4.10 (4H, br. s), 3.90 (3H, s), 3.06 (4H, br. s); ^13^C-NMR (150 MHz, CDCl_3_), δ(ppm): 171.5, 149.0, 136.6, 126.3, 117.5, 113.4, 109.8, 55.6, 52.0.

##### *(3-amino-4-methoxyphenyl)(morpholino)methanone (14 g)* light brown solid, yield 80%

^1^H-NMR (400 MHz, CDCl_3_), δ(ppm): 7.15 (1H, t, *J* = 7.7 Hz), 6.72 − 6.70 (3H, m), 3.75 (4H, br. s), 3.60 (2H, br. s), 3.47 (4H, m); ^13^C-NMR (100 MHz, CDCl_3_), δ(ppm): 170.6, 146.6, 136.3, 129.4, 116.7, 116.4, 113.5, 66.9, 48.1, 42.4.

##### *(3-amino-4-methylphenyl)(morpholino)methanone (14h)* light brown solid, yield 96%

^1^H-NMR (600 MHz, CDCl_3_), δ(ppm): 7.05 (1H, d, *J* = 7.5 Hz), 6.71 (1H, d, *J* = 1.4 Hz), 6.68 (1H, dd, *J* = 7.5, 1.4 Hz), 3.71 − 3.50 (10H, m), 2.17 (3H, s); ^13^C-NMR (150 MHz, CDCl_3_), δ (ppm): 1170.7, 144.8, 134.1, 130.4, 124.1, 116.9, 113.4, 66.9, 48.2, 42.5, 17.2.

##### *(3-amino-4-methoxyphenyl)(morpholino)methanone (14i)* light brown solid, yield 94%

^1^H-NMR (600 MHz, CDCl_3_), δ (ppm): 6.78 (1H, d, *J* = 8.1 Hz), 6.77 (2H, br. s), 3.89 (2H, br. s), 3.87 (3H, s), 3.67 (8H, m); ^13^C-NMR (150 MHz, CDCl_3_), δ (ppm): 170.7, 148.4, 136.3, 127.9, 117.6, 113.8, 109.7, 66.9, 55.5.

##### *(3-amino-4-chlorophenyl)(morpholino)methanone (14j)* light brown solid, yield 92%

^1^H-NMR (600 MHz, CDCl_3_), δ(ppm): 7.26 (1H, d, *J* = 8.0 Hz), 6.80 (1H, d, *J* = 1.8 Hz), 6.67 (1H, dd, *J* = 8.0, 1.8 Hz), 4.23 (2H, br. s), 3.72 − 3.45 (8H, m); ^13^C-NMR (150 MHz, CDCl_3_), δ (ppm): 169.7, 143.3, 134.8, 129.4, 120.5, 117.0, 114.3, 66.8, 48.1, 42.5.

##### *(3-amino-2-fluorophenyl)(morpholino)methanone (14k)* light brown solid, yield 97%

^1^H-NMR (600 MHz, CDCl_3_), δ(ppm): 6.97 (1H, t, *J* = 7.8 Hz), 6.81 (1H, t, *J* = 8.3 Hz), 6.68 (1H, t, *J* = 6.9 Hz), 3.83 (2H, br. s), 3.81 (2H, br. s), 3.77 (2H, d, *J* = 4.2 Hz), 3.64 (2H, br. s), 3.36 (2H, br. s); ^13^C-NMR (150 MHz, CDCl_3_), δ (ppm): 165.5, 147.7, 146.1, 134.8, 134.8, 125.0, 125.0, 123.7, 123.6, 117.8, 117.8, 117.3, 66.9, 66.8, 47.4, 42.3.

#### Synthesis of intermediate 15a–k

To a solution of intermediate **10** (1 mmol), intermediate **14** (1 mmol), and DIEA (2 mmol) in DMF (10 ml), and HATU (1 mmol) was added at room temperature for 24 h. The mixture was added water (50 ml), and extracted with ethyl acetate (30 ml) three times. The combined organic layers were washed with water, saturated aqueous sodium bicarbonate and brine, and then dried over anhydrous sodium sulphate. After removing the solvent under reduced pressure, the crude product was purified by flash chromatography on silica gel, eluting with dichloromethane/methanol (2–4%), yield 78–93%.

##### 2–(1,3-dimethyl-2,6-dioxo-1,2,3,6-tetrahydro-9H-purin-9-yl)-N-(3-(thiomorpholine-4-carbonyl)phenyl)acetamide (15a)

White solid, m.p. 246–248 °C, yield 79%. ^1^H-NMR (600 MHz, DMSO-*d6*), δ (ppm): 10.58 (1H, s), 8.07 (1H, s), 7.67 (1H, s), 7.58 (1H, d, *J* = 8.1 Hz), 7.40 (1H, t, *J* = 7.8 Hz), 7.09 (1H, d, *J* = 7.8 Hz), 5.22 (2H, s), 3.85 (2H, br. s), 3.54 (2H, br. s), 3.46 (3H, s), 3.19 (3H, s), 2.66 (2H, br. s), 2.58 (2H, br. s); ^13^C-NMR (150 MHz, DMSO-*d6*), δ (ppm): 169.4, 165.6, 154.9, 151.4, 148.4, 144.2, 139.1, 137.1, 129.7, 122.1, 120.3, 117.6, 106.9, 50.0, 49.2, 44.3, 29.9, 27.9, 27.4, 27.0; HRMS (ESI)+ calculated for C_20_H_22_N_6_O_4_S, [M + H]^+^: m/z 443.1496, found 443.1504.

##### 2–(1,3-dimethyl-2,6-dioxo-1,2,3,6-tetrahydro-9H-purin-9-yl)-N-(2-methyl-5-(thiomorpholine-4-carbonyl)phenyl)acetamide (15b)

White solid, m.p. 251–252 °C, yield 86%. ^1^H-NMR (600 MHz, DMSO-*d6*), δ (ppm): 9.82 (1H, s), 8.09 (1H, s), 8.08 (1H, s), 7.48 (1H, s), 7.30 (1H, d, *J* = 7.8 Hz), 7.12 (1H, d, *J* = 6.9 Hz), 5.25 (2H, s), 3.82 (2H, br. s), 3.56 (2H, br. s), 3.46 (3H, s), 3.22 (3H, s), 2.63 (2H, br. s), 2.56 (2H, br. s), 2.29 (3H, s); ^13^C-NMR (150 MHz, DMSO-*d6*), δ (ppm): 169.4, 165.9, 154.9, 151.5, 148.4, 144.1, 136.0, 134.1, 133.2, 131.0, 124.0, 123.3, 107.0, 49.1, 29.9, 27.9, 27.0, 18.1; HRMS (ESI)+ calculated for C_21_H_24_N_6_O_4_S, [2M + H]^+^: m/z 913.3244, found [2M + H]^+^ 913.3248.

##### 2–(1,3-dimethyl-2,6-dioxo-1,2,3,6-tetrahydro-9H-purin-9-yl)-N-(2-methoxy-5-(thiomorpholine-4-carbonyl)phenyl)acetamide (15c)

White solid, m.p. 257–258 °C, yield 90%. ^1^H-NMR (600 MHz, DMSO-*d6*), δ (ppm): 9.88 (1H, s), 8.07 (1H, s), 8.08 (1H, s), 7.17 − 7.12 (2H, m), 5.29 (2H, s), 3.92 (3H, s), 3.69 (4H, br. s), 3.46 (3H, s), 3.20 (3H, s), 2.59 (4H, br. s); ^13^C-NMR (150 MHz, DMSO-*d6*), δ (ppm): 169.6, 166.0, 162.7, 154.9, 151.4, 150.5, 148.4, 144.2, 128.1, 127.0, 123.9, 120.5, 111.5, 106.9, 56.4, 55.3, 49.4, 29.9, 27.9; HRMS (ESI)+ calculated for C_21_H_24_N_6_O_5_S, [M + H]^+^: m/z 473.1602, found 473.1627.

##### 2–(1,3-dimethyl-2,6-dioxo-1,2,3,6-tetrahydro-9H-purin-9-yl)-N-(3–(1,1-dioxidothiomorpholine-4-carbonyl)phenyl)acetamide (15d)

White solid, m.p. 224–226 °C, yield 95%. ^1^H-NMR (600 MHz, DMSO-*d6*), δ (ppm):10.59 (1H, s), 8.07 (1H, s), 7.75 (1H, s), 7.60 (1H, d, *J* = 7.6 Hz), 7.42 (1H, t, *J* = 7.9 Hz), 7.21 (1H, d, *J* = 7.5 Hz), 5.75 (1H, s), 5.22 (2H, s), 4.00 (2H, br. s), 3.70 (2H, br. s), 3.45 (3H, s), 3.25 (4H, br. s), 3.22 (4H, br. s), 3.20 (3H, s); ^13^C-NMR (150 MHz, DMSO-*d6*), δ (ppm): 169.4, 165.6, 154.9, 151.4, 148.4, 144.2, 139.1, 136.1, 129.6, 122.3, 120.7, 118.0, 106.9, 55.3, 51.2, 49.2, 29.9, 27.9; HRMS (ESI)+ calculated for C_20_H_22_N_6_O_6_S, [M + Na]^+^: m/z 497.1214, found 497.1202.

##### 2–(1,3-dimethyl-2,6-dioxo-1,2,3,6-tetrahydro-9H-purin-9-yl)-N-(5–(1,1-dioxidothiomorpholine-4-carbonyl)-2-methylphenyl)acetamide (15e)

White solid, m.p. 231–233 °C, yield 92%. ^1^H-NMR (600 MHz, DMSO-*d6*), δ (ppm): 9.93 (1H, s), 8.09 (1H, s), 7.58 (1H, s), 7.32 (1H, d, *J* = 7.6 Hz), 7.22 (1H, d, *J* = 7.6 Hz), 5.26 (2H, s), 3.96 (2H, s), 3.70 (2H, br. s), 3.45 (3H, s), 3.22 (4H, br. s), 3.21 (3H, s), 2.30 (3H, s); ^13^C-NMR (150 MHz, DMSO-*d6*), δ (ppm): 169.5, 165.8, 154.9, 151.5, 148.4, 144.1, 136.1, 133.7, 133.1, 131.0, 124.2, 123.6, 107.0, 51.2, 49.0, 29.9, 27.9, 18.2; HRMS (ESI)+ calculated for C_21_H_24_N_6_O_6_S, [M-H]^-^: m/z 487.1405, found 487.1404.

##### 2–(1,3-dimethyl-2,6-dioxo-1,2,3,6-tetrahydro-9H-purin-9-yl)-N-(5–(1,1-dioxidothiomorpholine-4-carbonyl)-2-methoxyphenyl)acetamide (15f)

White solid, 247–248 °C, yield 95%. ^1^H-NMR (600 MHz, DMSO-*d6*), δ (ppm): 9.89 (1H, s), 8.12 (1H, s), 8.07 (1H, s), 7.26 (1H, dd, *J* = 8.3, 1.5 Hz), 7.15 (1H, d, *J* = 8.3 Hz), 5.29 (2H, s), 3.94 (3H, s), 3.85 (4H, br. s), 3.46 (3H, s), 3.22 (4H, br. s), 3.20 (3H, s); ^13^C-NMR (150 MHz, DMSO-*d6*), δ (ppm): 169.6, 166.0, 154.9, 151.4, 150.8, 148.4, 144.2, 127.0, 124.2, 120.9, 111.4, 106.9, 56.4, 51.2, 49.4, 40.4, 29.9, 27.9; HRMS (ESI)^-^ calculated for C_21_H_24_N_6_O_7_S, [M-H]^-^: m/z 503.1354, found 503.1355.

##### 2–(1,3-dimethyl-2,6-dioxo-1,2,3,6-tetrahydro-9H-purin-9-yl)-N-(3-(morpholine-4-carbonyl)phenyl)acetamide (15 g)

White solid, m.p. 266–268 °C, yield 96%. ^1^H-NMR (600 MHz, DMSO-*d6*), δ (ppm): 10.58 (1H, s), 8.08 (1H, s), 7.67 (1H, s), 7.59 (1H, d, *J* = 8.2 Hz), 7.40 (1H, t, *J* = 7.8 Hz), 7.11 (1H, d, *J* = 7.6 Hz), 5.22 (2H, s), 3.61 (6H, br. s), 3.54 (2H, s), 3.45 (3H, s), 3.19 (3H, s); ^13^C-NMR (150 MHz, DMSO-*d6*), δ (ppm): 169.1, 165.6, 154.9, 151.4, 148.4, 144.2, 139.1, 136.6, 129.6, 122.4, 120.4, 118.0, 106.9, 66.5, 55.3, 49.2, 48.1, 42.5, 29.9, 27.9; HRMS (ESI)+ calculated for C_20_H_22_N_6_O_5_, [M + H]^+^: m/z 427.1724, found 427.1729.

##### 2–(1,3-dimethyl-2,6-dioxo-1,2,3,6-tetrahydro-9H-purin-9-yl)-N-(2-methyl-5-(morpholine-4-carbonyl)phenyl)acetamide (15h)

White solid, m.p. 270–271 °C, yield 88%. ^1^H-NMR (600 MHz, DMSO-*d6*), δ (ppm): 9.83 (1H, s), 8.09 (1H, s), 8.08 (1H, s), 7.49 (1H, s), 7.30 (1H, d, *J* = 7.8 Hz), 7.14 (1H, d, *J* = 7.6 Hz), 5.25 (2H, s), 3.57 (8H, br. s), 3.45 (3H, s), 3.22 (3H, s), 2.29 (3H, s); ^13^C-NMR (100 MHz, CDCl_3_), δ (ppm): 169.0, 165.8, 154.9, 151.5, 148.4, 144.1, 136.0, 133.6, 133.4, 130.9, 124.4, 123.8, 107.0, 66.5, 49.0, 29.9, 27.9, 18.1; HRMS (ESI)+ calculated for C_21_H_24_N_6_O_5_, [M + H]^+^: m/z 441.1881, found 441.11885.

##### 2–(1,3-dimethyl-2,6-dioxo-1,2,3,6-tetrahydro-9H-purin-9-yl)-N-(2-methoxy-5-(morpholine-4-carbonyl)phenyl)acetamide (15i)

White solid, m.p. 278–279 °C, yield 94%. ^1^H-NMR (600 MHz, DMSO-*d6*), δ (ppm): 9.87 (1H, s), 8.07 (1H, d, *J* = 8.8 Hz), 7.19 (1H, d, *J* = 8.7 Hz), 7.13 (1H, d, *J* = 8.7 Hz), 5.29 (2H, s), 3.93 (3H, s), 3.55 (4H, br. s), 3.46 (4H, br. s), 3.45 (3H, s), 3.21 (3H, s); ^13^C-NMR (100 MHz, CDCl_3_), δ (ppm): 169.2, 166.0, 154.9, 151.5, 150.6, 148.4, 144.2, 127.6, 127.0, 124.3, 120.9, 111.4, 106.9, 66.5, 56.4, 49.4, 29.9, 27.9; HRMS (ESI)^-^ calculated for C_21_H_24_N_6_O_6_, [M-H]^-^: m/z 455.1685, found 455.1674.

##### N-(2-chloro-5-(morpholine-4-carbonyl)phenyl)-2–(1,3-dimethyl-2,6-dioxo-1,2,3,6-tetrahydro-9H-purin-9-yl)acetamide (15j)

White solid, m.p. 262–264 °C, yield 91%. ^1^H-NMR (600 MHz, DMSO-*d6*), δ (ppm): 10.16 (1H, s), 8.09 (1H, s), 7.81 (1H, d, *J* = 1.3 Hz), 7.60 (1H, d, *J* = 8.2 Hz), 7.24 (1H, dd, *J* = 8.2, 1.3 Hz), 5.31 (2H, s), 3.59 (6H, br. s), 3.52 (2H, s), 3.45 (3H, s), 3.21 (3H, s); ^13^C-NMR (100 MHz, CDCl_3_), δ (ppm): 169.8, 166.3, 154.9, 151.4, 148.3, 144.2, 138.8, 138.6, 131.1, 131.0, 128.7, 128.5, 128.0, 127.5, 106.9, 57.0, 48.5, 40.4, 34.3, 29.9, 27.9; HRMS (ESI)^-^ calculated for C_20_H_21_ClN_6_O_5_, [M-H]^-^: m/z 459.1189, found 459.1181.

##### 2–(1,3-dimethyl-2,6-dioxo-1,2,3,6-tetrahydro-9H-purin-9-yl)-N-(2-fluoro-3-(morpholine-4-carbonyl)phenyl)acetamide (15k)

White solid, m.p. 265–267 °C, yield 93%. ^1^H-NMR (600 MHz, DMSO-*d6*), δ (ppm): 10.39 (1H, s), 8.08 (1H, s), 7.98 (1H, t, *J* = 7.8 Hz), 7.28 (1H, d, *J* = 6.5 Hz), 7.24 (1H, t, *J* = 7.9 Hz), 7.15 (1H, t, *J* = 6.2 Hz), 5.29 (2H, s), 3.66 (6H, br. s), 3.53 (2H, s), 3.46 (3H, s), 3.20 (3H, s); ^13^C-NMR (100 MHz, CDCl_3_), δ (ppm): 169.0, 166.3, 154.8, 151.4, 148.3, 144.1, 136.9, 129.2, 128.7, 128.4, 106.8, 53.8, 51.5, 48.3, 44.0, 41.2, 29.9, 27.9; HRMS (ESI)+ calculated for C_20_H_21_FN_6_O_5_, [M + H]^+^: m/z 445.1630, found 445.1626.

#### General procedure synthesis of intermediates 17

To a solution of intermediate **11** (5 mmol), amine derivatives (**16**, 5 mmol) and DIEA (10 mmol) in DMF (10 ml), and HATU was added at room temperature for 24 h. The mixture was added water (50 ml), extracted with ethyl acetate (30 ml) three times. The combined organic layers were washed with water, saturated aqueous sodium bicarbonate and brine, and then dried over anhydrous sodium sulphate. After removing the solvent under reduced pressure, the crude product was purified by flash chromatography on silica gel, eluting with dichloromethane/methanol (1–2%), yield 79–96%.

##### *N-cyclopropyl-3-nitrobenzamide (17a)* light yellow solid, yield 79%

^1^H-NMR (600 MHz, CDCl_3_), δ (ppm): 8.56 (1H, s), 8.33 (1H, d, *J* = 8.1 Hz), 8.16 (1H, d, *J* = 7.7 Hz), 7.63 (1H, d, *J* = 7.7 Hz), 6.72 (1H, br. s), 2.94 (1H, m), 0.92 − 0.88 (2H, m), 0.70 − 0.68 (2H, m); ^13^C-NMR (150 MHz, CDCl_3_), δ (ppm): 166.5, 148.1, 136.0, 133.3, 129.8, 126.0, 121.6, 23.4, 6.7.

##### *N-cyclopropyl-4-methyl-3-nitrobenzamide (17b)* light yellow solid, yield 90%

^1^H-NMR (600 MHz, CDCl_3_), δ (ppm): 8.32 (1H, s), 7.96 (1H, dd, *J* = 8.0, 1.5 Hz), 7.43 (1H, d, *J* = 8.0 Hz), 6.56 (1H, br. s), 2.94 (1H, m), 2.65 (3H, s), 0.93 − 0.88 (2H, m), 0.71 − 0.66 (2H, m); ^13^C-NMR (150 MHz, CDCl_3_), δ (ppm): 166.5, 148.9, 137.0, 133.4, 133.2, 131.4, 122.8, 23.3, 20.4, 6.7.

##### *N-cyclopropyl-4-methoxy-3-nitrobenzamide (17c)* light yellow solid, yield 76%

^1^H-NMR (600 MHz, CDCl_3_), δ (ppm): 8.22 (1H, d, *J* = 2.1 Hz), 8.05 (1H, dd, *J* = 8.7, 2.1 Hz), 7.14 (1H, d, *J* = 8.7 Hz), 6.57 (1H, br. s), 4.02 (3H, s), 2.91 (1H, m), 2.65 (3H, s), 0.91 − 0.85 (2H, m), 0.69 − 0.66 (2H, m); ^13^C-NMR (150 MHz, CDCl_3_), δ (ppm): 166.3, 155.1, 138.8, 133.4, 126.5, 124.2, 113.4, 56.8, 23.3, 23.1, 6.6.

##### *N-cyclopentyl-3-nitrobenzamide (17d)* light yellow solid, yield 89%

^1^H-NMR (600 MHz, CDCl_3_), δ (ppm): 8.56 (1H, br. s), 8.34 (1H, ddd, *J* = 8.1, 2.1, 0.9 Hz), 8.17 (1H, d, *J* = 8.1 Hz), 7.65 (1H, t, *J* = 8.0 Hz), 6.36 (1H, d, *J* = 5.3 Hz), 4.44 (1H, m), 2.18 − 2.10 (2H, m), 1.80 − 1.74 (2H, m), 1.71 − 1.66 (2H, m), 1.60 − 1.52 (2H, m); ^13^C-NMR (150 MHz, CDCl_3_), δ (ppm): 164.8, 148.1, 136.4, 133.3, 129.8, 125.8, 121.6, 52.1, 52.0, 33.1, 33.1, 23.8.

##### *N-cyclopentyl-4-methyl-3-nitrobenzamide (17e)* light yellow solid, yield 84%

^1^H-NMR (600 MHz, CDCl_3_), δ (ppm): 8.30 (1H, d, *J* = 1.5 Hz), 7.94 (1H, dd, *J* = 8.0, 1.5 Hz), 7.41 (1H, d, *J* = 8.0 Hz), 6.27 (1H, d, *J* = 4.6 Hz), 4.40 (1H, m), 2.64 (3H, s), 2.13 − 2.07 (2H, m), 1.77 − 1.70 (2H, m), 1.70 − 1.64 (2H, m), 1.56 − 1.49 (2H, m); ^13^C-NMR (150 MHz, CDCl_3_), δ (ppm): 164.7, 148.9, 136.7, 134.0, 133.1, 131.4, 122.8, 52.0, 33.1, 23.8, 20.4.

##### *N-cyclopentyl-4-methoxy-3-nitrobenzamide (17f)* light yellow solid, yield 76%

^1^H-NMR (600 MHz, CDCl_3_), δ (ppm): 8.13 (1H, d, *J* = 2.1 Hz), 7.96 (1H, dd, *J* = 8.8, 2.1 Hz), 7.05 (1H, d, *J* = 8.8 Hz), 6.17 (1H, d, *J* = 6.4 Hz), 4.31 (1H, m), 3.93 (3H, s), 2.04 − 1.98 (2H, m), 1.70 − 1.63 (2H, m), 1.62 − 1.55 (2H, m), 1.48 − 1.42 (2H, m); ^13^C-NMR (150 MHz, CDCl_3_), δ (ppm): 164.4, 154.9, 138.9, 133.4, 127.0, 124.1, 113.4, 56.8, 51.8, 33.1, 33.1, 29.6, 23.8.

##### *N-cyclohexyl-3-nitrobenzamide (17 g)* light yellow solid, yield 88%

^1^H-NMR (600 MHz, CDCl_3_), δ (ppm): 8.19 (1H, s), 7.84 (1H, d, *J* = 8.5 Hz), 7.81 (1H, d, *J* = 8.5 Hz), 6.12 (1H, d, *J* = 6.6 Hz), 3.96 (1H, m), 2.04 − 2.02 (2H, br. s), 1.79 − 1.77 (2H, br. s), 1.68 − 1.66 (1H, m), 1.45 − 1.38 (2H, m), 1.29 − 1.22 (2H, m); ^13^C-NMR (150 MHz, CDCl_3_), δ (ppm): 163.3, 149.6, 135.5, 135.4, 131.4, 123.9, 117.5, 49.4, 33.1, 25.4, 24.9.

##### *N-cyclohexyl-4-methyl-3-nitrobenzamide (17h)* light yellow solid, yield 86%

^1^H-NMR (400 MHz, CDCl_3_), δ (ppm): 8.30 (1H, s), 7.94 (1H, d, *J* = 7.9 Hz), 7.42 (1H, d, *J* = 7.9 Hz), 6.12 (1H, d, *J* = 6.7 Hz), 3.97 (1H, m), 2.04 − 2.02 (2H, br. s), 1.81 − 1.76 (2H, br. s), 1.69 − 1.66 (1H, m), 1.45 − 1.38 (2H, m), 1.29 − 1.22 (2H, m); ^13^C-NMR (150 MHz, CDCl_3_), δ (ppm): 164.1, 148.9, 136.7, 134.1, 133.2, 131.5, 122.8, 49.1, 33.1, 25.5, 24.9, 20.5.

##### *N-cyclohexyl-4-methoxy-3-nitrobenzamide (17i)* light yellow solid, yield 92%

^1^H-NMR (600 MHz, CDCl_3_), δ (ppm): 8.22 (1H, d, *J* = 2.1 Hz), 8.05 (1H, dd, *J* = 8.7, 2.1 Hz), 7.14 (1H, d, *J* = 8.7 Hz), 6.08 (1H, d, *J* = 7.3 Hz), 4.02 (3H, s), 3.96 (1H, m), 2.04 − 2.02 (2H, br. s), 1.81 − 1.76 (2H, br. s), 1.69 − 1.66 (1H, m), 1.45 − 1.38 (2H, m), 1.29 − 1.22 (2H, m); ^13^C-NMR (150 MHz, CDCl_3_), δ (ppm): 163.8, 163.8, 155.0, 138.9, 133.3, 127.2, 124.0, 113.4, 56.8, 49.1, 49.0, 33.1, 25.4, 24.9.

##### *N-cycloheptyl-3-nitrobenzamide (17j)* light yellow solid, yield 92%

^1^H-NMR (600 MHz, CDCl_3_), δ (ppm): 8.56 (1H, t, *J* = 2.1 Hz), 8.35 (1H, ddd, *J* = 8.2, 2.1, 0.9 Hz), 8.16 (1H, d, *J* = 8.2 Hz), 7.65 (1H, t, *J* = 8.2 Hz), 6.28 (1H, d, *J* = 6.7 Hz), 4.18 (1H, m), 2.10 − 2.04 (2H, m), 1.70 − 1.68 (4H, m), 1.57 − 1.56 (6H, m); ^13^C-NMR (150 MHz, CDCl_3_), δ (ppm): 163.9, 148.1, 136.7, 133.2, 129.7, 125.8, 121.5, 51.5, 35.1, 27.9, 24.1.

##### *N-cycloheptyl-4-methyl-3-nitrobenzamide (17k)* light yellow solid, yield 82%

^1^H-NMR (600 MHz, CDCl_3_), δ (ppm): 8.30 (1H, s), 7.93 (1H, d, *J* = 7.9 Hz), 7.41 (1H, d, *J* = 7.9 Hz), 6.23 (1H, d, *J* = 6.7 Hz), 4.15 (1H, m), 2.64 (3H, s), 2.06 − 2.02 (2H, m), 1.70 − 1.67 (4H, m), 1.57 − 1.55 (6H, m); ^13^C-NMR (150 MHz, CDCl_3_), δ (ppm): 163.8, 148.9, 136.7, 134.2, 133.2, 131.4, 122.8, 51.3, 35.1, 28.0, 24.1, 20.5.

##### *N-cycloheptyl-4-methoxy-3-nitrobenzamide (17 l)* light yellow solid, yield 89%

^1^H-NMR (600 MHz, CDCl_3_), δ (ppm): 8.21 (1H, d, *J* = 2.2 Hz), 8.05 (1H, dd, *J* = 8.7, 2.2 Hz), 7.14 (1H, d, *J* = 8.7 Hz), 6.17 (1H, d, *J* = 7.40 Hz), 4.14 (1H, m), 4.02 (3H, s), 2.06 − 2.02 (2H, m), 1.70 − 1.67 (4H, m), 1.57 − 1.55 (6H, m); ^13^C-NMR (150 MHz, CDCl_3_), δ (ppm): 163.5, 154.9, 138.9, 133.3, 127.2, 124.0, 113.4, 56.8, 51.3, 51.2, 35.1, 35.1, 28.0, 24.1.

#### Synthesis of compounds 18

To a solution of intermediate **17** (3 mmol), iron powder (12 mmol) and ammonium chloride (NH_4_Cl, 15 mmol) in 95% ethanol (20 ml), and a catalytic amount of glacial acetic acid was added under strenuous stirring, and then the mixture was allowed to warm up to reflux for 4 h. The additional iron powder was removed by filtration, and the filtrate was concentrated to dryness to give the crude product and used directly for the next reaction without purification, yield 80–96%.

##### 3-amino-N-cyclopropylbenzamide (18a) light brown solid, yield 86%

^1^H-NMR (600 MHz, CDCl_3_), δ(ppm): 7.16 (1H, t, *J* = 7.8 Hz), 7.11 (1H, s), 7.00 (1H, d, *J* = 7.8 Hz), 6.77 (1H, dd, *J* = 7.8, 1.5 Hz), 6.31 (1H, s), 3.80 (2H, br. s), 2.87 (1H, m), 0.84 (2H, m), 0.59 (2H, m); ^13^C-NMR (150 MHz, CDCl_3_), δ(ppm): 169.1, 146.8, 135.6, 129.3, 117.8, 116.2, 113.7, 23.1, 6.7.

##### 3-amino-N-cyclopropyl-4-methylbenzamide (18b) light brown solid, yield 89%

^1^H-NMR (600 MHz, CDCl_3_), δ(ppm): 7.12 (1H, d, *J* = 1.5 Hz), 7.04 (1H, d, *J* = 7.7 Hz), 6.95 (1H, dd, *J* = 7.7, 1.5 Hz), 6.27 (1H, br. s), 3.72 (2H, br. s), 2.87 (1H, m), 2.17 (3H, s), 0.84 (2H, m), 0.59 (2H, m); ^13^C-NMR (150 MHz, CDCl_3_), δ(ppm): 169.0, 144.8, 133.3, 130.3, 125.8, 116.2, 113.6, 23.0, 17.3, 6.7.

##### *3-amino-N-cyclopropyl-4-methoxybenzamide (18c)* light brown solid, yield 81%

^1^H-NMR (600 MHz, CDCl_3_), δ(ppm): 7.16 (1H, d, *J* = 2.1 Hz), 7.07 (1H, dd, *J* = 8.3, 2.1 Hz), 6.74 (1H, d, *J* = 8.3 Hz), 6.23 (1H, br. s), 3.87 (5H, br. s), 2.86 (1H, m), 0.84 (2H, m), 0.59 (2H, m); ^13^C-NMR (150 MHz, CDCl_3_), δ(ppm): 168.8, 149.7, 136.2, 127.2, 117.1, 113.5, 109.4, 55.5, 23.0, 6.7.

##### *3-amino-N-cyclopentylbenzamide (18d)* light brown solid, yield 84%

^1^H-NMR (600 MHz, CDCl_3_), δ(ppm): 7.18 (1H, t, *J* = 7.8 Hz), 7.12 (1H, s), 7.04 (1H, d, *J* = 7.6 Hz), 6.78 (1H, dd, *J* = 7.6, 1.5 Hz), 6.07 (1H, s), 4.38 (1H, m), 2.11 − 2.05 (2H, m), 1.74 − 1.71 (2H, m), 1.68 − 1.62 (2H, m), 1.51 − 1.45 (2H, m); ^13^C-NMR (150 MHz, CDCl_3_), δ(ppm): 167.4, 146.7, 136.1, 129.3, 117.7, 116.2, 113.7, 51.6, 33.2, 23.8.

##### *3-amino-N-cyclopentyl-4-methylbenzamide (18e)* light brown solid, yield 83%

^1^H-NMR (600 MHz, CDCl_3_), δ(ppm): 7.14 (1H, s), 7.07 (1H, d, *J* = 7.7 Hz), 6.99 (1H, d, *J* = 7.7 Hz), 6.05 (1H, br. s), 4.38 (1H, m), 2.19 (3H, s), 2.11 − 2.05 (2H, m), 1.74 − 1.71 (2H, m), 1.68 − 1.62 (2H, m), 1.51 − 1.45 (2H, m); ^13^C-NMR (150 MHz, CDCl_3_), δ(ppm): 167.3, 144.8, 133.8, 130.3, 125.6, 116.2, 113.6, 51.5, 33.2, 23.8, 17.3.

##### *3-amino-N-cyclopentyl-4-methoxybenzamide (18f)* light brown solid, yield 79%

^1^H-NMR (600 MHz, CDCl_3_), δ(ppm): 7.15 (1H, d, *J* = 1.2 Hz), 7.09 (1H, dd, *J* = 8.2, 1.2 Hz), 6.75 (1H, d, *J* = 8.2 Hz), 5.98 (1H, d, *J* = 5.4 Hz), 4.39 − 4.34 (1H, m), 3.88 (2H, s), 3.87 (3H, s), 2.09 − 2.04 (2H, m), 1.74 − 1.68 (2H, m), 1.67 − 1.62 (2H, m), 1.49 − 1.43 (2H, m); ^13^C-NMR (150 MHz, CDCl_3_), δ(ppm): 167.1, 149.5, 136.1, 127.8, 117.1, 113.5, 109.5, 55.6, 51.5, 33.2, 33.2, 23.8, 23.8.

##### *3-amino-N-cyclohexylbenzamide (18 g)* light brown solid, yield 86%

^1^H-NMR (600 MHz, CDCl_3_), δ(ppm): 7.18 (1H, t, *J* = 7.7 Hz), 7.12 (1H, s), 7.04 (1H, d, *J* = 7.5 Hz), 6.78 (1H, d, *J* = 7.1 Hz), 5.99 (1H, br. s), 3.96 (1H, m), 2.02 (2H, m), 1.75 (2H, m), 1.65 (1H, m), 1.43 (2H, m), 1.22 (3H, m); ^13^C-NMR (150 MHz, CDCl_3_), δ(ppm): 166.9, 146.7, 136.3, 129.3, 117.6, 116.2, 113.7, 48.5, 33.2, 25.5, 24.9.

##### *3-amino-N-cyclohexyl-4-methylbenzamide (18h)* light brown solid, yield 80%

^1^H-NMR (600 MHz, CDCl_3_), δ(ppm): 7.14 (1H, s), 7.07 (1H, d, *J* = 7.7 Hz), 7.00 (1H, dd, *J* = 7.7, 1.5 Hz), 5.97 (1H, br. s), 3.96 (1H, m), 2.19 (3H, s), 2.02 (2H, m), 1.75 (2H, m), 1.65 (1H, m), 1.43 (2H, m), 1.22 (3H, m); ^13^C-NMR (150 MHz, CDCl_3_), δ(ppm): 166.8, 144.8, 133.9, 130.3, 125.6, 116.2, 113.6, 48.4, 33.2, 25.6, 24.9, 17.3.

##### *3-amino-N-cyclohexyl-4-methoxybenzamide (18i)* light brown solid, yield 82%

^1^H-NMR (600 MHz, CDCl_3_), δ(ppm): 7.17 (1H, d, *J* = 1.9 Hz), 7.11 (1H, dd, *J* = 8.3, 1.9 Hz), 6.77 (1H, d, *J* = 8.3 Hz), 5.92 (1H, br. s), 3.95 (1H, m), 3.89 (3H, s), 2.02 (2H, m), 1.75 (2H, m), 1.65 (1H, m), 1.43 (2H, m), 1.22 (3H, m); ^13^C-NMR (150 MHz, CDCl_3_), δ(ppm): 166.6, 149.5, 136.1, 127.9, 117.1, 113.5, 109.4, 55.5, 48.4, 33.3, 25.6, 24.9.

##### *3-amino-N-cycloheptylbenzamide (18j)* light brown solid, yield 87%

^1^H-NMR (600 MHz, CDCl_3_), δ(ppm): 7.19 (1H, t, *J* = 7.8 Hz), 7.12 (2H, s), 7.04 (1H, d, *J* = 7.5 Hz), 6.79 (1H, d, *J* = 7.3 Hz), 6.05 (1H, d, *J* = 5.3 Hz), 4.15 (1H, m), 2.02 (2H, m), 1.66 (4H, m), 1.55 (6H, m); ^13^C-NMR (150 MHz, CDCl_3_), δ(ppm): 166.6, 146.7, 136.3, 129.3, 117.6, 116.2, 113.7, 50.7, 35.1, 28.0, 24.1.

##### *3-amino-N-cycloheptyl-4-methylbenzamide (18k)* light brown solid, yield 88%

^1^H-NMR (600 MHz, CDCl_3_), δ(ppm): 7.11 (1H, d, *J* = 1.0 Hz), 7.05 (1H, d, *J* = 7.7 Hz), 6.97 (1H, dd, *J* = 7.7, 1.0 Hz), 6.02 (1H, d, *J* = 6.6 Hz), 4.13 (1H, m), 3.72 (2H, br. s), 2.17 (3H, s), 2.01 − 1.97 (2H, m), 1.64 − 1.49 (10H, m); ^13^C-NMR (150 MHz, CDCl_3_), δ(ppm): 166.5, 144.8, 134.1, 130.3, 125.5, 116.2, 113.6, 50.7, 35.2, 28.1, 24.1, 17.3.

##### *3-amino-N-cycloheptyl-4-methoxybenzamide (18 l)* White solid, yield 91%

^1^H-NMR (600 MHz, CDCl_3_), δ(ppm): 7.15 (1H, d, *J* = 1.0 Hz), 7.08 (1H, d, *J* = 8.0 Hz), 6.75 (1H, dd, *J* = 8.0, 1.0 Hz), 5.96 (2H, d, *J* = 5.7 Hz), 4.12 (1H, s), 3.88 (5H, br. s), 2.01 − 2.00 (2H, m), 1.64 (4H, br. s), 1.53 (6H, m); ^13^C-NMR (150 MHz, CDCl_3_), δ(ppm): 166.3, 149.5, 136.1, 128.0, 117.0, 113.5, 109.5, 55.5, 50.6, 35.2, 28.1, 24.1.

#### Synthesis of compounds 19a–l

To a solution of intermediate **10** (1 mmol), intermediate **18** (1 mmol) and DIEA (2 mmol) in DMF (10 ml), and HATU (1 mmol) was added at room temperature for 24 h. The mixture was added water (50 ml), extracted with ethyl acetate (30 ml) three times. The combined organic layers were washed with water, saturated aqueous sodium bicarbonate and brine, and then dried over anhydrous sodium sulphate. After removing the solvent under reduced pressure, the crude product was purified by flash chromatography on silica gel, eluting with dichloromethane/methanol (2–4%), yield 75–90%.

##### N-cyclopropyl-3–(2-(1,3-dimethyl-2,6-dioxo-1,2,3,6-tetrahydro-9H-purin-9-yl)acetamido)benzamide (19a)

White solid, m.p. 222–224 °C, yield 76%. ^1^H-NMR (600 MHz, DMSO-*d6*), δ (ppm): 10.56 (1H, s), 8.44 (1H, d, *J* = 4.1 Hz), 8.08 (1H, s), 7.98 (1H, s), 7.73 (1H, d, *J* = 8.0 Hz), 7.49 (1H, d, *J* = 7.7 Hz), 7.38 (1H, t, *J* = 7.9 Hz), 5.21 (2H, s), 3.46 (3H, s), 3.19 (3H, s), 2.83 (1H, m), 0.68 (2H, br. s), 0.56 (2H, br. s); ^13^C-NMR (150 MHz, CDCl_3_), δ (ppm): 167.8, 165.5, 154.9, 151.4, 148.4, 144.2, 139.0, 135.7, 129.2, 122.4, 122.0, 118.7, 106.9, 49.1, 29.9, 27.9, 23.5, 6.1; HRMS (ESI)^-^ calculated for C_19_H_20_N_6_O_4_, [M-H]^-^: m/z 395.1473, found 395.1463.

##### *N-cyclopropyl-3–(2-(1,3-dimethyl-2,6-dioxo-1,2,3,6-tetrahydro-9H-purin-9-yl)acetamido)-4-*methylbenzamide *(19b)*

White solid, m.p. 218–219 °C, yield 80%. ^1^H-NMR (600 MHz, DMSO-*d6*), δ (ppm): 9.85 (1H, s), 8.39 (1H, d, *J* = 4.0 Hz), 8.09 (1H, s), 7.84 (1H, s), 7.56 (1H, d, *J* = 8.0 Hz), 7.29 (1H, d, *J* = 8.0 Hz), 5.25 (2H, s), 3.45 (3H, s), 3.22 (3H, s), 2.82 (1H, m), 2.27 (3H, s), 0.67 (2H, br. s), 0.54 (2H, br. s); ^13^C-NMR (150 MHz, DMSO-*d6*), δ (ppm): 167.4, 165.7, 154.9, 151.5, 148.4, 144.1, 136.0, 135.8, 132.9, 130.6, 124.7, 124.5, 106.9, 48.9, 29.9, 27.9, 23.4, 18.2, 6.1; HRMS (ESI)+ calculated for C_20_H_22_N_6_O_4_, [M + H]^+^: m/z 411.1775, found 411.1776.

##### *N-cyclopropyl-3–(2-(1,3-dimethyl-2,6-dioxo-1,2,3,6-tetrahydro-9H-purin-9-yl)acetamido)-4-*methoxybenzamide *(19c)*

White solid, m.p. 231–233 °C, yield 86%. ^1^H-NMR (600 MHz, DMSO-*d6*), δ (ppm): 9.81 (1H, s), 8.39 (1H, s), 8.28 (1H, d, *J* = 3.8 Hz), 8.08 (1H, s), 7.58 (1H, dd, *J* = 8.5, 1.7 Hz), 7.10 (1H, d, *J* = 8.6 Hz), 5.29 (2H, s), 3.92 (3H, s), 3.45 (3H, s), 3.21 (3H, s), 2.79 (1H, m), 0.65 (2H, br. s), 0.53 (2H, br. s); ^13^C-NMR (150 MHz, DMSO-*d6*), δ (ppm): 167.5, 165.7, 154.9, 152.1, 151.5, 148.3, 144.2, 127.0, 126.8, 124.3, 121.7, 110.8, 106.9, 56.4, 49.3, 29.9, 27.9, 23.4, 6.1; HRMS (ESI)^-^ calculated for C_20_H_22_N_6_O_5_, [M-H]^−^: m/z 425.1579, found 425.1570.

##### *N-cyclopentyl-3–(2-(1,3-dimethyl-2,6-dioxo-1,2,3,6-tetrahydro-9H-purin-9-yl)acetamido)*benzamide *(19d)*

White solid, m.p. 207–208 °C, yield 87%. ^1^H-NMR (600 MHz, DMSO-*d6*), δ (ppm): 10.06 (1H, s), 8.40 (1H, d, *J* = 7.2 Hz), 8.11 (1H, s), 8.03 (1H, d, *J* = 1.7 Hz), 7.77 (1H, d, *J* = 8.3 Hz), 7.61 (1H, d, *J* = 7.2 Hz), 5.30 (2H, s), 4.19 (1H, m), 3.45 (3H, s), 3.22 (3H, s), 1.86 (2H, br. s), 1.67 (2H, br. s), 1.51 (4H, m); ^13^C-NMR (150 MHz, DMSO-*d6*), δ (ppm): 166.2, 165.5, 162.7, 154.9, 151.4, 148.9, 148.4, 144.2, 138.9, 136.2, 129.1, 128.9, 122.6, 121.8, 118.8, 116.5, 114.9, 113.3, 106.9, 51.3, 51.2, 49.1, 36.2, 32.5, 31.2, 29.9, 27.9, 24.0; HRMS (ESI)+ calculated for C_21_H_24_N_6_O_4_, [M + H]^+^: m/z 425.1932, found 425.1935.

##### *N-cyclopentyl-3–(2-(1,3-dimethyl-2,6-dioxo-1,2,3,6-tetrahydro-9H-purin-9-yl)acetamido)-4-*methylbenzamide *(19e)*

White solid, m.p. 211–213 °C, yield 89%. ^1^H-NMR (600 MHz, DMSO-*d6*), δ (ppm): 9.84 (1H, s), 8.23 (1H, d, *J* = 7.3 Hz), 8.09 (1H, s), 7.84 (1H, d, *J* = 1.5 Hz), 7.60 (1H, dd, *J* = 7.9, 1.5 Hz), 7.29 (1H, d, *J* = 7.9 Hz), 5.26 (2H, s), 4.19 (1H, m), 3.45 (3H, s), 3.22 (3H, s), 2.27 (3H, s), 1.86 (2H, br. s), 1.67 (2H, br. s), 1.50 (2H, br. s); ^13^C-NMR (150 MHz, DMSO-*d6*), δ (ppm): 165.8, 165.7, 154.9, 151.5, 148.4, 144.1, 135.9, 135.6, 133.3, 130.5, 124.8, 124.7, 106.9, 51.3, 48.9, 32.5, 29.9, 27.9, 24.0, 18.2; HRMS (ESI)- calculated for C_22_H_26_N_6_O_4_, [M-H]^-^: m/z 437.1943, found 437.1932.

##### *N-cyclopentyl-3–(2-(1,3-dimethyl-2,6-dioxo-1,2,3,6-tetrahydro-9H-purin-9-yl)acetamido)-4-*methoxybenzamide *(19f)*

White solid, m.p. 219–220 °C, yield 81%. ^1^H-NMR (600 MHz, DMSO-*d6*), δ (ppm): 9.80 (1H, s), 8.38 (1H, s), 8.12 (1H, d, *J* = 7.3 Hz), 8.08 (1H, s), 7.62 (1H, dd, *J* = 8.0, 2.0 Hz), 7.11 (1H, d, *J* = 8.6 Hz), 5.29 (2H, s), 4.17 (1H, m), 3.93 (3H, s), 3.46 (3H, s), 3.21 (3H, s), 1.84 (2H, br. s), 1.66 (2H, br. s), 1.49 (4H, m); ^13^C-NMR (150 MHz, DMSO-*d6*), δ (ppm): 165.9, 165.7, 154.9, 152.0, 151.5, 148.4, 144.2, 127.5, 126.7, 124.4, 121.9, 110.7, 106.9, 56.4, 51.3, 49.3, 32.5, 29.9, 27.9, 24.0; HRMS (ESI)+ calculated for C_22_H_26_N_6_O_5_, [M + H]^+^: m/z 455.2037, found 455.2041.

##### N-cyclohexyl-3–(2-(1,3-dimethyl-2,6-dioxo-1,2,3,6-tetrahydro-9H-purin-9-yl)acetamido)benzamide (19g)

White solid, m.p. 195–197 °C, yield 88%. ^1^H-NMR (600 MHz, DMSO-*d6*), δ (ppm): 10.56 (1H, s), 8.26 (1H, d, *J* = 7.9 Hz), 8.08 (1H, s), 7.95 (1H, s), 7.74 (1H, d, *J* = 8.0 Hz), 7.52 (1H, d, *J* = 7.7 Hz), 7.38 (1H, t, *J* = 7.9 Hz), 5.21 (2H, s), 3.93 (1H, m), 3.46 (3H, s), 3.20 (3H, s), 1.82 (2H, br. s), 1.64 (2H, br. s), 1.57 (4H, m), 1.42 (2H, m); ^13^C-NMR (150 MHz, DMSO-*d6*), δ (ppm): 165.5, 165.4, 154.9, 151.5, 148.4, 144.2, 138.9, 136.4, 129.1, 122.6, 121.8, 118.8, 106.9, 50.9, 49.1, 40.4, 34.7, 29.9, 28.2, 27.9, 24.3; HRMS (ESI)+ calculated for C_22_H_26_N_6_O_4_, [M + H]^+^: m/z 439.2088, found 439.2108.

##### *N-cyclohexyl-3–(2-(1,3-dimethyl-2,6-dioxo-1,2,3,6-tetrahydro-9H-purin-9-yl)acetamido)-4-*methylbenzamide *(19h)*

White solid, m.p. 199–201 °C, yield 85%. ^1^H-NMR (600 MHz, DMSO-*d6*), δ (ppm): 9.84 (1H, s), 8.15 (1H, d, *J* = 8.0 Hz), 8.09 (1H, s), 7.85 (1H, s), 7.60 (1H, d, *J* = 8.0 Hz), 7.29 (1H, d, *J* = 7.9 Hz), 5.26 (2H, s), 3.73 (1H, m), 3.45 (3H, s), 3.20 (3H, s), 2.28 (3H, s), 1.78 (2H, br. s), 1.72 (2H, br. s), 1.59 (1H, d, *J* = 12.4 Hz), 1.29 (4H, m), 1.10 (1H, m); ^13^C-NMR (150 MHz, DMSO-*d6*), δ (ppm): 165.7, 165.2, 154.9, 151.5, 148.4, 144.1, 135.9, 135.6, 133.4, 130.5, 124.9, 124.7, 106.9, 48.9, 48.7, 32.8, 29.9, 27.9, 25.7, 25.4, 18.2; HRMS (ESI)+ calculated for C_23_H_28_N_6_O_4_, [M + H]^+^: m/z 453.2245, found 453.2251.

##### *N-cyclohexyl-3–(2-(1,3-dimethyl-2,6-dioxo-1,2,3,6-tetrahydro-9H-purin-9-yl)acetamido)-4-*methoxybenzamide *(19i)*

White solid, m.p. 185–187 °C, yield 86%. ^1^H-NMR (600 MHz, DMSO-*d6*), δ (ppm): 9.80 (1H, s), 8.39 (1H, s), 8.08 (1H, s), 8.02 (1H, d, *J* = 7.9 Hz), 7.62 (1H, dd, *J* = 8.6, 1.7 Hz), 7.11 (1H, d, *J* = 8.6 Hz), 5.29 (2H, s), 3.92 (3H, s), 3.71 (1H, m), 3.45 (3H, s), 3.21 (3H, s), 1.77 (2H, br. s), 1.71 (2H, br. s), 1.59 (1H, d, *J* = 12.4 Hz), 1.31 − 1.23 (4H, m), 1.10 − 1.08 (1H, m); ^13^C-NMR (150 MHz, DMSO-*d6*), δ (ppm): 165.7, 165.3, 154.9, 152.0, 151.5, 148.4, 144.2, 127.5, 126.7, 124.4, 121.9, 110.7, 106.9, 56.4, 49.3, 48.7, 32.9, 29.9, 27.9, 25.7, 25.4; HRMS (ESI)+ calculated for C_23_H_28_N_6_O_5_, [M + H]^+^: m/z 469.2194, found 469.2201.

##### *N-cycloheptyl-3–(2-(1,3-dimethyl-2,6-dioxo-1,2,3,6-tetrahydro-9H-purin-9-yl)acetamido)*benzamide *(19j)*

White solid, m.p. 189–190 °C, yield 85%. ^1^H-NMR (600 MHz, DMSO-*d6*), δ (ppm): 10.55 (1H, s), 8.25 (1H, d, *J* = 7.9 Hz), 8.08 (1H, s), 7.95 (1H, s), 7.74 (1H, d, *J* = 8.1 Hz), 7.52 (1H, d, *J* = 7.7 Hz), 7.38 (1H, t, *J* = 7.9 Hz), 5.21 (2H, s), 3.94 (1H, m), 3.46 (3H, s), 3.22 (3H, s), 1.82 (2H, br. s), 1.64 (2H, br. s), 1.56 (4H, m), 1.49 (2H, m), 1.40 (2H, m); ^13^C-NMR (150 MHz, DMSO-*d6*), δ (ppm): 165.5, 165.4, 154.9, 151.4, 148.4, 144.2, 138.9, 136.4, 129.1, 122.6, 121.8, 118.8, 106.9, 50.9, 49.1, 34.7, 29.9, 28.2, 27.9, 24.3; HRMS (ESI)+ calculated for C_23_H_28_N_6_O_4_, [M + H]^+^: m/z 453.2245, found 453.2246.

##### *N-cycloheptyl-3–(2-(1,3-dimethyl-2,6-dioxo-1,2,3,6-tetrahydro-9H-purin-9-yl)acetamido)-4-*methylbenzamide *(19k)*

White solid, m.p. 194–196 °C, yield 87%. ^1^H-NMR (600 MHz, DMSO-*d6*), δ (ppm): 9.84 (1H, s), 8.19 (1H, d, *J* = 7.9 Hz), 8.09 (1H, s), 7.83 (1H, s), 7.59 (1H, dd, *J* = 7.9, 1.3 Hz), 7.29 (1H, d, *J* = 7.9 Hz), 5.26 (2H, s), 3.93 (1H, m), 3.45 (3H, s), 3.22 (3H, s), 2.27 (3H, s), 1.82 (2H, br. s), 1.64 (2H, br. s), 1.56 (4H, m), 1.49 (2H, m), 1.40 (2H, m); ^13^C-NMR (150 MHz, DMSO-*d6*), δ (ppm): 165.7, 165.1, 154.9, 151.5, 148.4, 144.1, 135.9, 135.7, 133.4, 130.5, 124.8, 124.7, 106.9, 50.8, 48.9, 40.4, 34.7, 29.9, 28.2, 27.9, 24.3, 18.2; HRMS (ESI)+ calculated for C_24_H_30_N_6_O_4_, [M + H]^+^: m/z 467.2401, found 467.2409.

##### *N-cycloheptyl-3–(2-(1,3-dimethyl-2,6-dioxo-1,2,3,6-tetrahydro-9H-purin-9-yl)acetamido)-4-*methoxybenzamide *(19l)*

White solid, m.p. 197–198 °C, yield 94%. ^1^H-NMR (600 MHz, DMSO-*d6*), δ (ppm): 9.79 (1H, s), 8.38 (1H, d, *J* = 1.5 Hz), 8.08 (1H, s), 8.07 (1H, d, *J* = 8.0 Hz), 7.62 (1H, dd, *J* = 8.0, 1.8 Hz), 7.10 (1H, d, *J* = 8.6 Hz), 5.29 (2H, s), 3.92 (3H, s), 3.91 (1H, m), 3.45 (3H, s), 3.21 (3H, s), 1.79 (2H, br. s), 1.63 (2H, br. s), 1.54 (4H, m), 1.48 (2H, m), 1.39 (2H, m); ^13^C-NMR (150 MHz, DMSO-*d6*), δ (ppm): 165.7, 165.1, 154.9, 152.0, 151.5, 148.3, 144.2, 127.6, 126.7, 124.4, 121.8, 110.7, 106.9, 56.4, 50.8, 49.3, 34.8, 29.9, 28.2, 27.9, 24.4; HRMS (ESI)+ calculated for C_24_H_30_N_6_O_4_, [M + H]^+^: m/z 483.2350, found 483.2344.

### Molecular docking

The discovery Studio 3.5 was used to perform molecular docking, and the structure of ATAD2 (PDB code 6YB4)^10^ was downloaded from https://www.rcsb.org. First, the potential active site was defined according to the reference ligand by a radius of 8.5 Å. The structure of ATAD2 bromodomain is prepared by removing water molecules, adding hydrogen atoms, and charging the Charmm forcefield. The ligands were prepared by adding hydrogen atoms and energy minimisation.

### Molecular dynamics simulations and binding free energy calculation

The MD simulations were conducted by Amber 10 package^26^. The steepest descent method was adopted, and energy minimisation was restrained with 0.1 kcal/mol•Å2 for 5000 steps. Next, the restraints of the ligand were removed, and the last energy minimisation was executed without any restraints. For the equilibration and production, the integration time step was set to 2 fs. The temperature was set from 0 to 310 K for 50 ps for the annealed program. Last, the equilibration of the complex system was processed for 500 ps without any restraints. The production was carried out for 200 ns under removing all restrictions. Additionally, the binding free energy of the complexs was calculated by MM-GBSA using AMBER10^27^.

### The enzymatic assay

The assay was performed by TR-FRET technology using recombinant bromodomain and BET Ligand^8^. The compounds were diluted with indicated folds in 5% DMSO in reaction buffer and 2 µl of the dilution was added to a 20 µl reaction so that the final concentration of DMSO is 0.5% in all of the reactions. The 20 µl reaction mixture consists of the ATAD2 recombinant bromodomain protein, the indicated amount of the inhibitor, ligand, and the reactive dyes. The reaction mixture was incubated for 3 h before reading the TR-FRET signal. Fluorescence signals for both the donor and acceptor dyes were measured using a Tecan Infinite M1000 plate reader. Binding experiments were performed in duplicate at each concentration and the data were analysed using GraphPad Prism.

### Cell culture, antibodies, and reagents

BT-549 cells, MDA-MB-231 cells, and MCF-10A cells were obtained from American Type Culture Collection (ATCC, Manassas, VA, USA) and were cultured in RPIM-1640 supplemented with 10% foetal bovine serum under a 5% CO_2_ atmosphere. The following antibodies were used in this study: ATAD2 (1:1000; 50,563, CST), c-Myc (1:1000; 18,583, CST), p-c-Myc^Ser62^ (1:1000; 13,748, CST), caspase-9 (1:1000; 9508, CST), Bax(1:2000; 5023,CST), Bcl-2(1:2000; 15,071,CST), caspase8(1:1000; 9746, CST), MMP-2(1:1000; 40,994,CST), E-cadherin (1:1000; 14,472,CST), PARP (1:2000; 9532, CST), caspase-3 (1:2000; 9665, CST), and β-actin (1:2000; 3700, CST). MTT (M2128, St. Louis, MO, USA). BAY-850 were purchased from *MCE* (MedChemExpress LLC, HY-119254, 98%).

### Cell viability assay

BT-549 cells were seeded at a density of 5 × 10^4^ cells/ml in a 96-well plate^15^. Following a 24-h incubation period, the cells were exposed to various concentrations of different compounds for the specified durations. Cell viability was evaluated using an MTT assay.

### 3D Cell culture

For 3D cell culture, 10,000 cells were placed in 96-well spherical plates for 24h and treated with different concentrations of compound **19f, BAY-859 or AM879**. The diameter of the cell balls was measured by photographing.

### Apoptosis assays

To assess apoptosis, BT-549 cells were exposed to varying concentrations of compound **19f**, and apoptosis ratios were quantified via Annexin-V/PI double staining.

### Immunofluorescence analysis

To prevent non-specific antibody binding, BT-549 cells were treated with a blocking buffer (PBS +1.5% goat serum) prior to immunofluorescence staining. The cells were subsequently incubated with a p-c-Myc^Ser62^ antibody (1:200) diluted in blocking buffer and allowed to incubate overnight at 4 °C. Afterward, the cells were further incubated with fluorescent-labelled secondary antibodies for 1 h at room temperature.

### Western blot

Cells were exposed to AM879, BAY-850 or compound **19f** for the specified time intervals^15^. Both adherent and floating cells were harvested and subsequently resuspended in a lysis buffer, followed by incubation at 4 °C for 1 h. Following centrifugation at 12,000 rpm for 20 min, the protein content of the supernatant was quantified using the BCA protein assay. Then protein were separated by 8–15% SDS-PAGE and transferred onto PVDF membranes. The membranes were blocked with blocking buffer and subjected to primary antibody incubation, followed by incubation with HRP-conjugated secondary antibody. Protein detection was achieved using ECL as the HRP substrate.

### Transwell assay

BT-549 cells were seeded into the upper chamber of a Transwell insert at a density of 15,000 cells per well. The cells were treated with either DMSO or compound **19f** for 48 h, after which non-migrated cells were removed from the upper surface, while the migrated cells were stained with crystal violet. Then, the cells were washed with PBS and imaged using a phase-contrast microscope.

### Statistical analysis

The data are presented as means ± standard error of the mean (SEM) and were confirmed by at least three independent experiments. Statistical analysis was performed using one-way analysis of variance (ANOVA) followed by Student–Newman–Keuls test for *post hoc* comparisons between multiple groups. A value of *p* < 0.05 was considered to be statistically significant.

## Supplementary Material

Supplemental Material
