# Supplementary material for: Fragment-based design, synthesis and biological evaluation of theophylline derivatives as ATAD2 inhibitors in BT-549 cells
Source: J Enzyme Inhib Med Chem. 2023 Aug 3;38(1):2242601. doi: 10.1080/14756366.2023.2242601 (PMC10402865; doi:10.1080/14756366.2023.2242601)
Supplement: Supplemental Material [file IENZ_A_2242601_SM5875.pdf]

Supporting Information

1. Figure S1

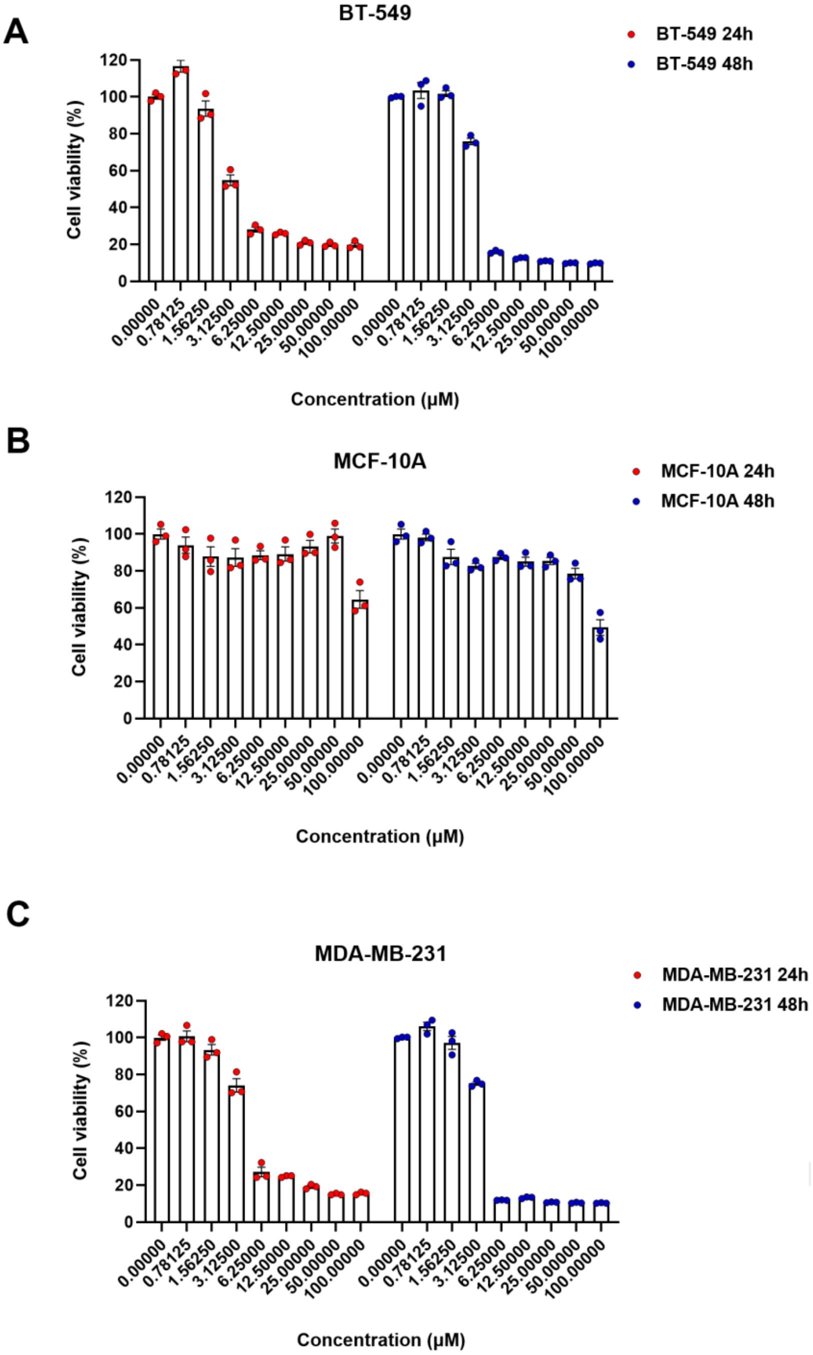

Figure S1 The effect of compound **19f** on the cell viability of BT-549 cells, MDA-MB-231 cells and MCF-10A cells.

2. original images of western blot

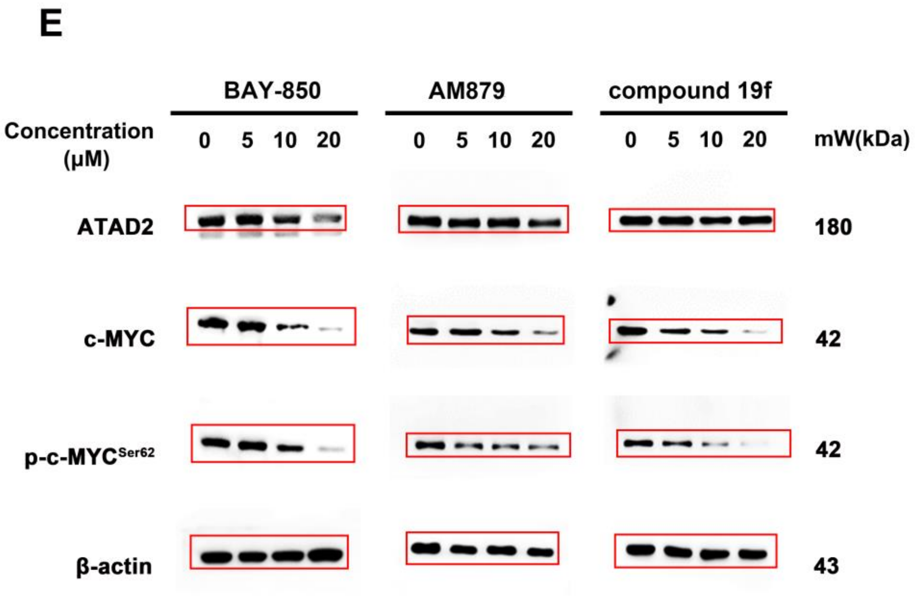
E
BAY-850
AM879
compound 19f
Concentration (μM)
0 5 10 20
mW(kDa)
ATAD2
180
c-MYC
42
p-c-MYC^Ser62
42
β-actin
43

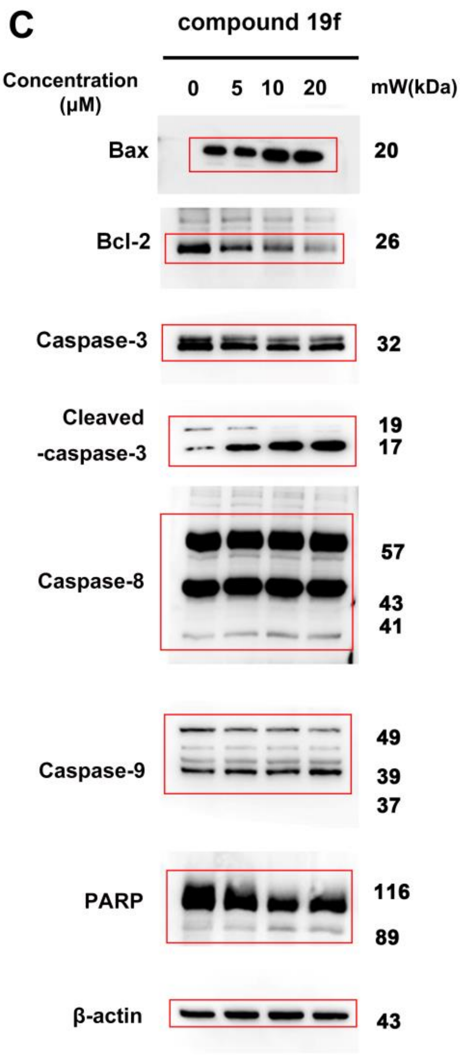
C
compound 19f
Concentration (μM)
0 5 10 20
mW(kDa)
Bax
20
Bcl-2
26
Caspase-3
32
Cleaved -caspase-3
19
17
Caspase-8
57
43
41
Caspase-9
49
39
37
PARP
116
89
β-actin
43

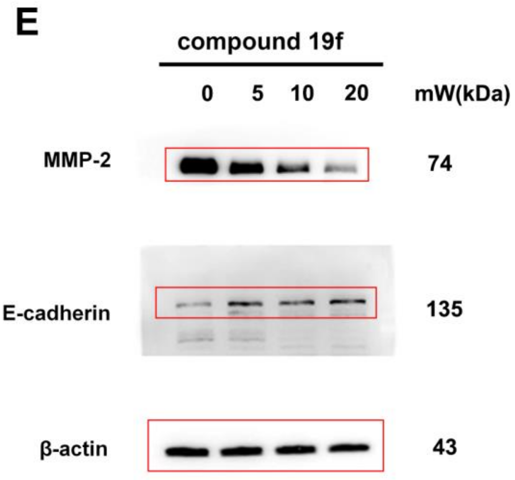

3. ^{1}H and ^{13}C NMR, HR-MS Spectrum of Compounds

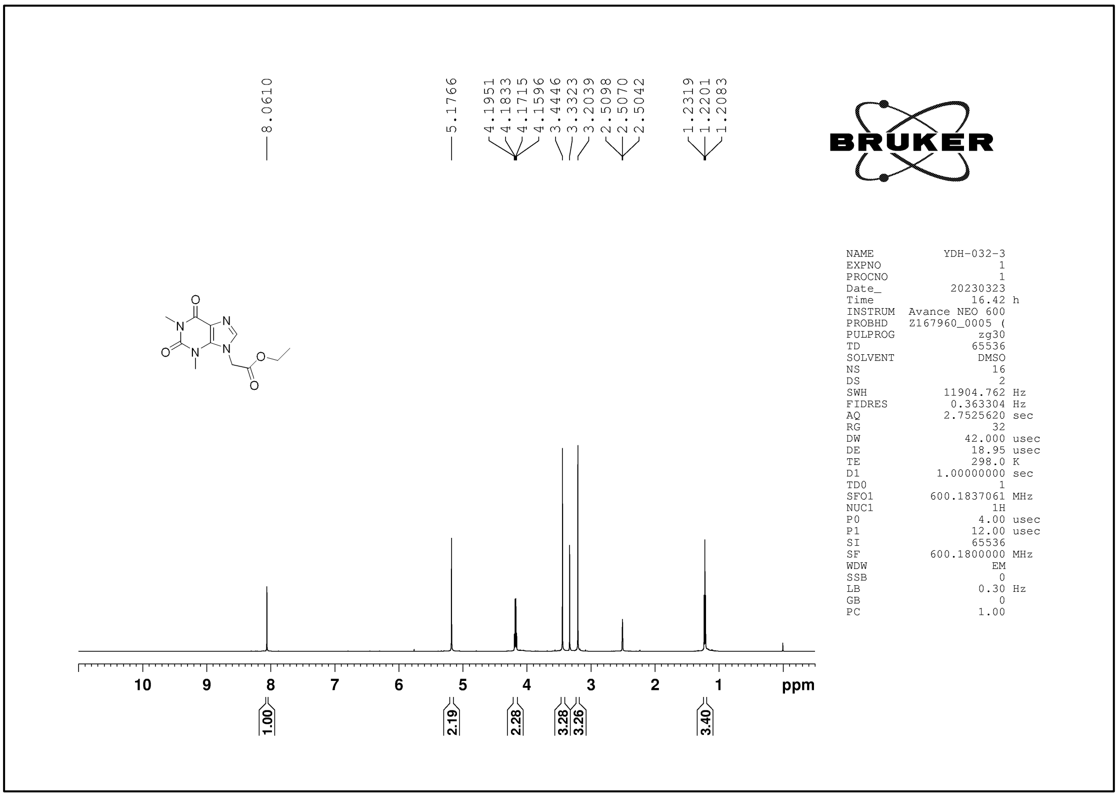

^{1}H-NMR spectrum of compound 9

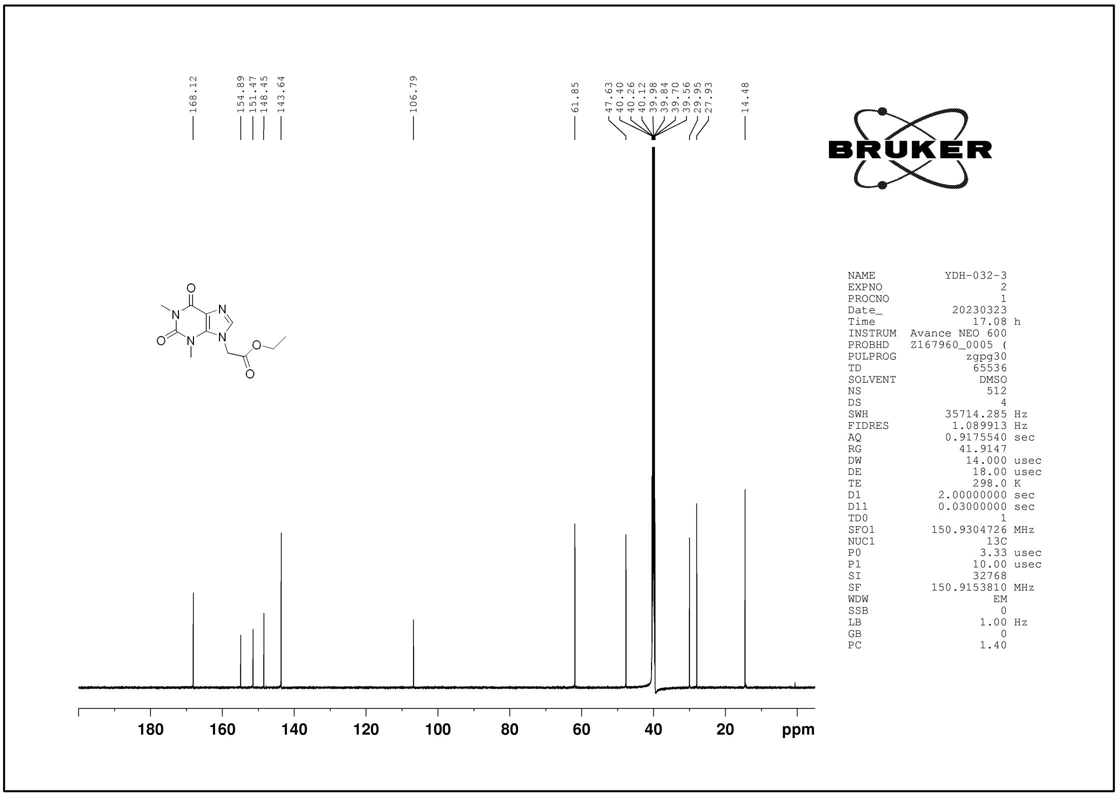

^{13}C-NMR spectrum of compound 9

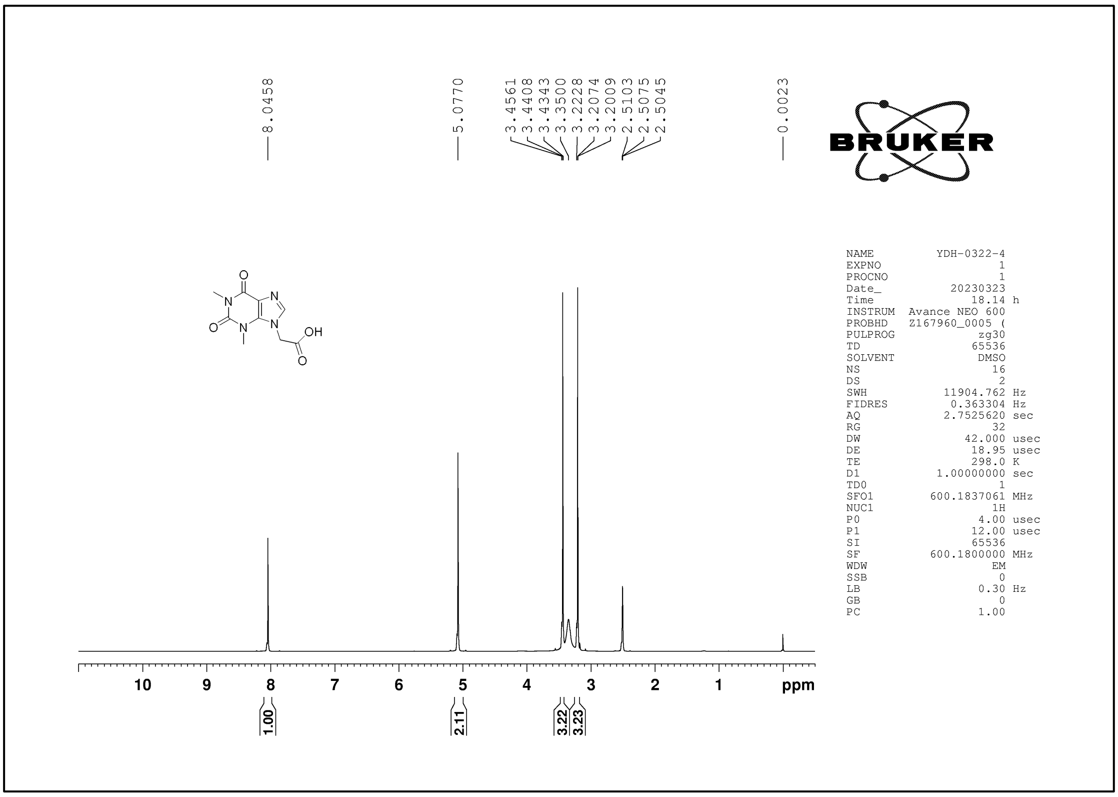

^{1}H-NMR spectrum of compound 10

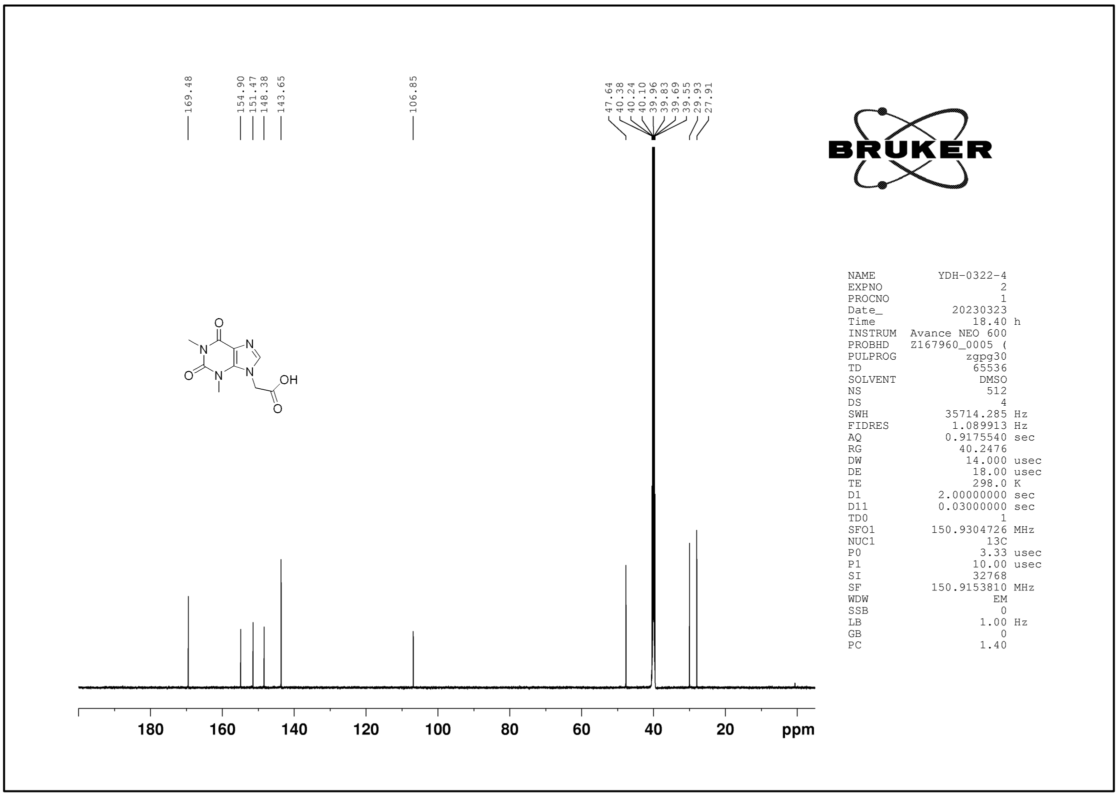

^{13}C-NMR spectrum of compound 10

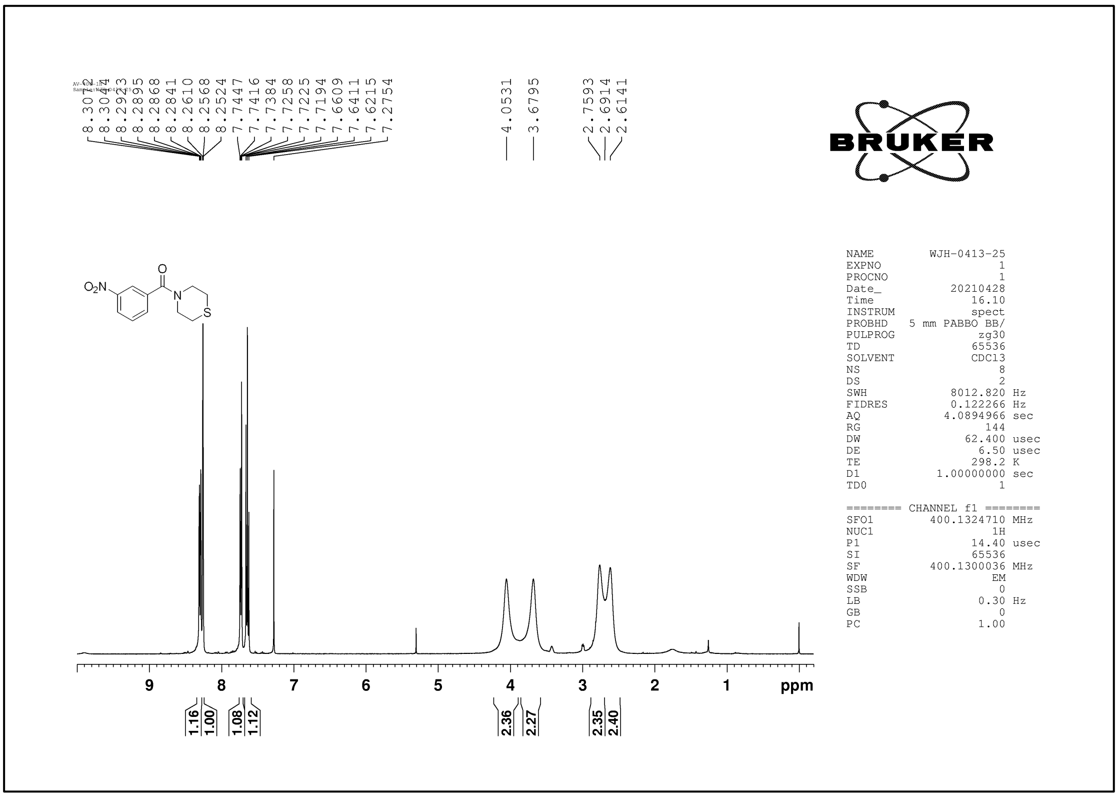

^{1}H-NMR spectrum of compound 13a

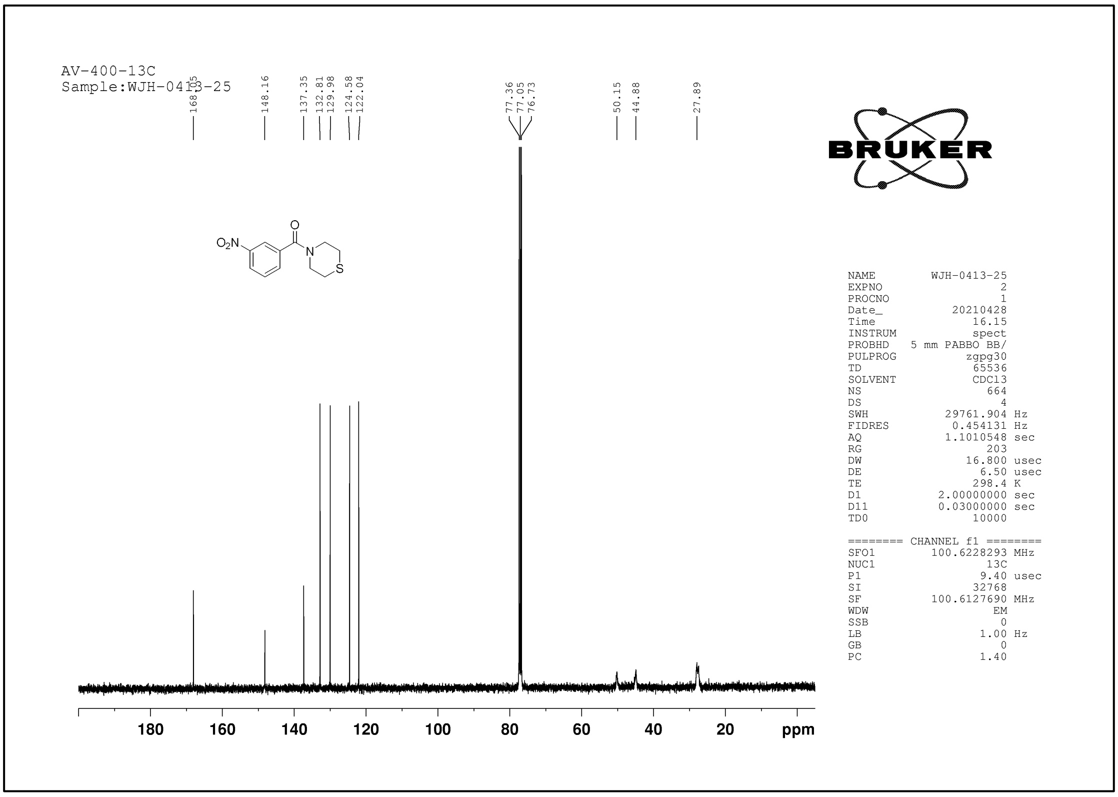

^{13}C-NMR spectrum of compound 13a

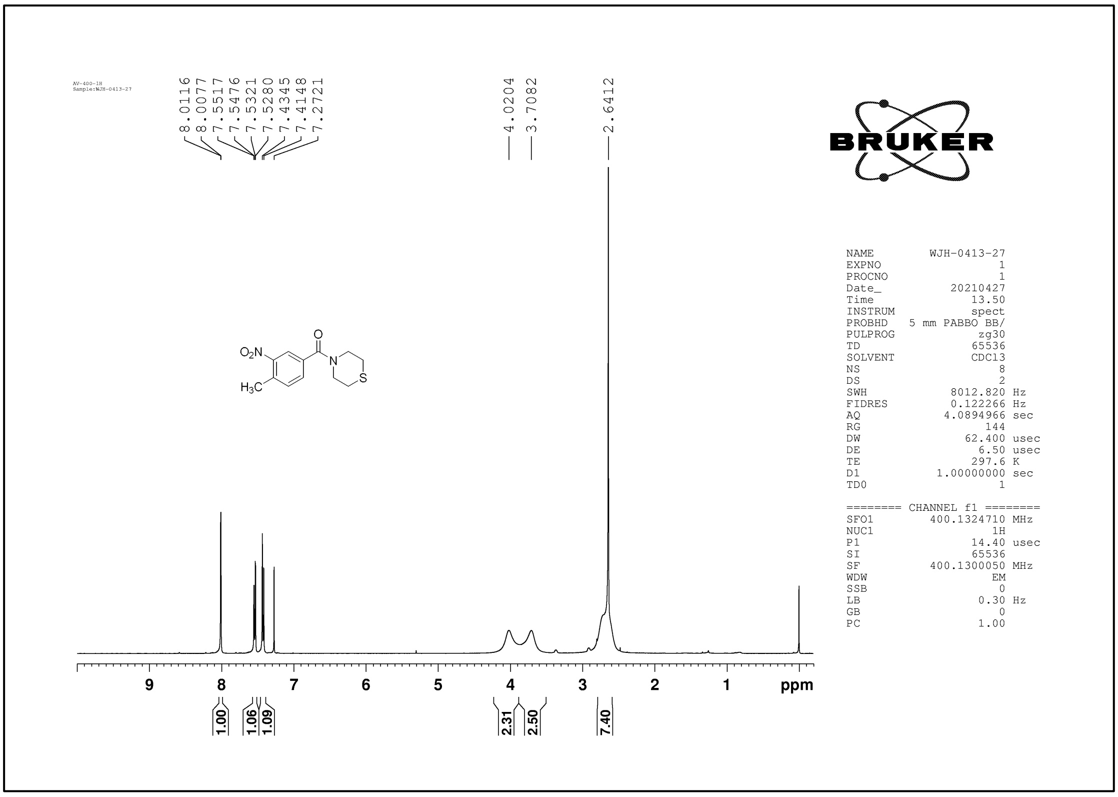

^{1}H-NMR spectrum of compound 13b

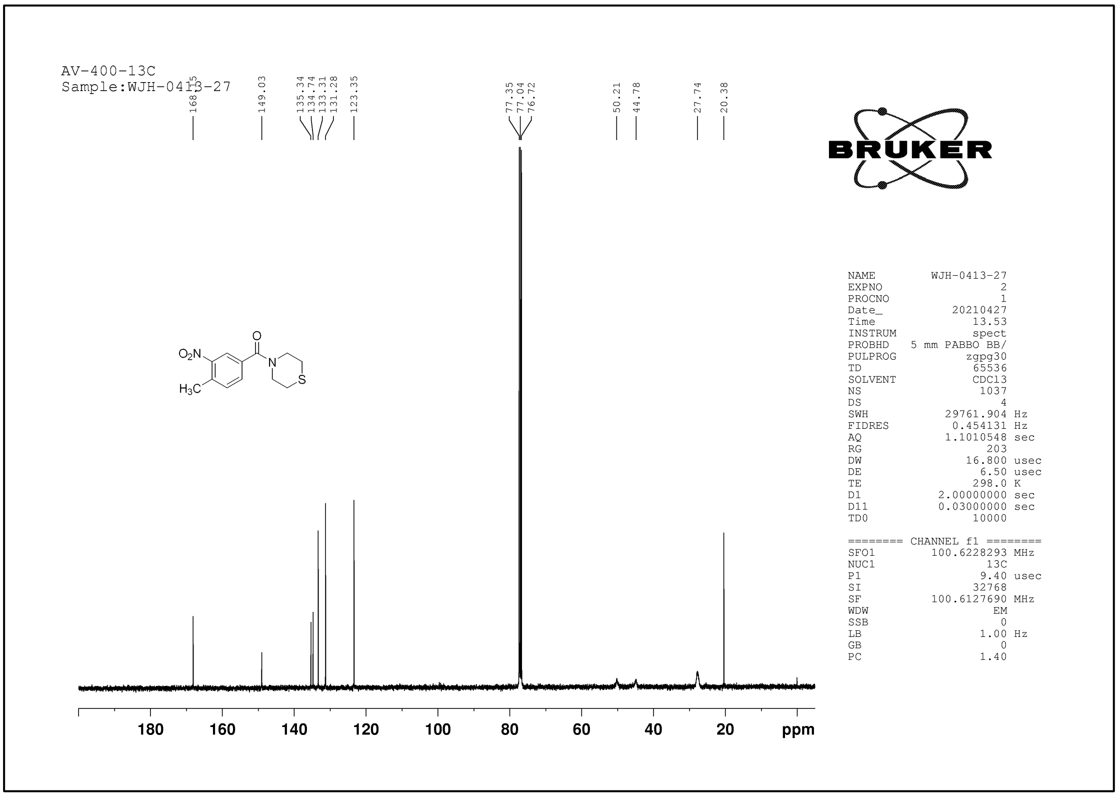

^{13}C-NMR spectrum of compound 13b

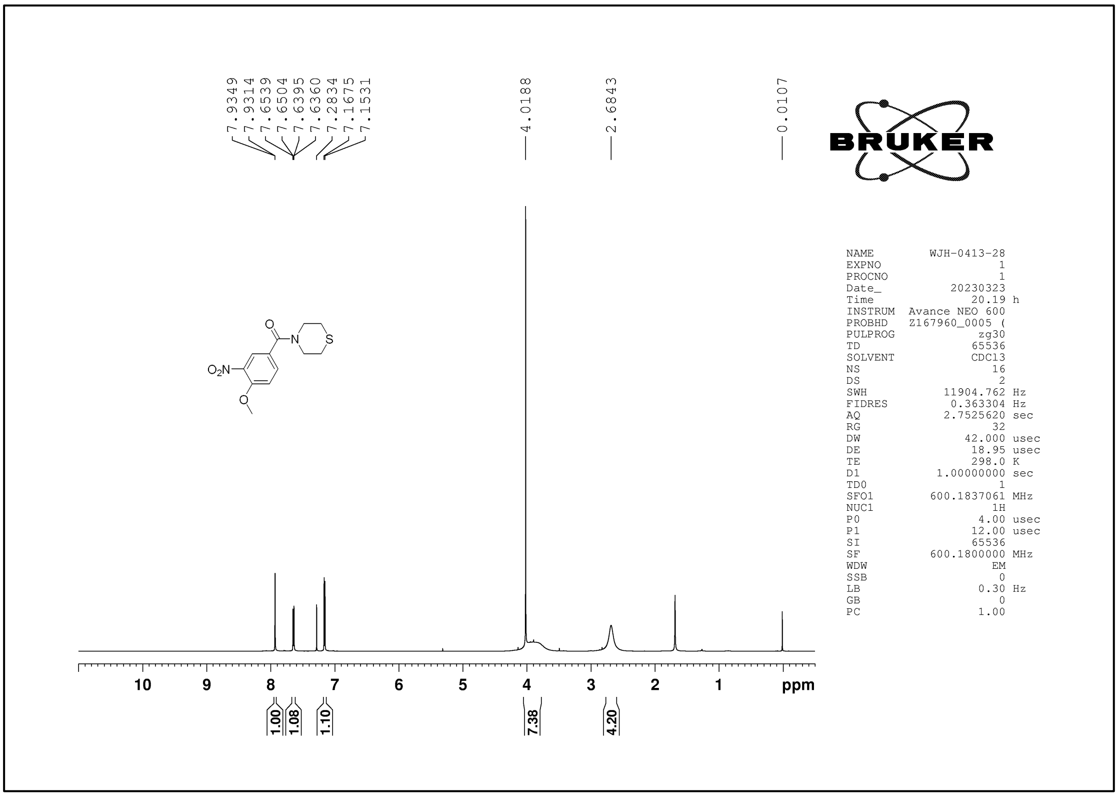

^{1}H-NMR spectrum of compound 13c

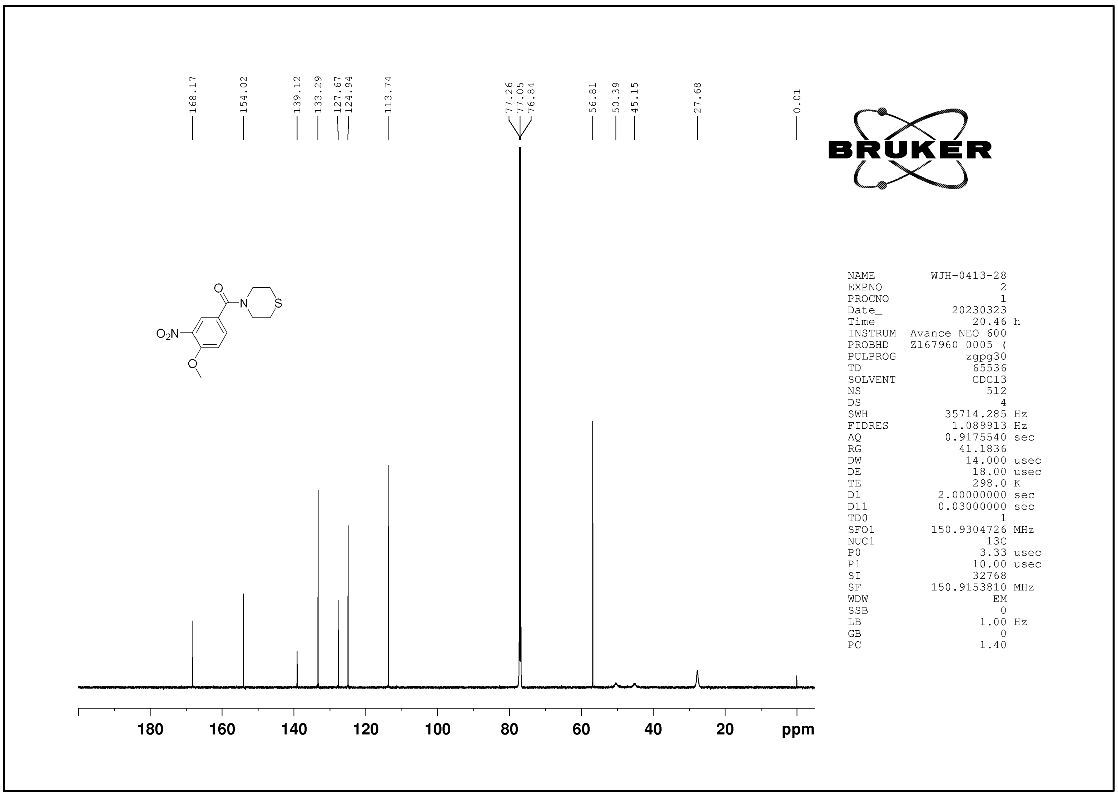

^{13}C-NMR spectrum of compound 13c

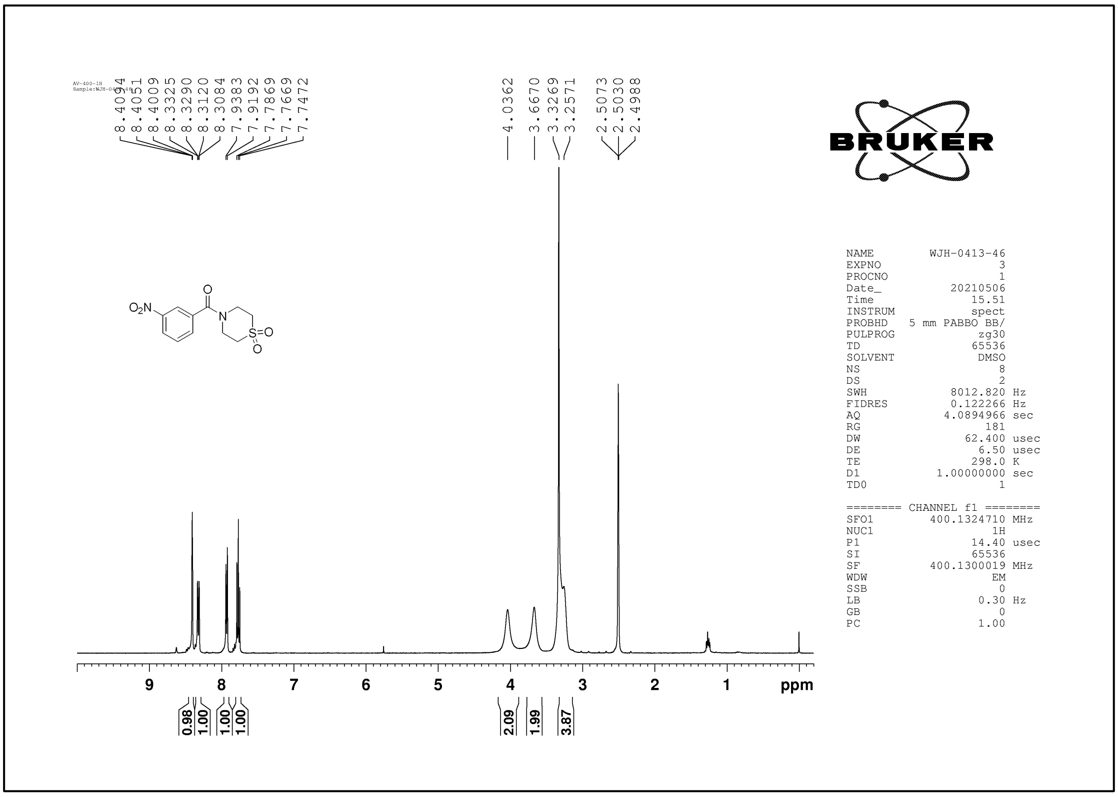

^{1}H-NMR spectrum of compound 13d

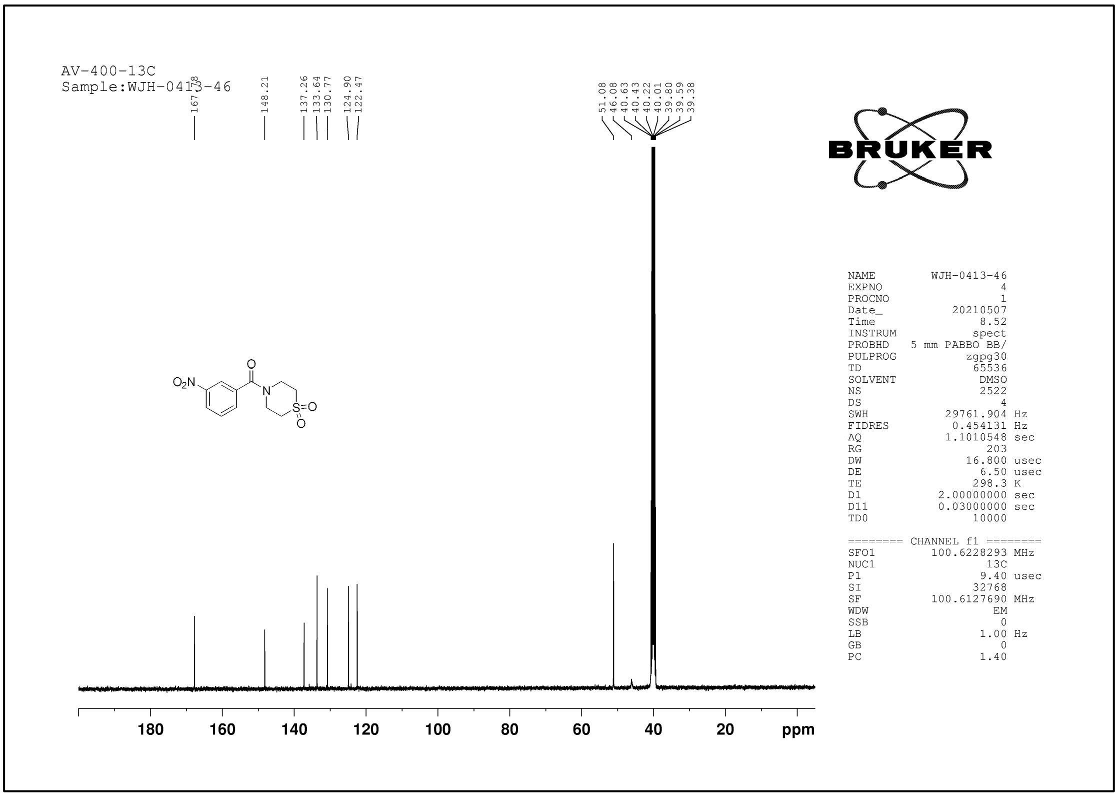

^{13}C-NMR spectrum of compound 13d

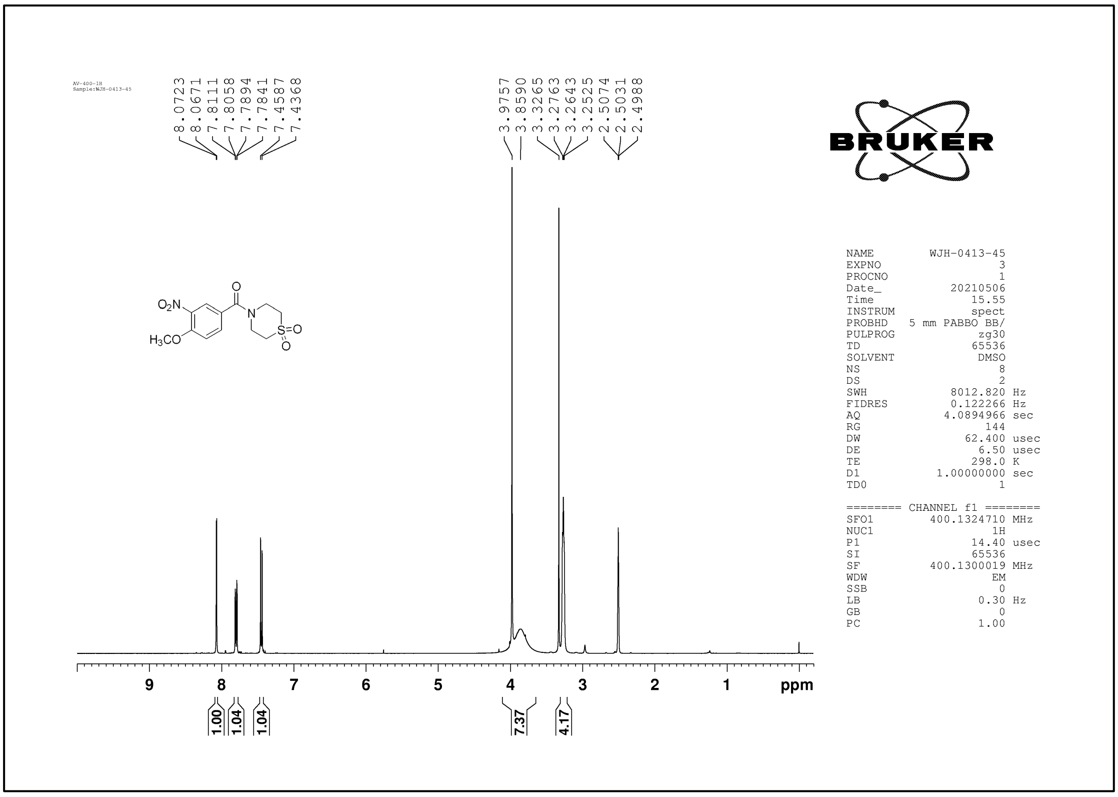

^{1}H-NMR spectrum of compound 13f

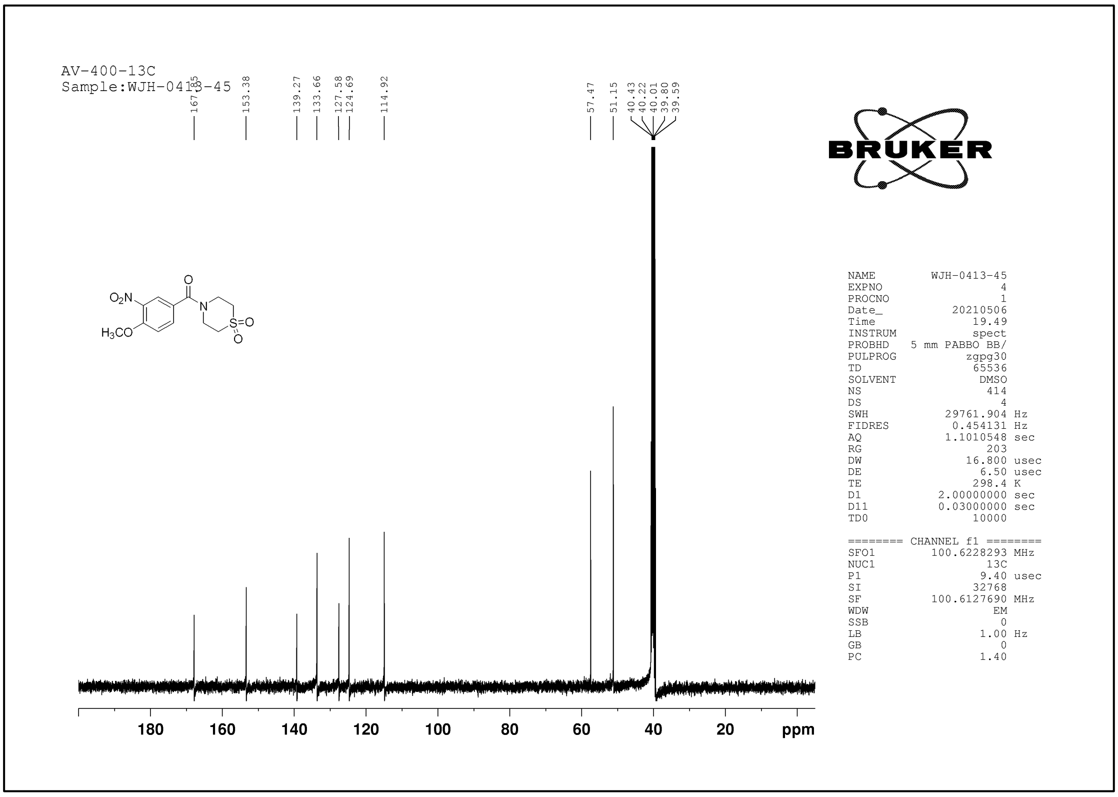

^{13}C-NMR spectrum of compound 13f

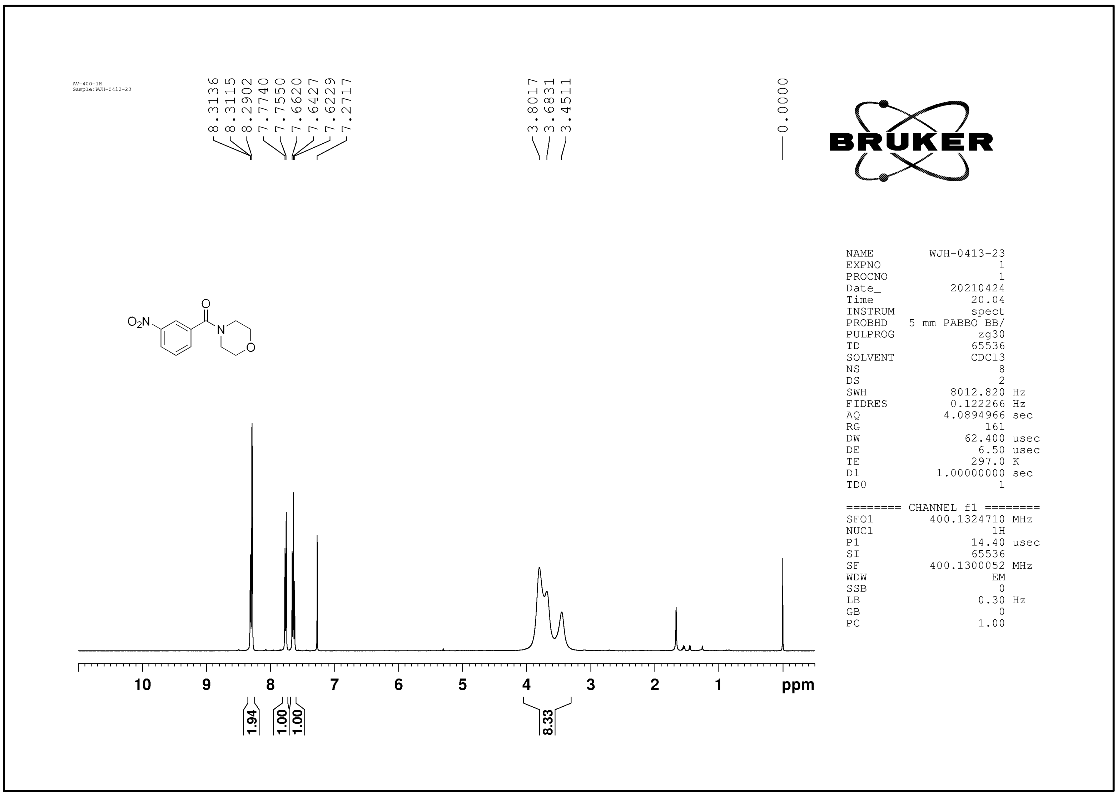

^{1}H-NMR spectrum of compound 13g

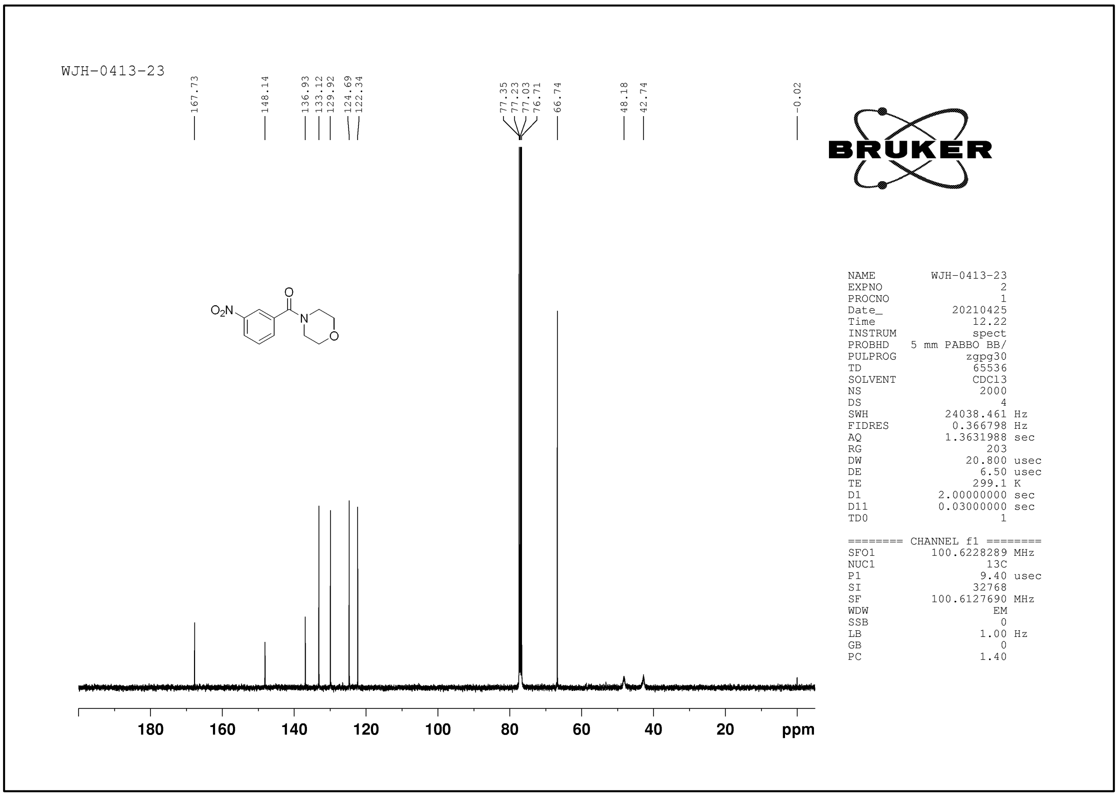

^{13}C-NMR spectrum of compound 13g

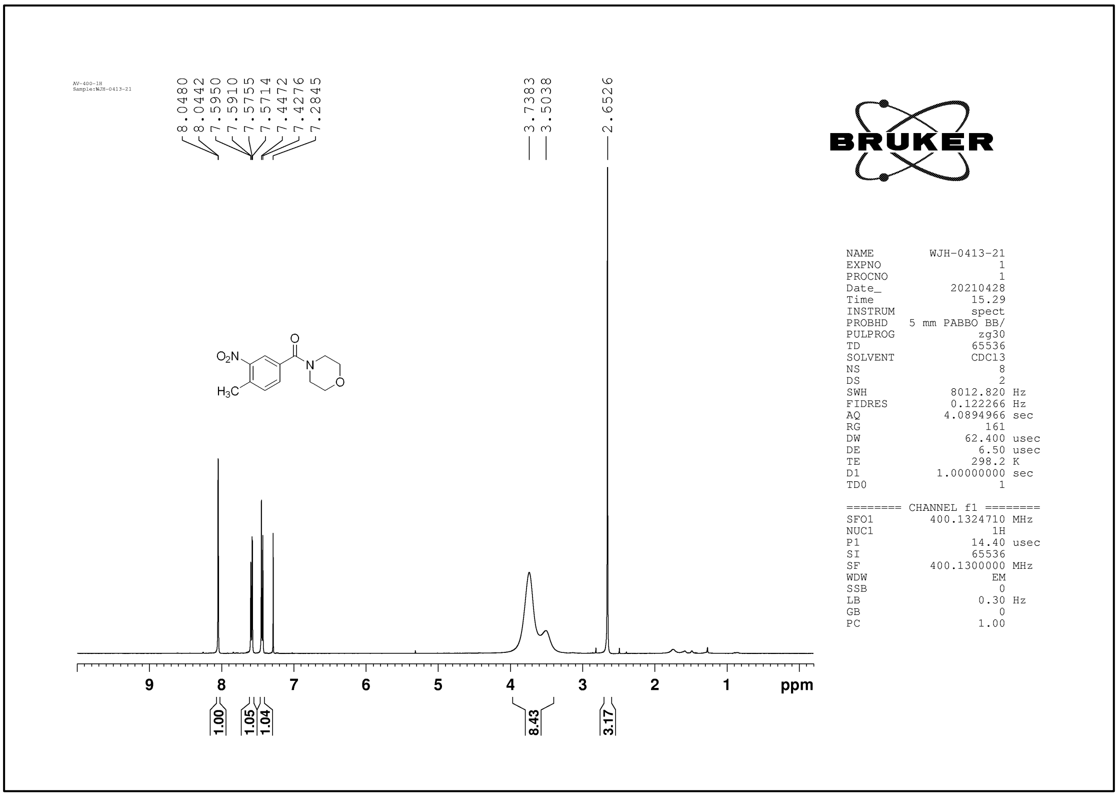

^{1}H-NMR spectrum of compound 13h

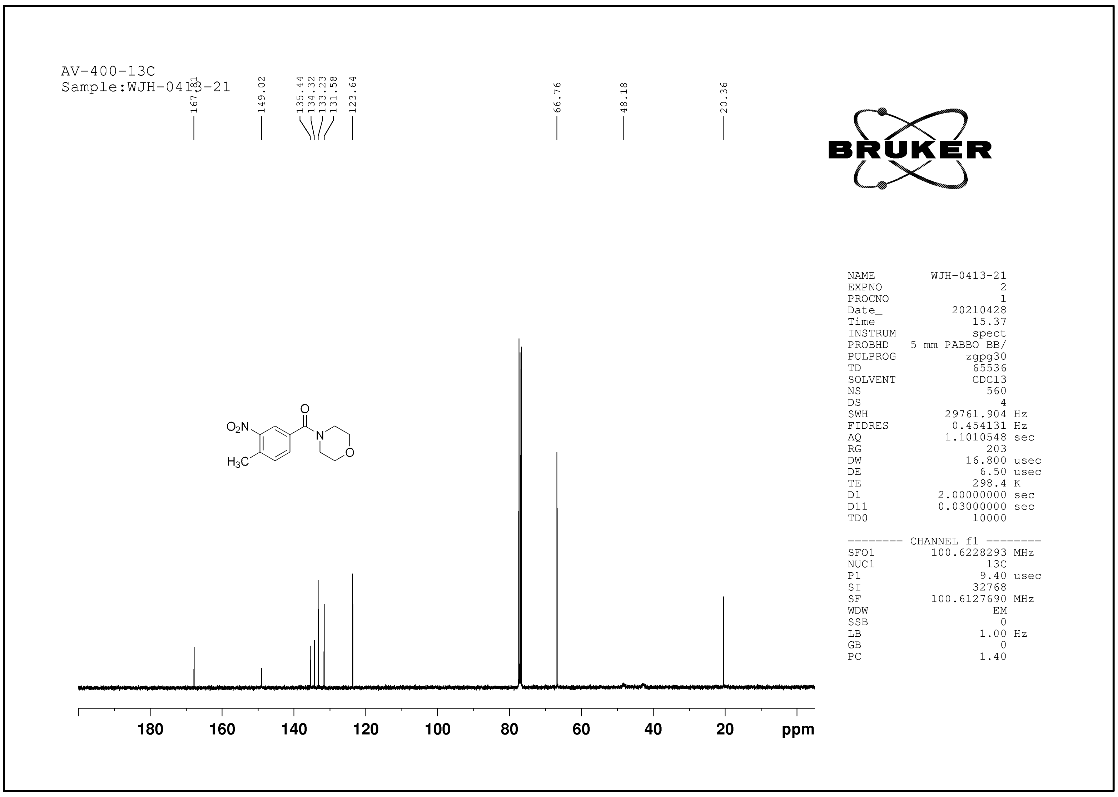

^{13}C-NMR spectrum of compound 13h

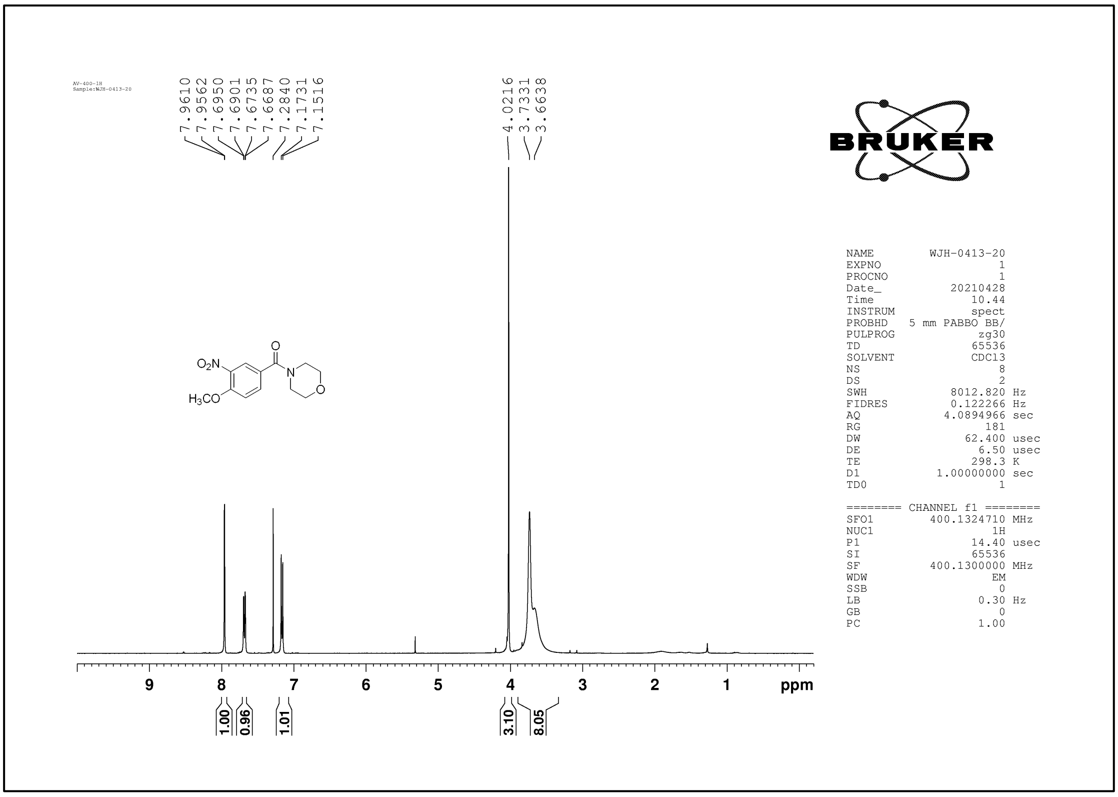

^{1}H-NMR spectrum of compound 13i

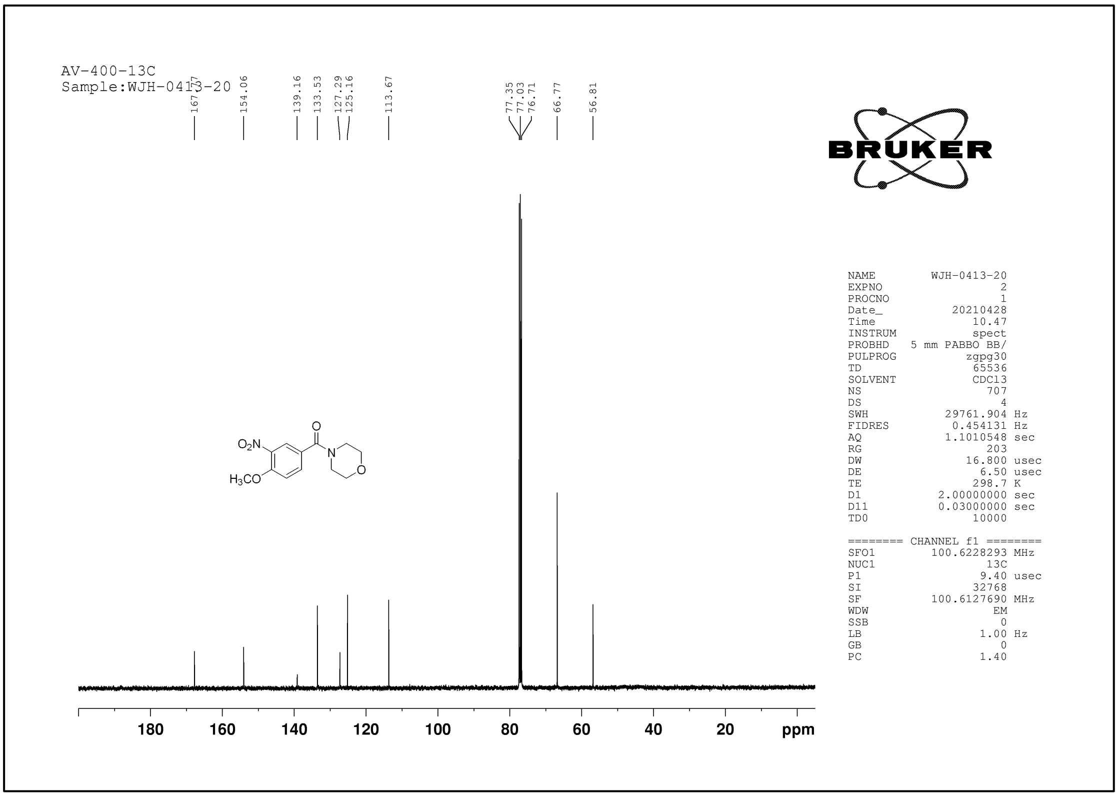

^{13}C-NMR spectrum of compound 13i

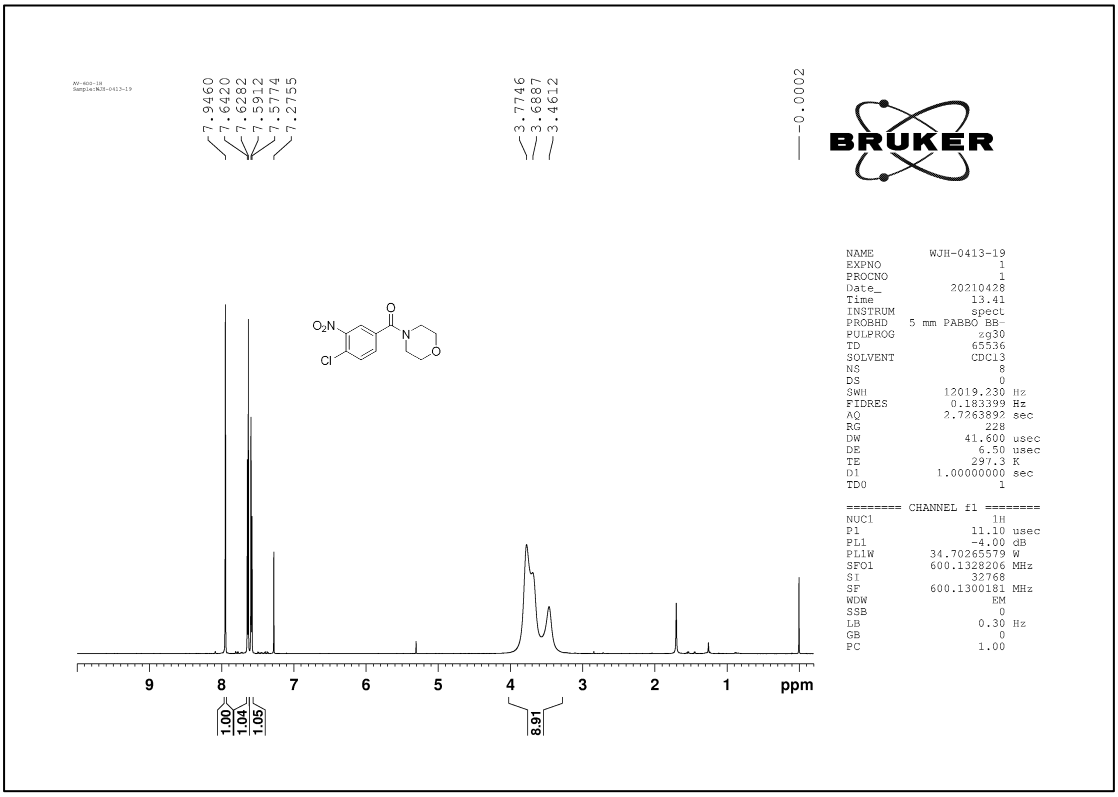

^{1}H-NMR spectrum of compound 13j

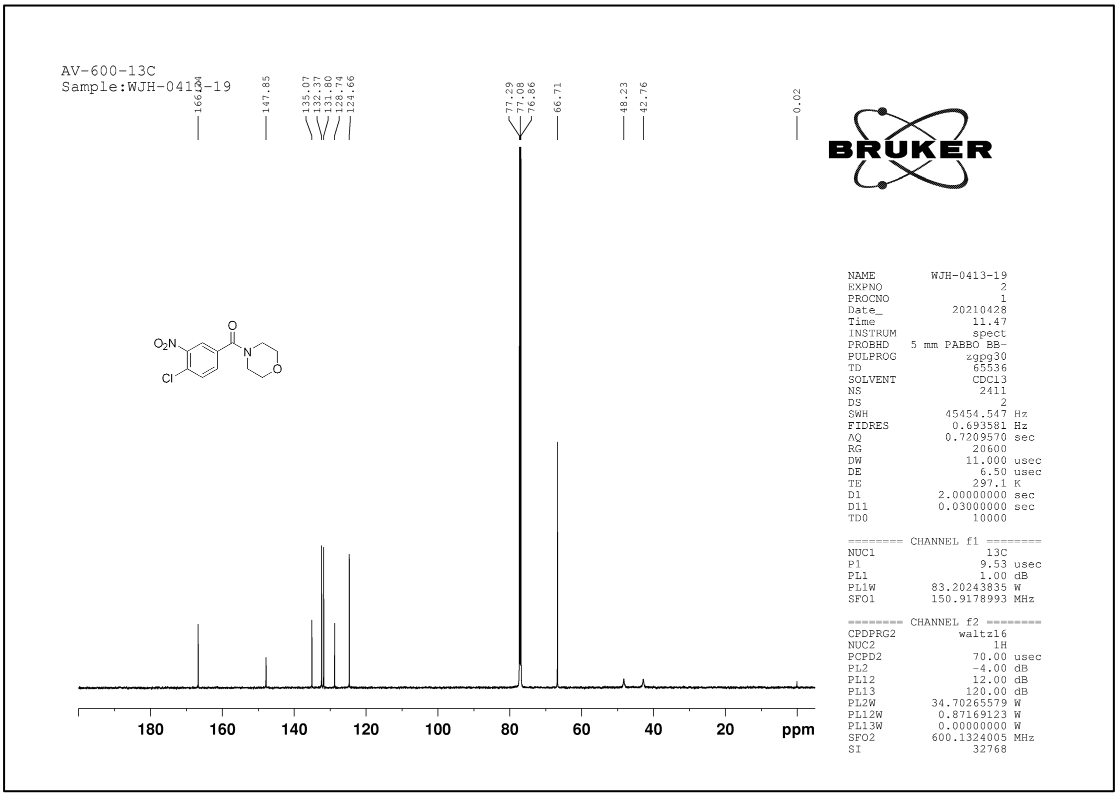

^{13}C-NMR spectrum of compound 13j

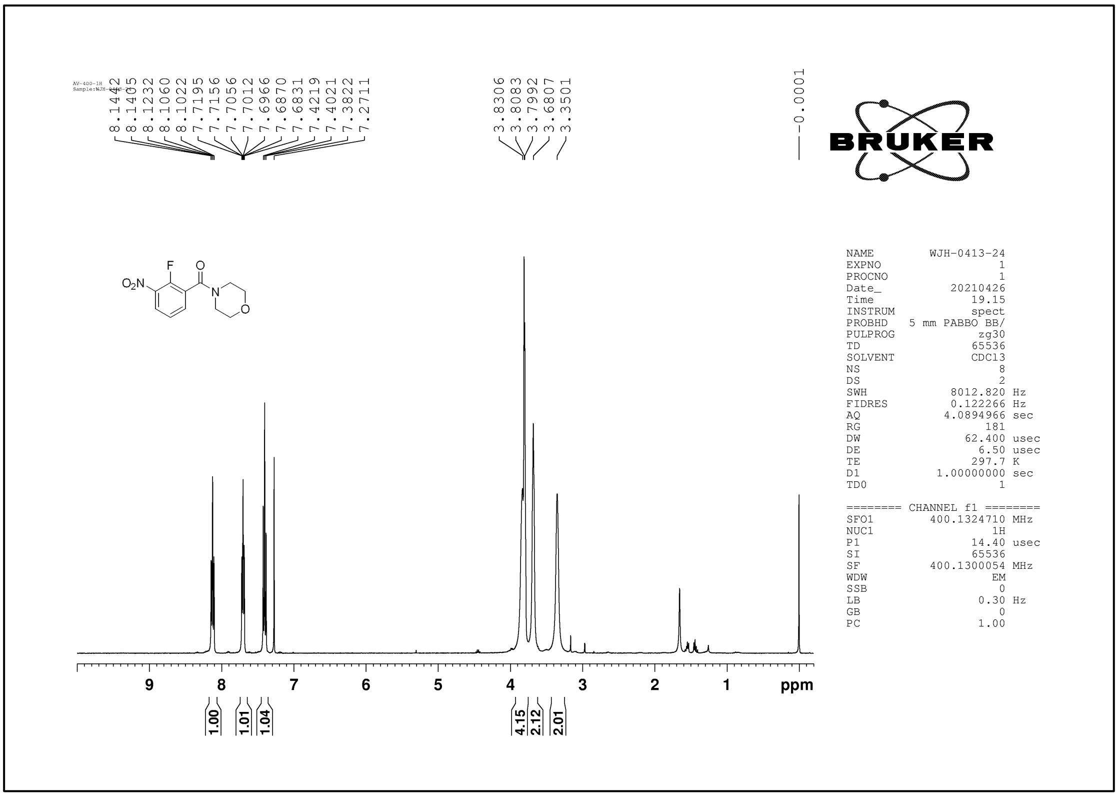

^{1}H-NMR spectrum of compound 13k

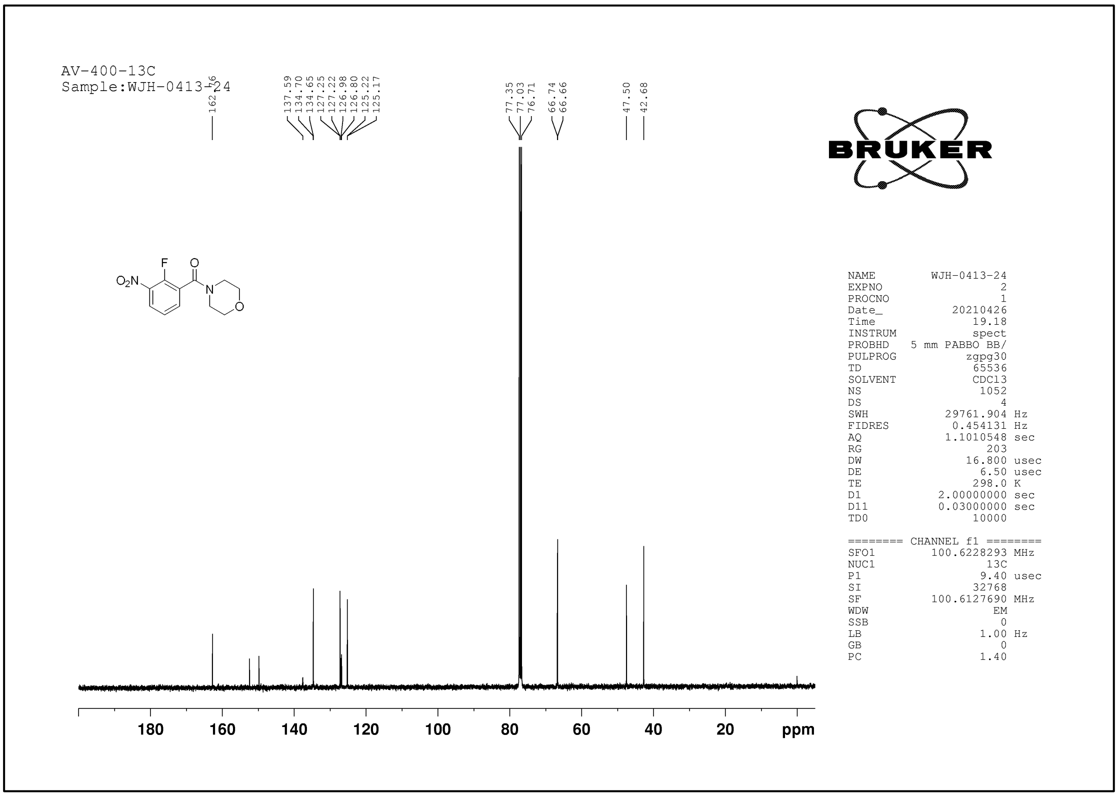

^{13}C-NMR spectrum of compound 13k

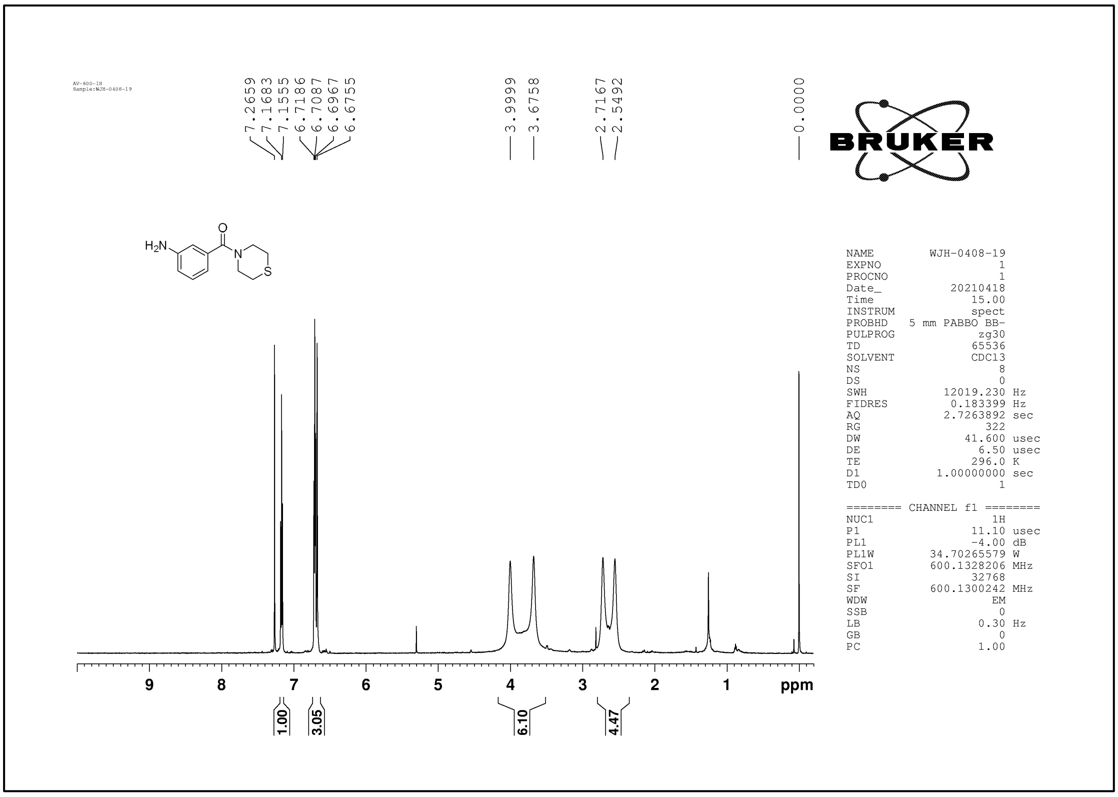

^{1}H-NMR spectrum of compound 14a

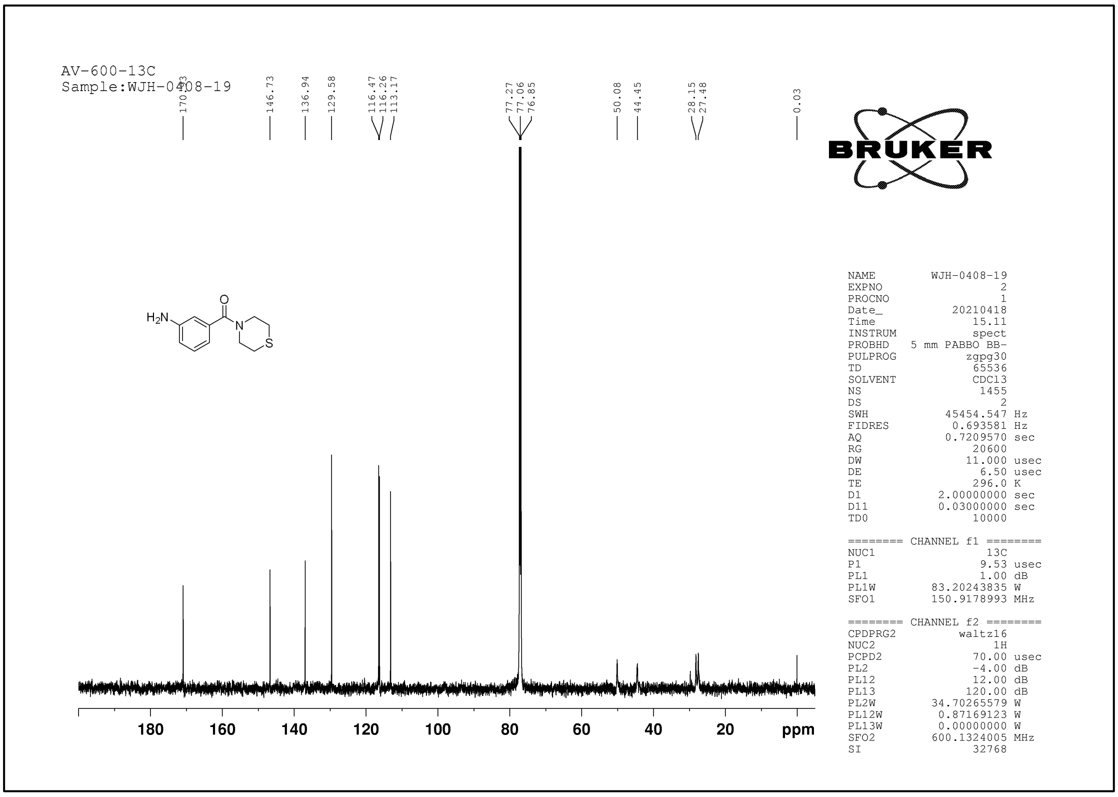

^{13}C-NMR spectrum of compound 14a

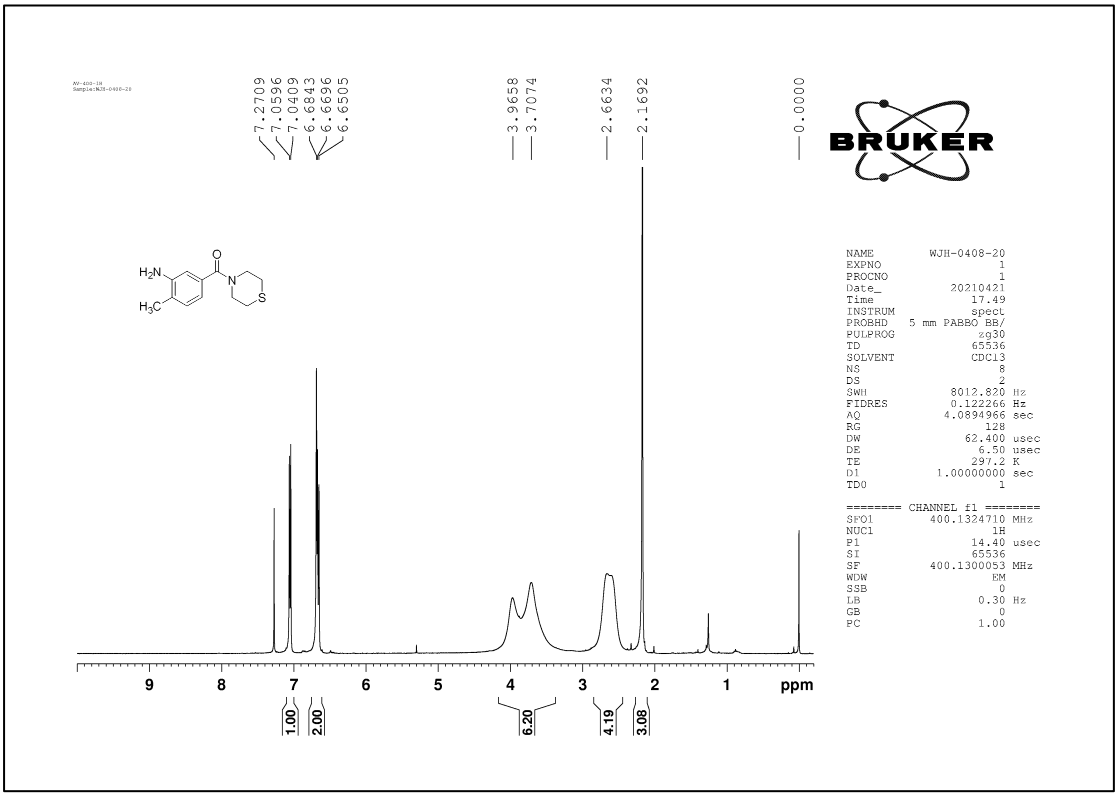

^{1}H-NMR spectrum of compound 14b

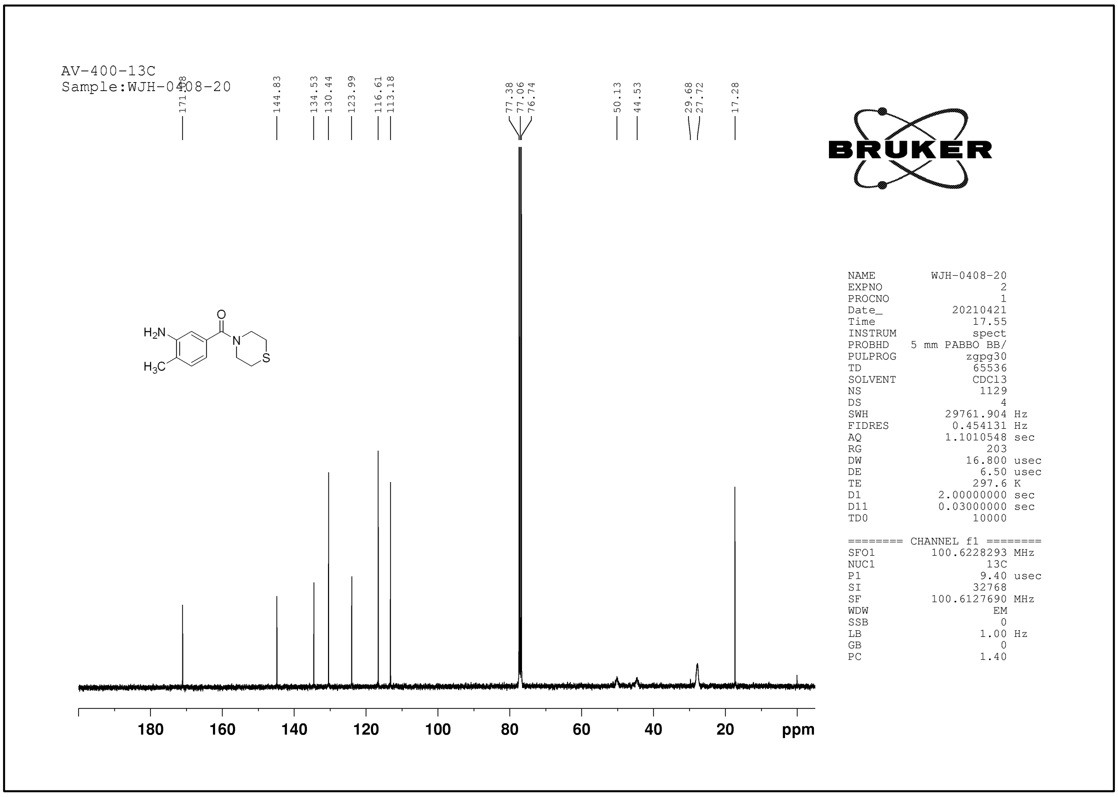

^{13}C-NMR spectrum of compound 14b

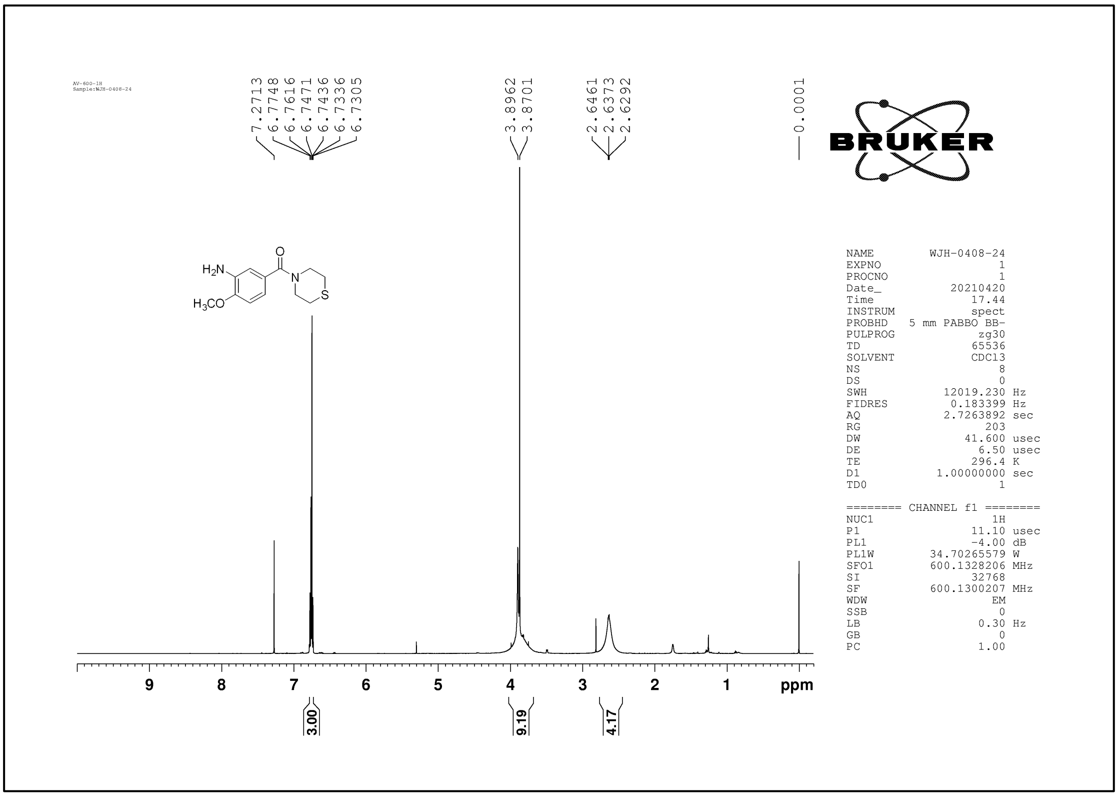

^{1}H-NMR spectrum of compound 14c

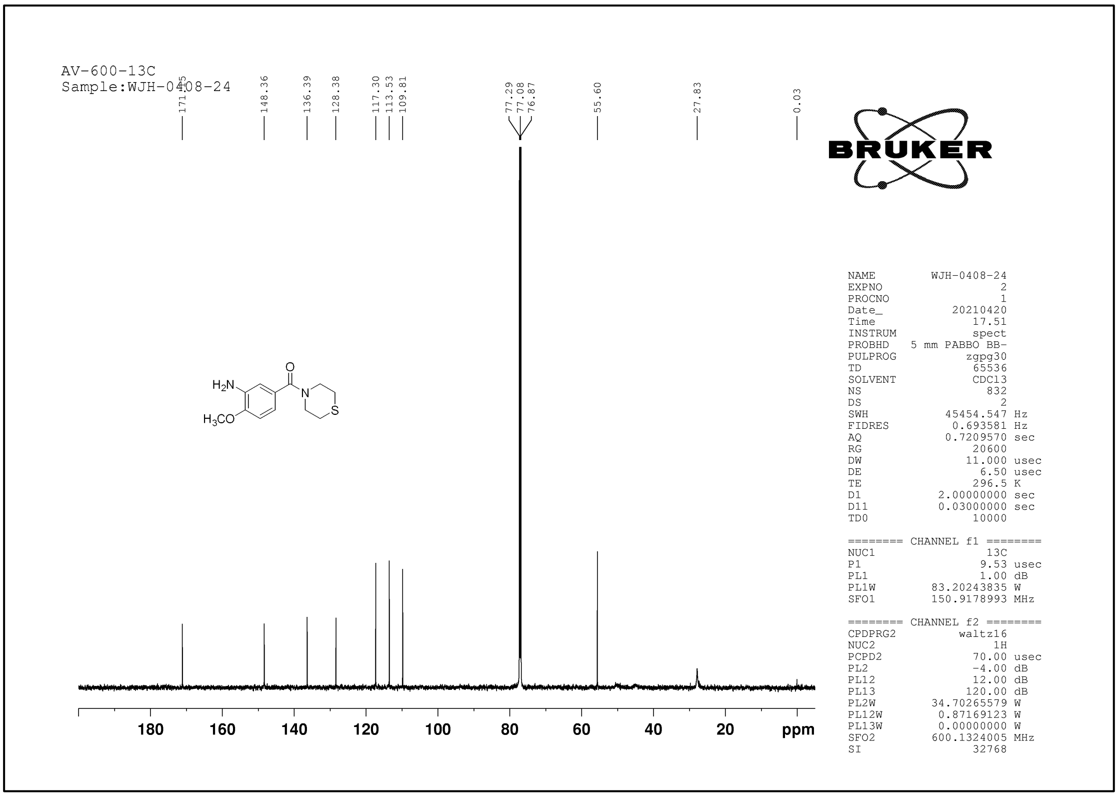

^{13}C-NMR spectrum of compound 14c

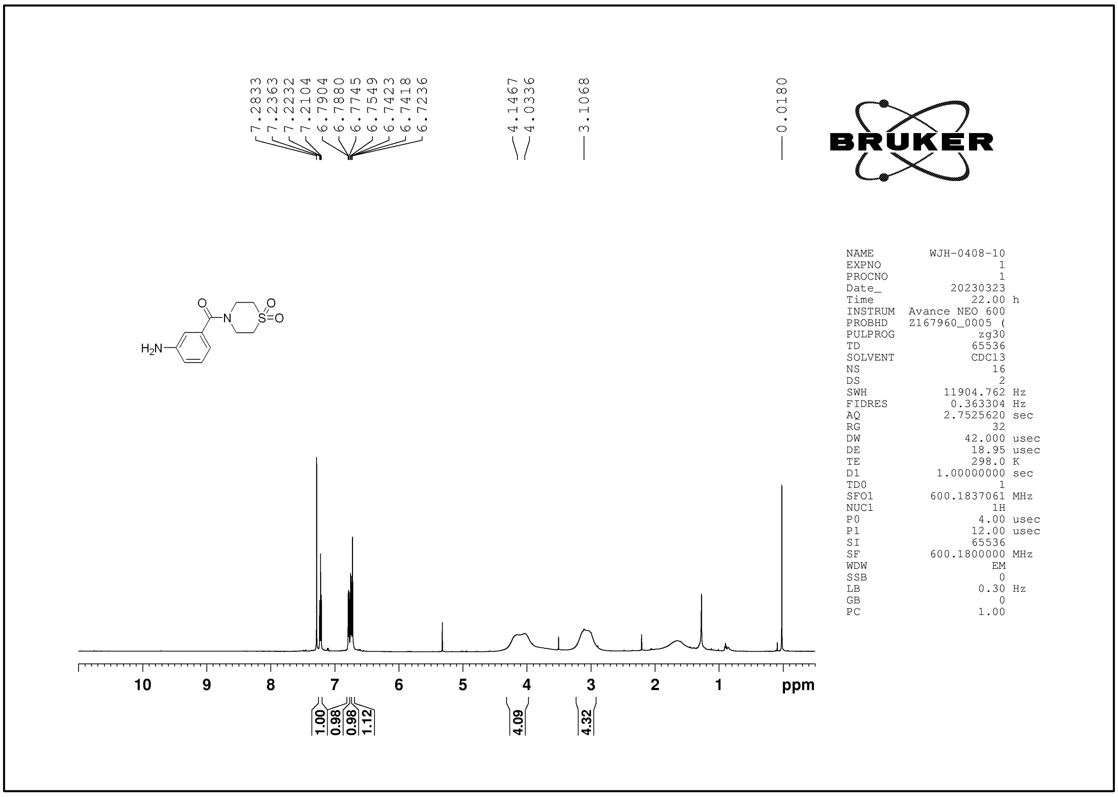

^{1}H-NMR spectrum of compound 14d

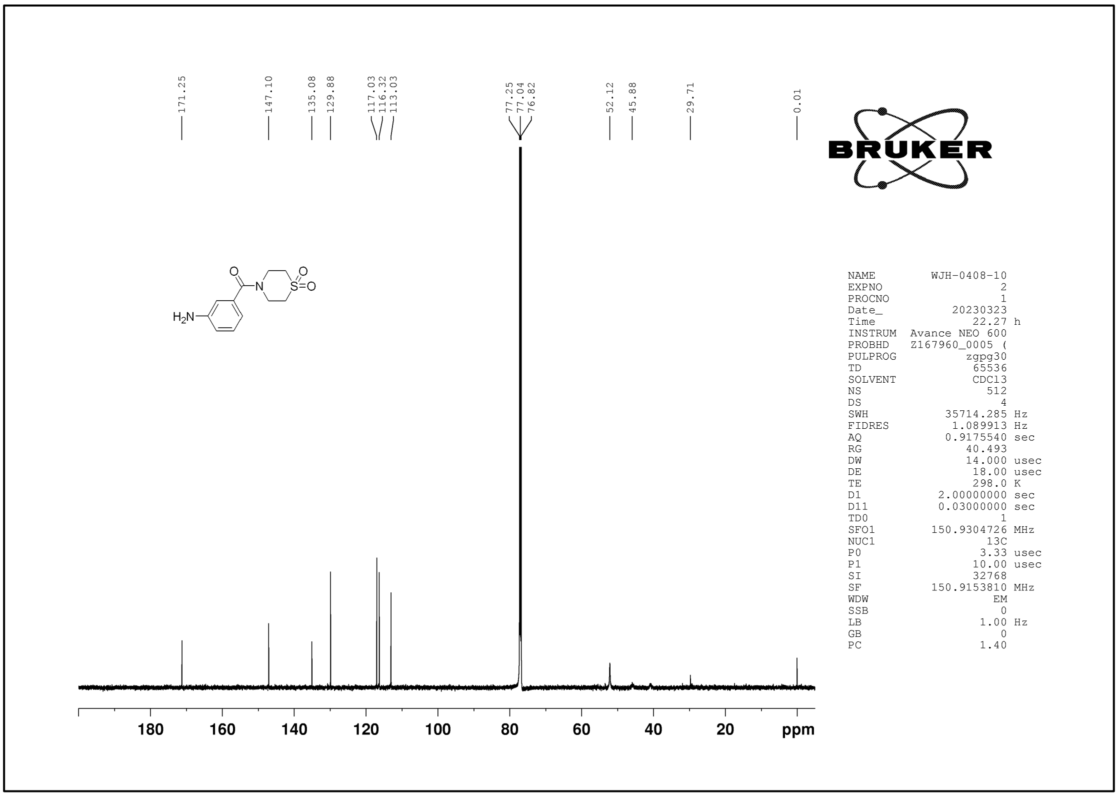

^{13}C-NMR spectrum of compound 14d

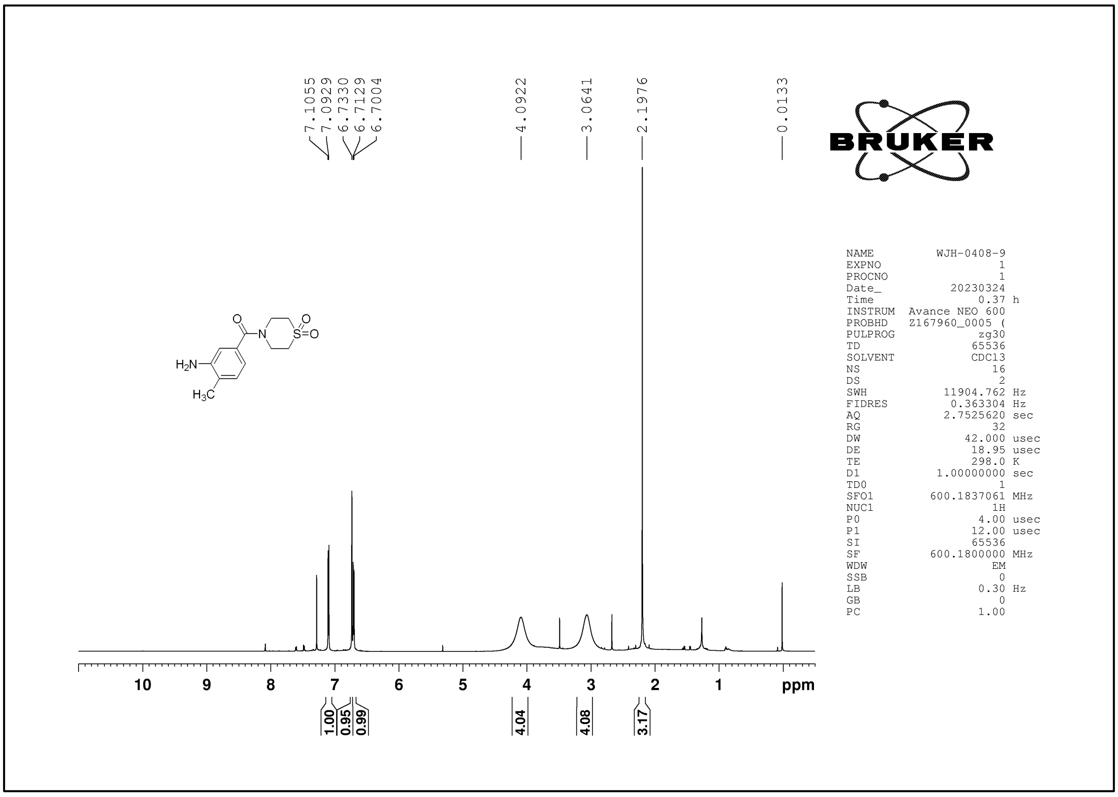

^{1}H-NMR spectrum of compound 14e

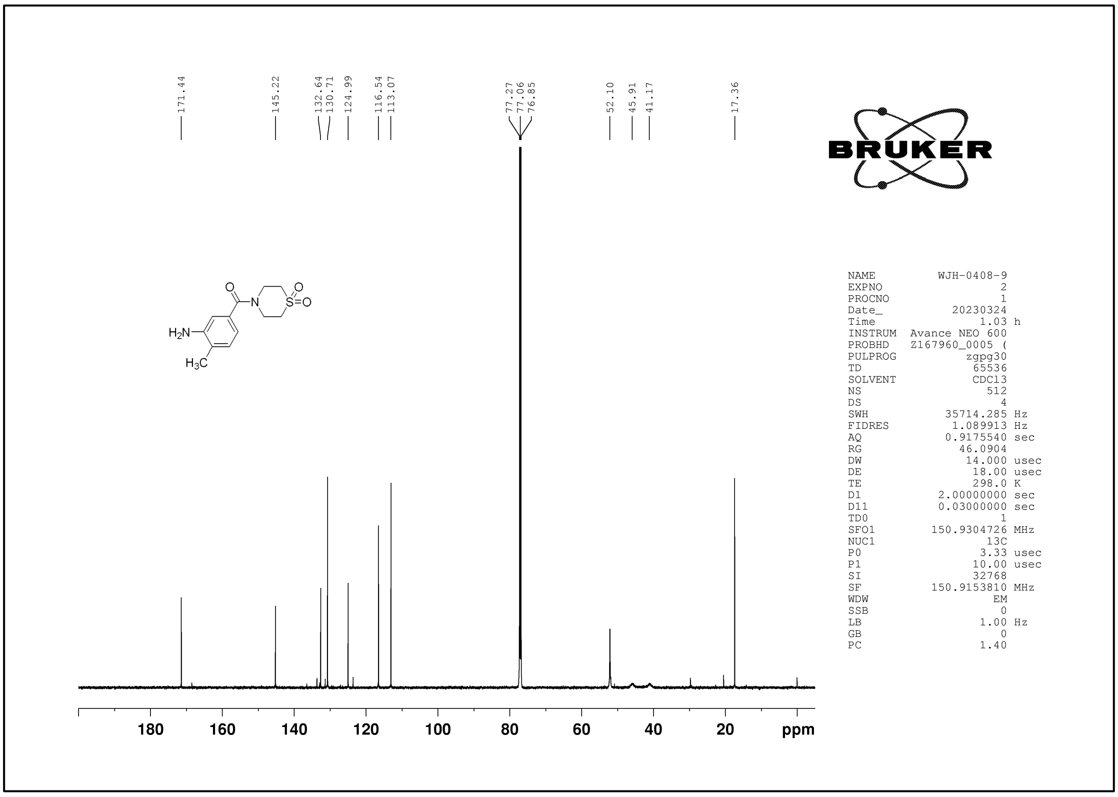

^{13}C-NMR spectrum of compound 14e

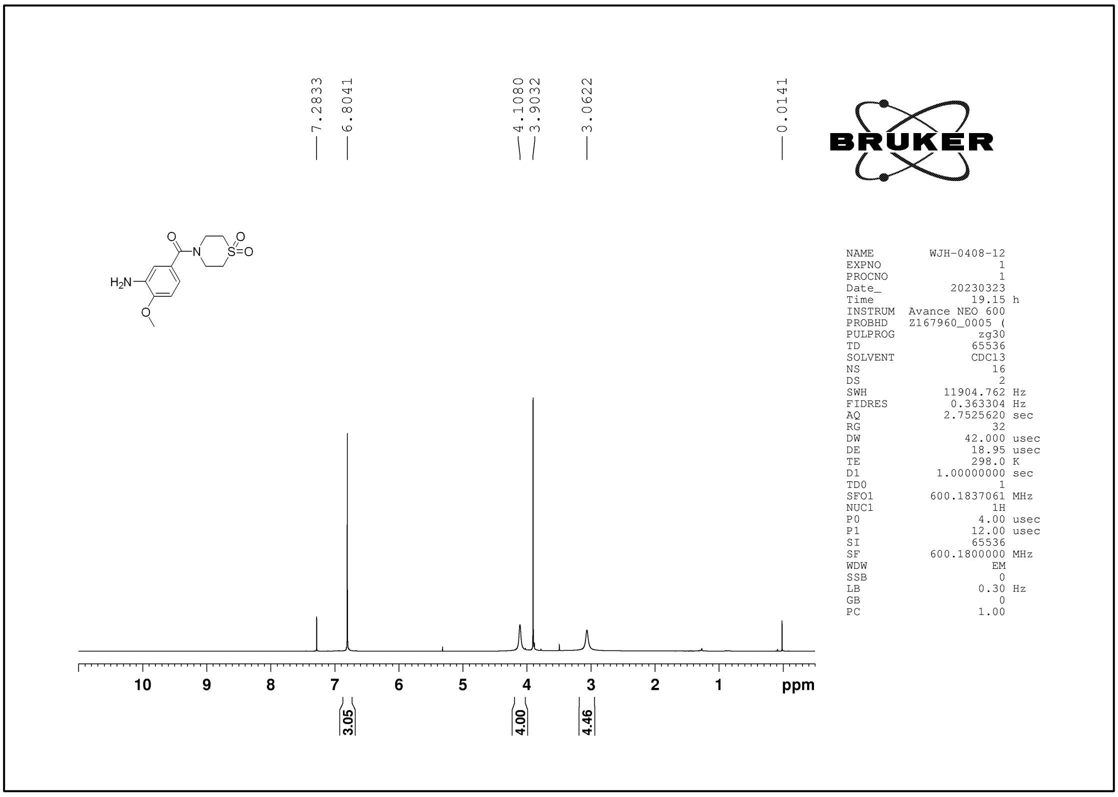

^{1}H-NMR spectrum of compound 14f

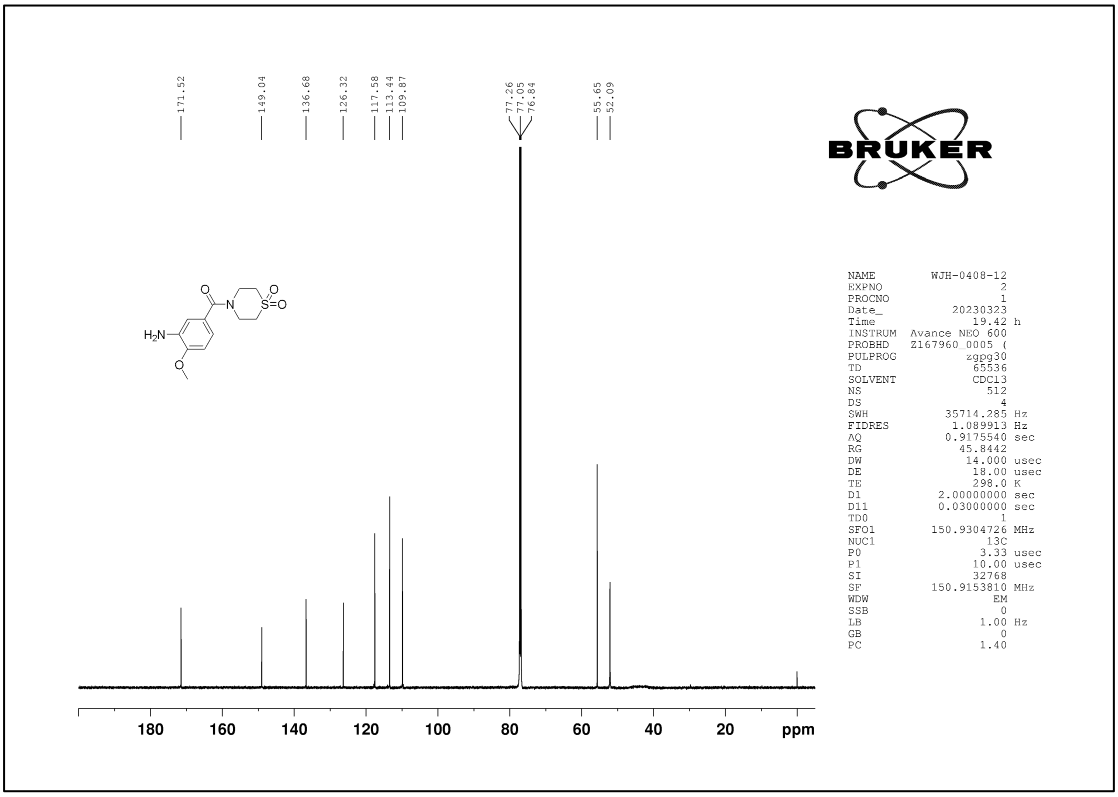

^{13}C-NMR spectrum of compound 14f

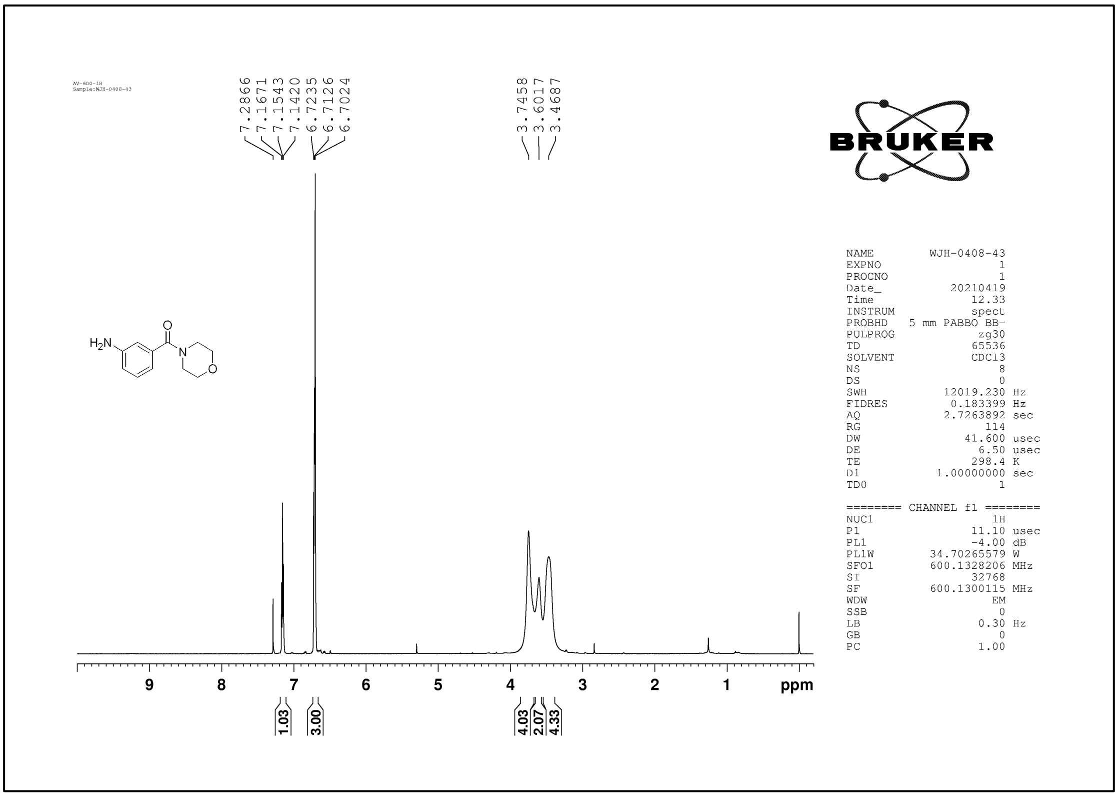

^{1}H-NMR spectrum of compound 14g

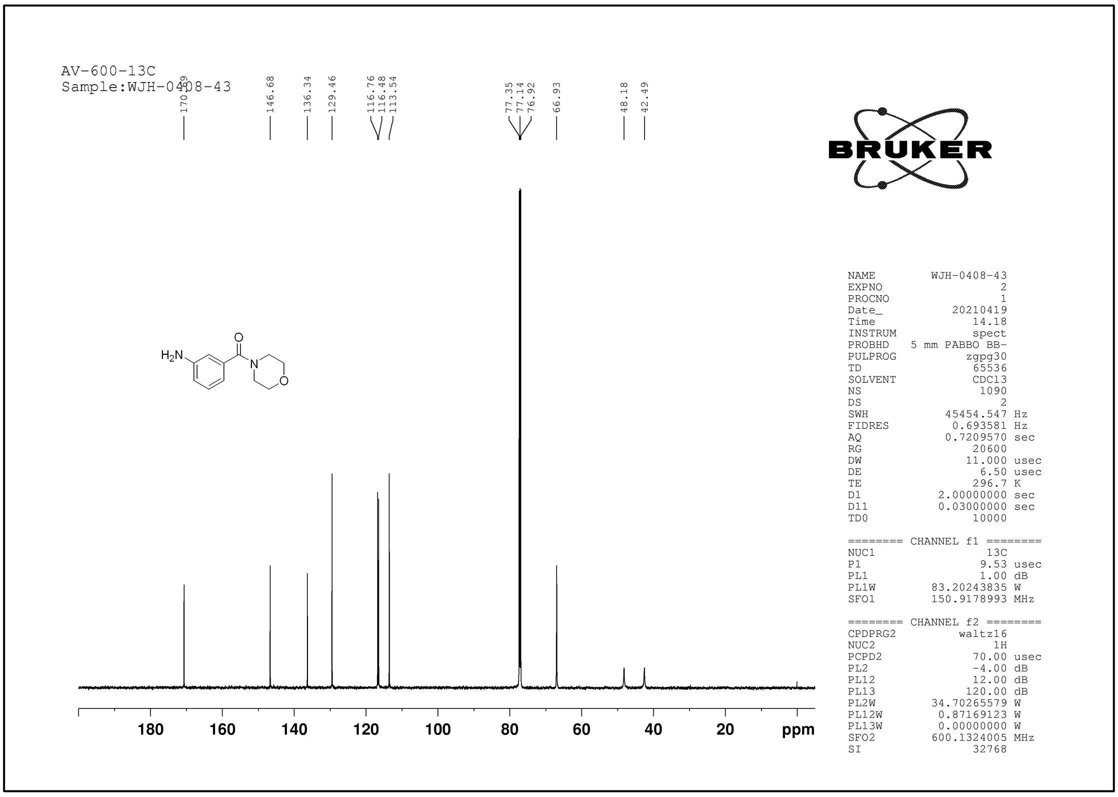

^{13}C-NMR spectrum of compound 14g

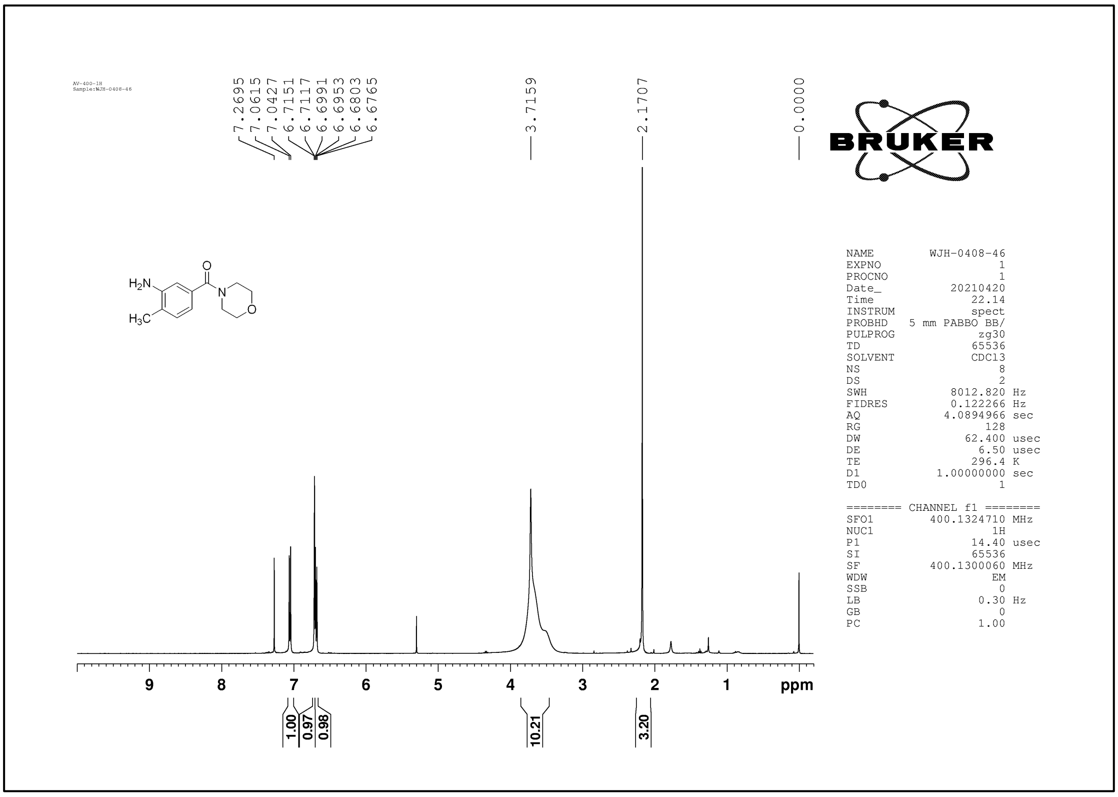

^{1}H-NMR spectrum of compound 14h

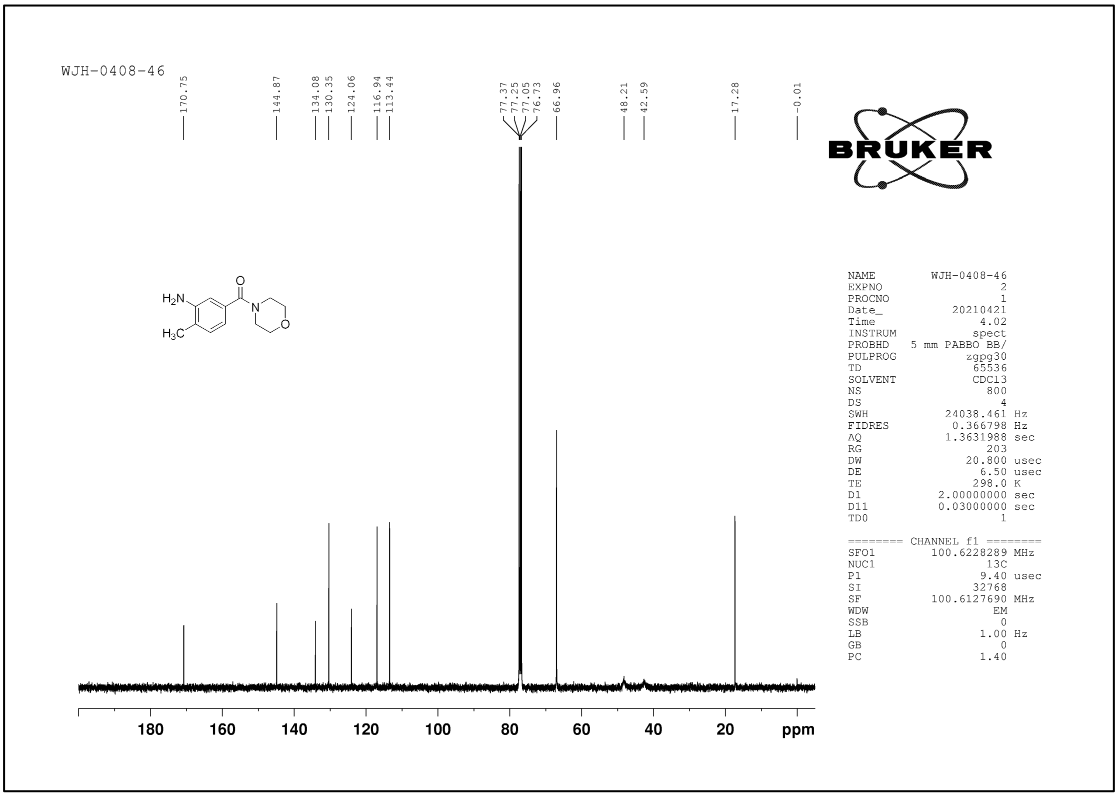

^{13}C-NMR spectrum of compound 14h

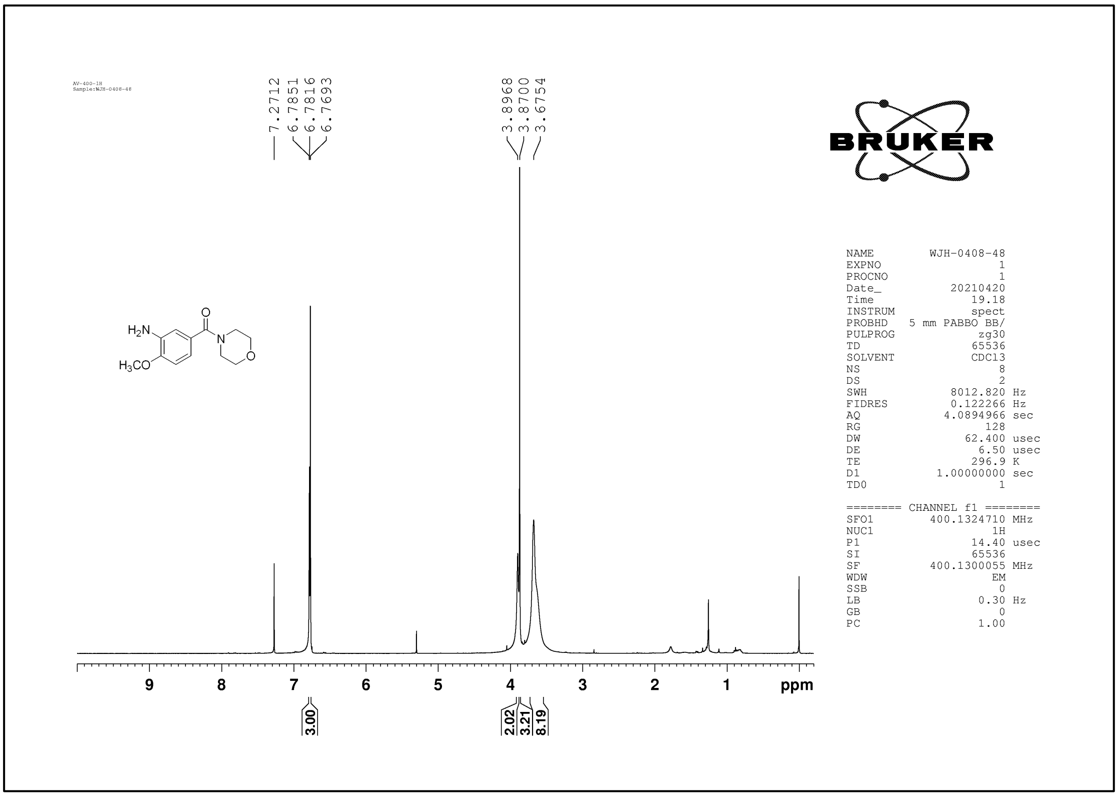

^{1}H-NMR spectrum of compound 14i

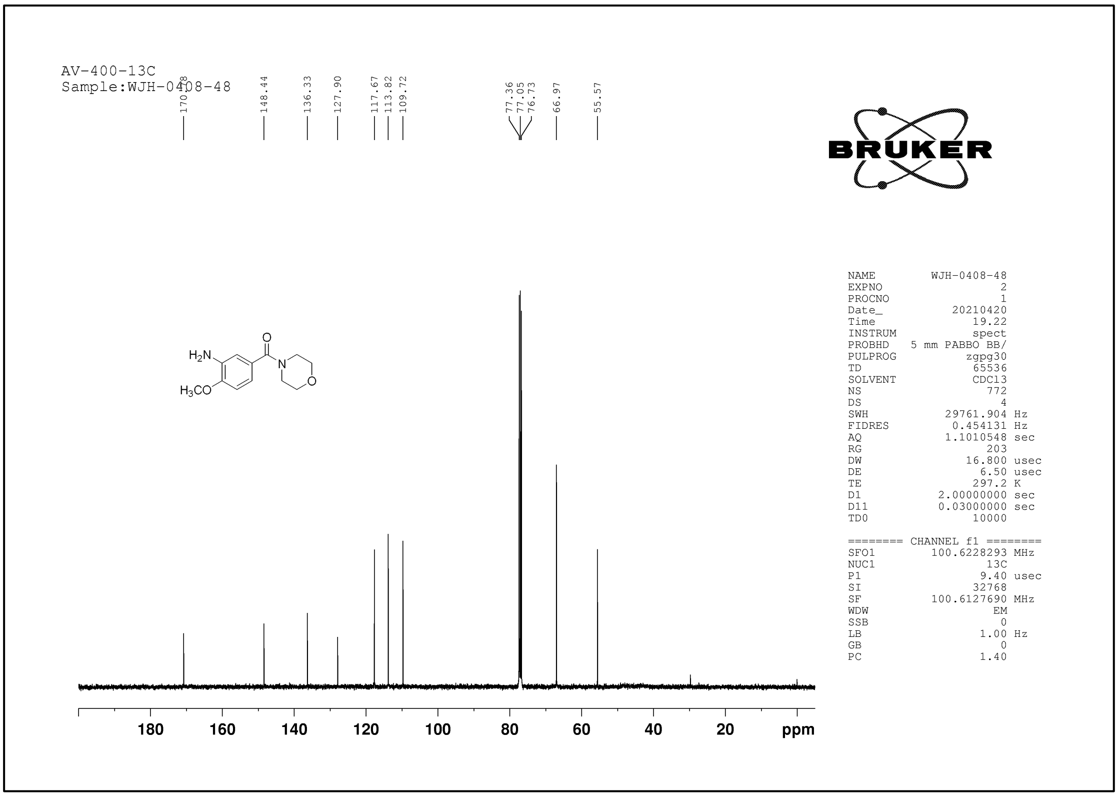

^{13}C-NMR spectrum of compound 14i

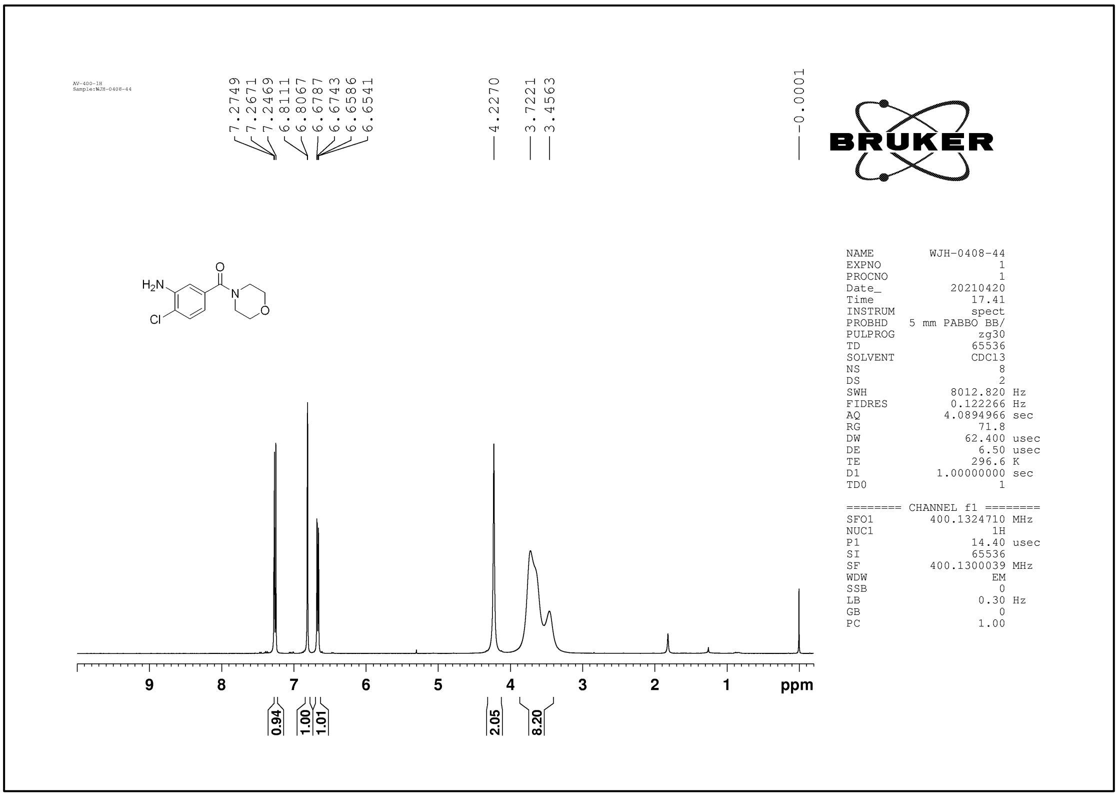

^{1}H-NMR spectrum of compound 14j

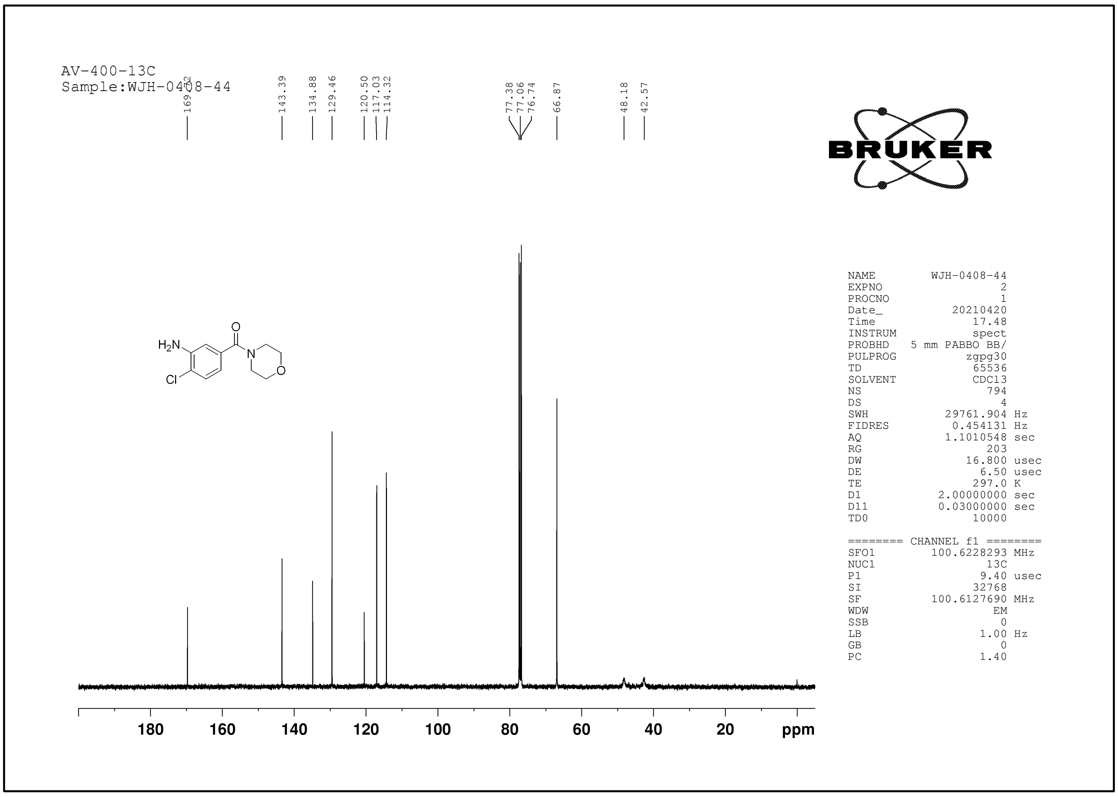

^{13}C-NMR spectrum of compound 14j

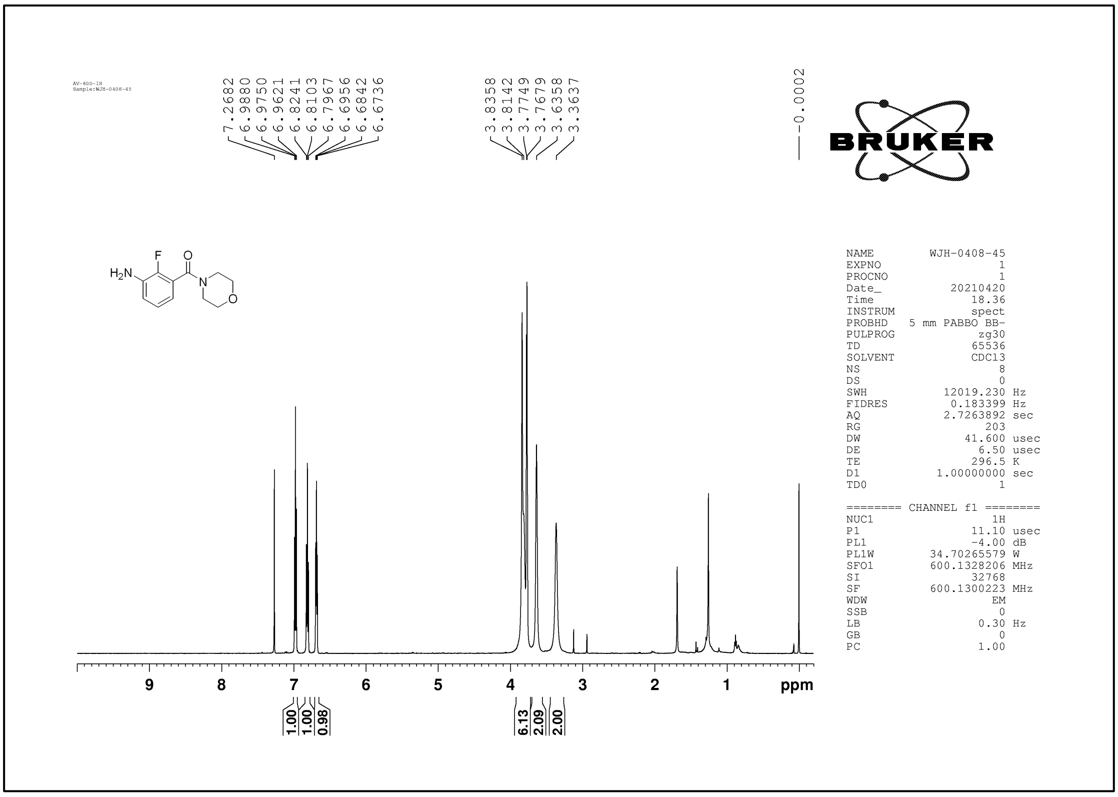

^{1}H-NMR spectrum of compound 14k

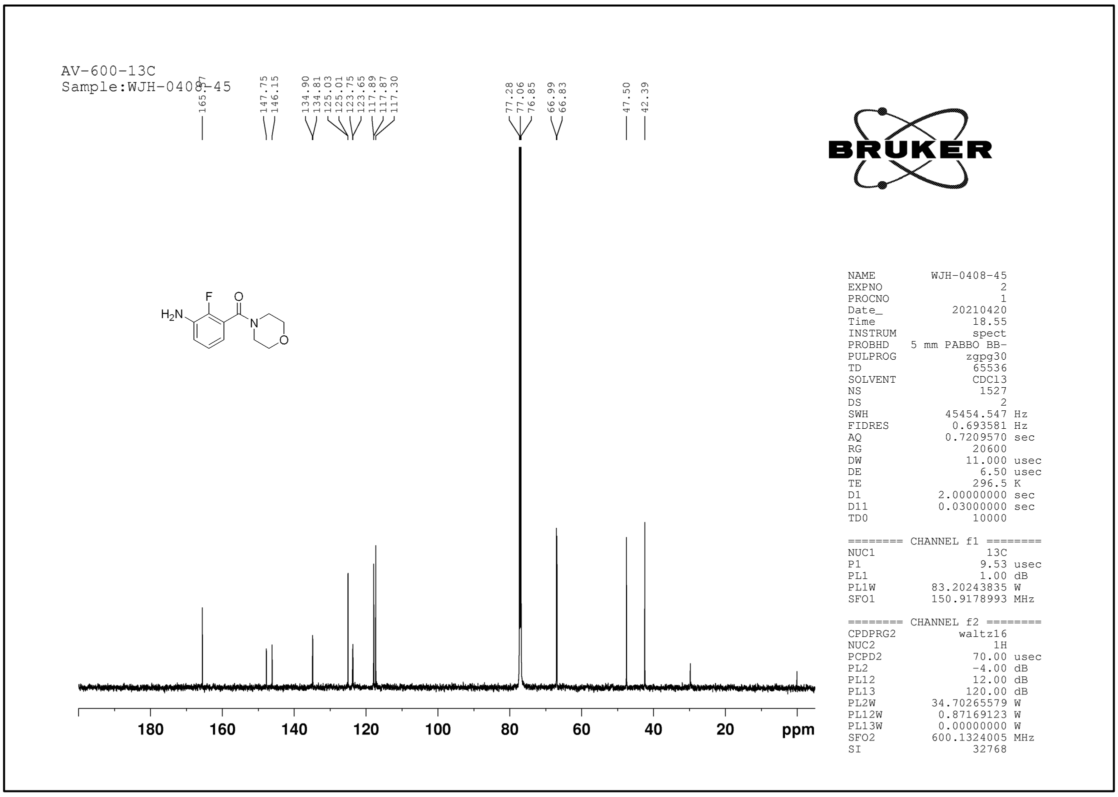

^{13}C-NMR spectrum of compound 14k

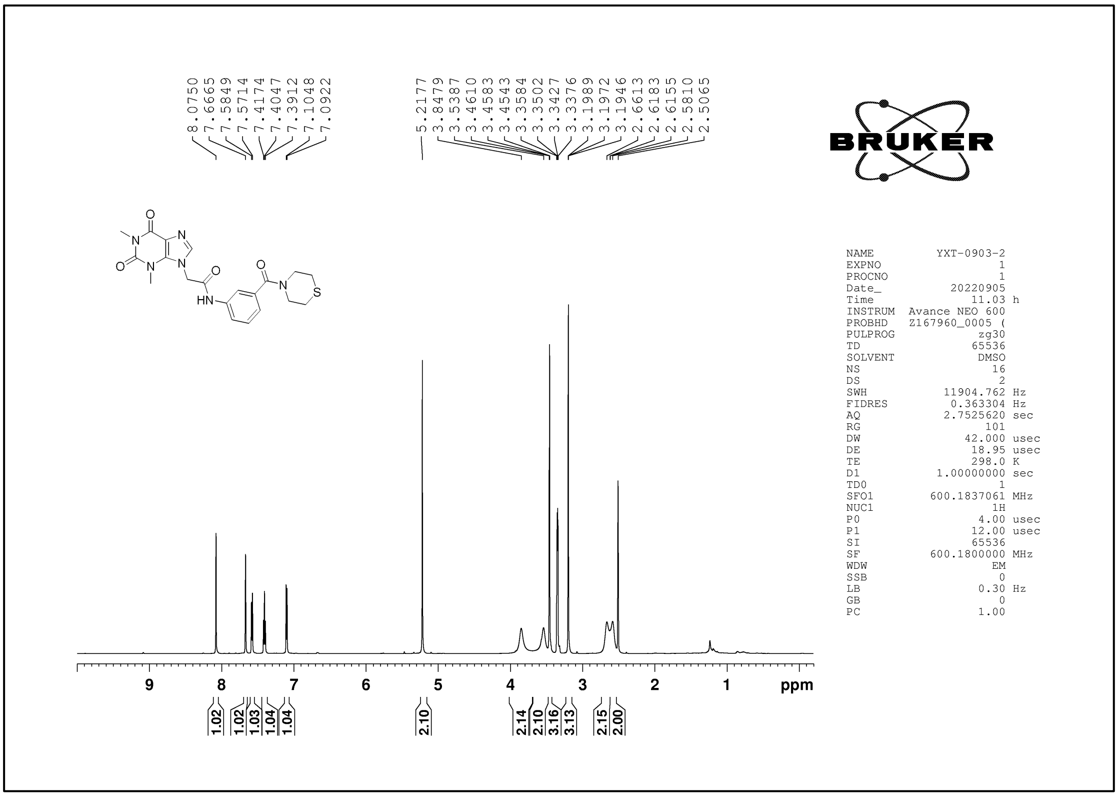

^{1}H-NMR spectrum of compound 15a

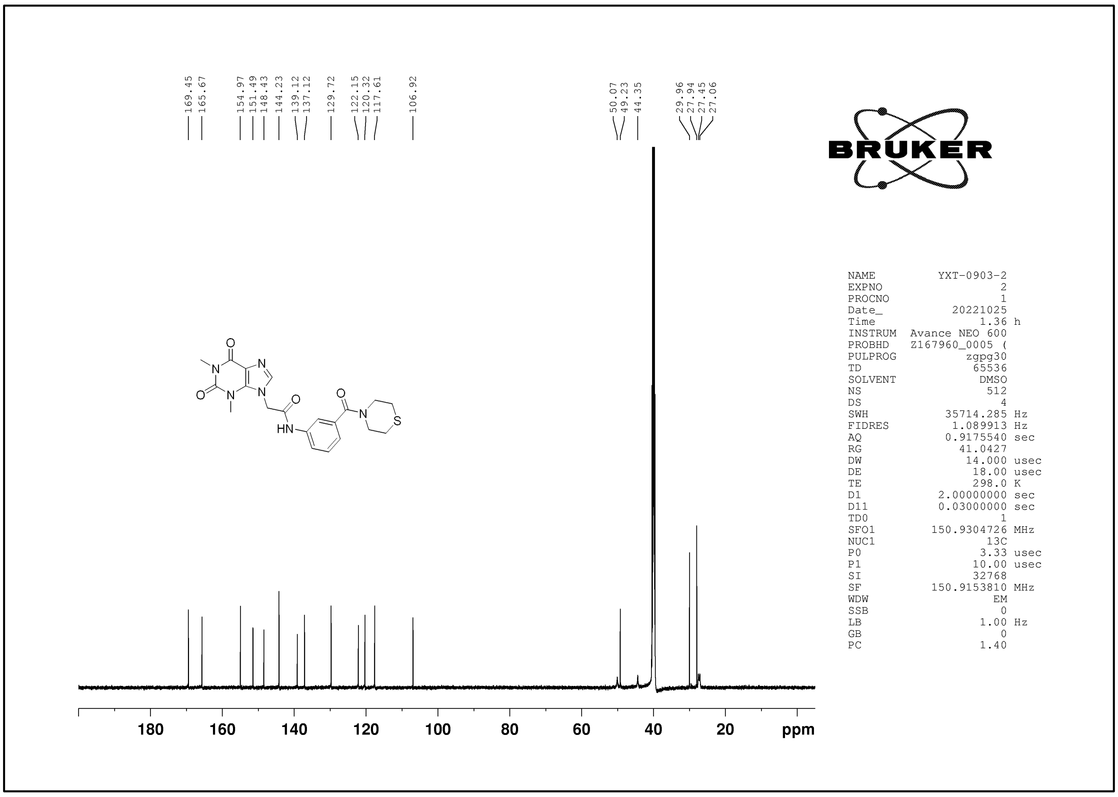

^{13}C-NMR spectrum of compound 15a

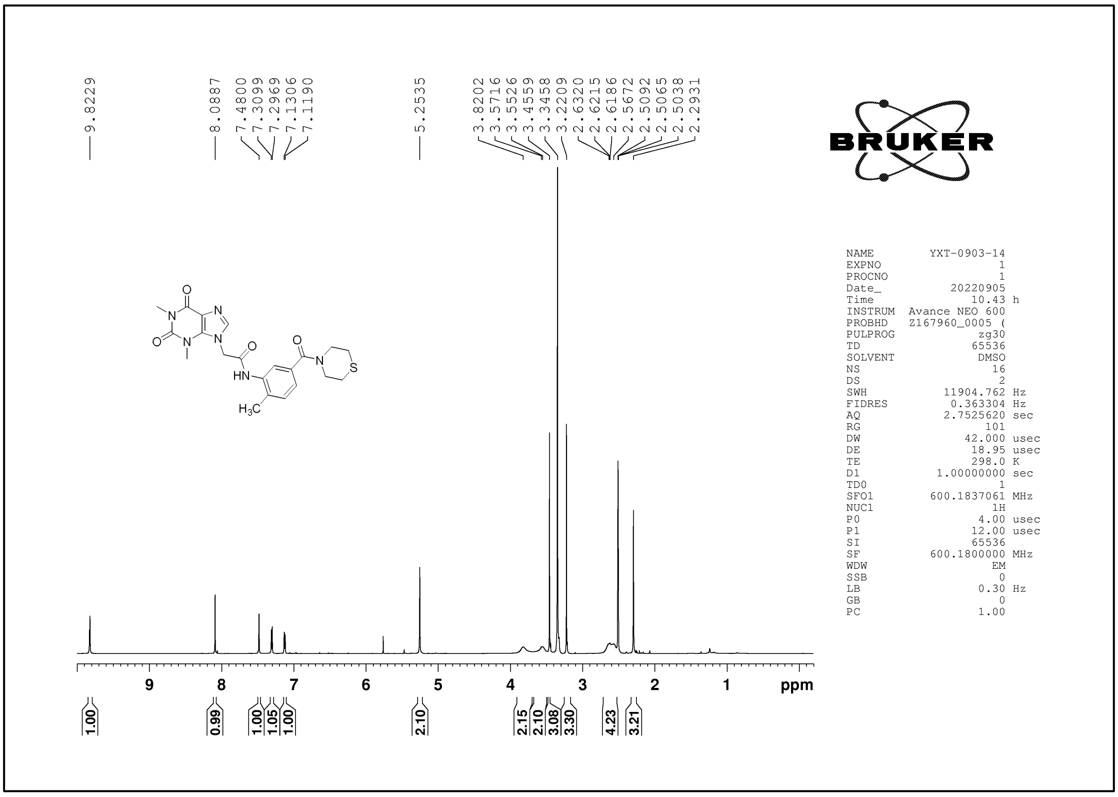

^{1}H-NMR spectrum of compound 15b

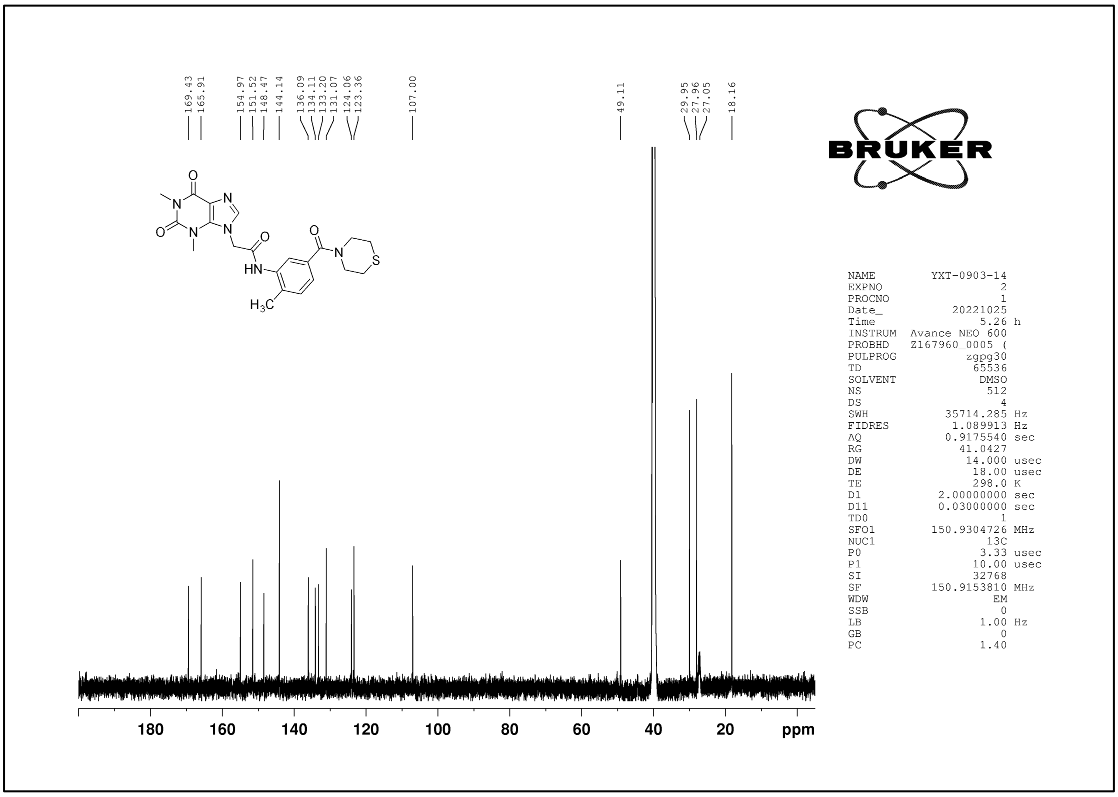

^{13}C-NMR spectrum of compound 15b

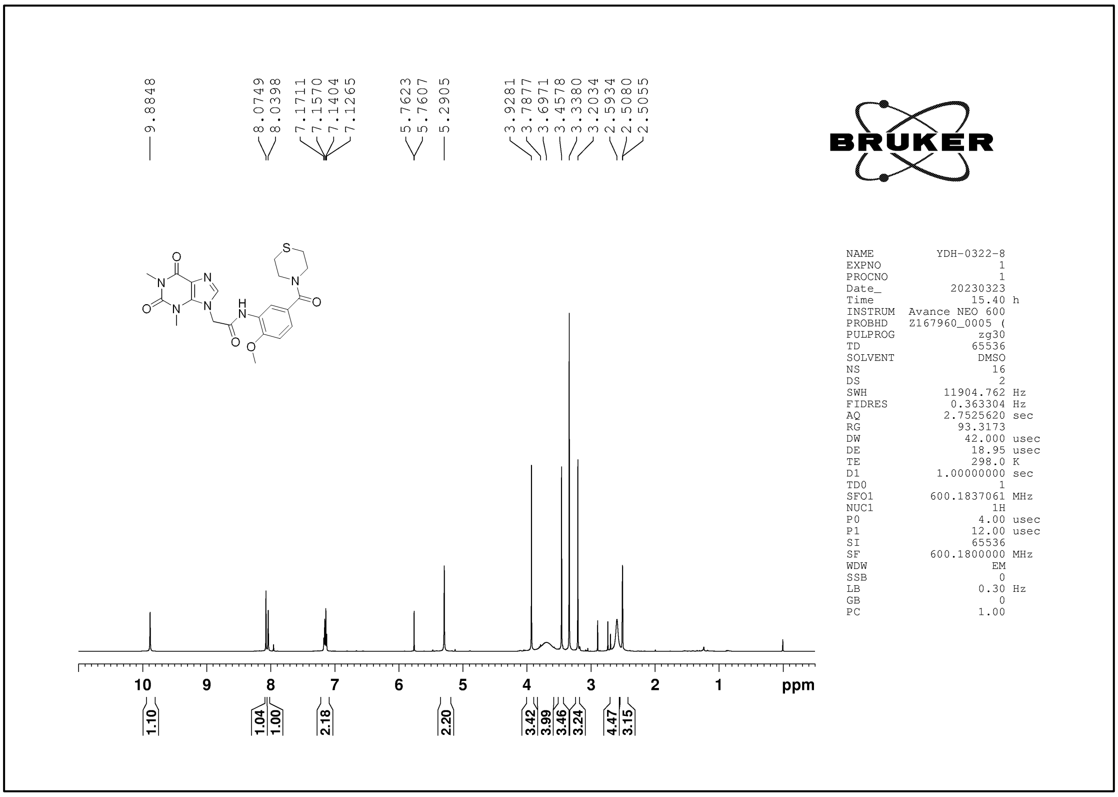

^{1}H-NMR spectrum of compound 15c

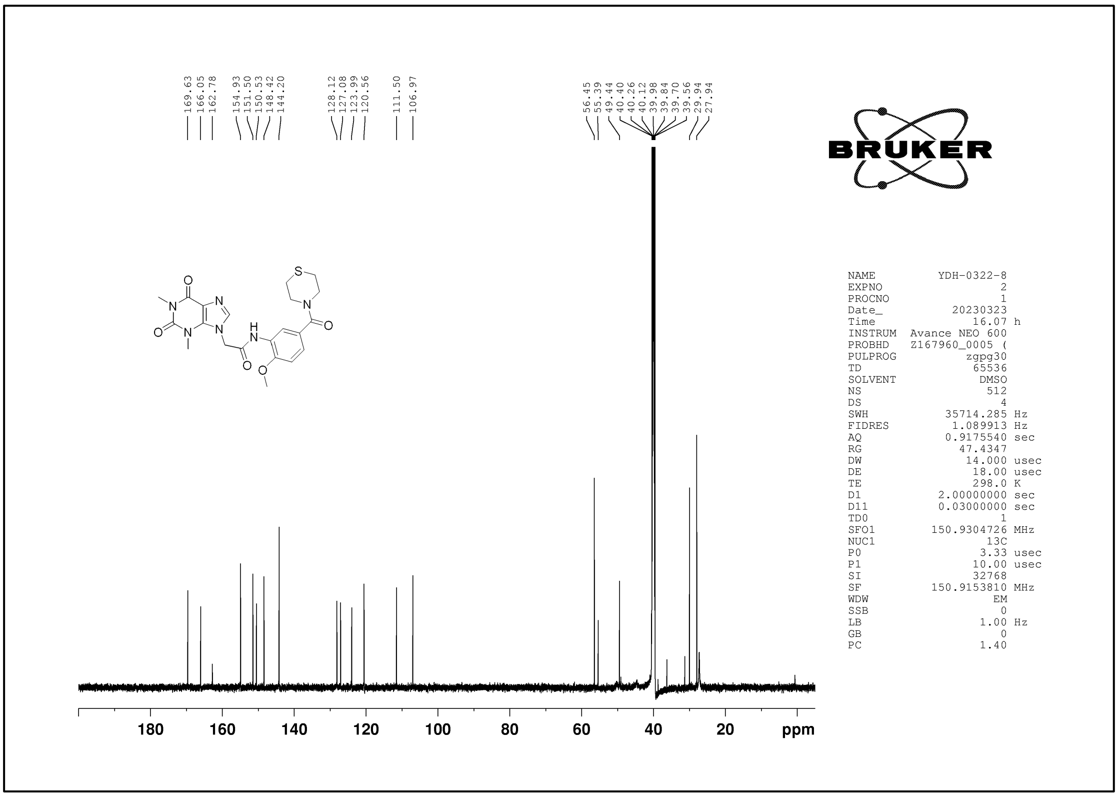

^{13}C-NMR spectrum of compound 15c

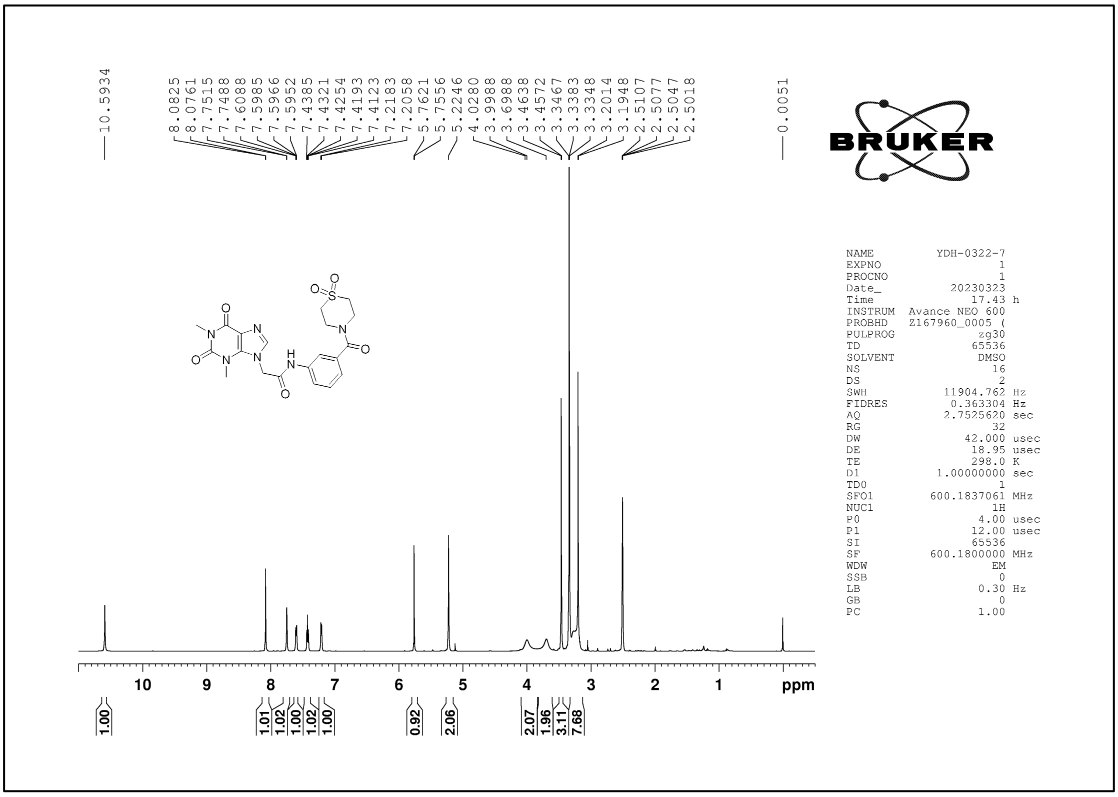

^{1}H-NMR spectrum of compound 15d

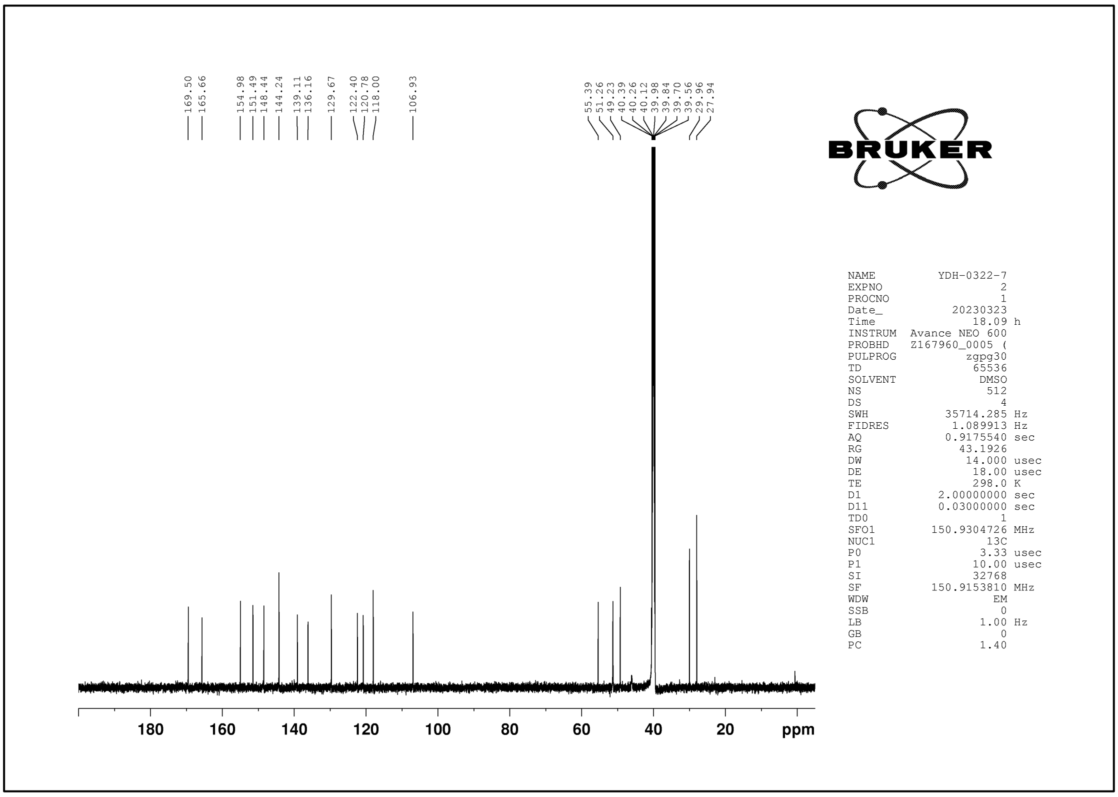

^{13}C-NMR spectrum of compound 15d

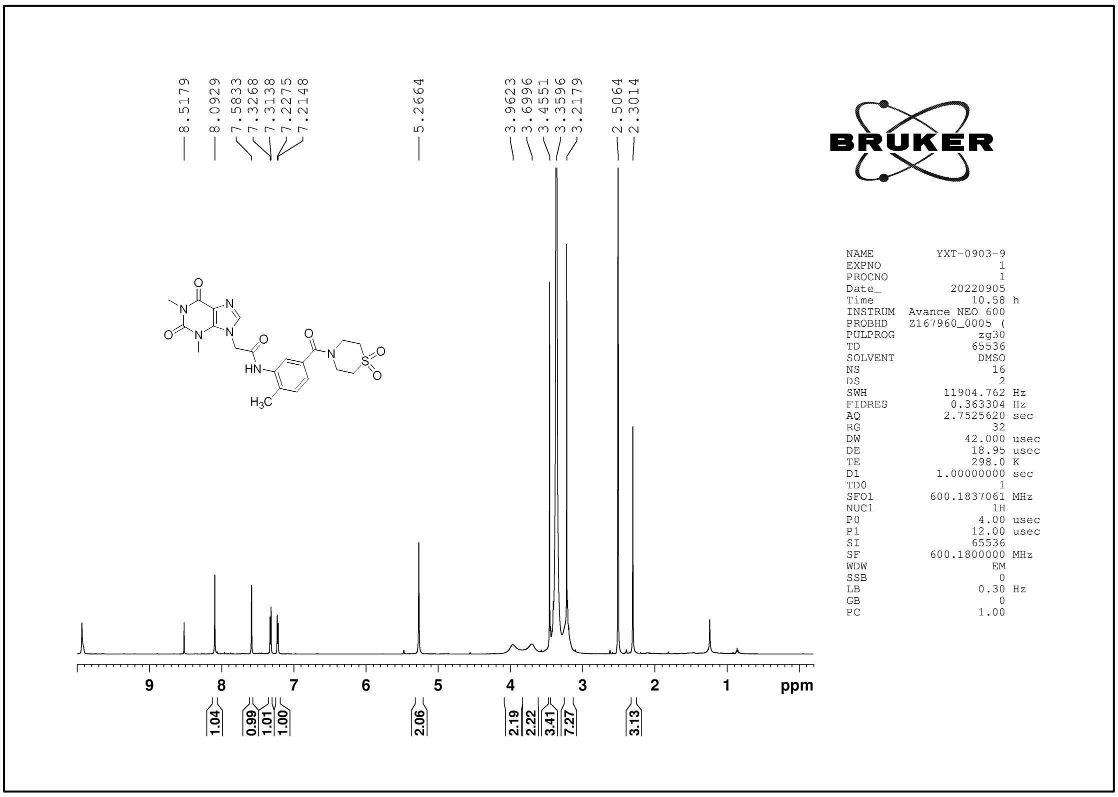

^{1}H-NMR spectrum of compound 15e

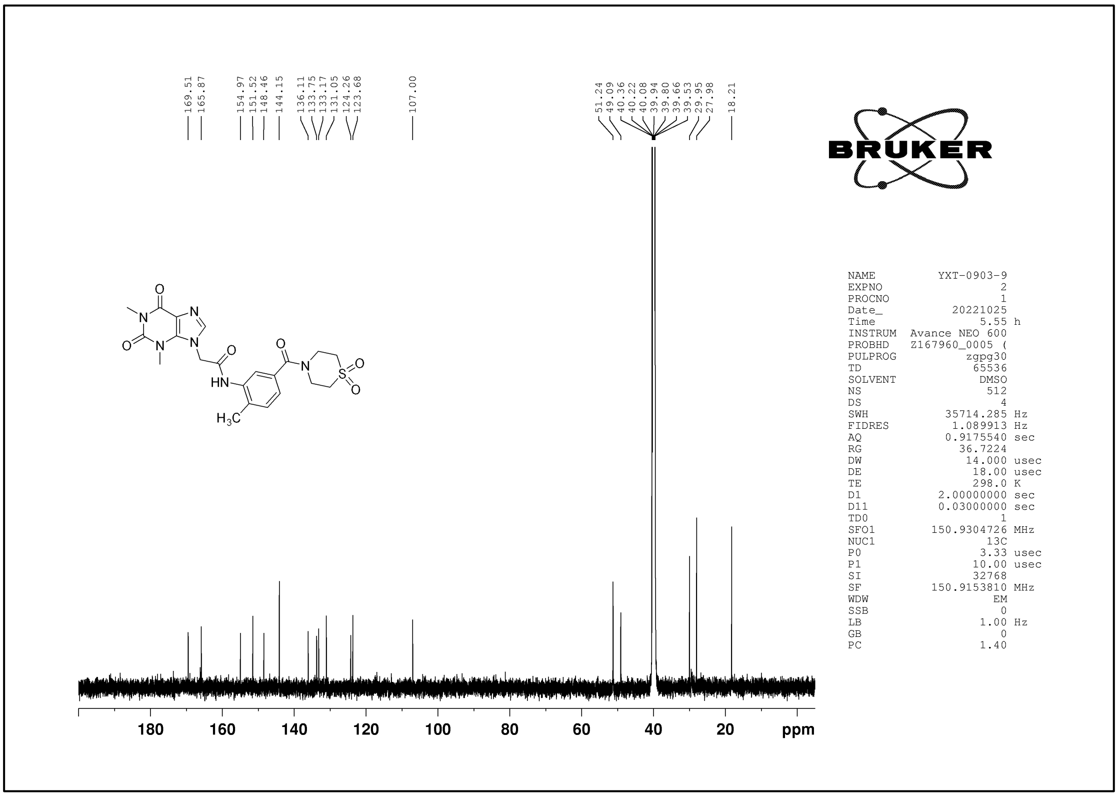

^{13}C-NMR spectrum of compound 15e

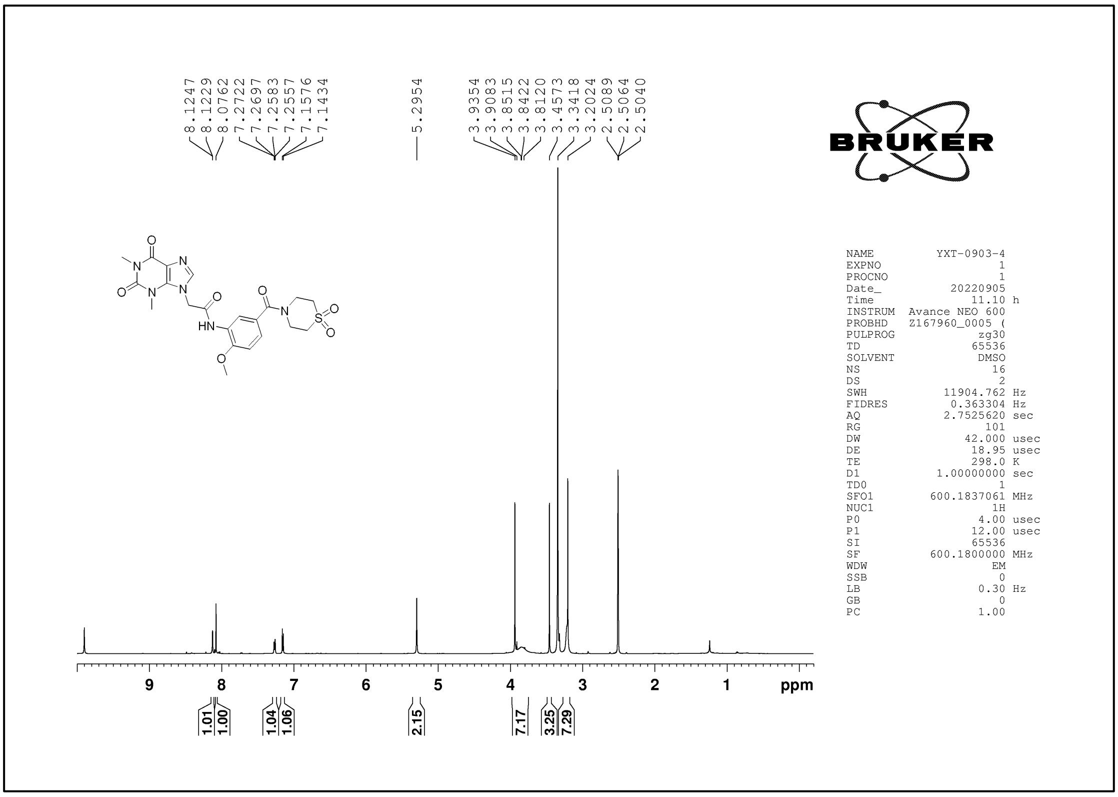

^{1}H-NMR spectrum of compound 15f

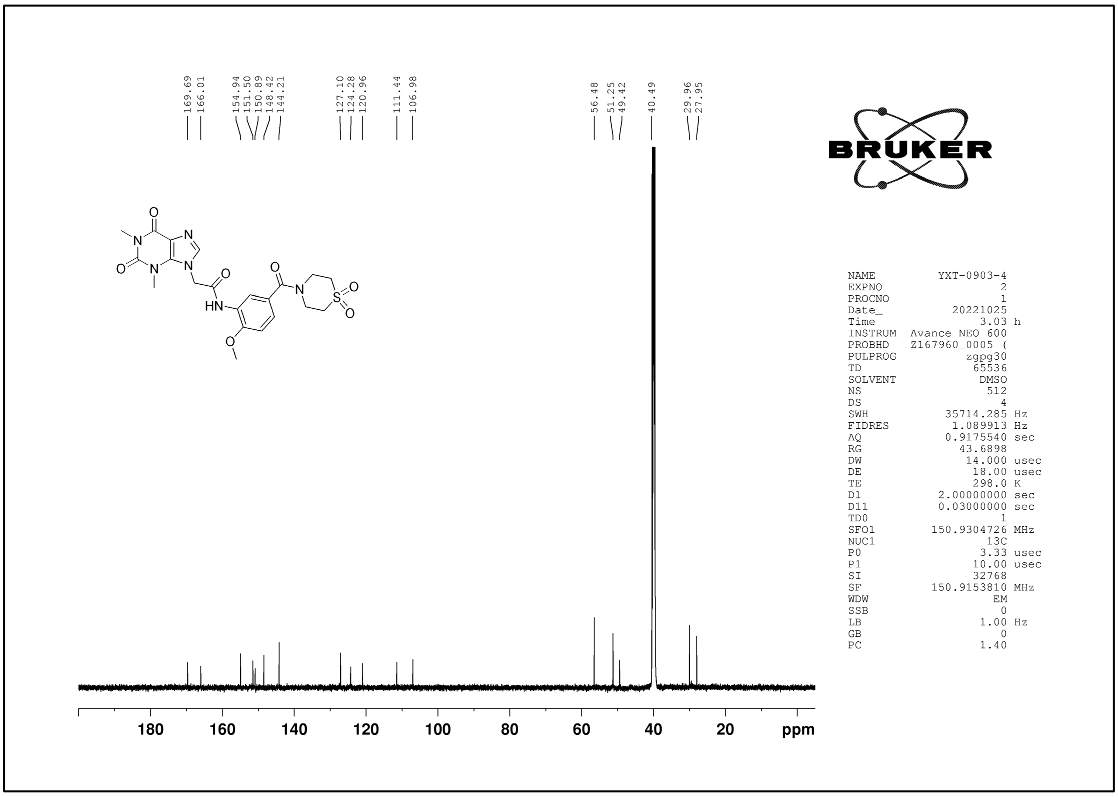

^{13}C-NMR spectrum of compound 15f

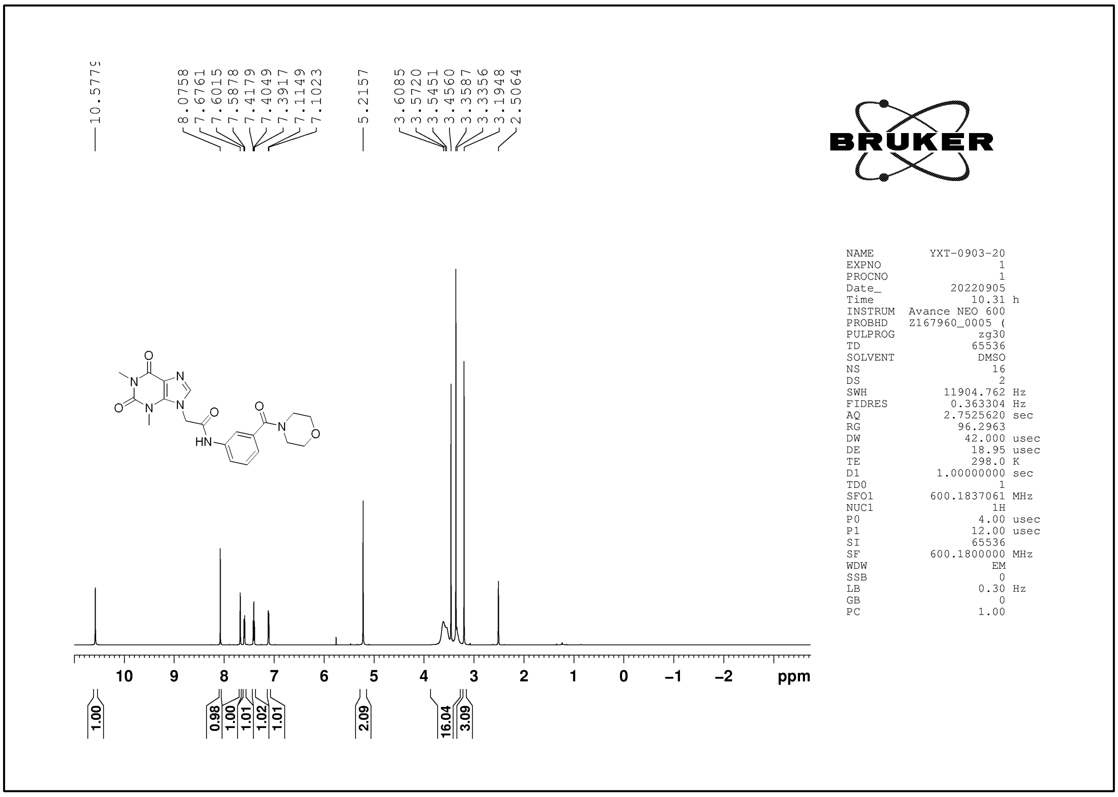

^{1}H-NMR spectrum of compound 15g

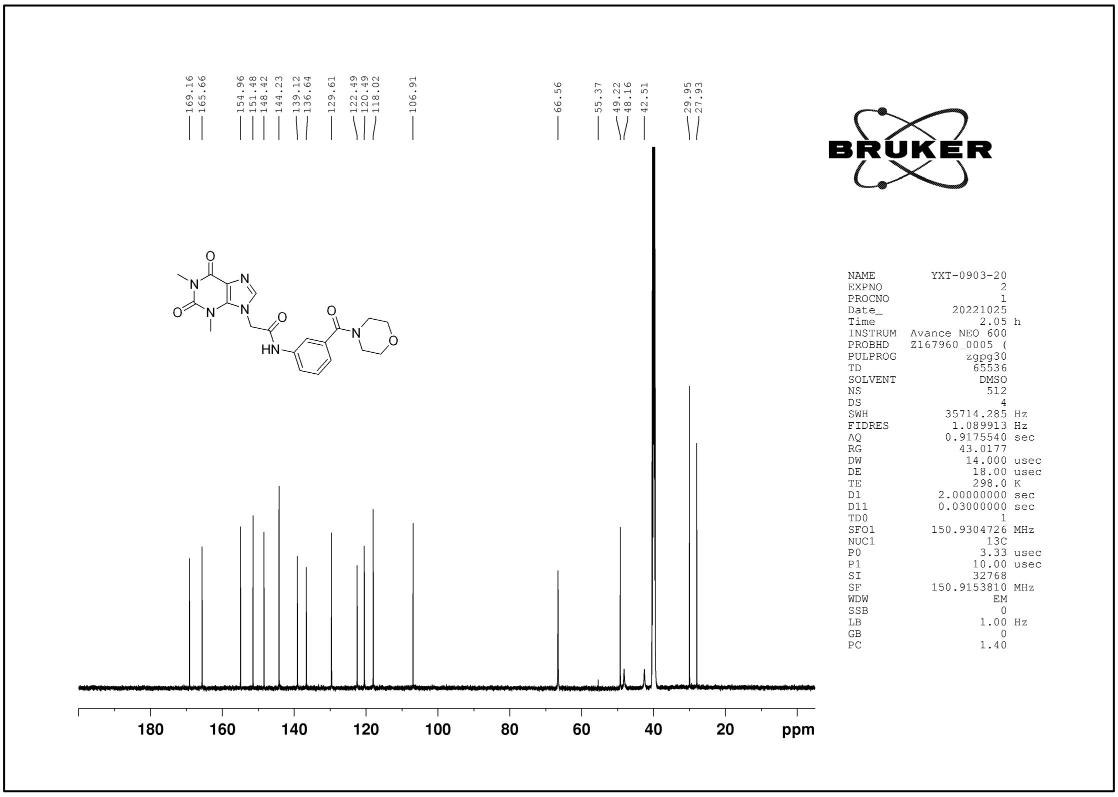

^{13}C-NMR spectrum of compound 15g

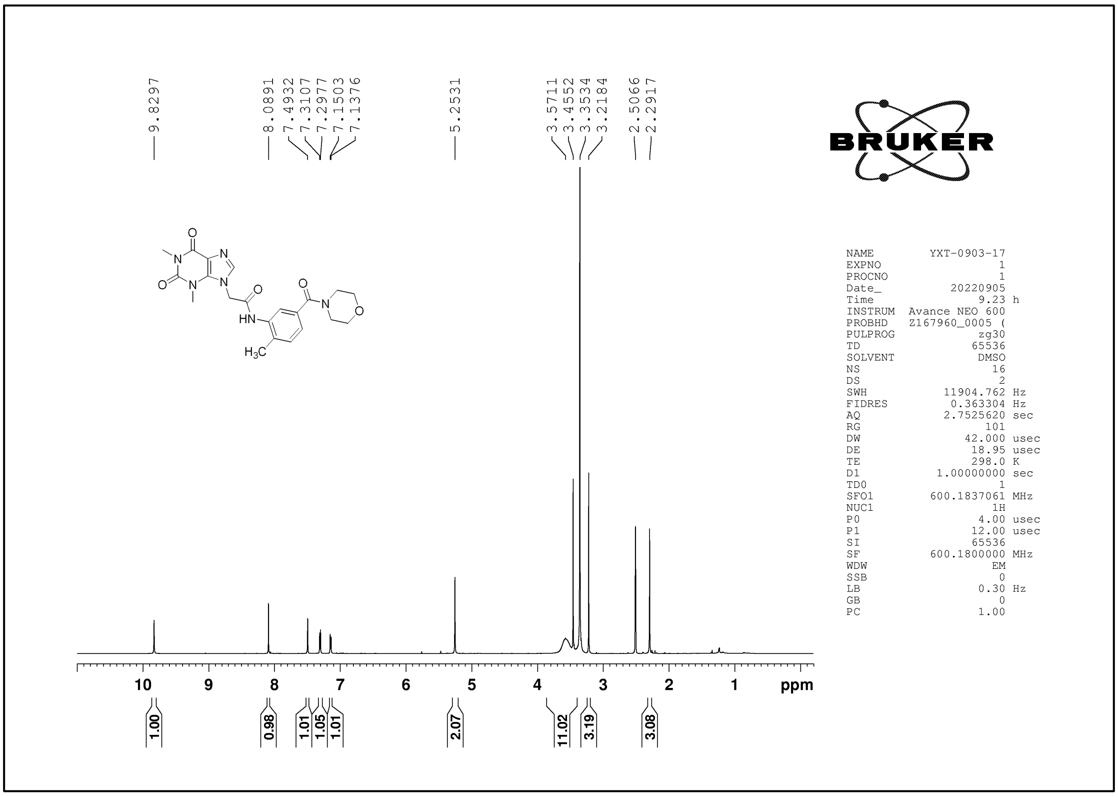

^{1}H-NMR spectrum of compound 15h

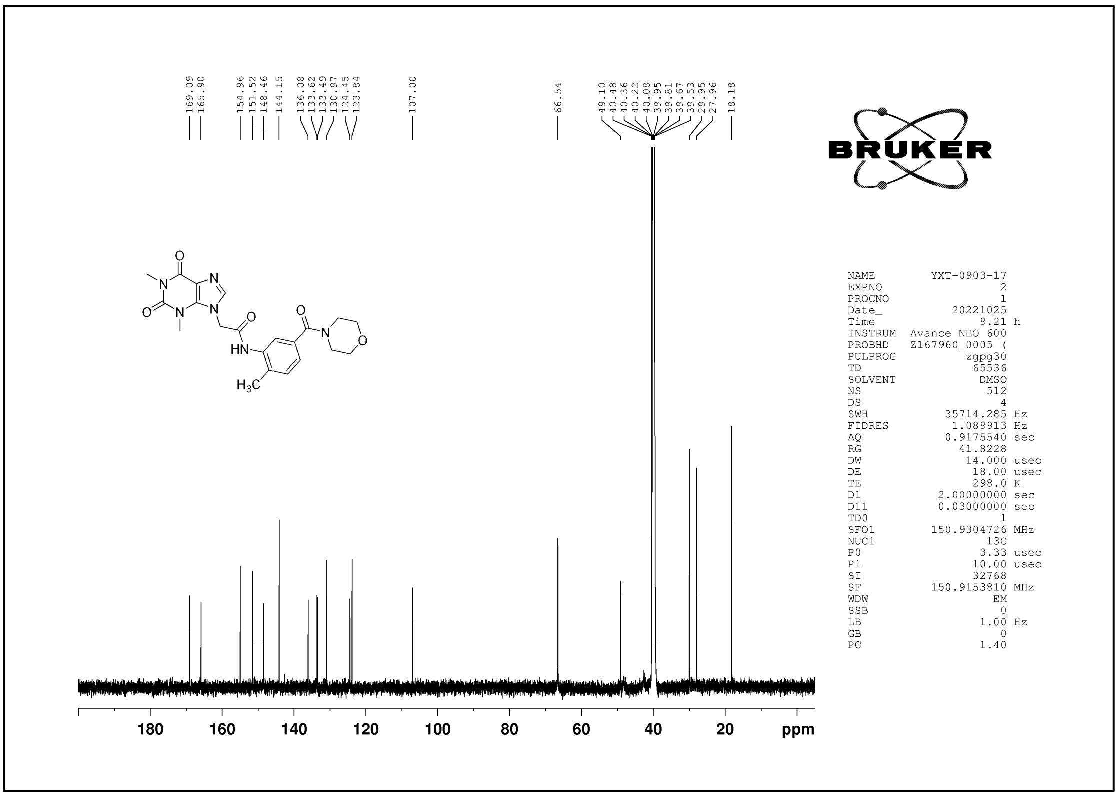

^{13}C-NMR spectrum of compound 15h

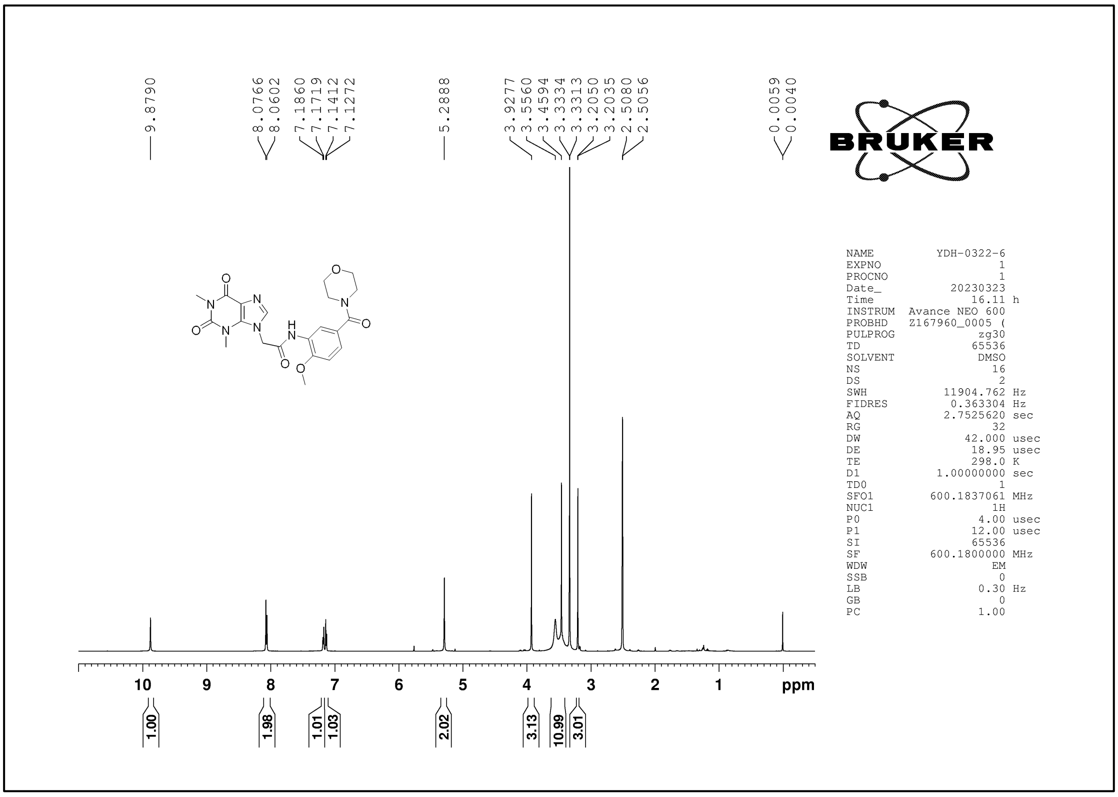

^{1}H-NMR spectrum of compound 15i

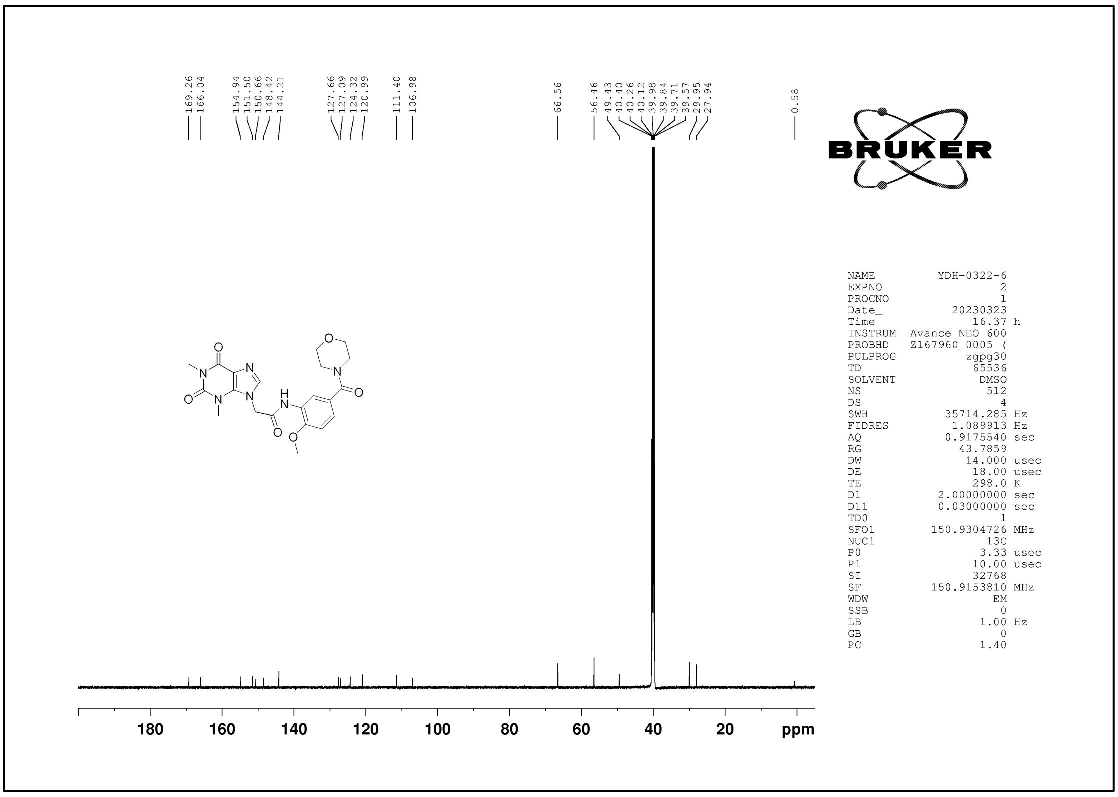

^{13}C-NMR spectrum of compound 15i

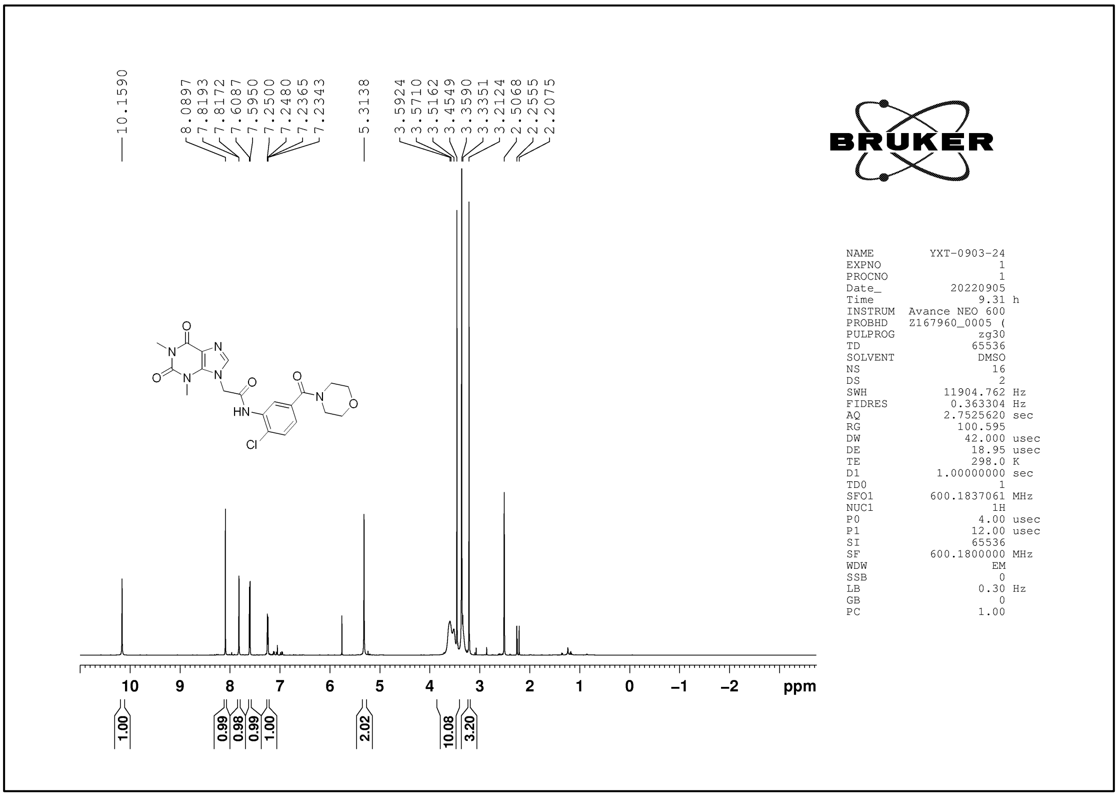

^{1}H-NMR spectrum of compound 15j

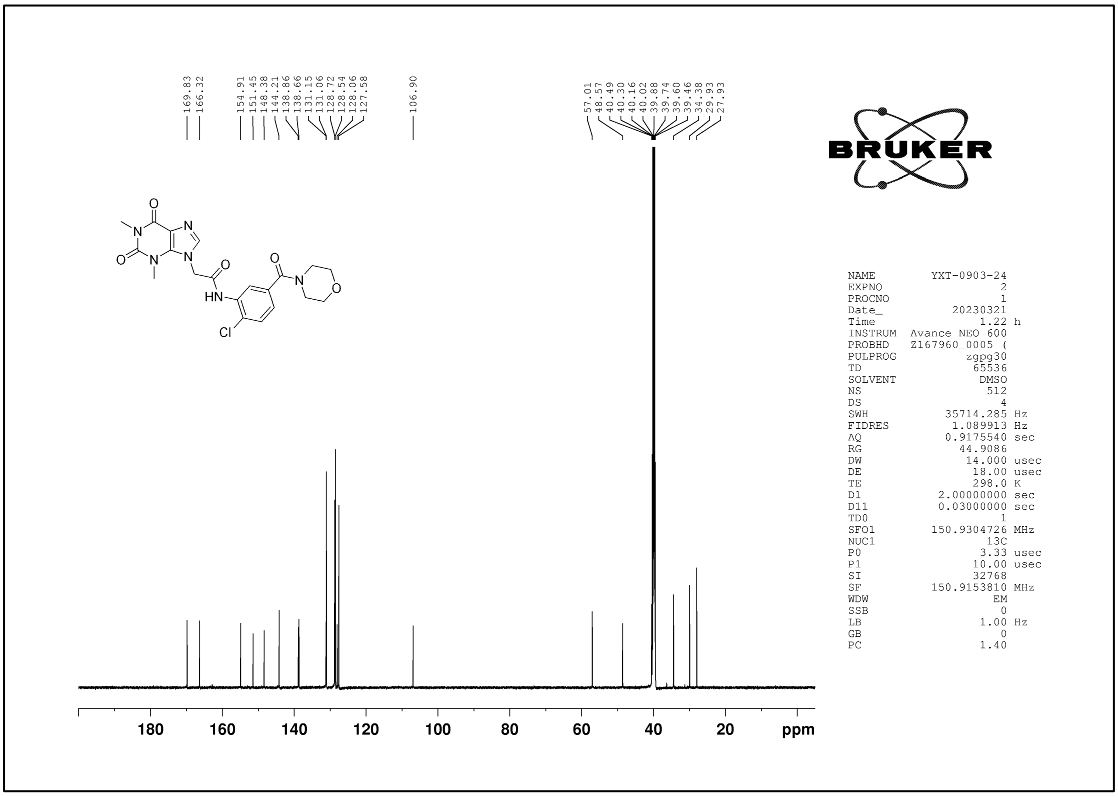

^{13}C-NMR spectrum of compound 15j

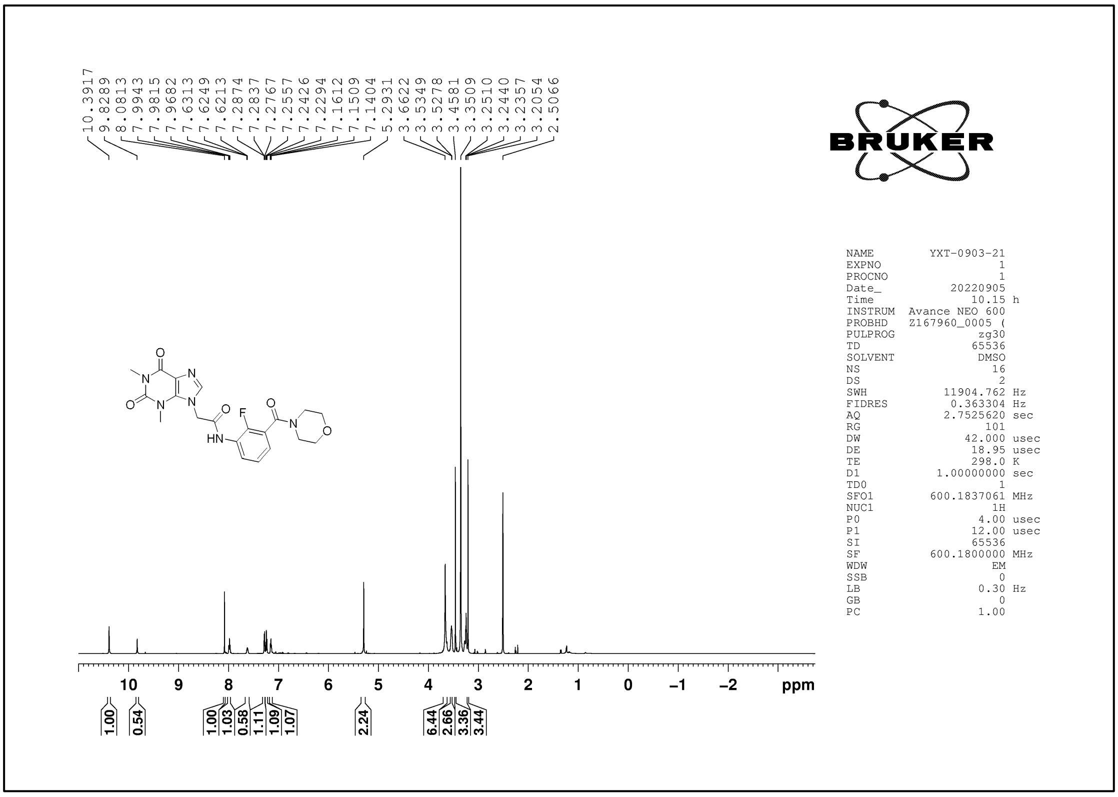

^{1}H-NMR spectrum of compound 15k

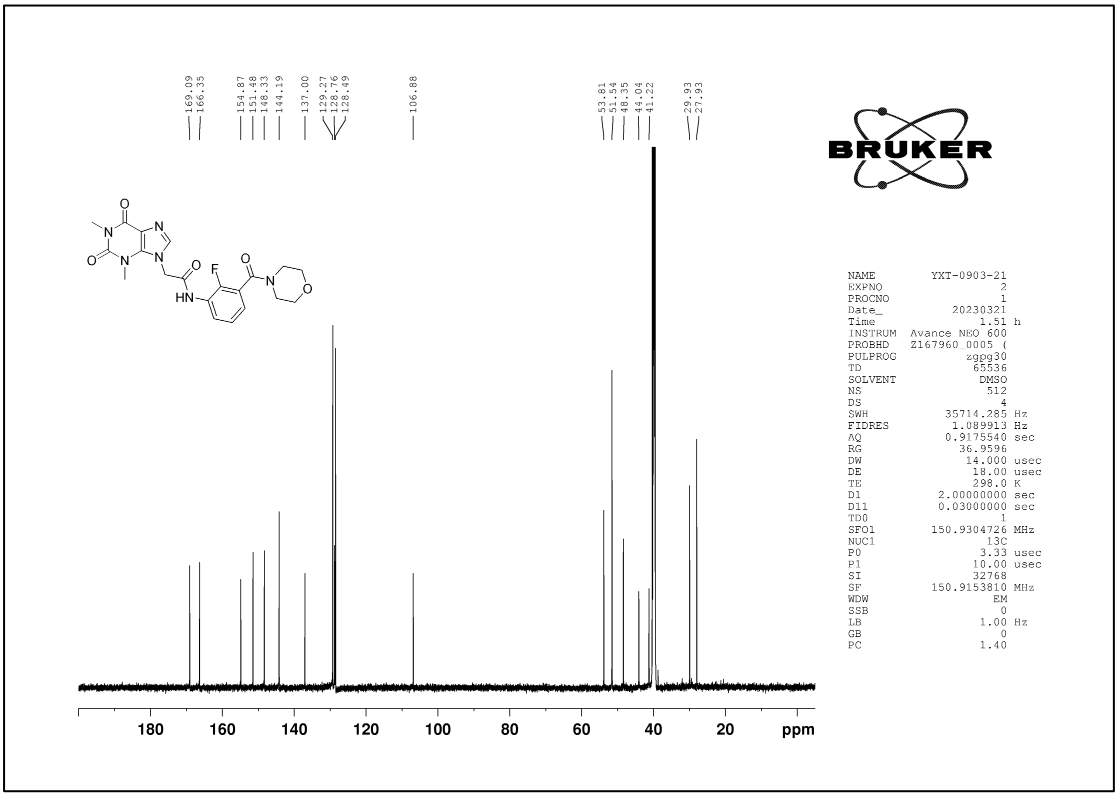

^{13}C-NMR spectrum of compound 15k

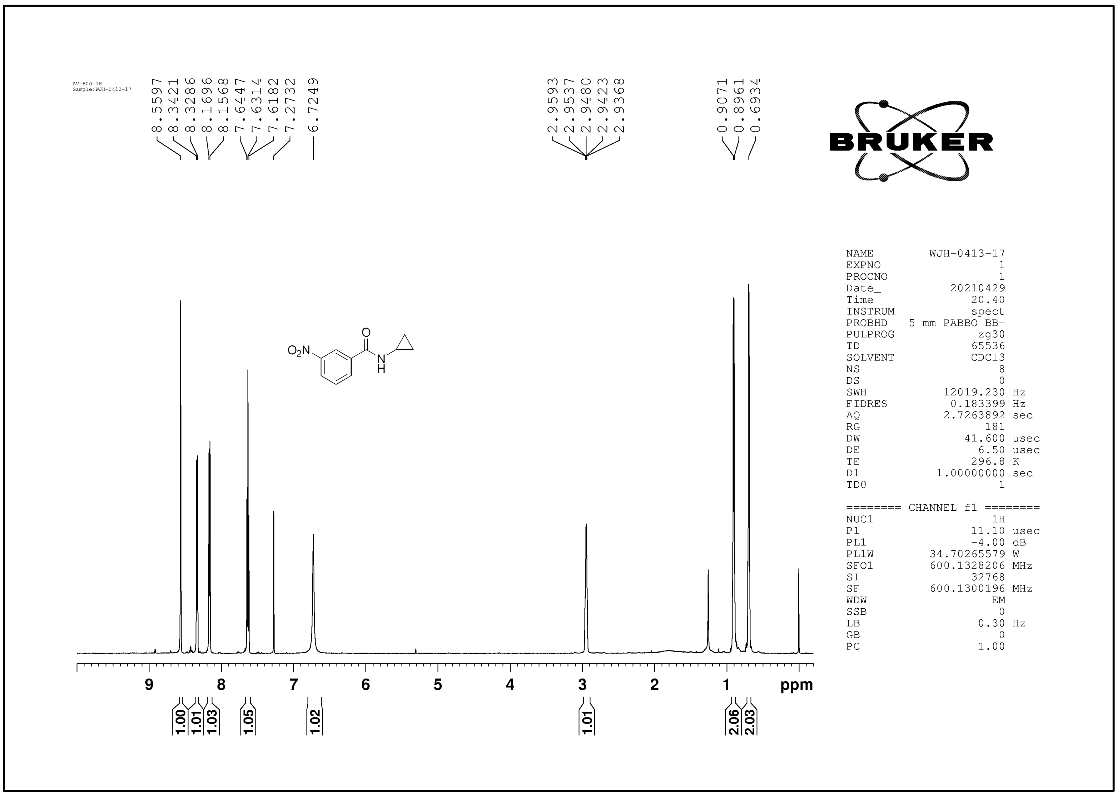

^{1}H-NMR spectrum of compound 17a

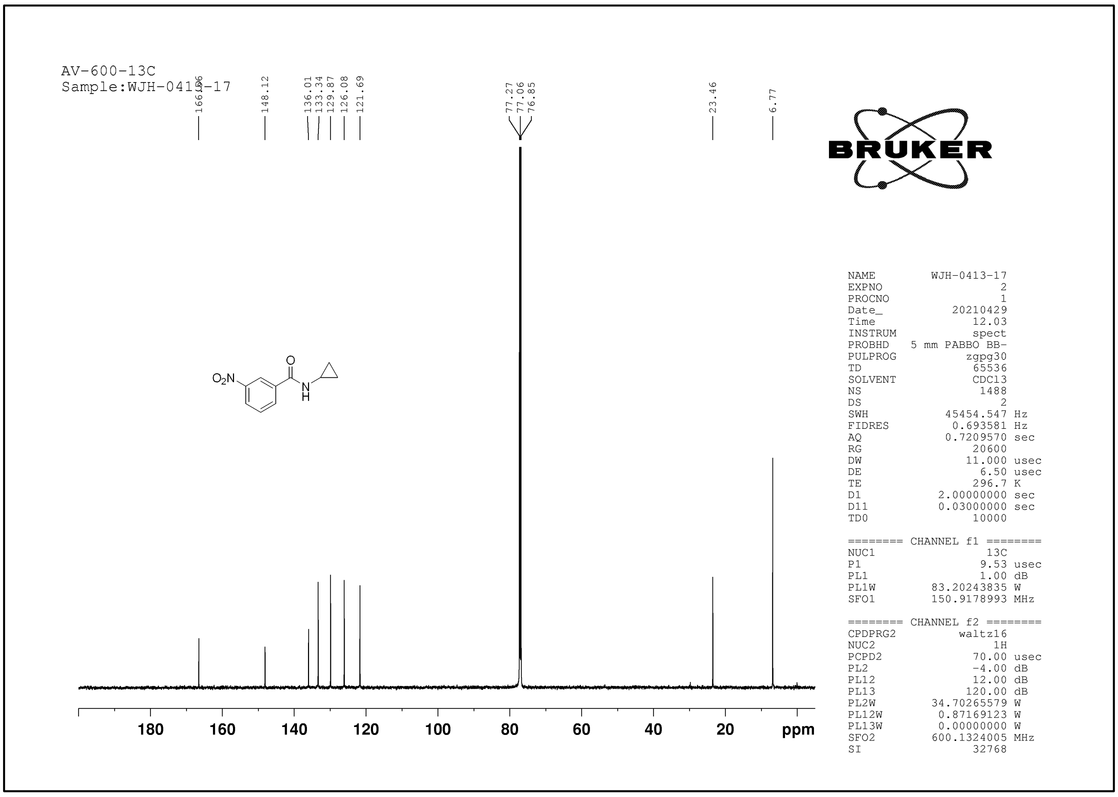

^{13}C-NMR spectrum of compound 17a

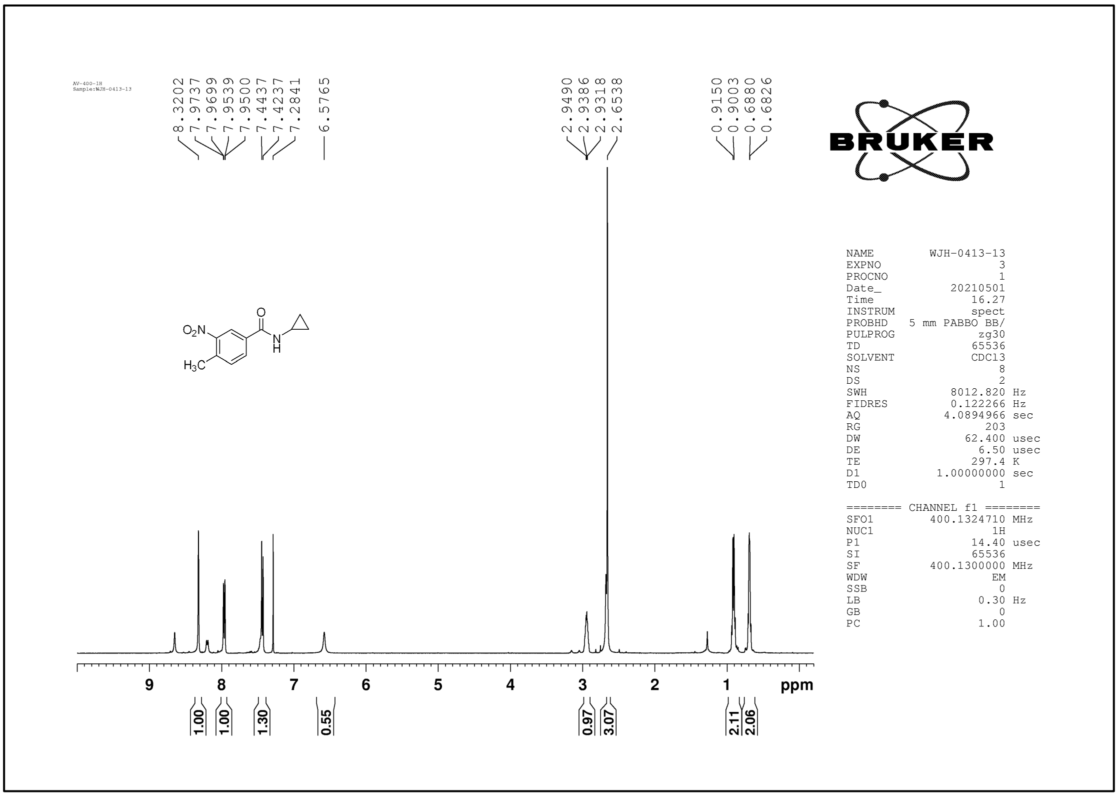

^{1}H-NMR spectrum of compound 17b

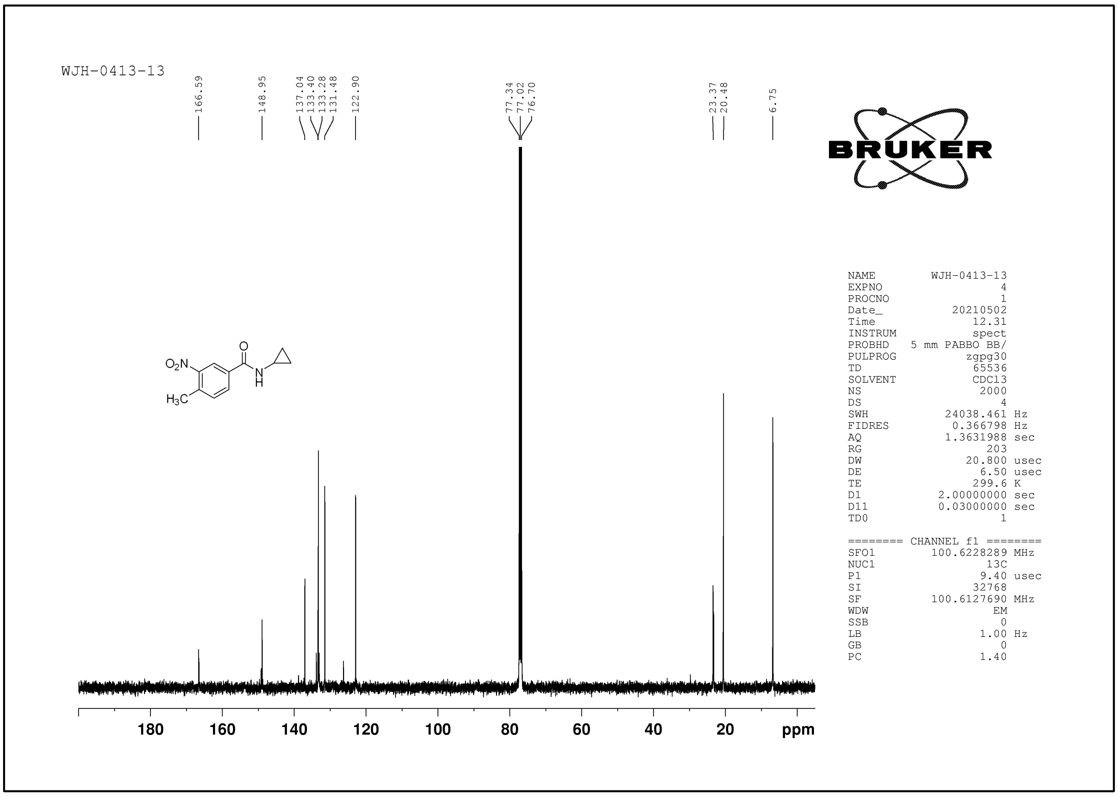

^{13}C-NMR spectrum of compound 17b

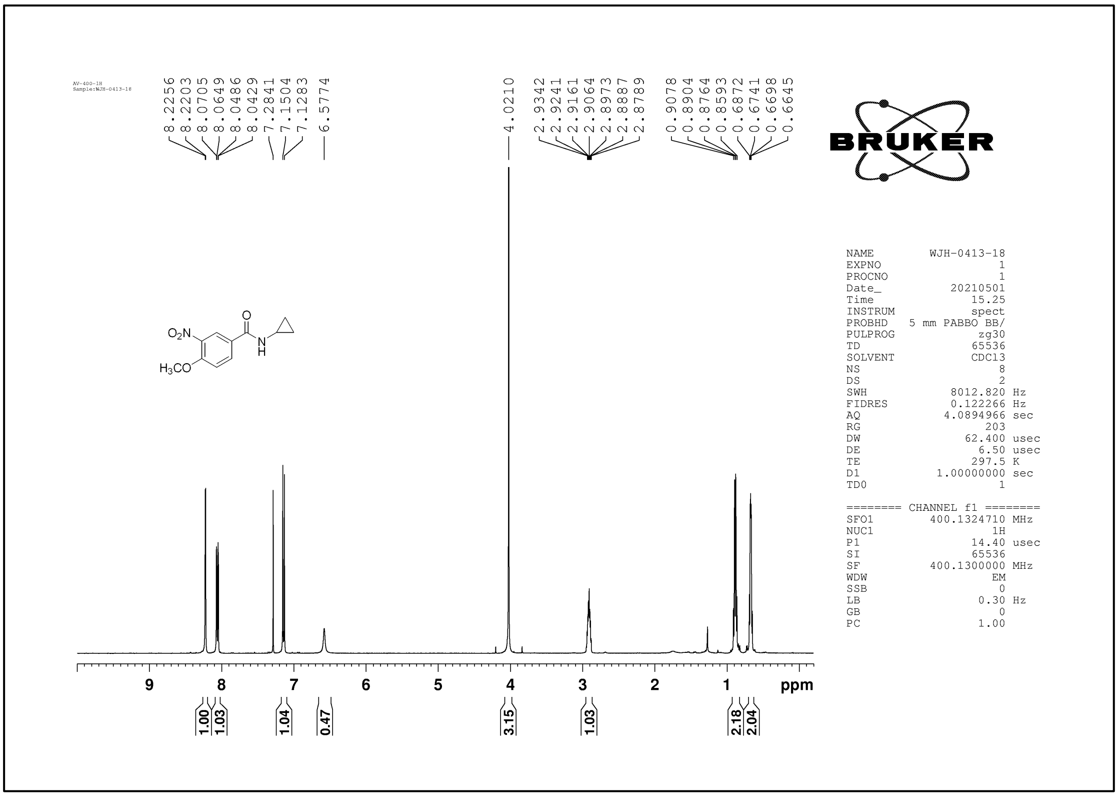

^{1}H-NMR spectrum of compound 17c

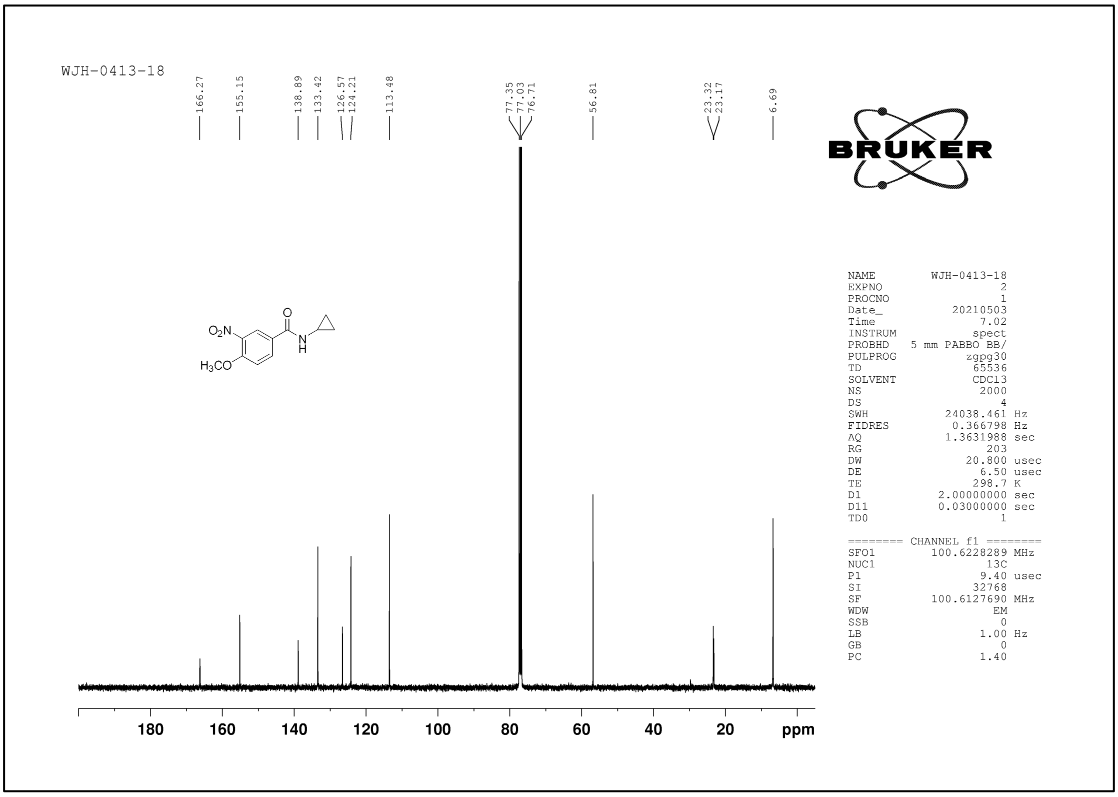

^{13}C-NMR spectrum of compound 17c

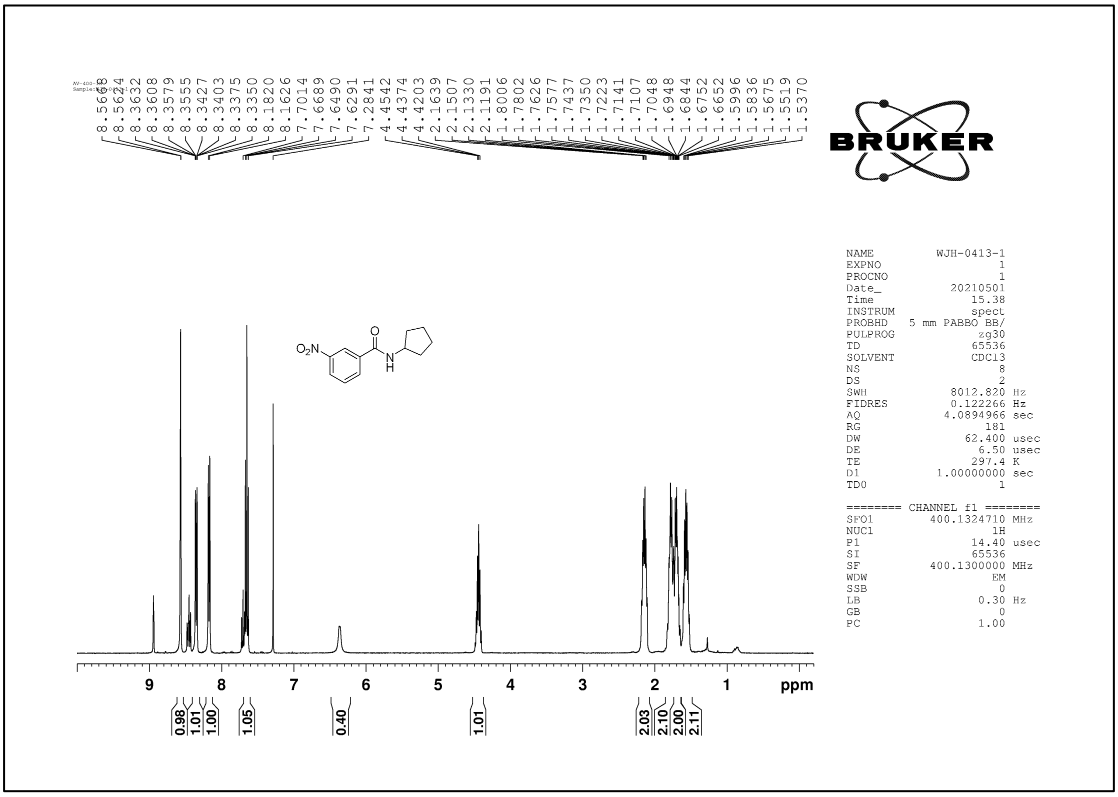

^{1}H-NMR spectrum of compound 17d

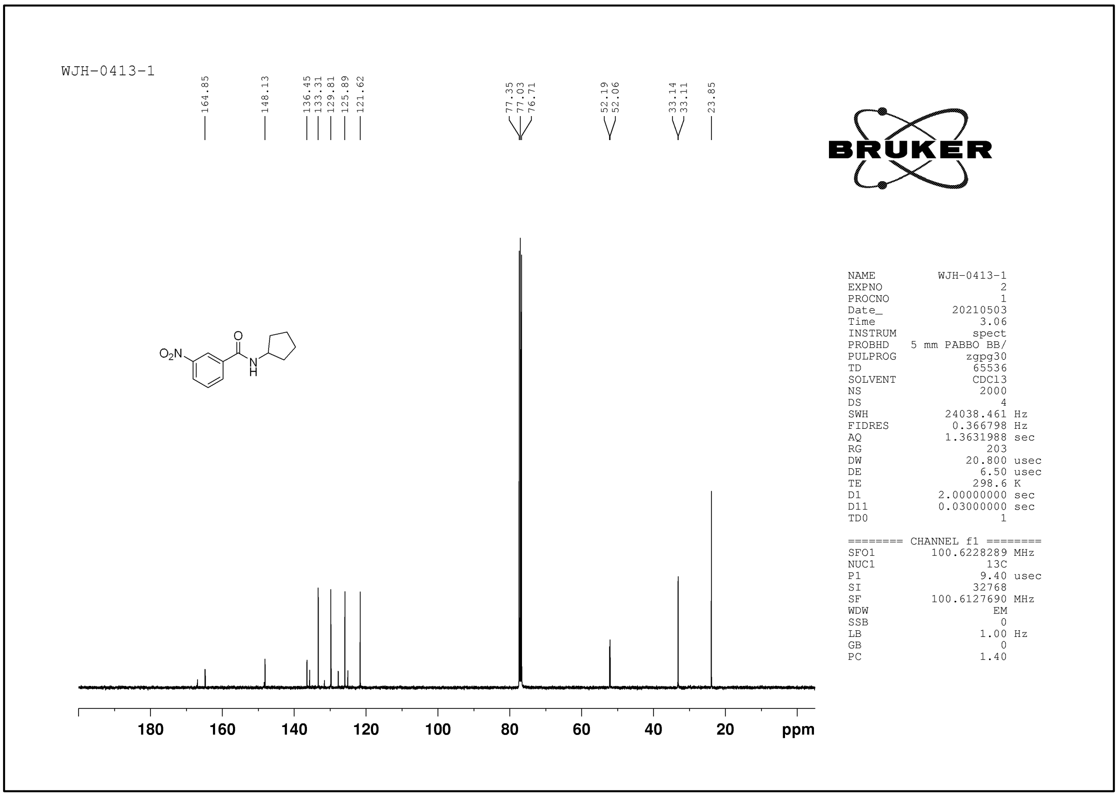

^{13}C-NMR spectrum of compound 17d

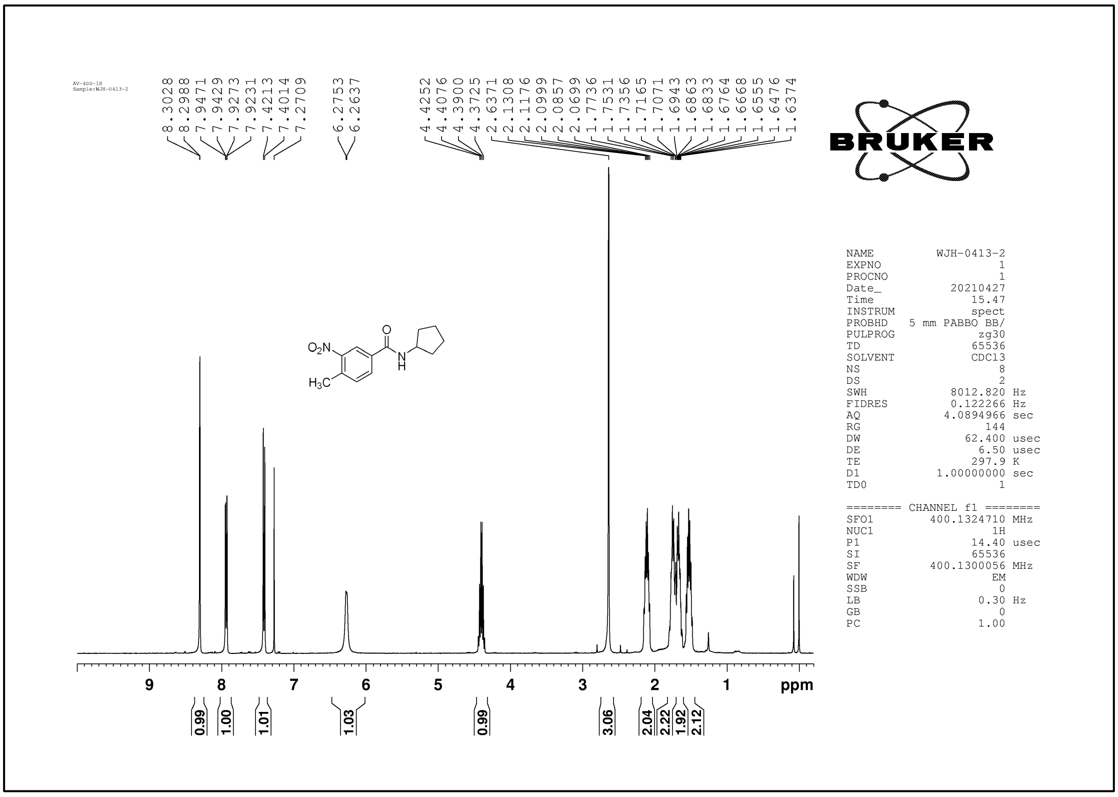

^{1}H-NMR spectrum of compound 17e

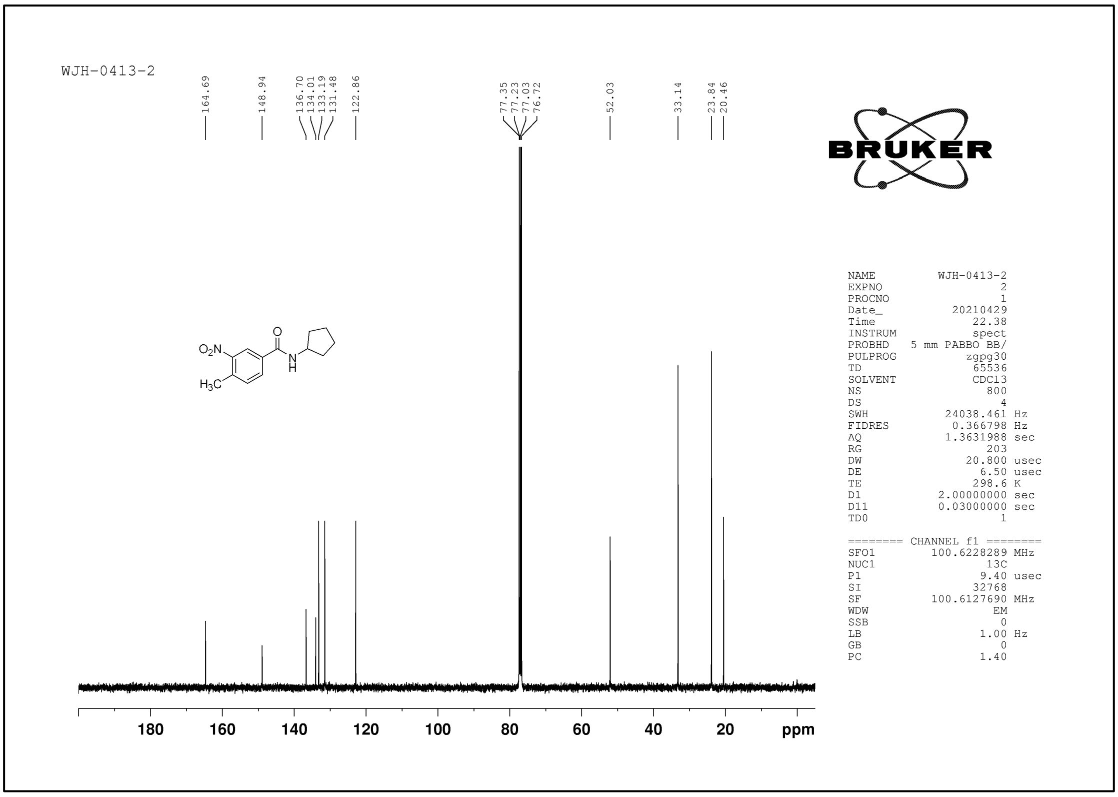

^{13}C-NMR spectrum of compound 17e

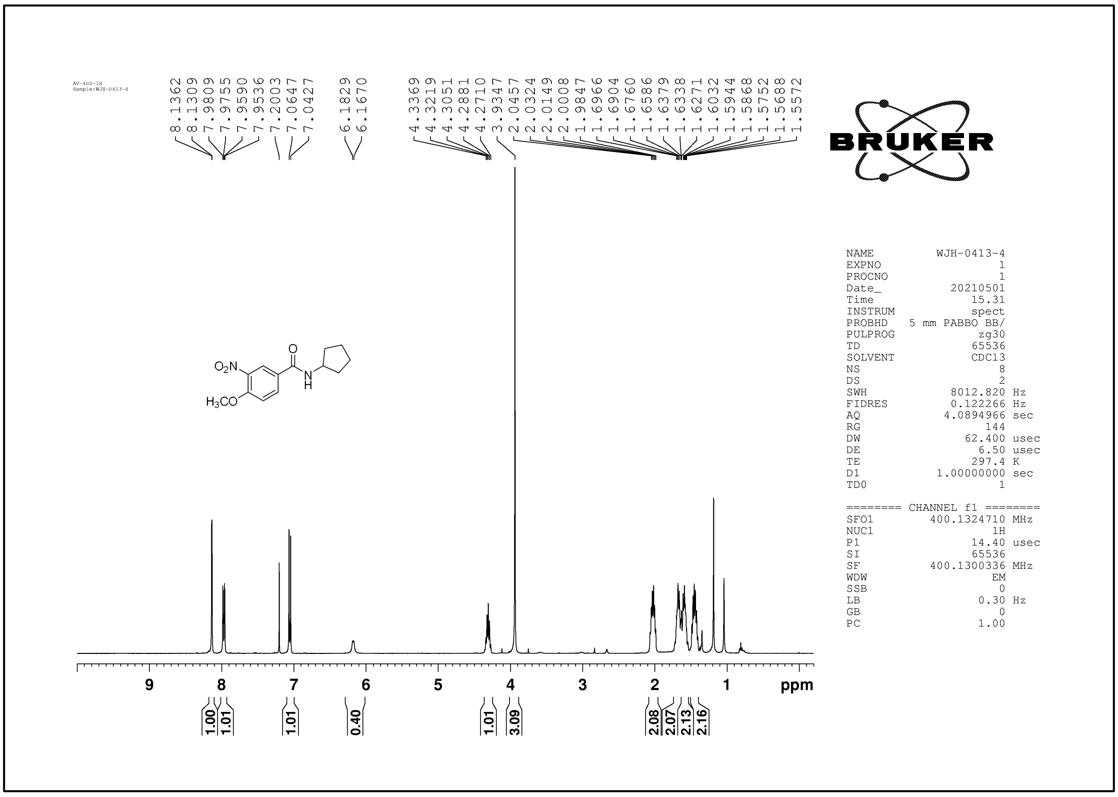

^{1}H-NMR spectrum of compound 17f

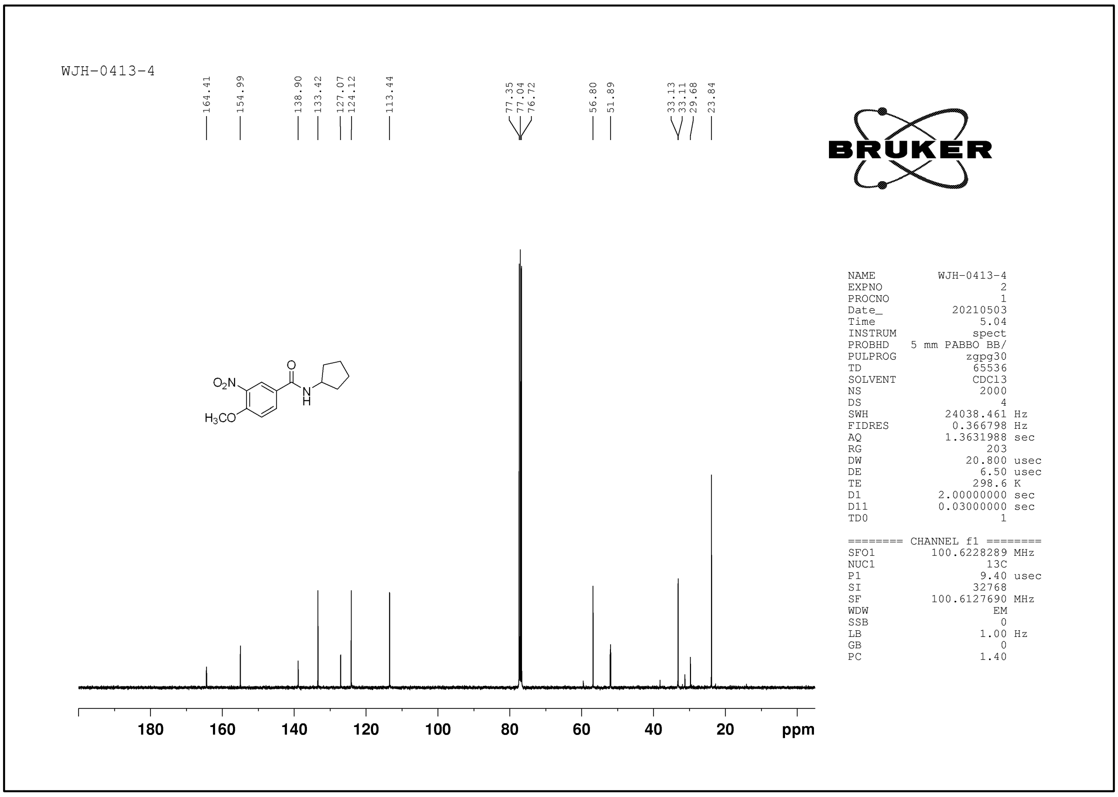

^{13}C-NMR spectrum of compound 17f

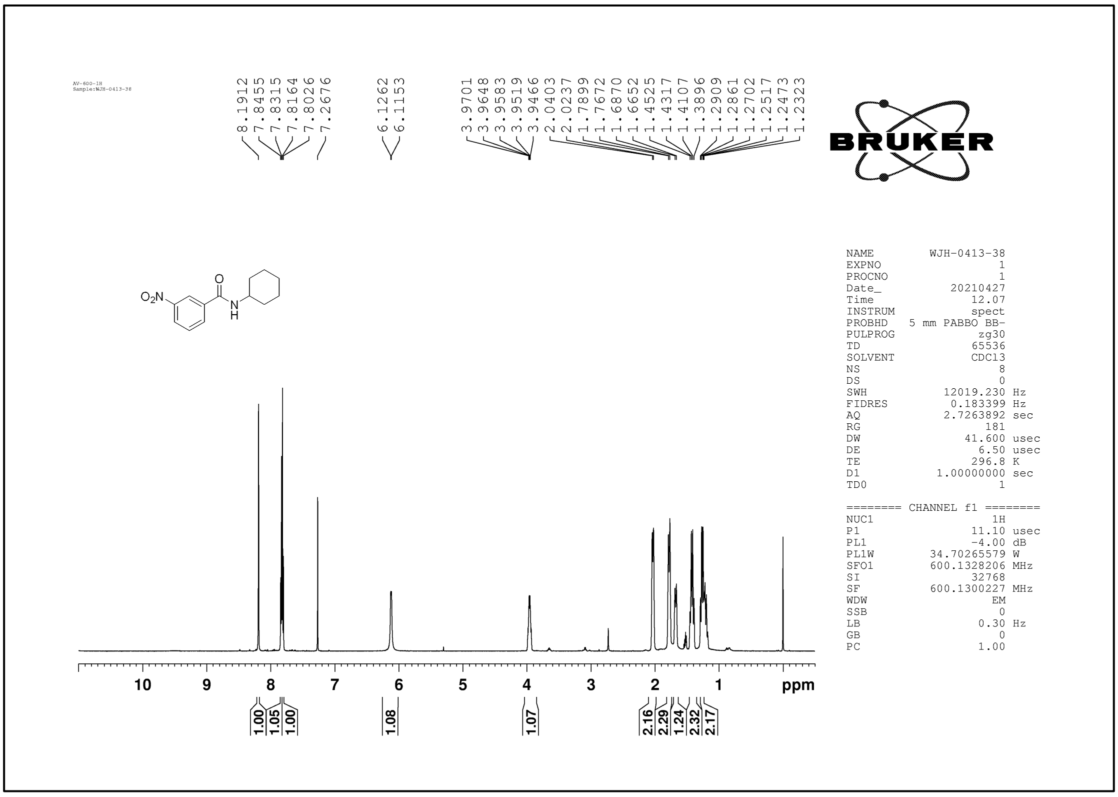

^{1}H-NMR spectrum of compound 17g

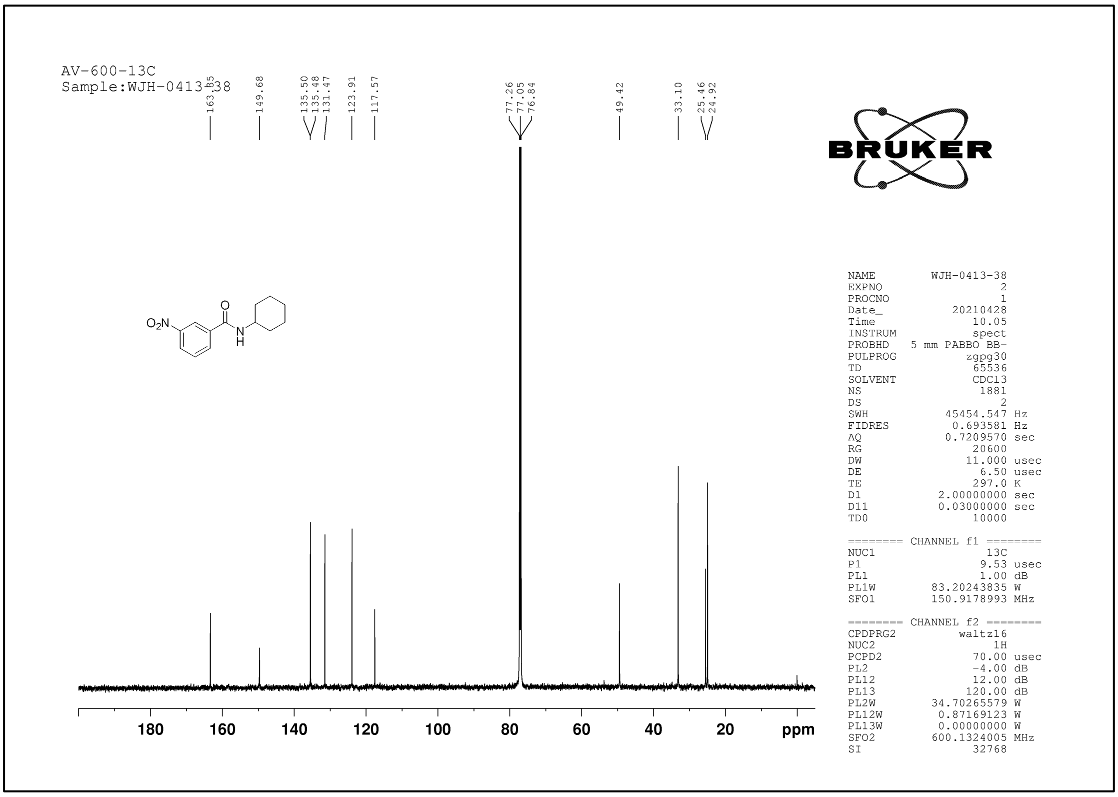

^{13}C-NMR spectrum of compound 17g

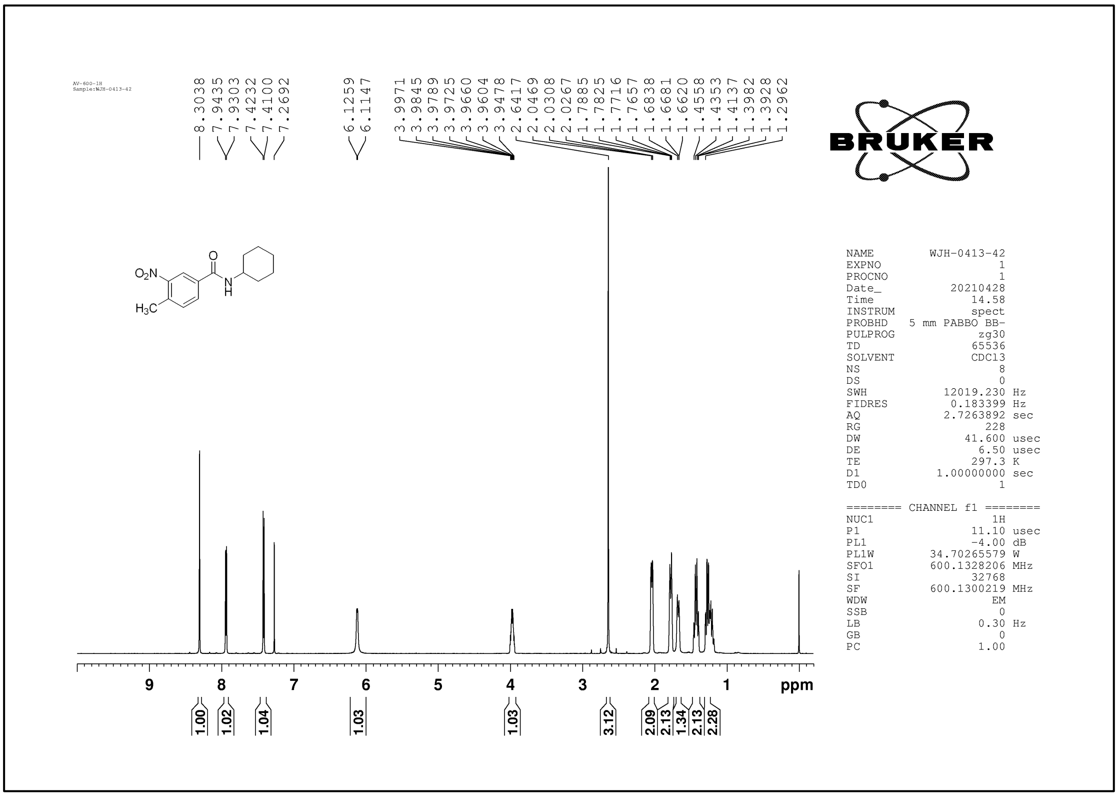

^{1}H-NMR spectrum of compound 17h

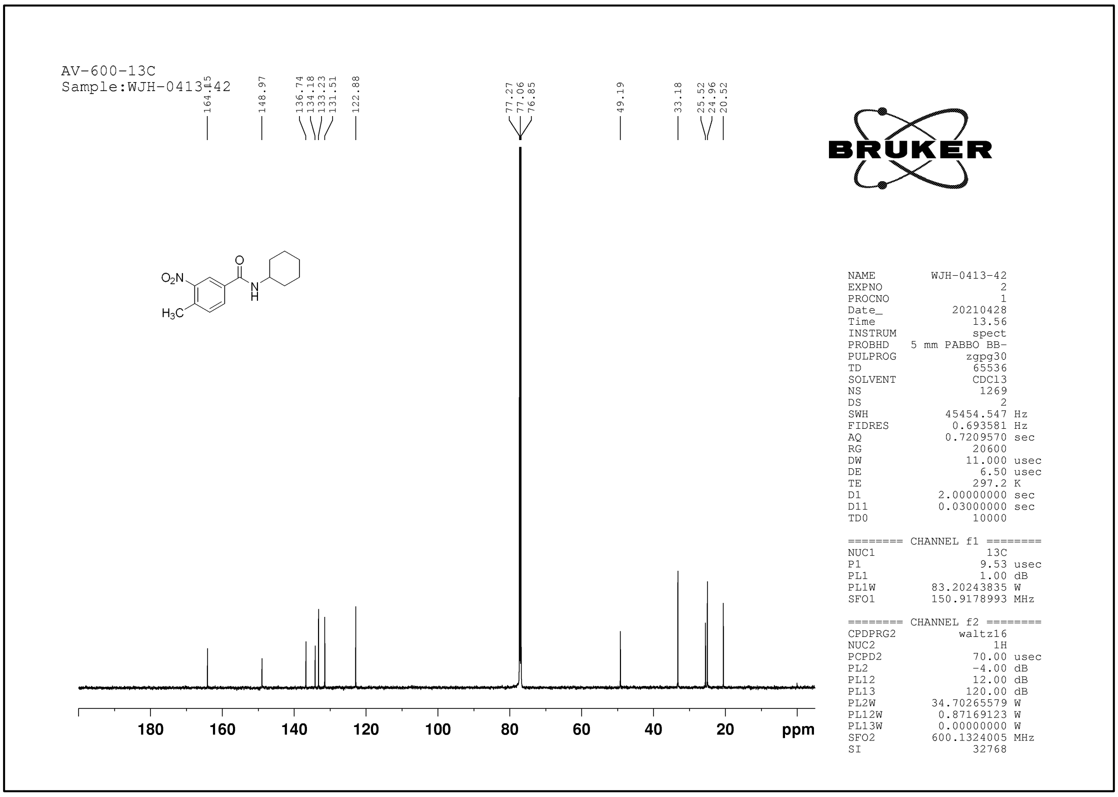

^{13}C-NMR spectrum of compound 17h

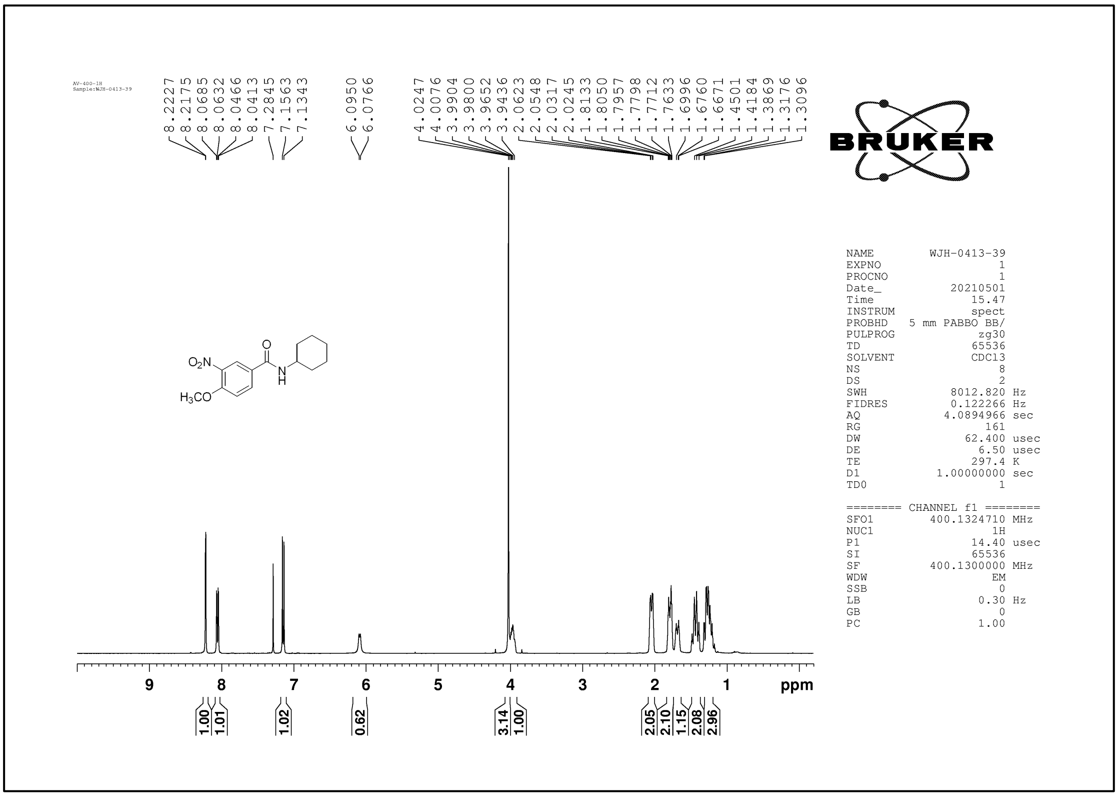

^{1}H-NMR spectrum of compound 17i

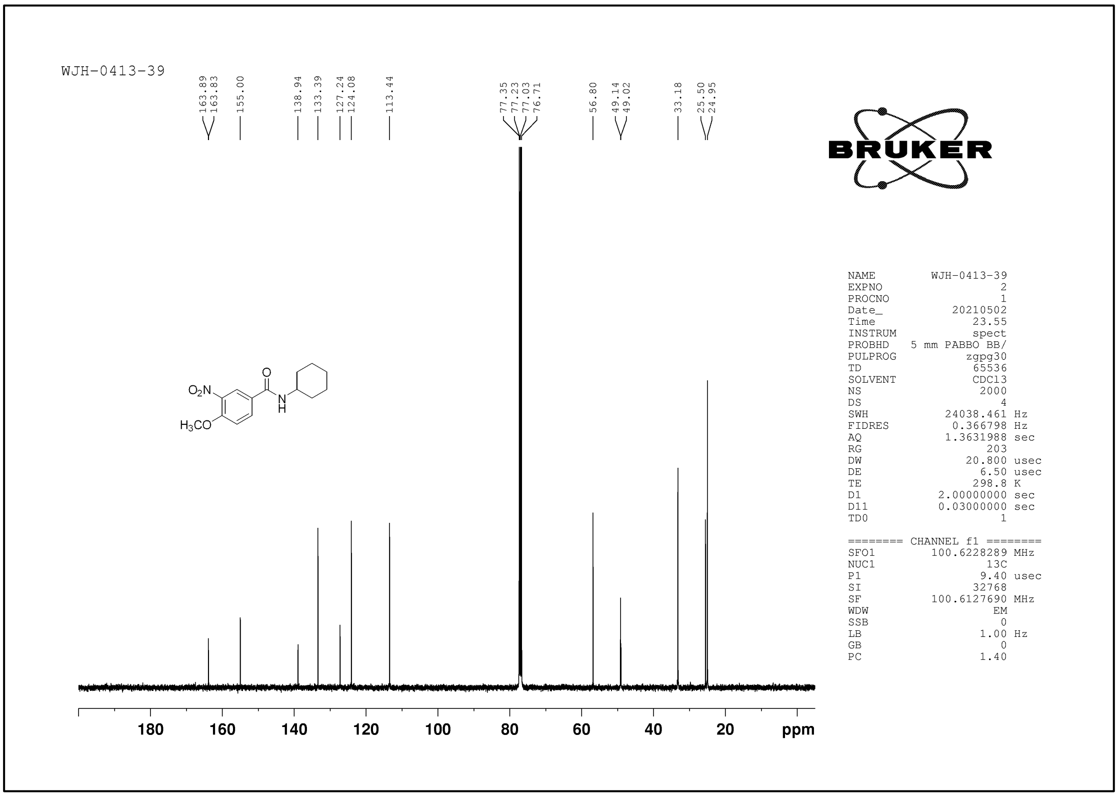

^{13}C-NMR spectrum of compound 17i

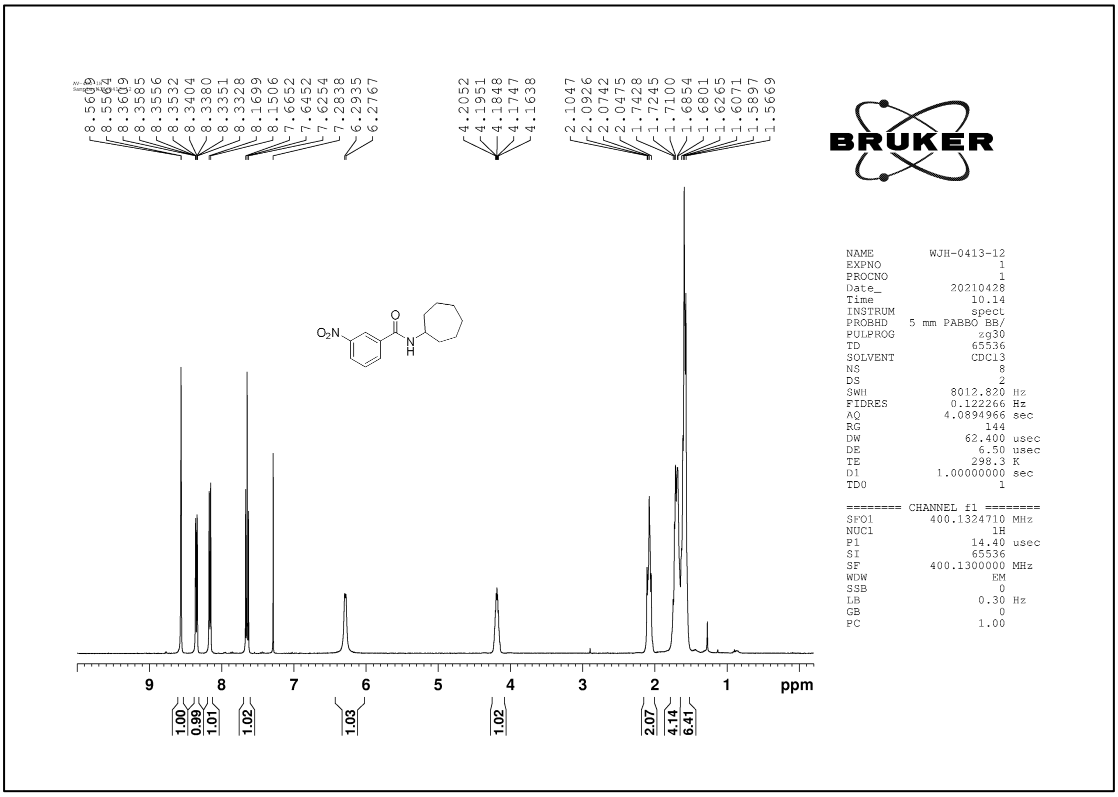

^{1}H-NMR spectrum of compound 17j

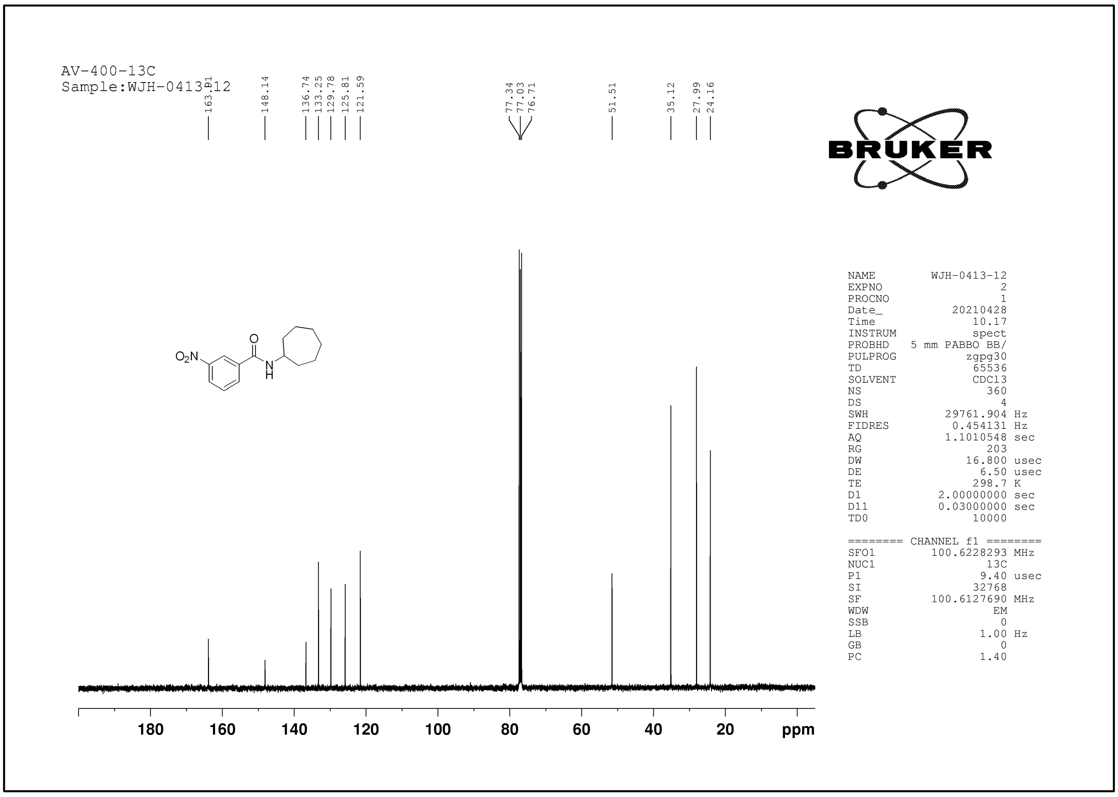

^{13}C-NMR spectrum of compound 17j

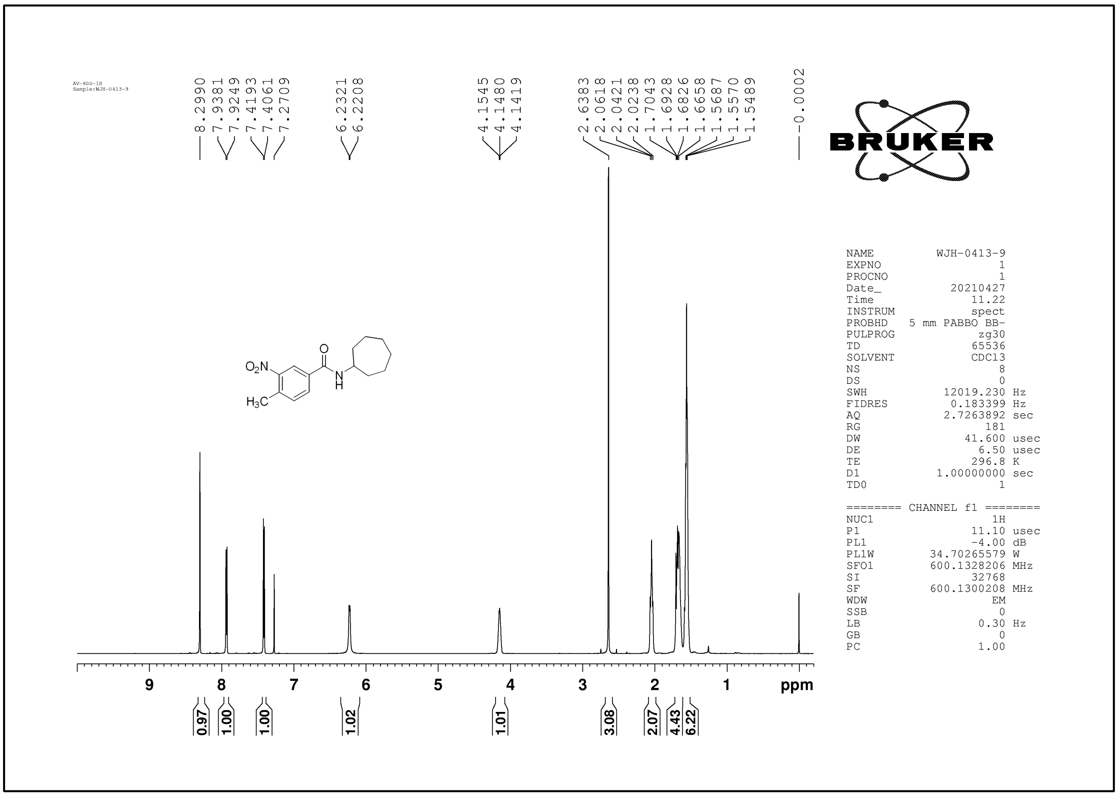

^{1}H-NMR spectrum of compound 17k

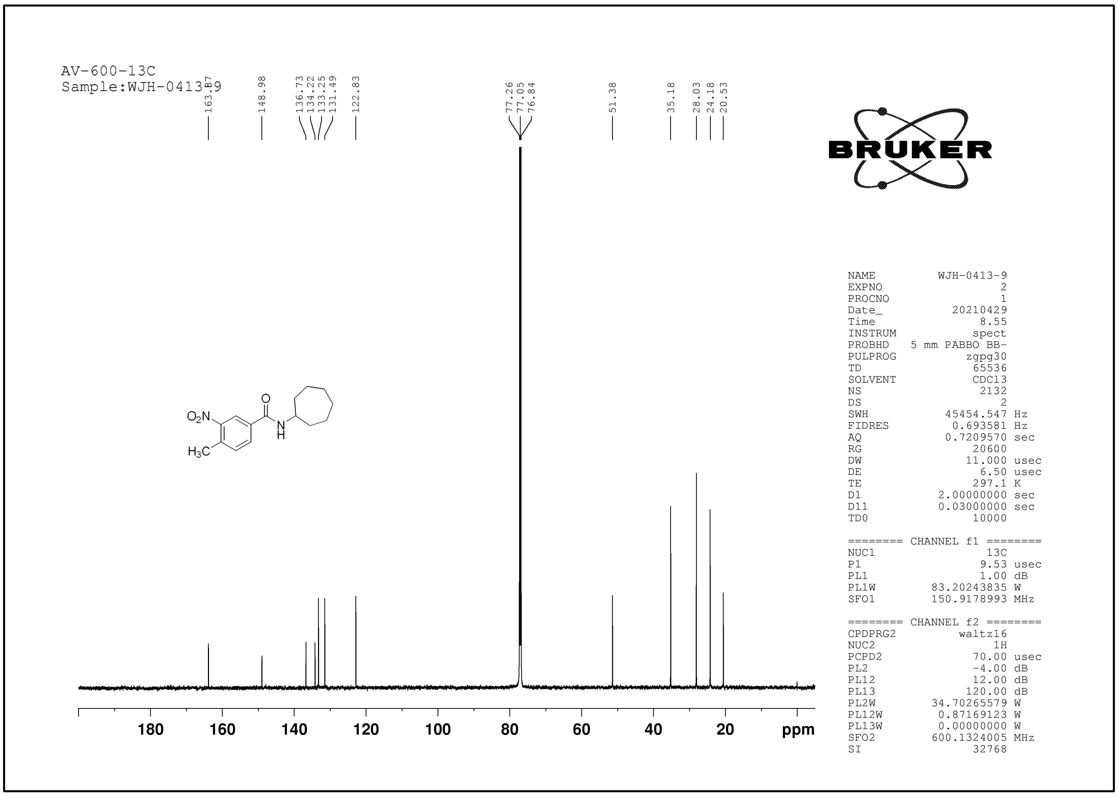

^{13}C-NMR spectrum of compound 17k

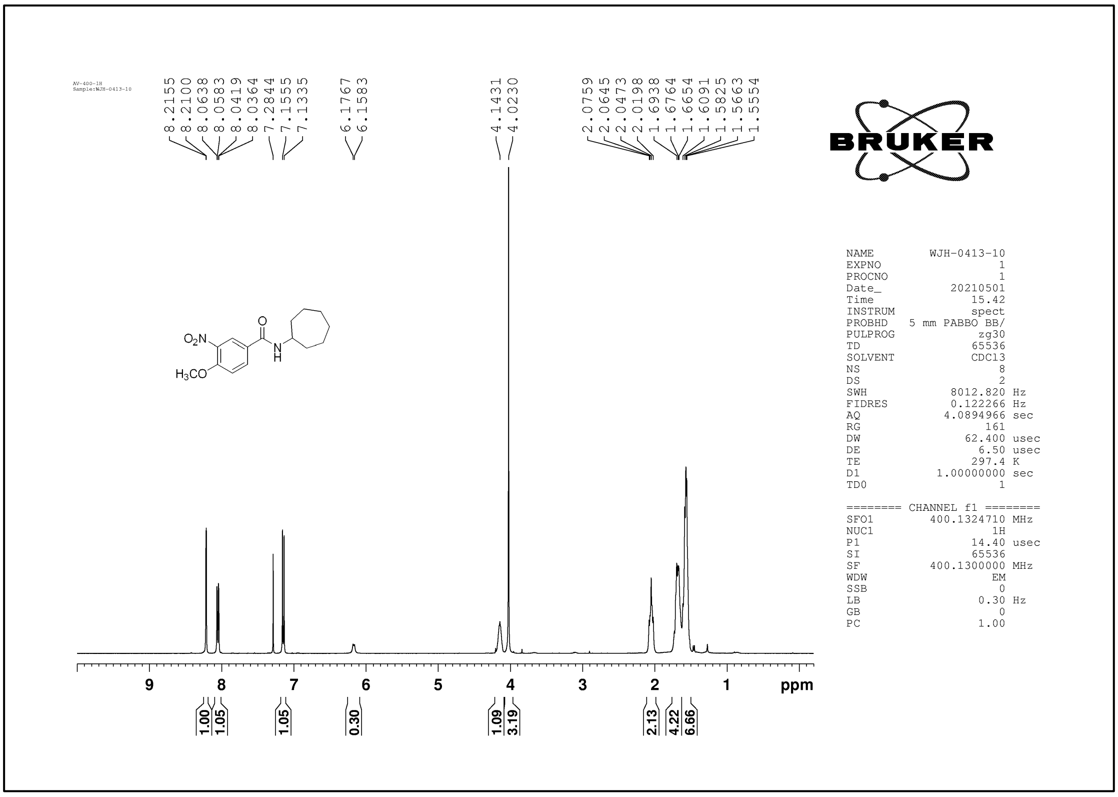

^{1}H-NMR spectrum of compound 17l

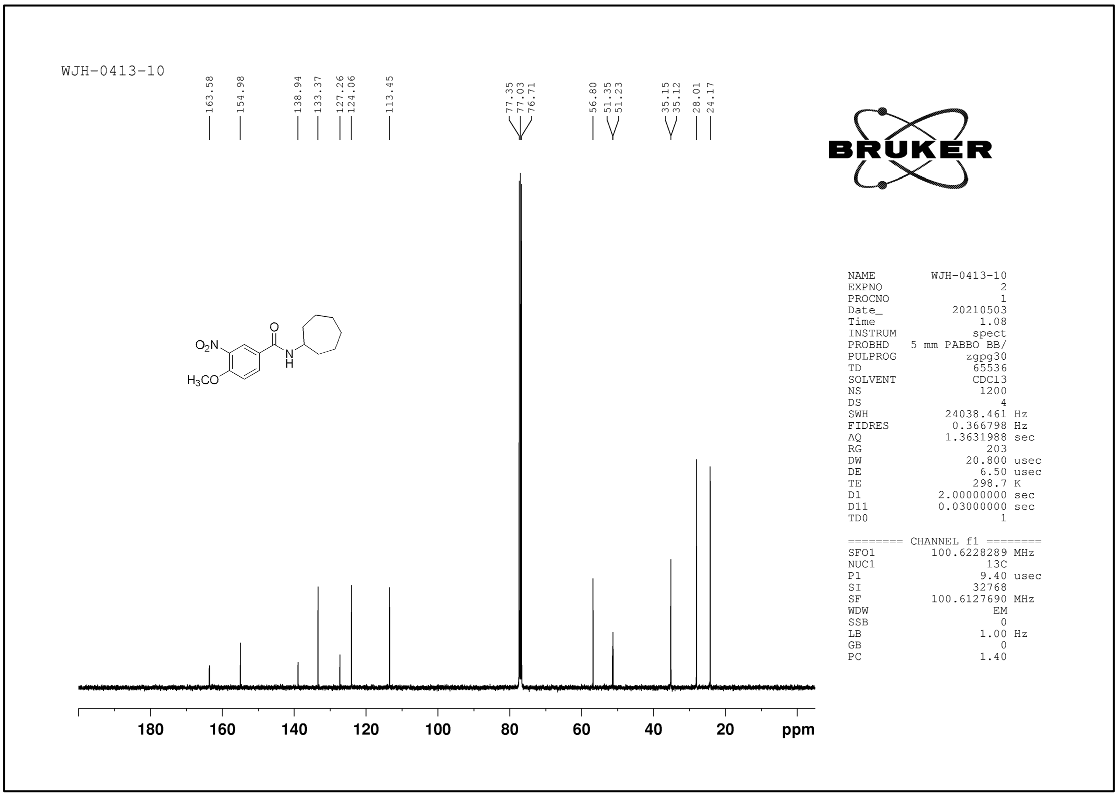

^{13}C-NMR spectrum of compound 17l

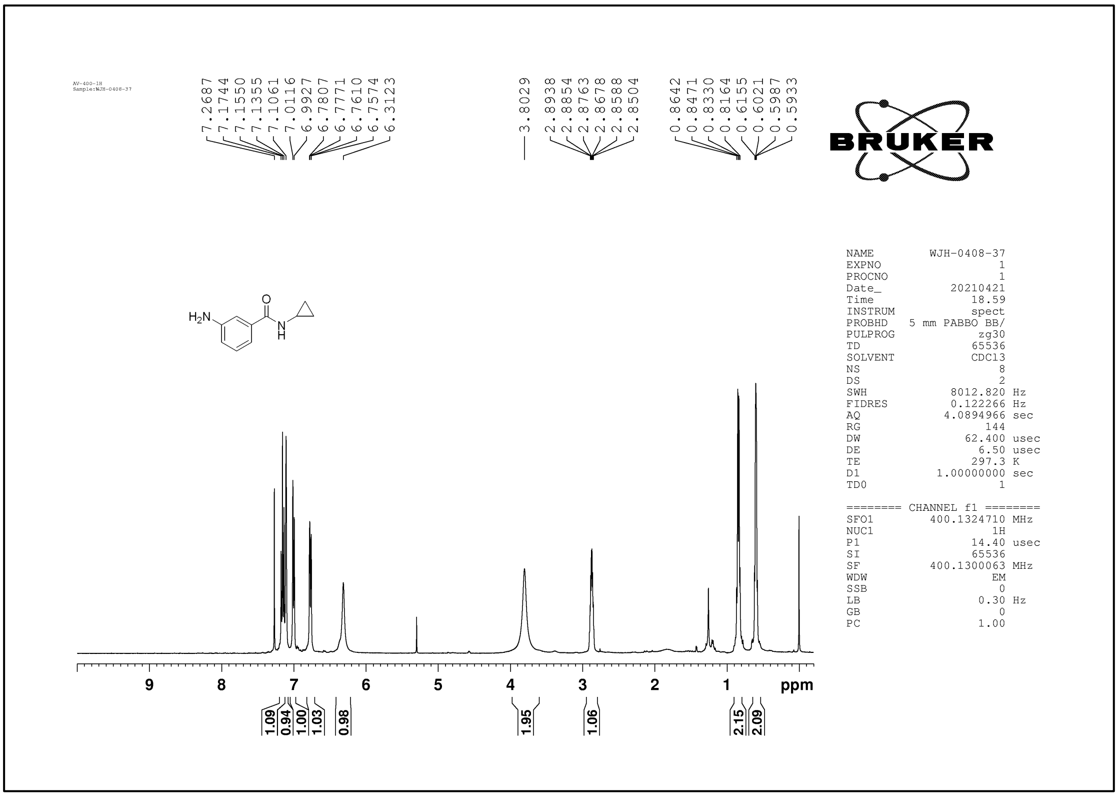

^{1}H-NMR spectrum of compound 18a

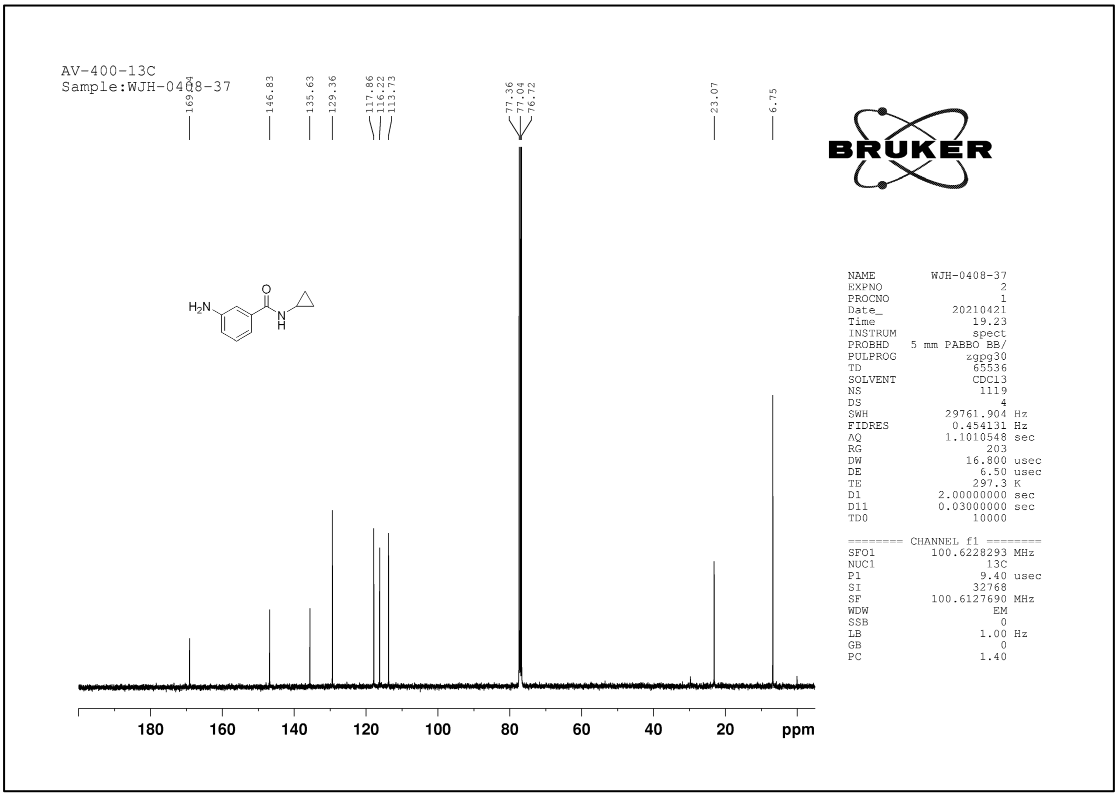

^{13}C-NMR spectrum of compound 18a

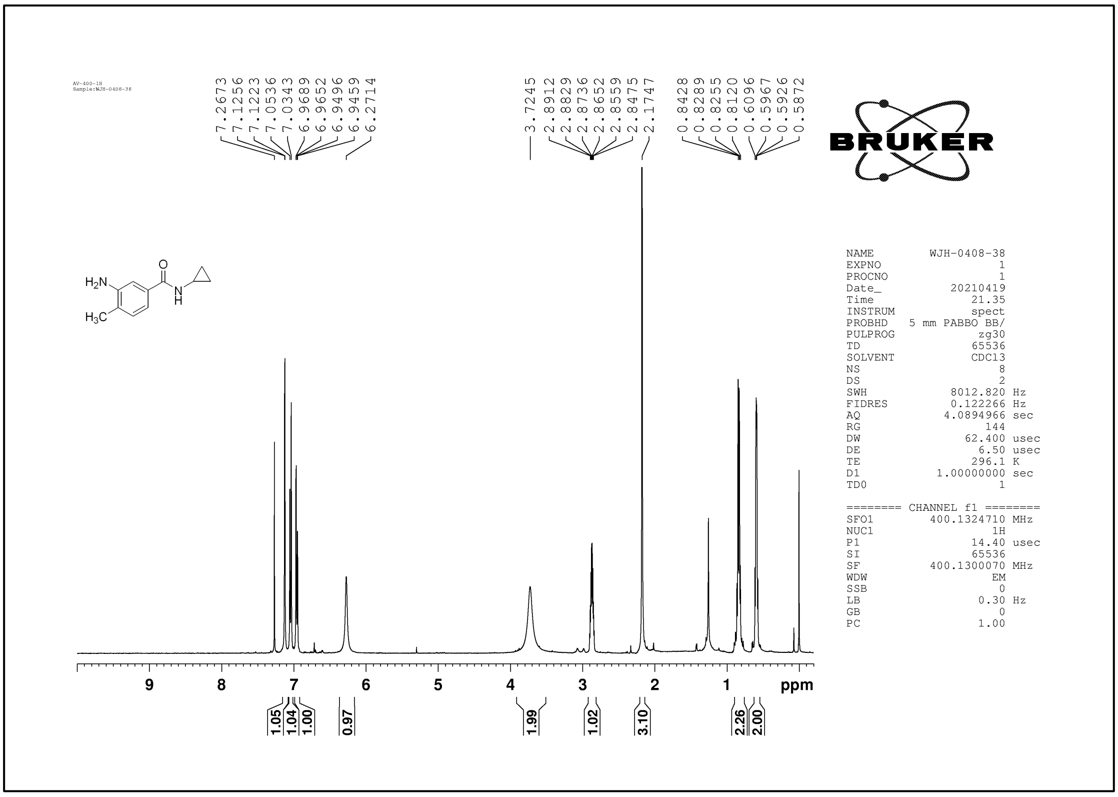

^{1}H-NMR spectrum of compound 18b

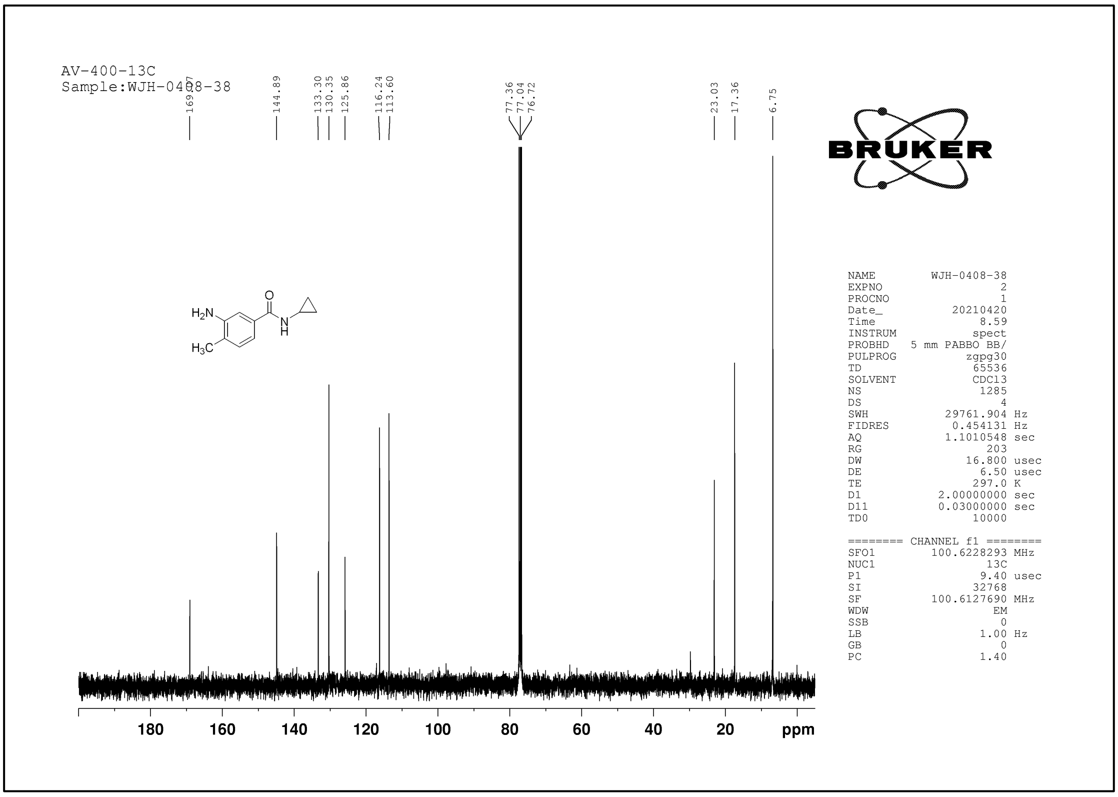

^{13}C-NMR spectrum of compound 18b

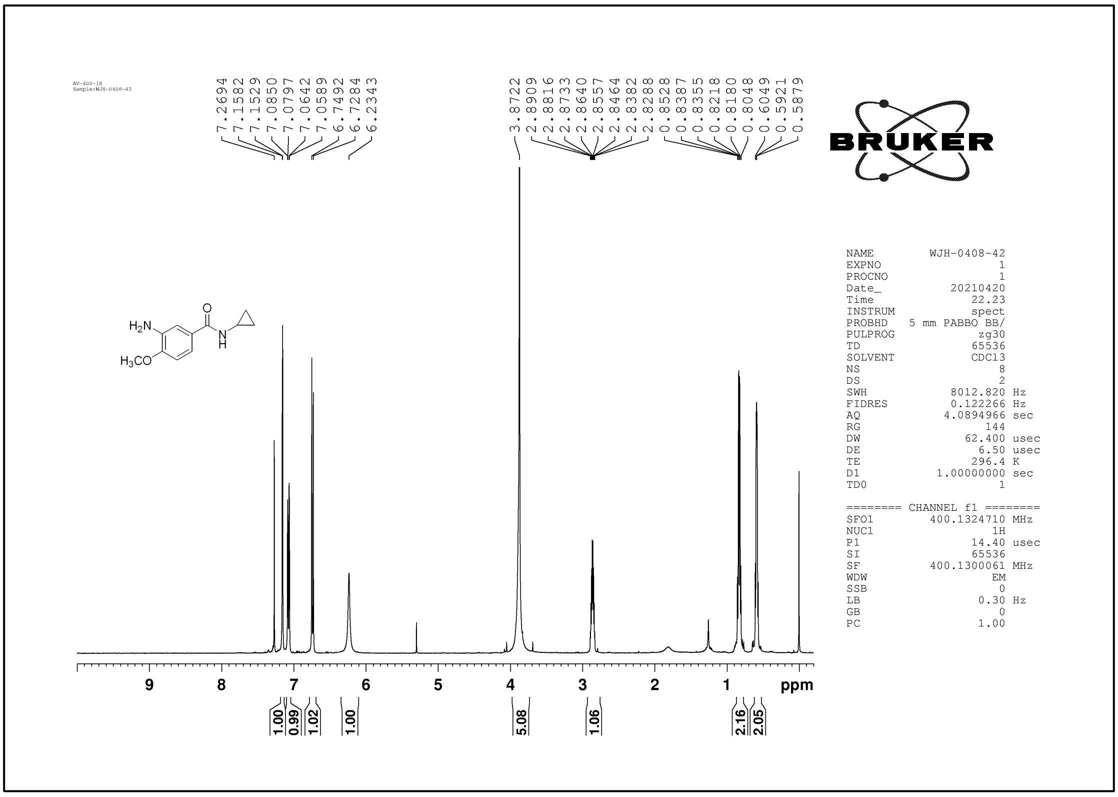

^{1}H-NMR spectrum of compound 18c

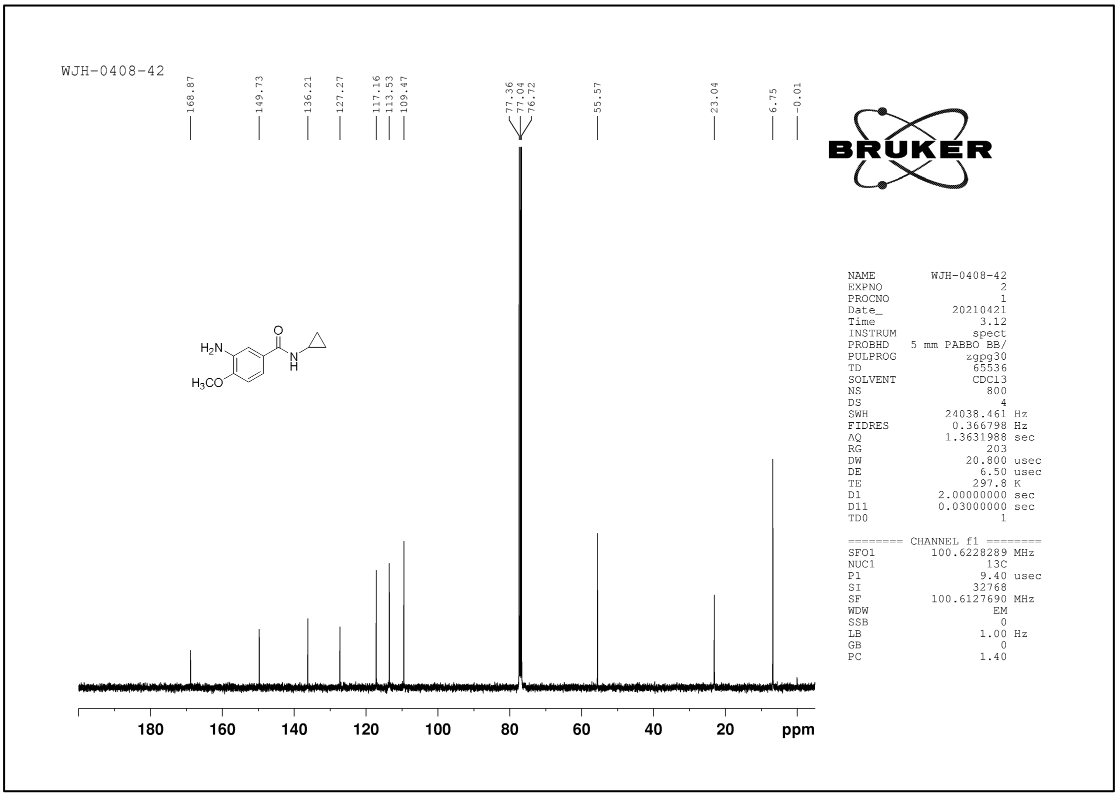

^{13}C-NMR spectrum of compound 18c

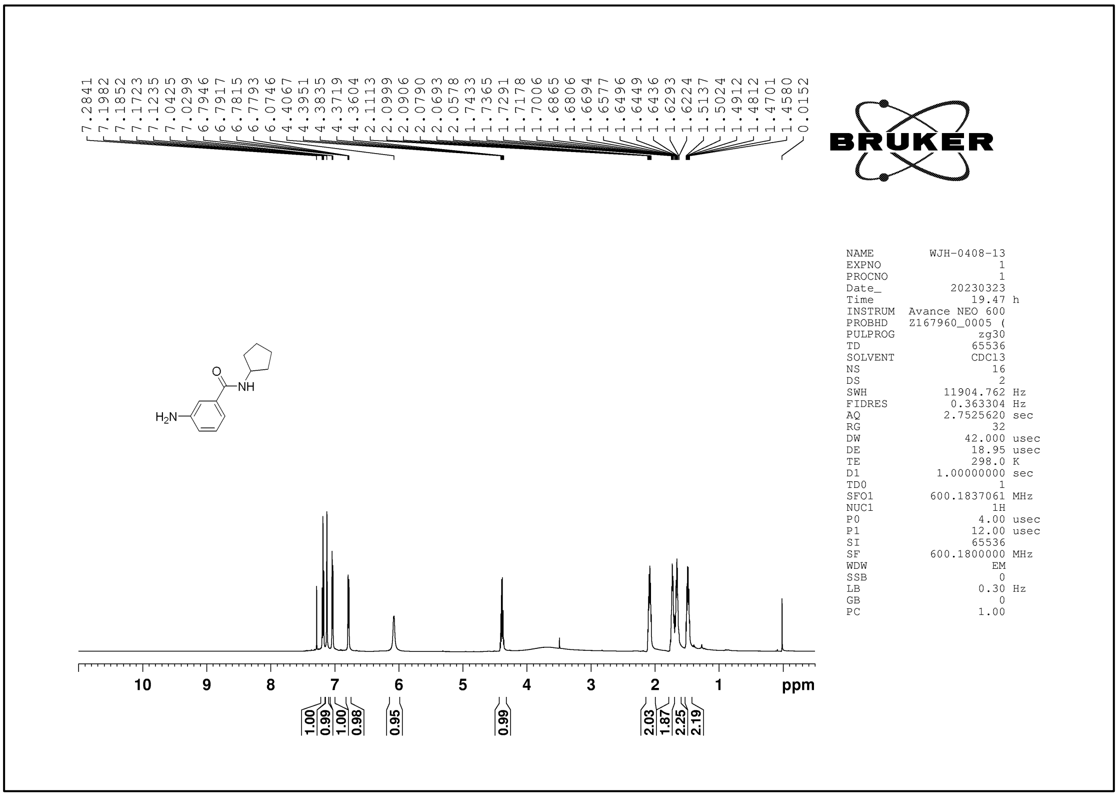

^{1}H-NMR spectrum of compound 18d

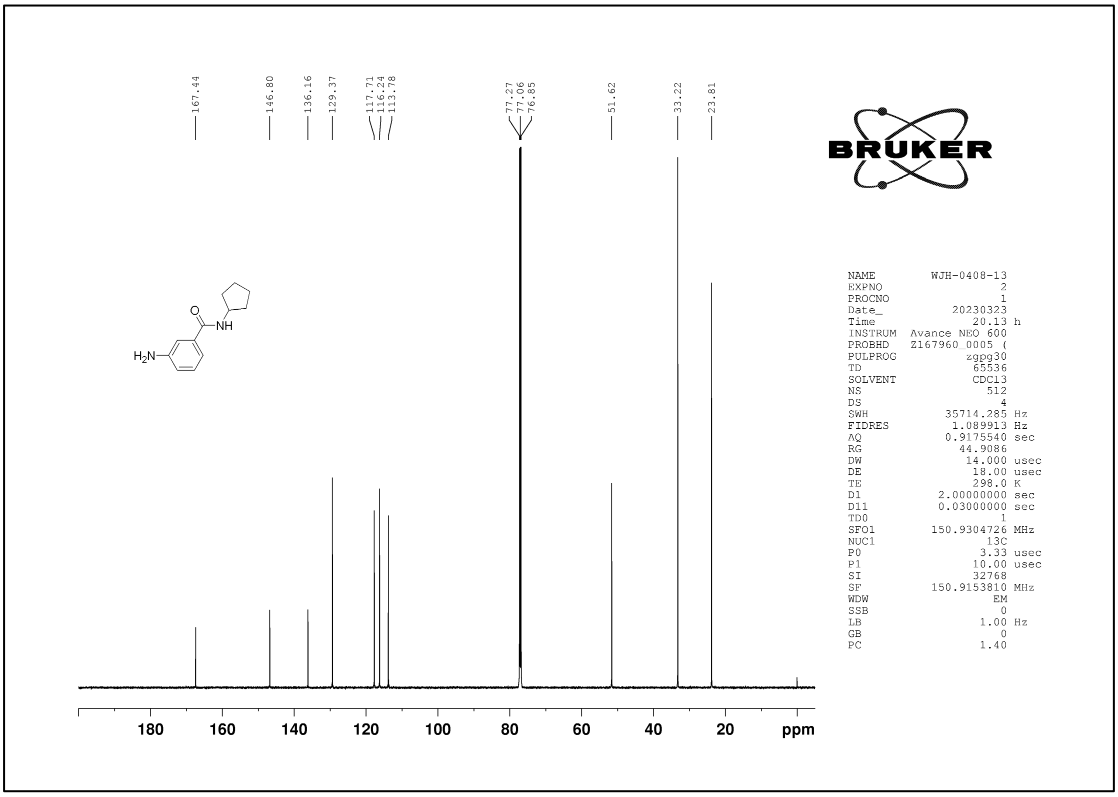

^{13}C-NMR spectrum of compound 18d

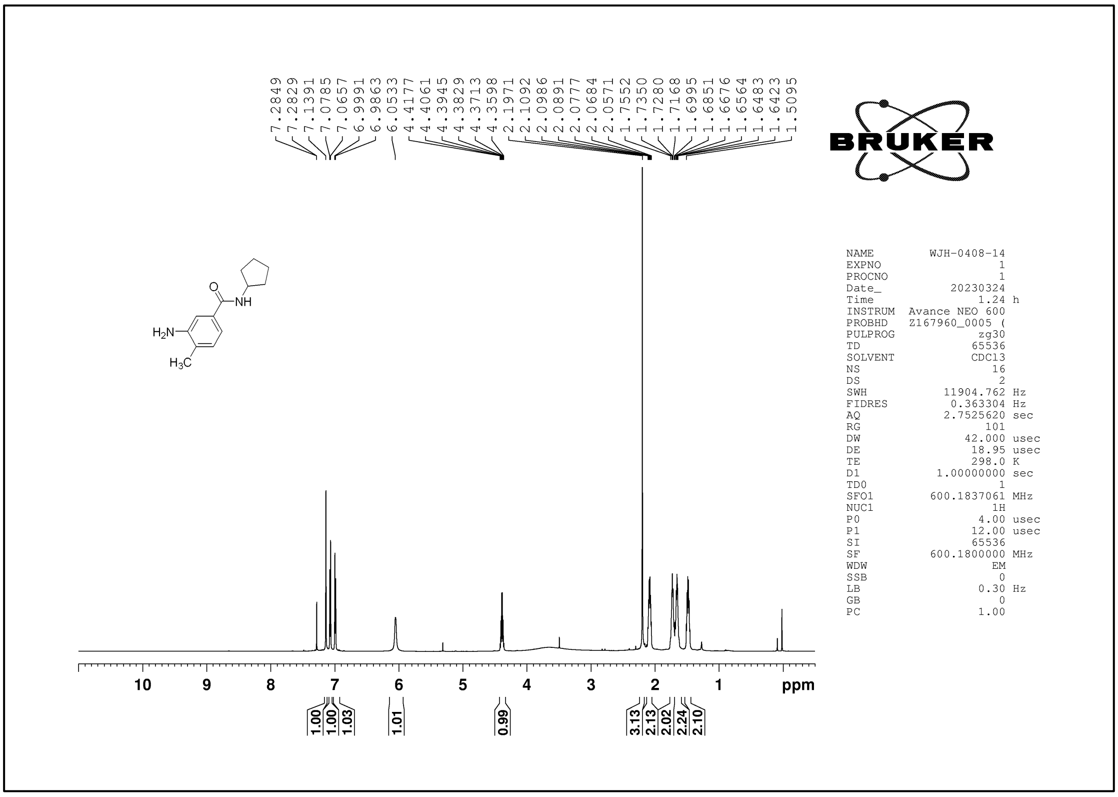

^{1}H-NMR spectrum of compound 18e

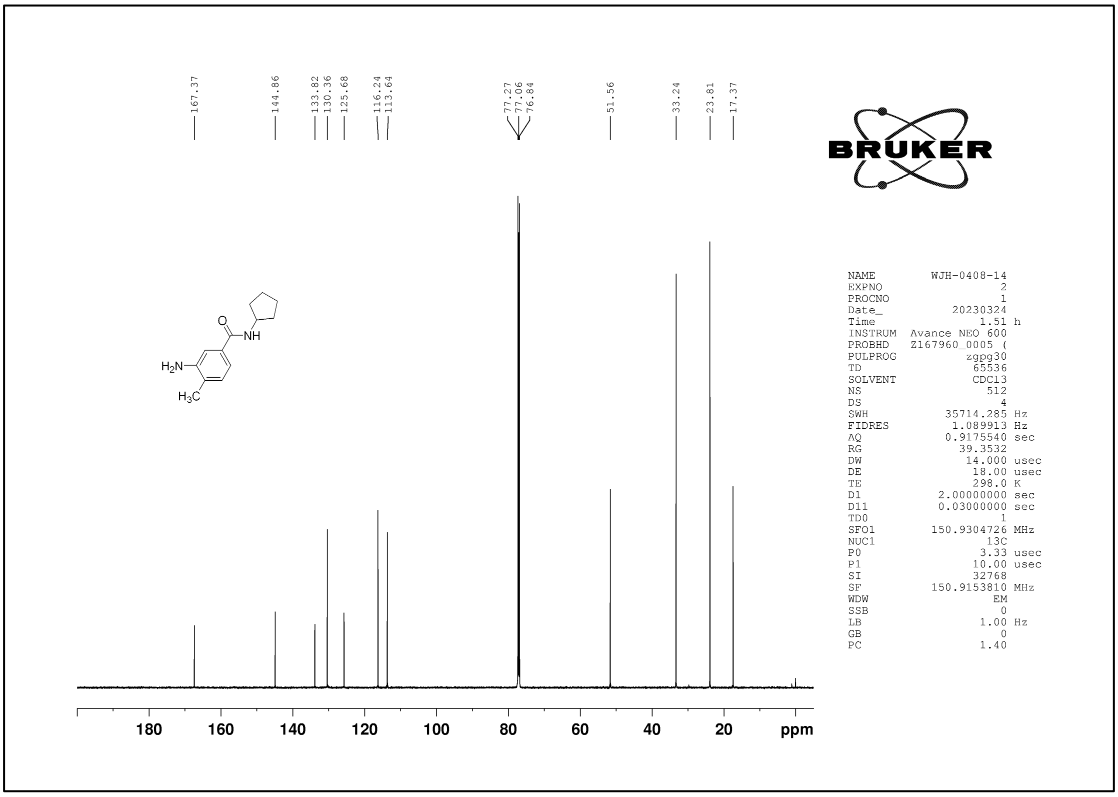

^{13}C-NMR spectrum of compound 18e

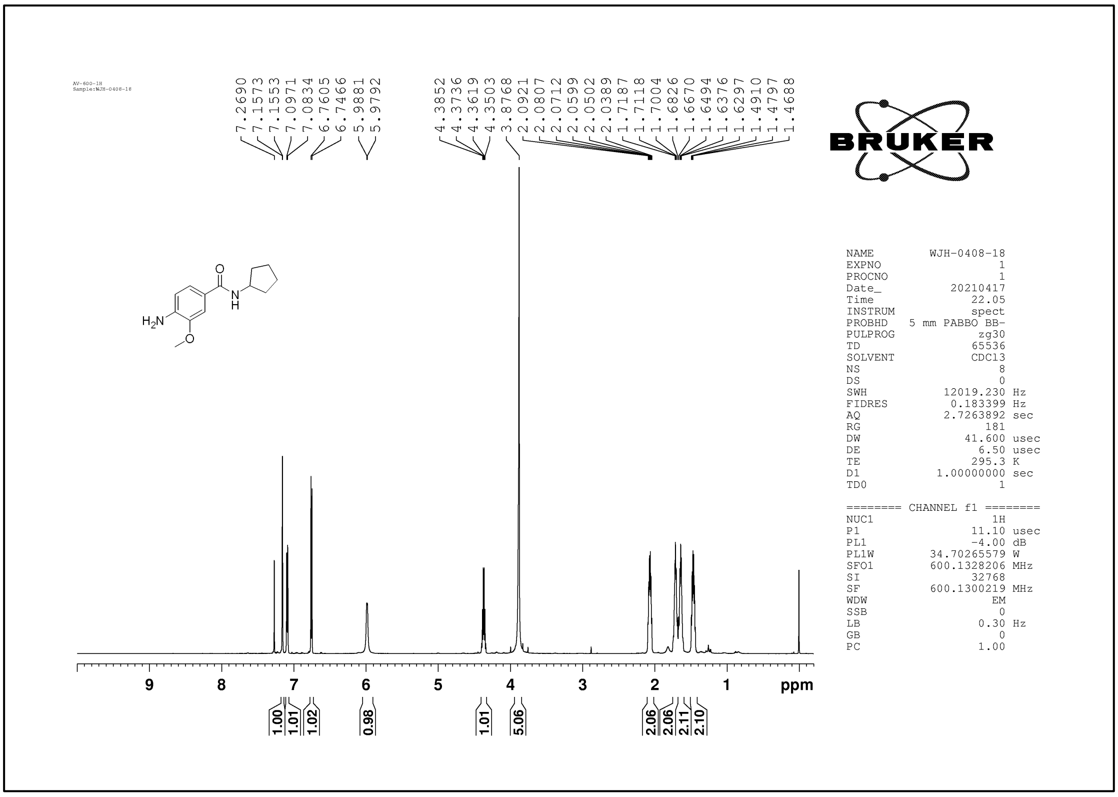

^{1}H-NMR spectrum of compound 18f

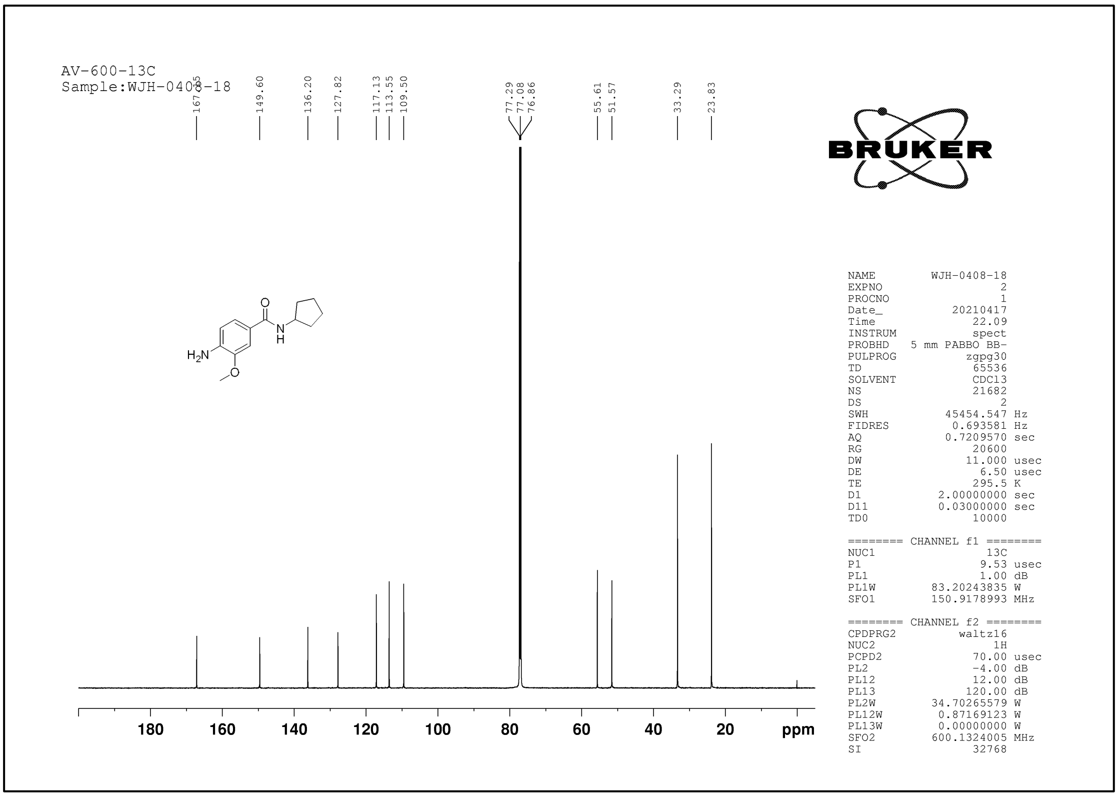

^{13}C-NMR spectrum of compound 18f

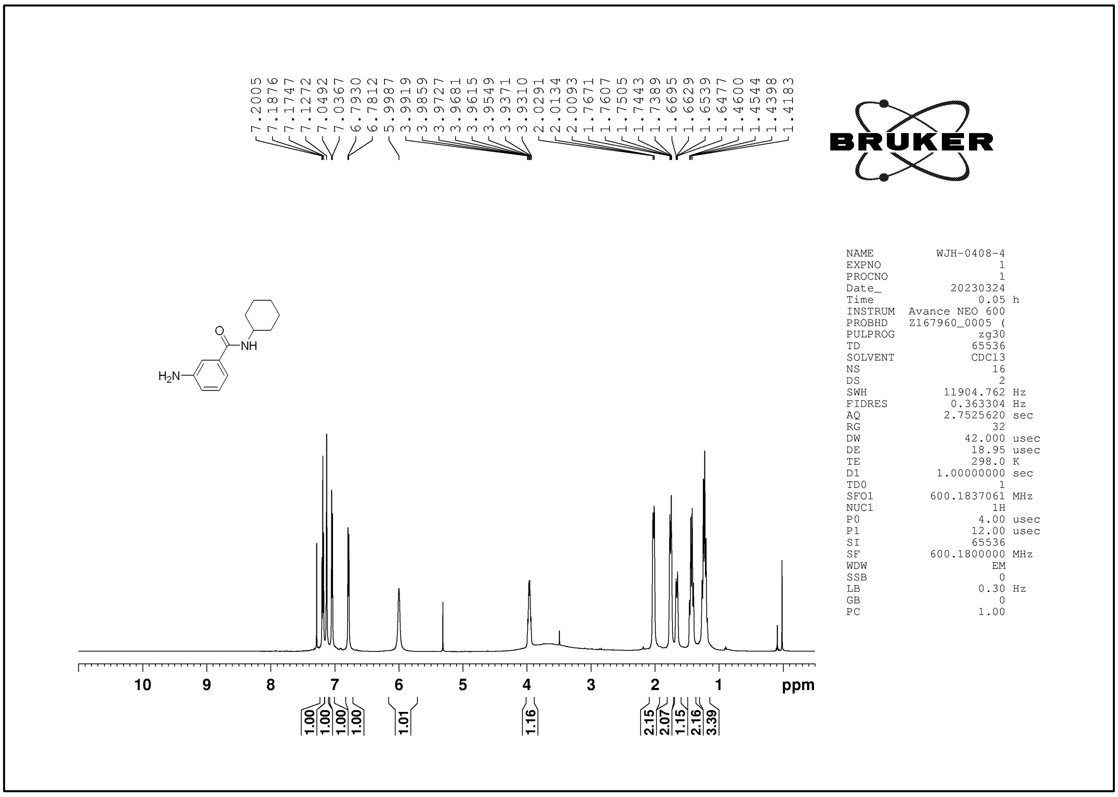

^{1}H-NMR spectrum of compound 18g

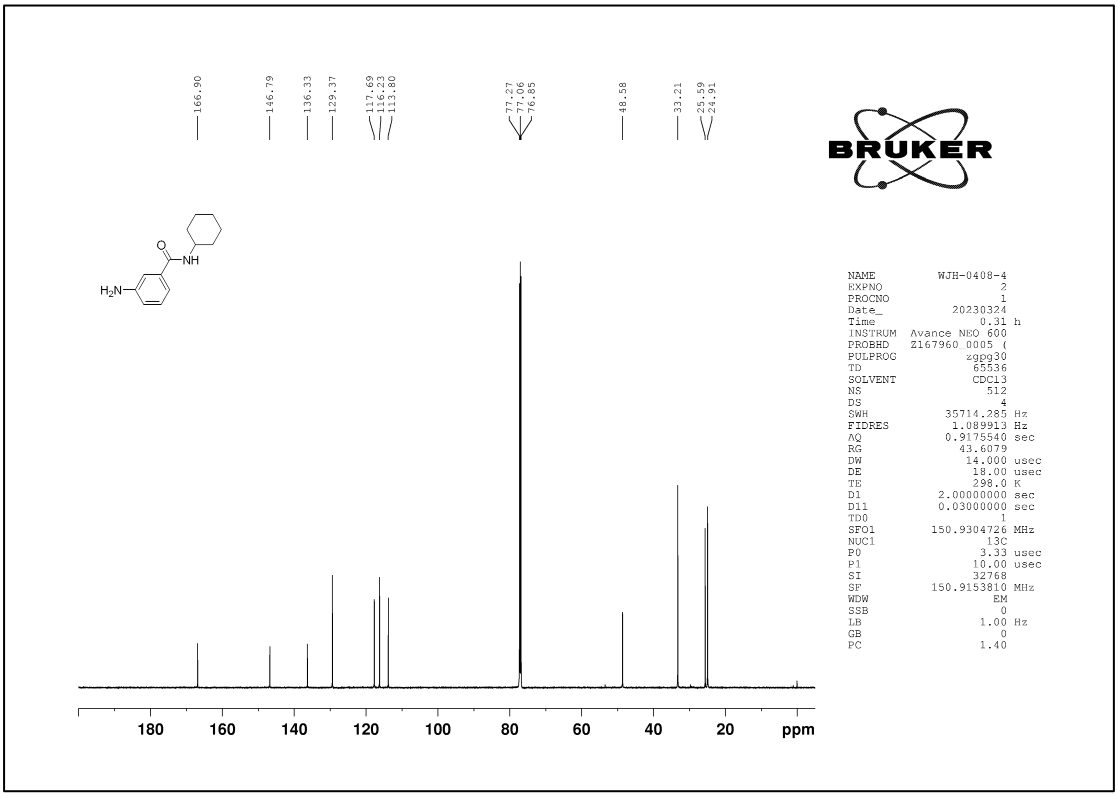

^{13}C-NMR spectrum of compound 18g

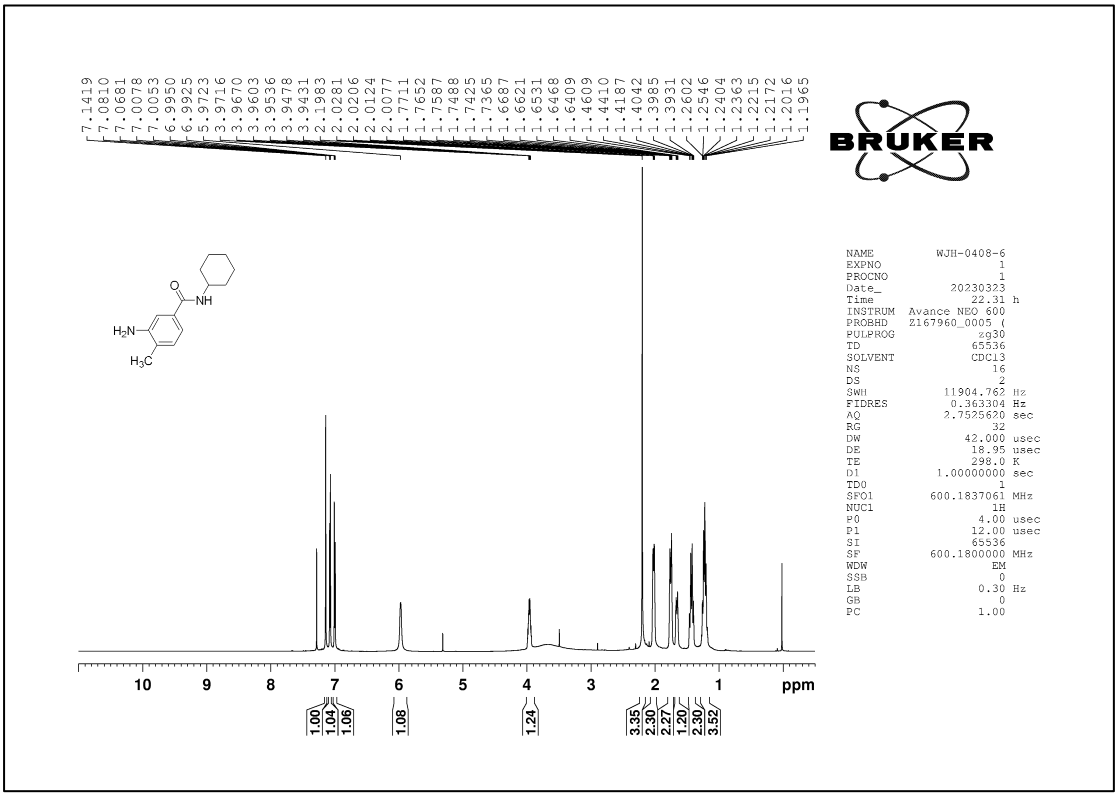

^{1}H-NMR spectrum of compound 18h

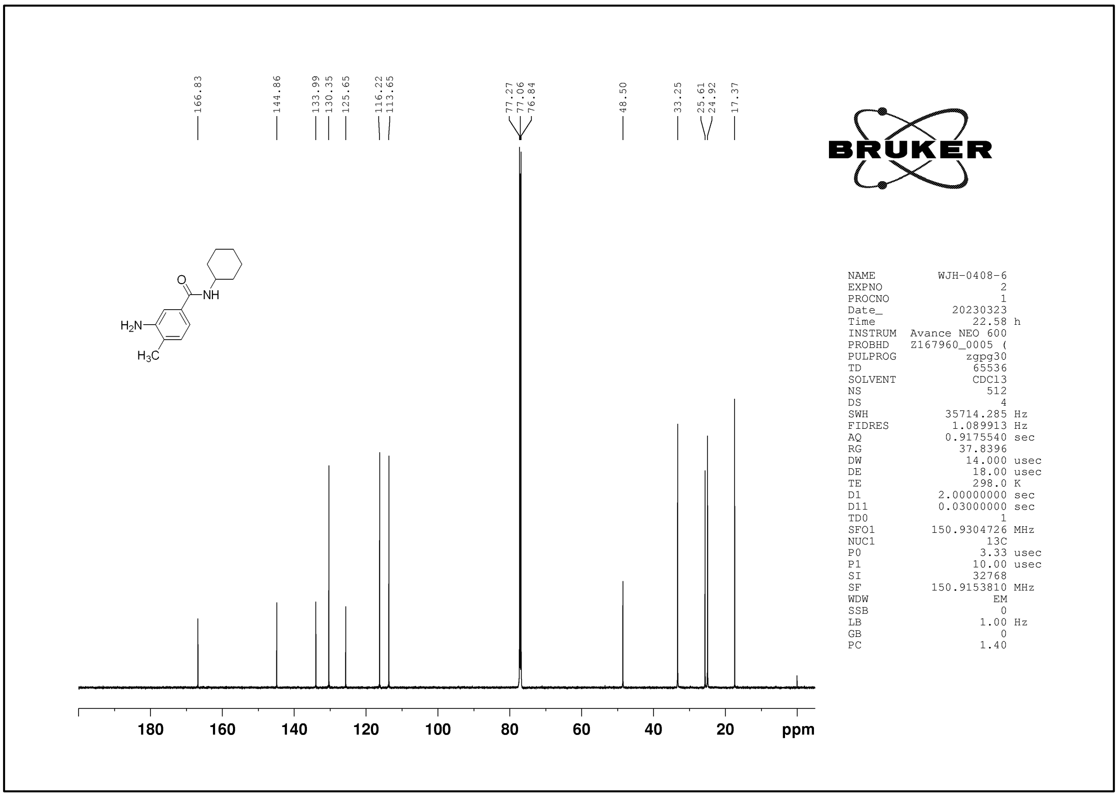

^{13}C-NMR spectrum of compound 18h

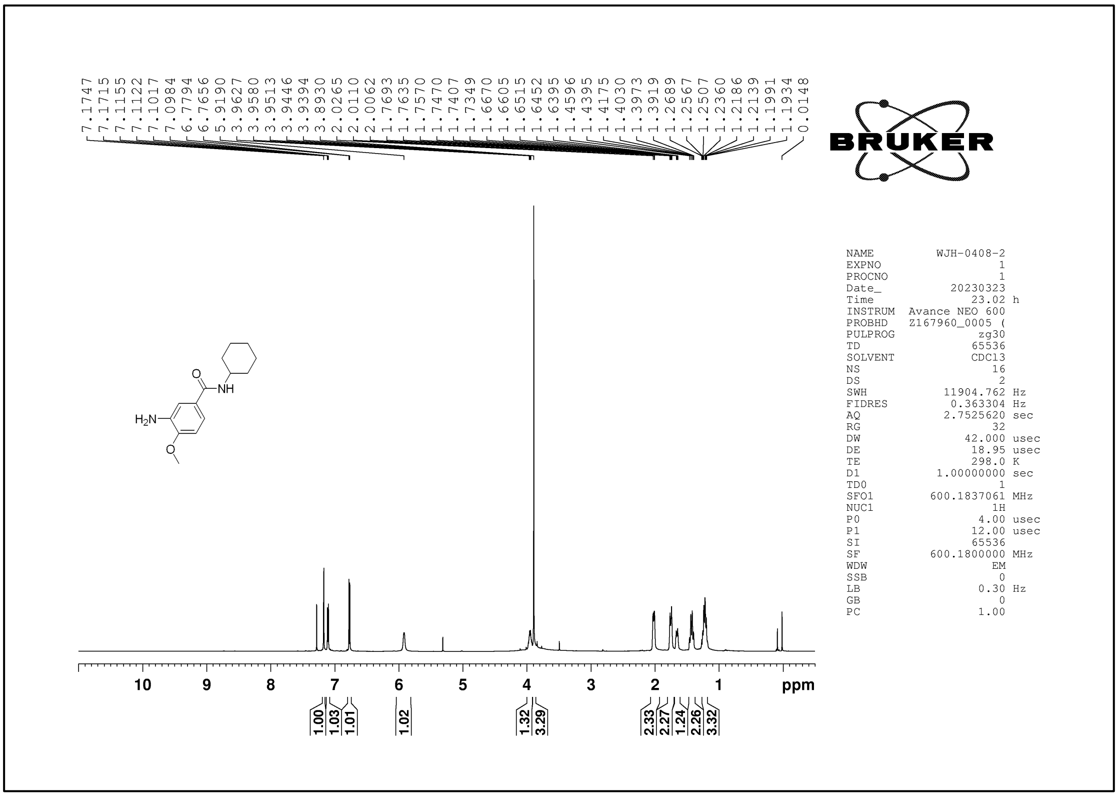

^{1}H-NMR spectrum of compound 18i

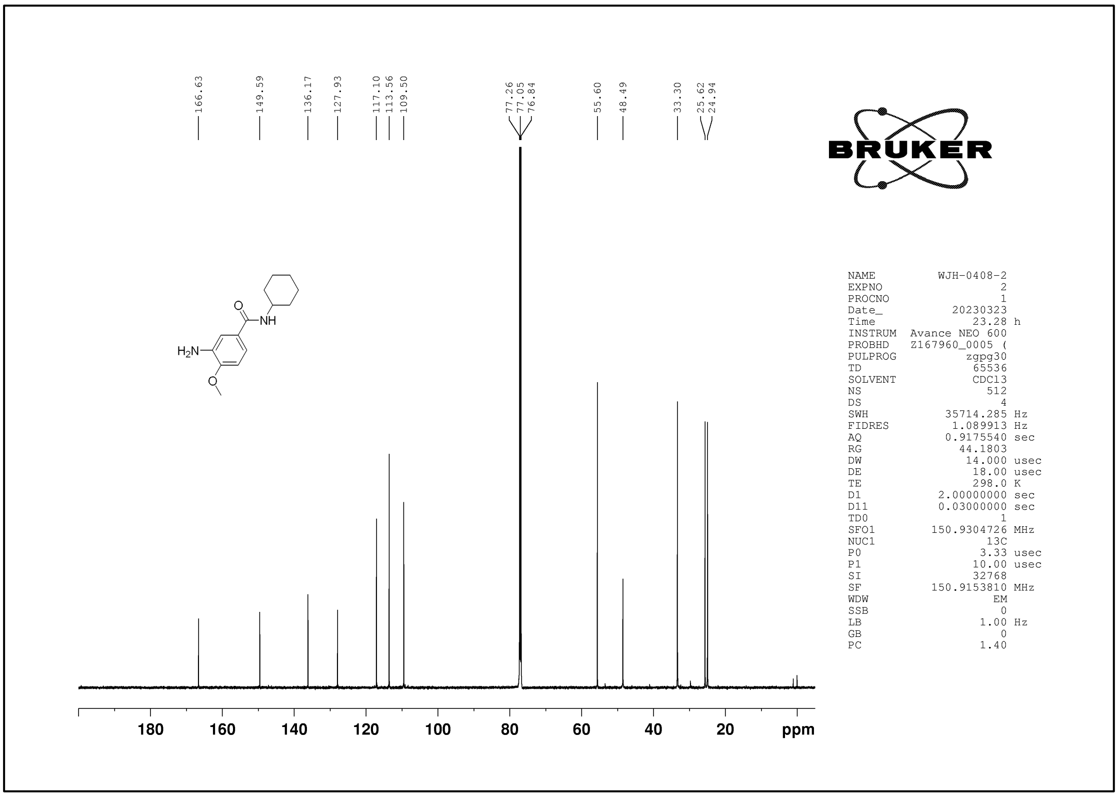

^{13}C-NMR spectrum of compound 18i

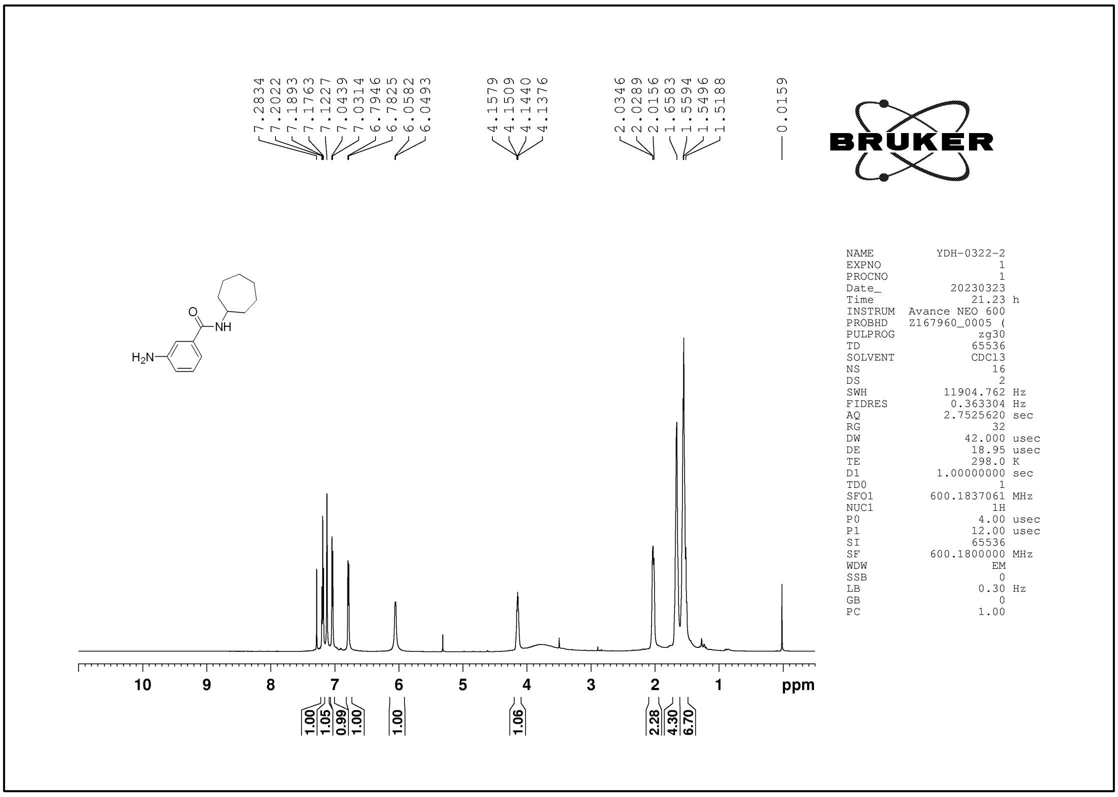

^{1}H-NMR spectrum of compound 18j

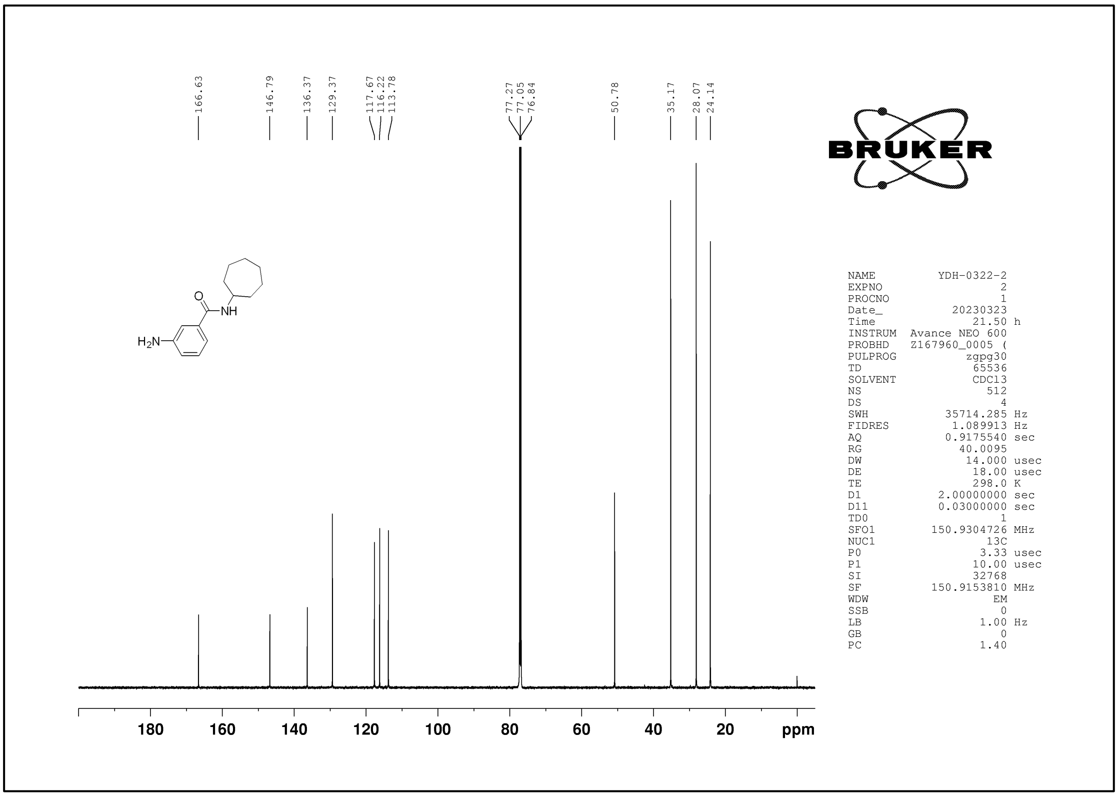

^{13}C-NMR spectrum of compound 18j

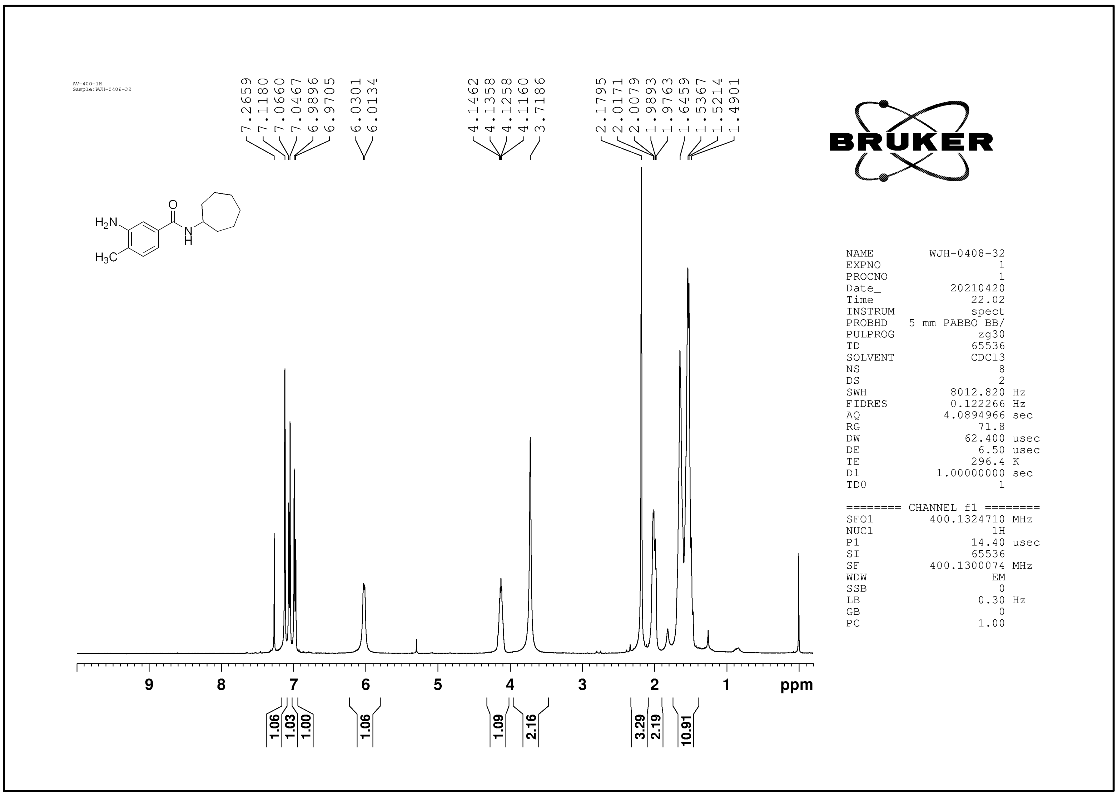

^{1}H-NMR spectrum of compound 18k

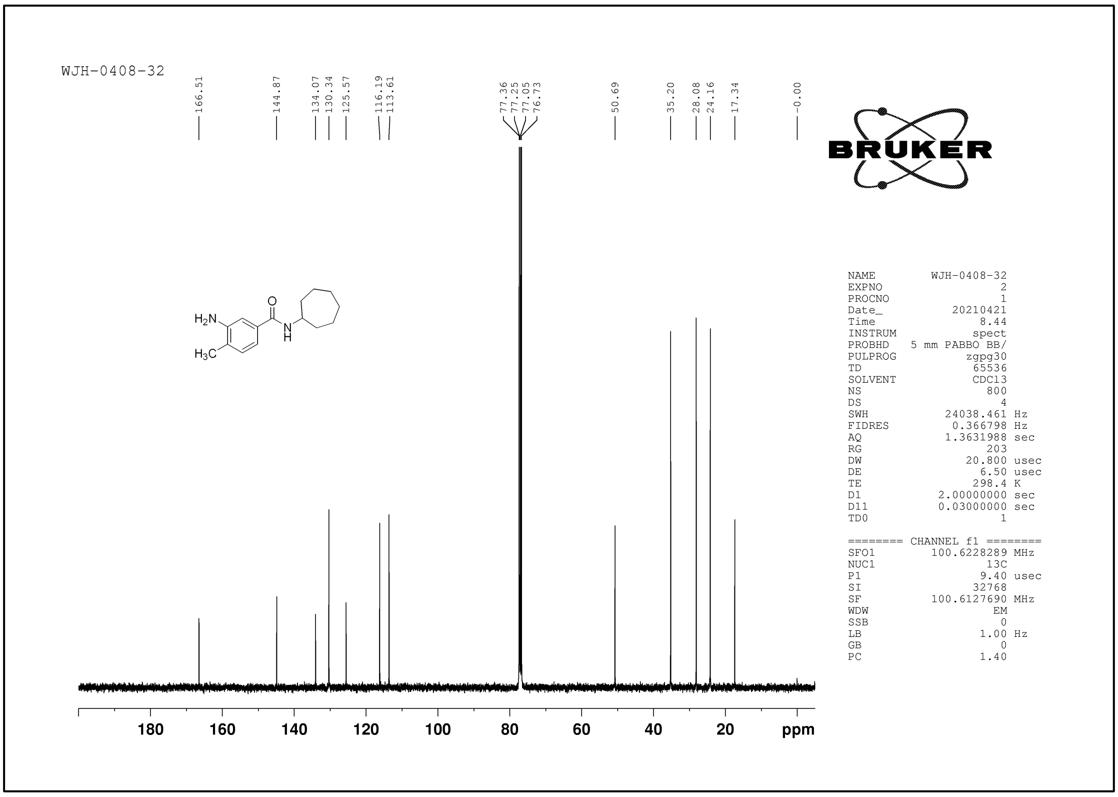

^{13}C-NMR spectrum of compound 18k

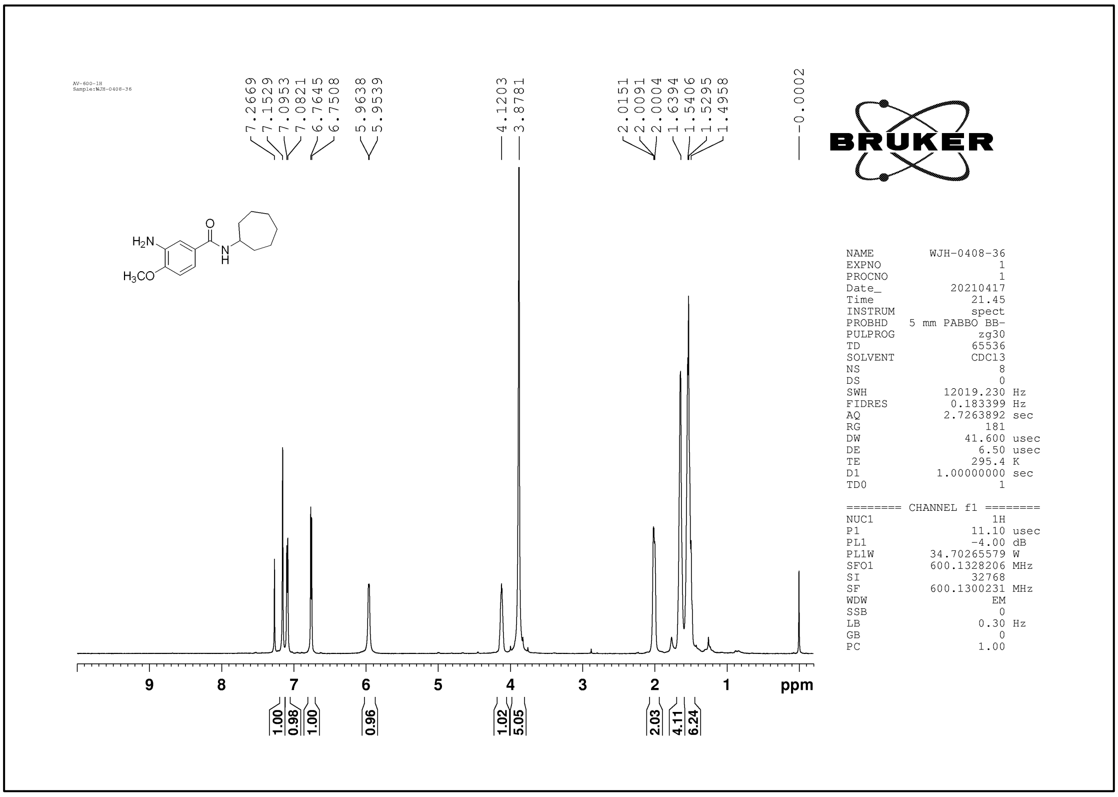

^{1}H-NMR spectrum of compound 18l

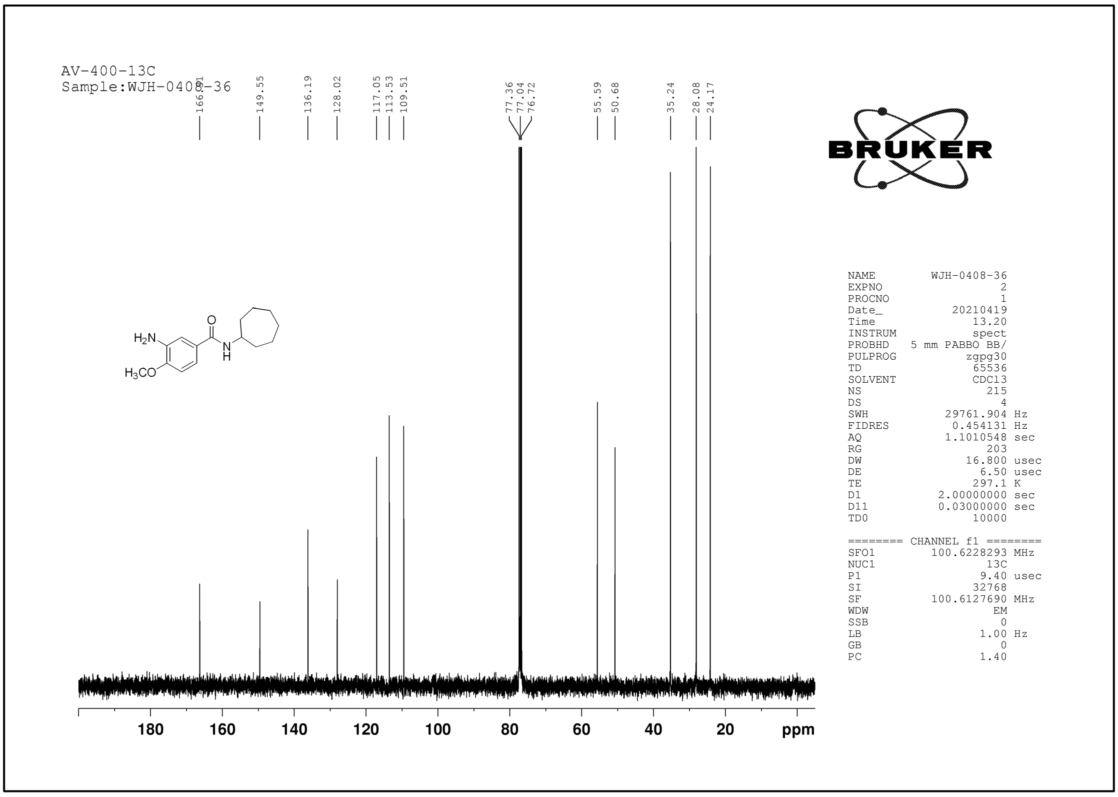

^{13}C-NMR spectrum of compound 18l

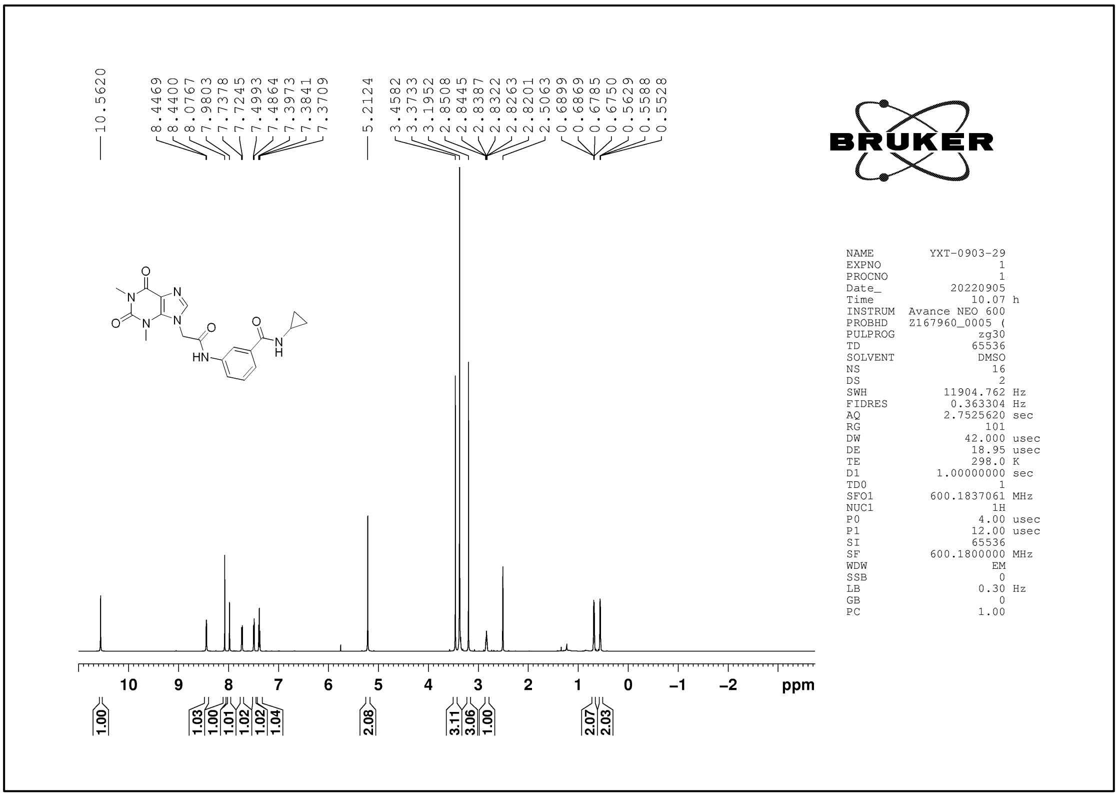

^{1}H-NMR spectrum of compound 19a

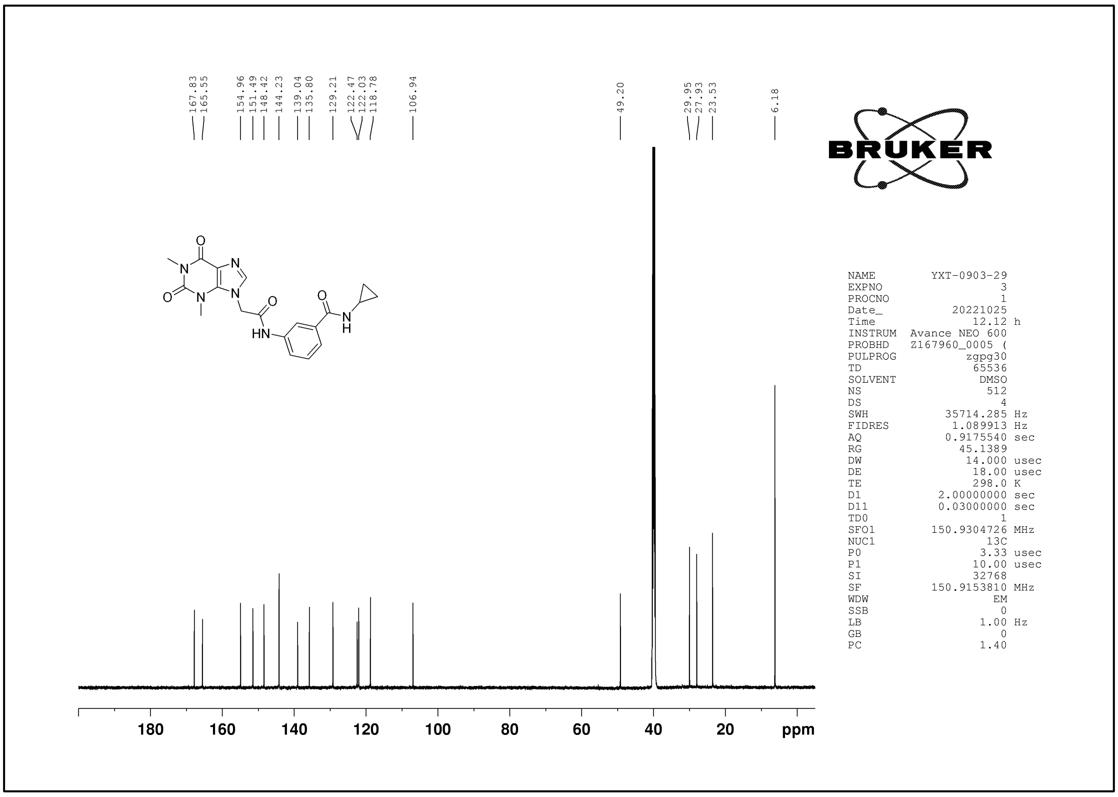

^{13}C-NMR spectrum of compound 19a

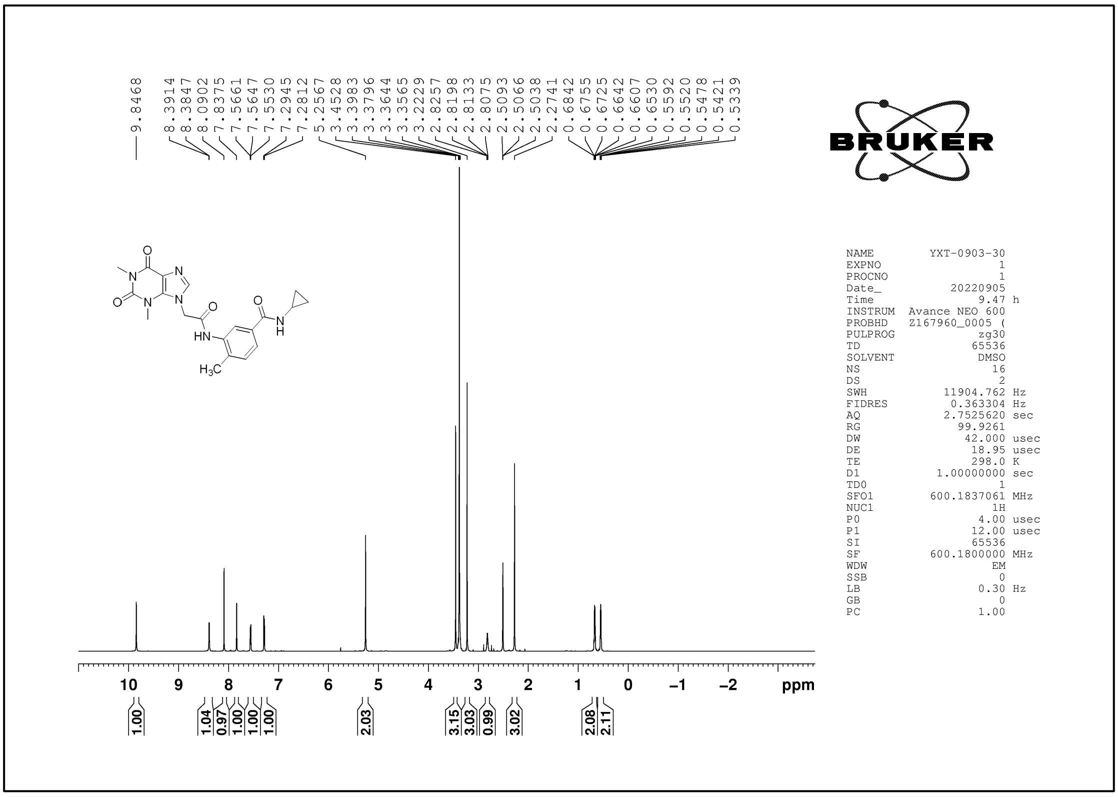

^{1}H-NMR spectrum of compound 19b

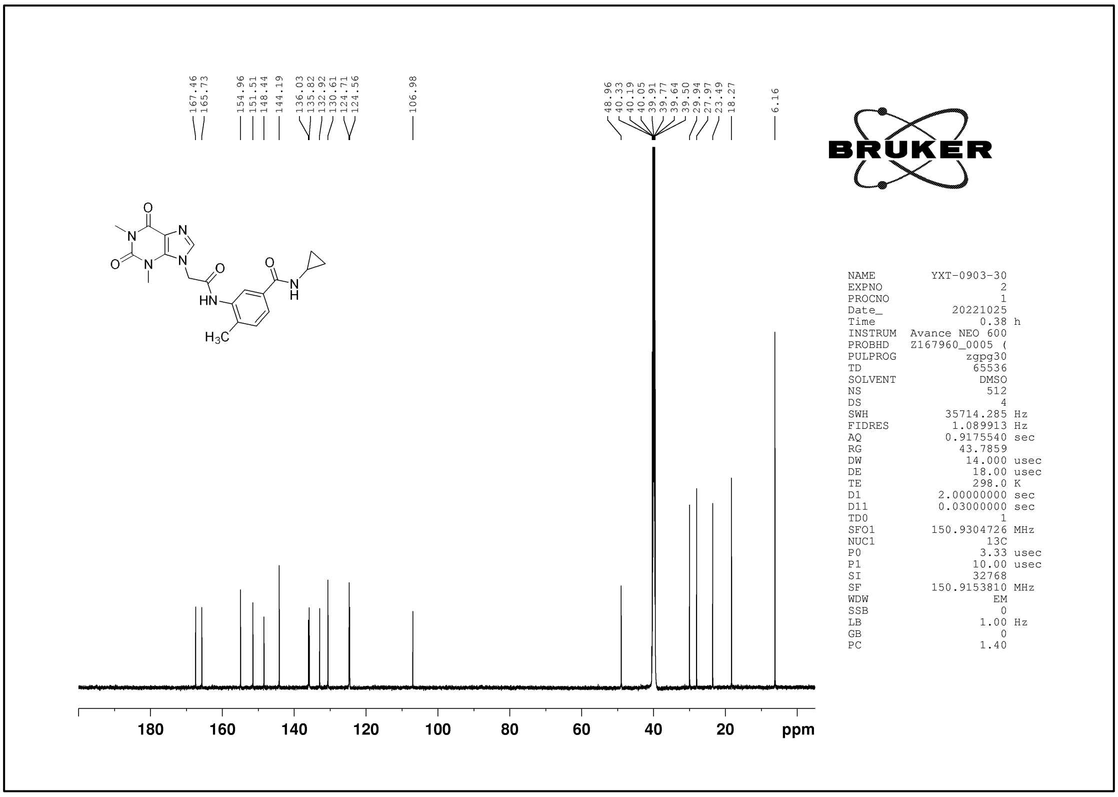

^{13}C-NMR spectrum of compound 19b

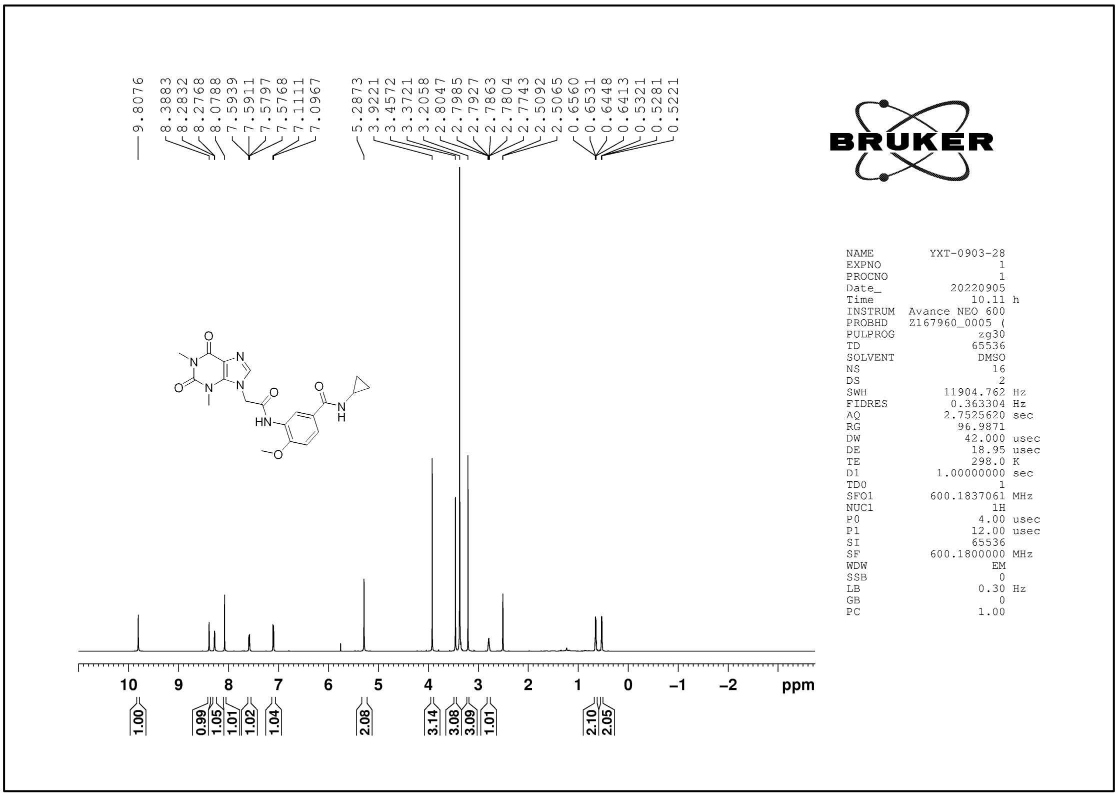

^{1}H-NMR spectrum of compound 19c

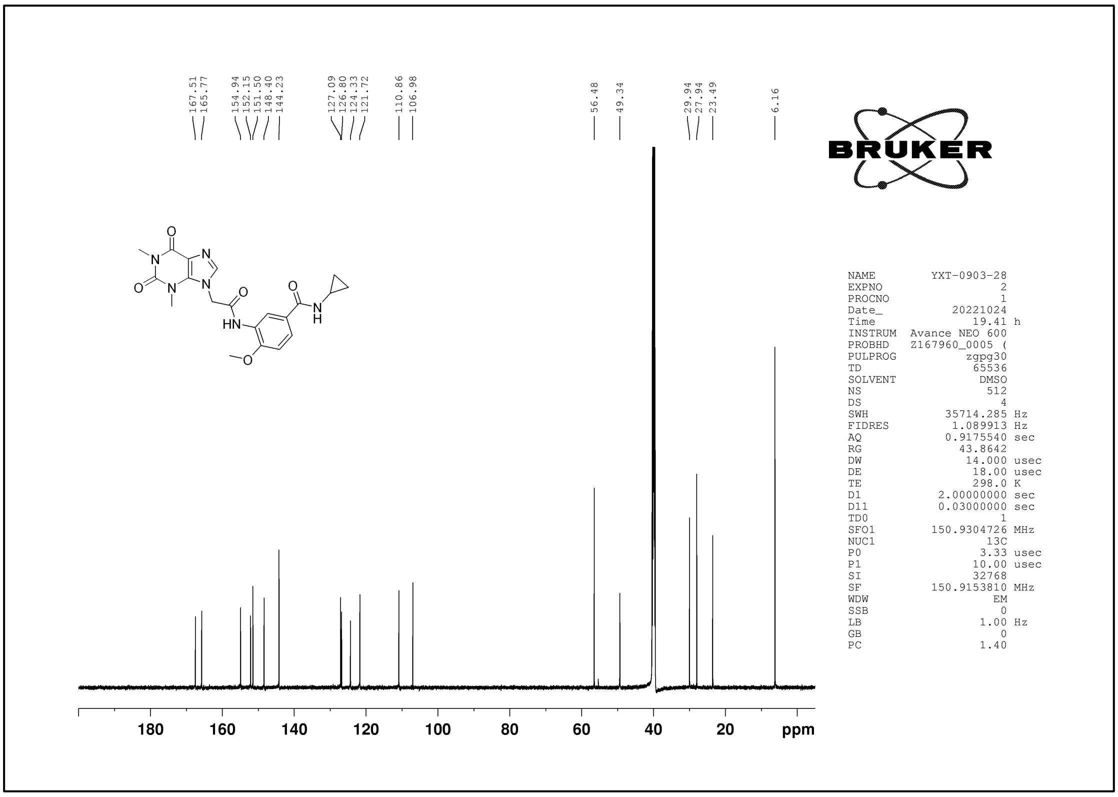

^{13}C-NMR spectrum of compound 19c

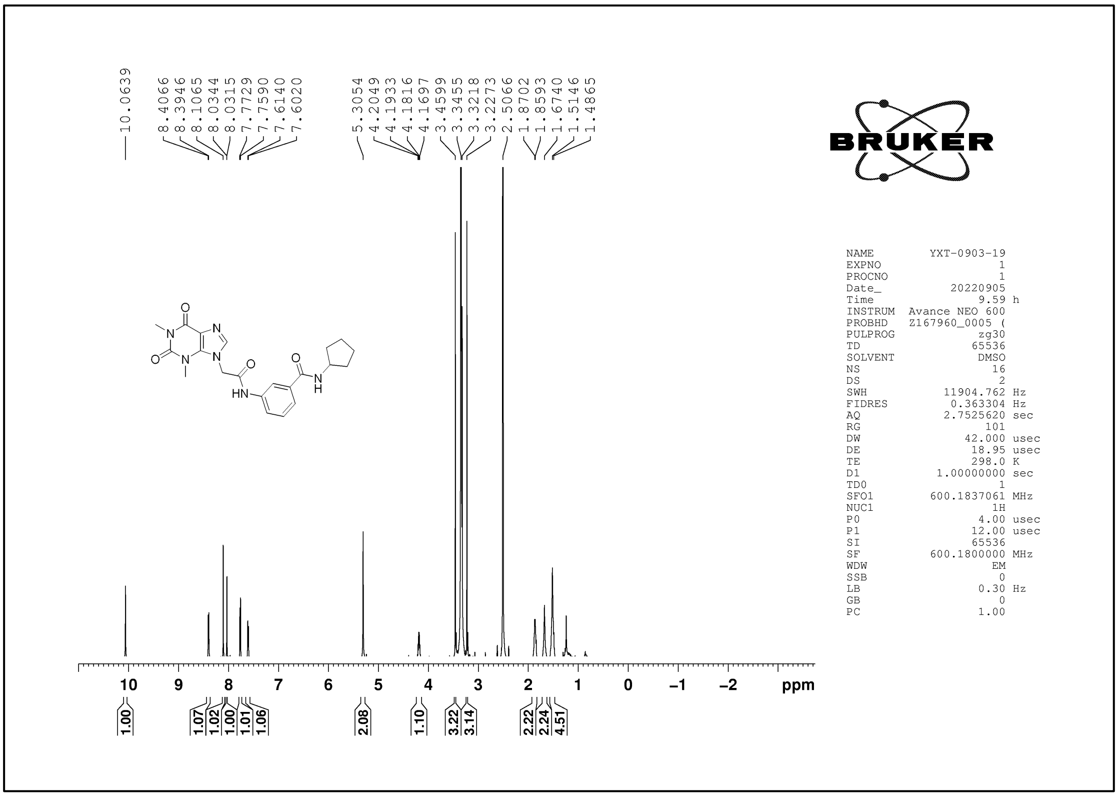

^{1}H-NMR spectrum of compound 19d

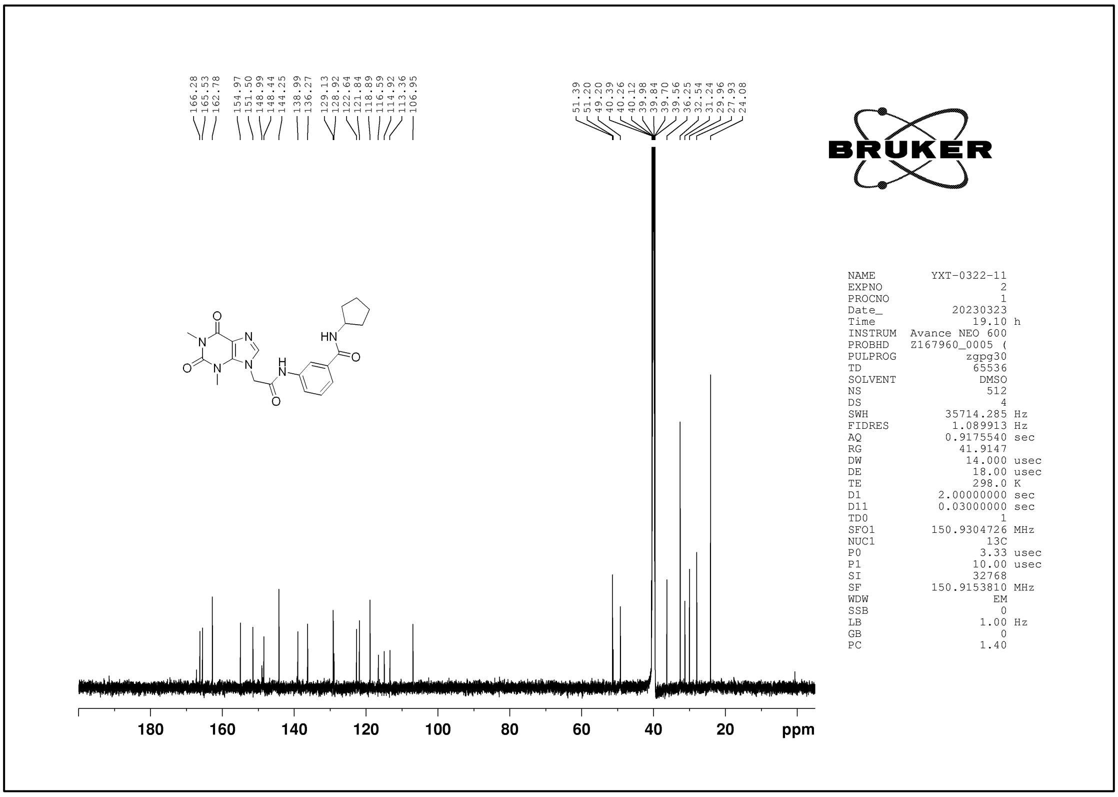

^{13}C-NMR spectrum of compound 19d

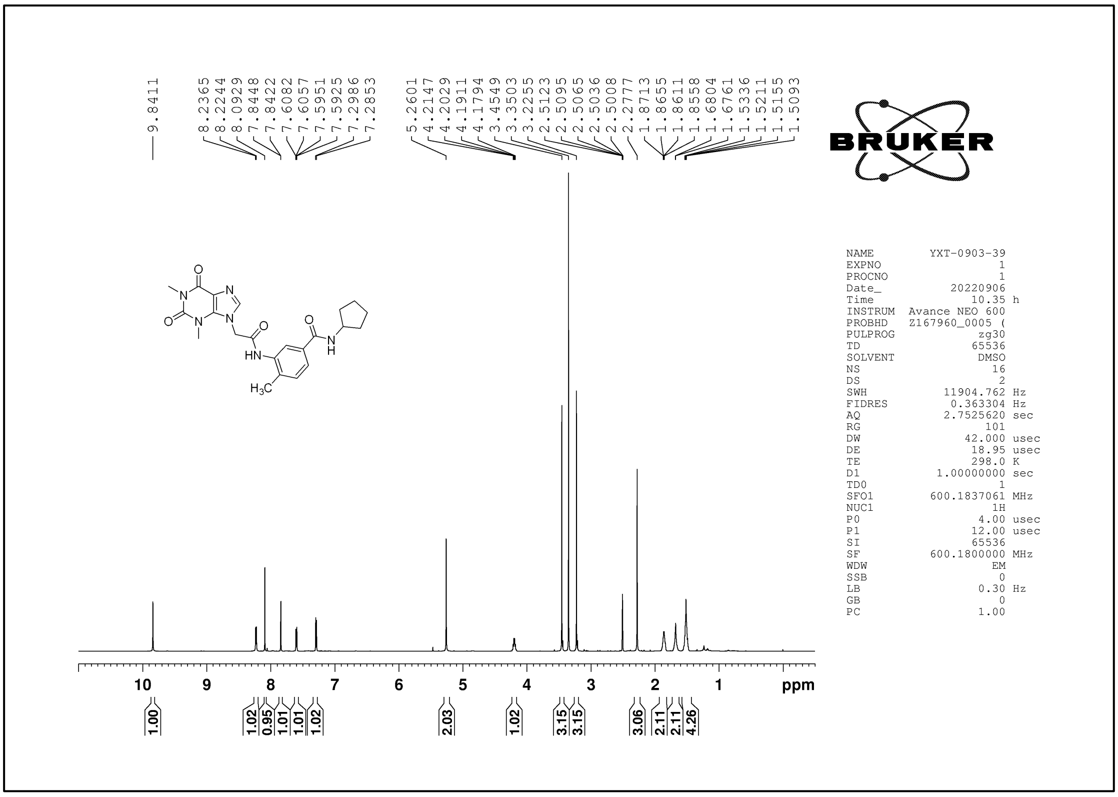

^{1}H-NMR spectrum of compound 19e

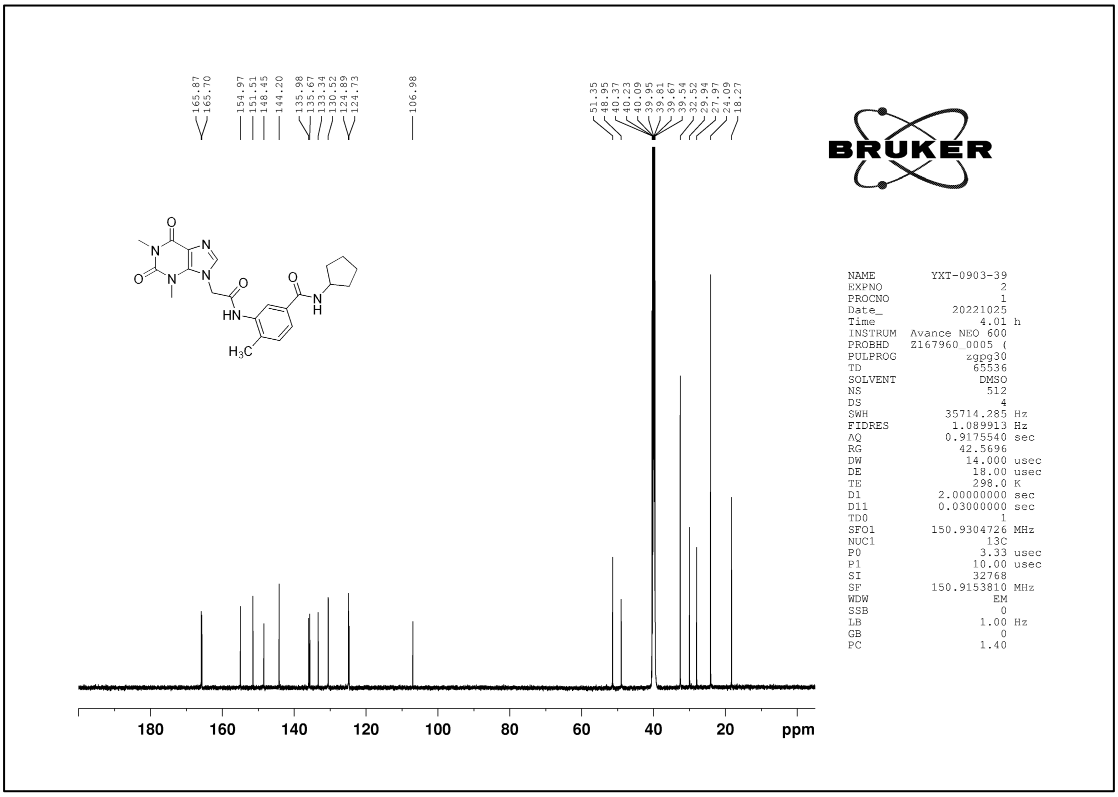

^{13}C-NMR spectrum of compound 19e

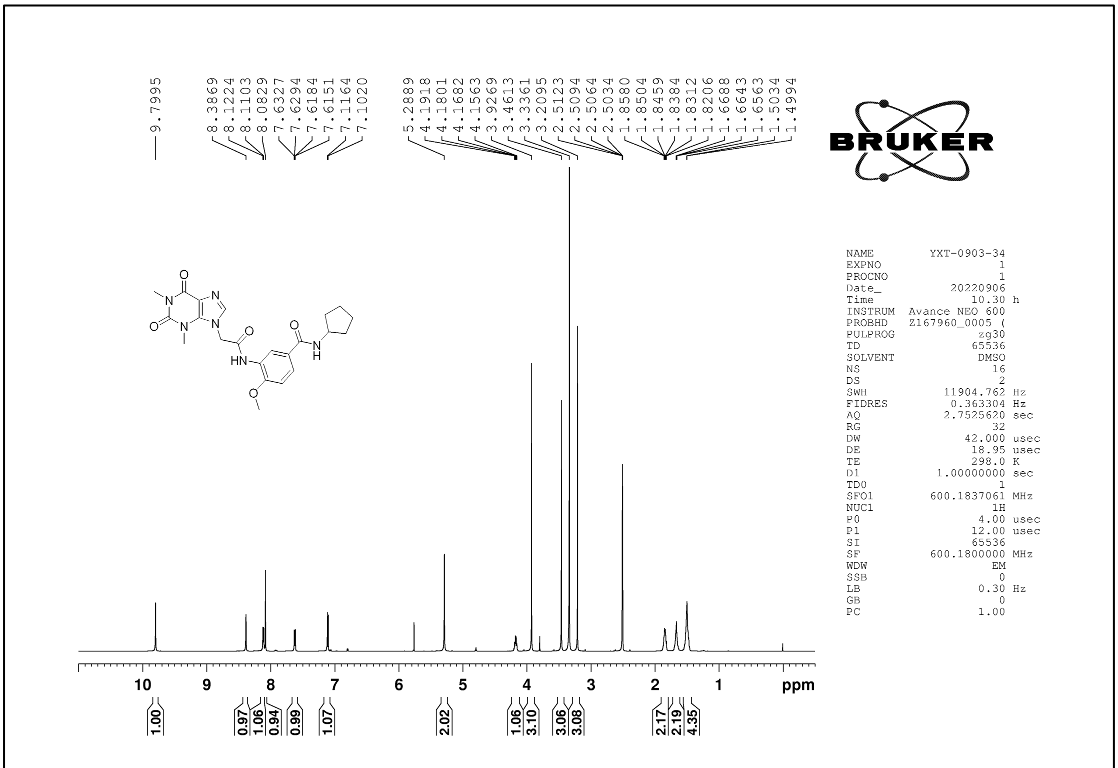

^{1}H-NMR spectrum of compound 19f

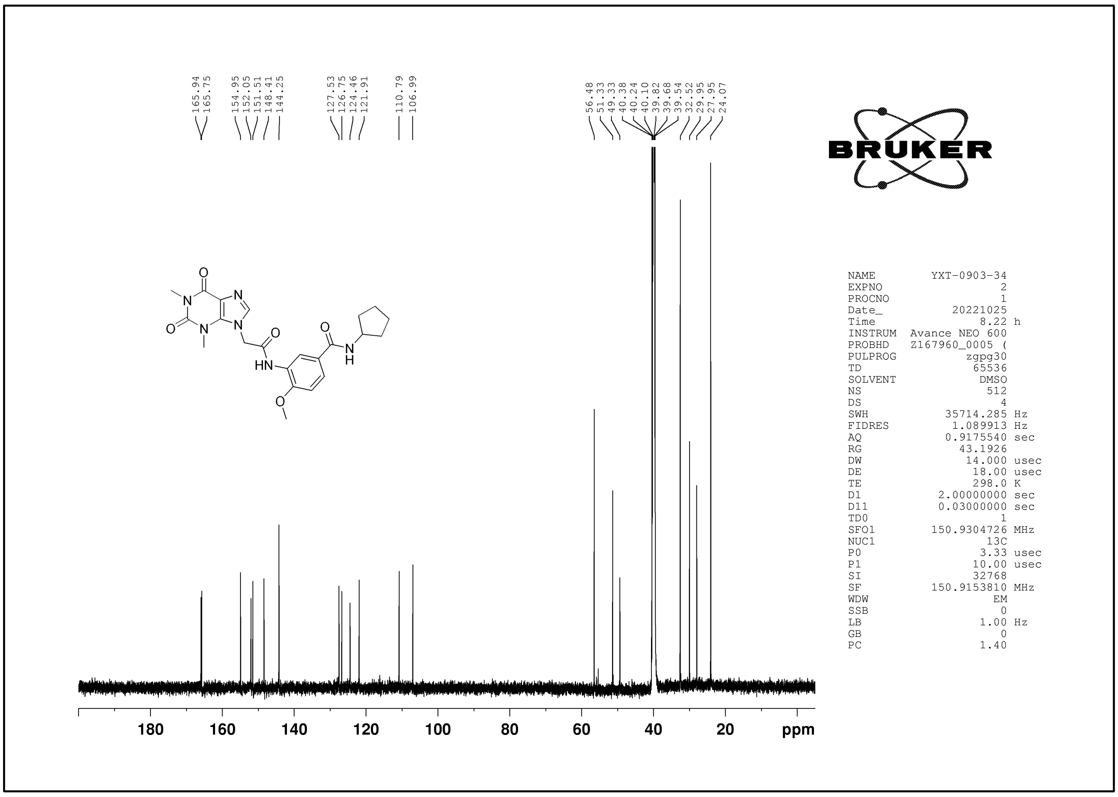

^{13}C-NMR spectrum of compound 19f

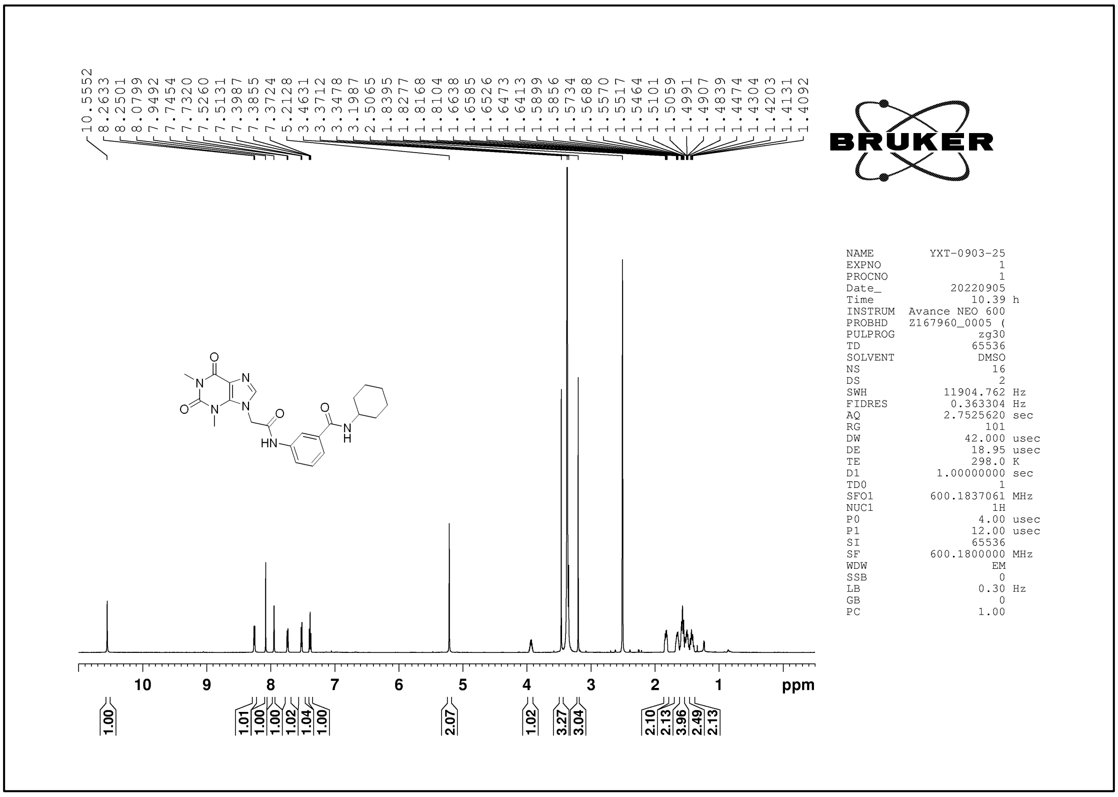

^{1}H-NMR spectrum of compound 19g

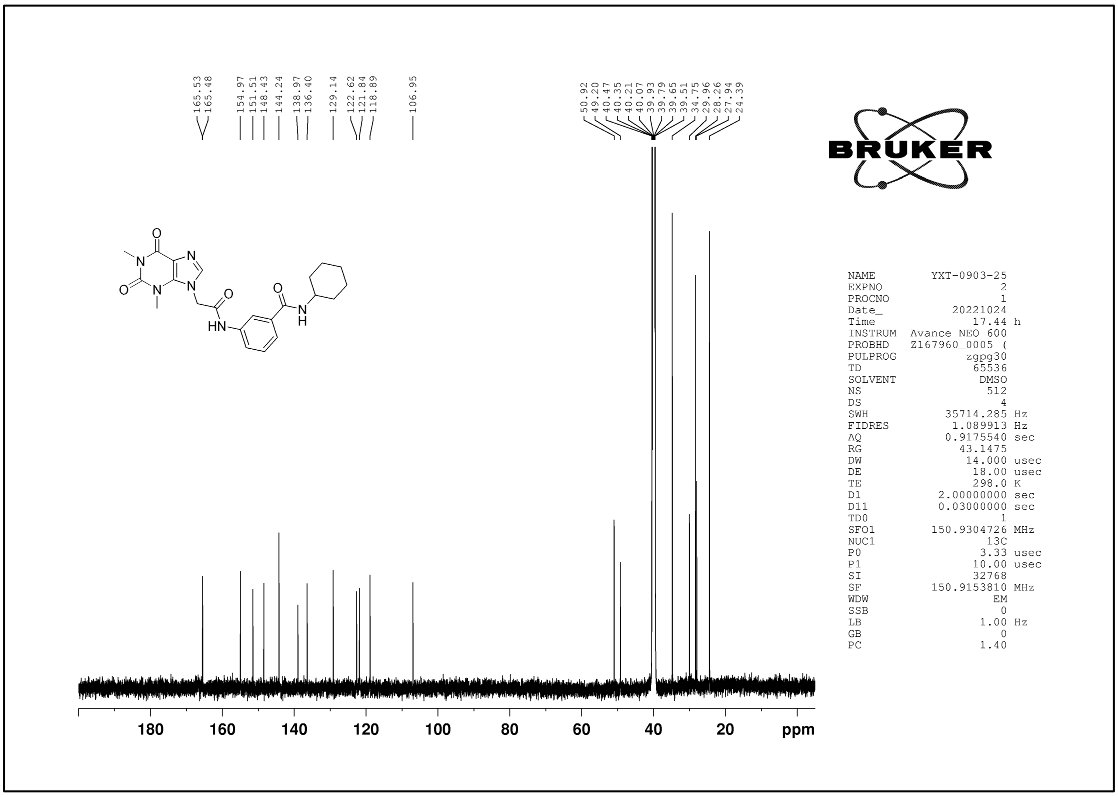

^{13}C-NMR spectrum of compound 19g

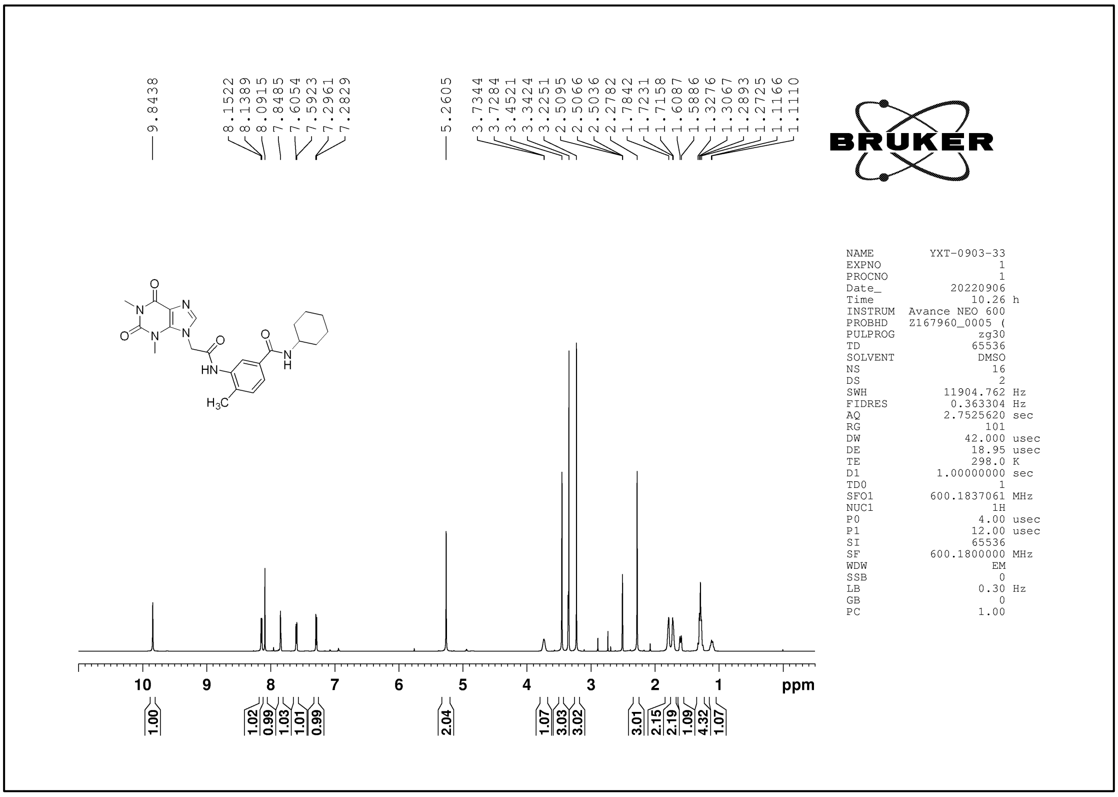

^{1}H-NMR spectrum of compound 19h

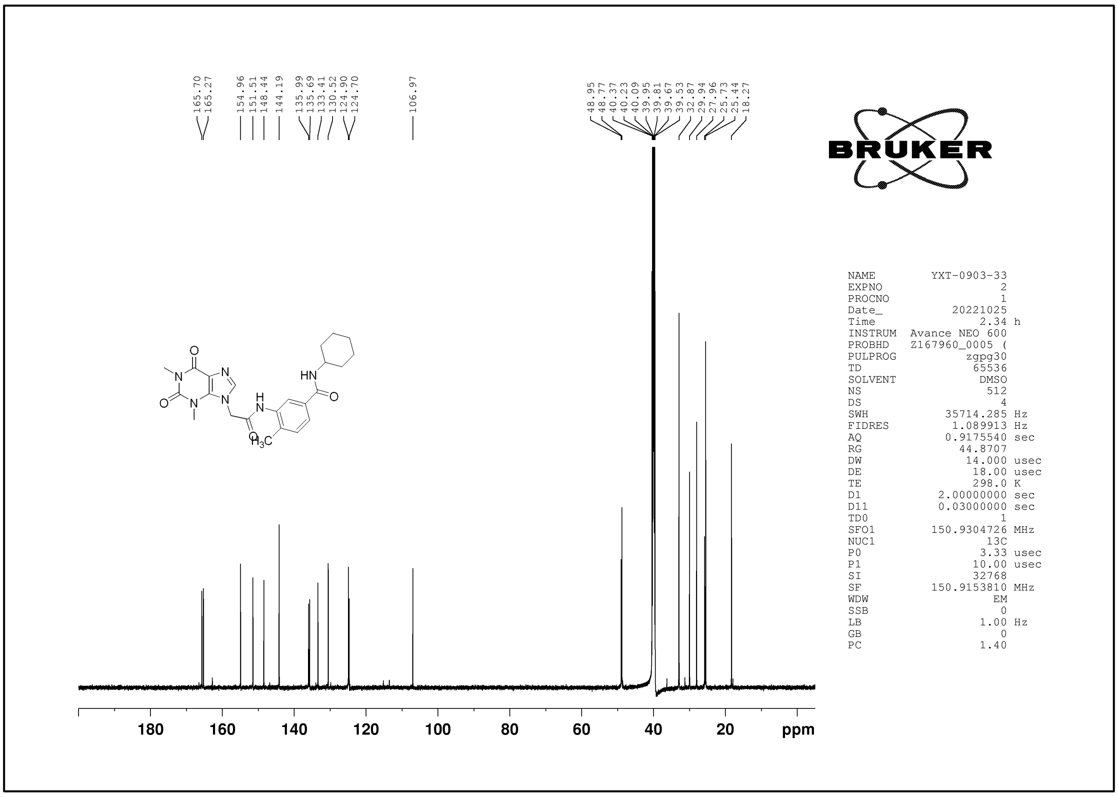

^{13}C-NMR spectrum of compound 19h

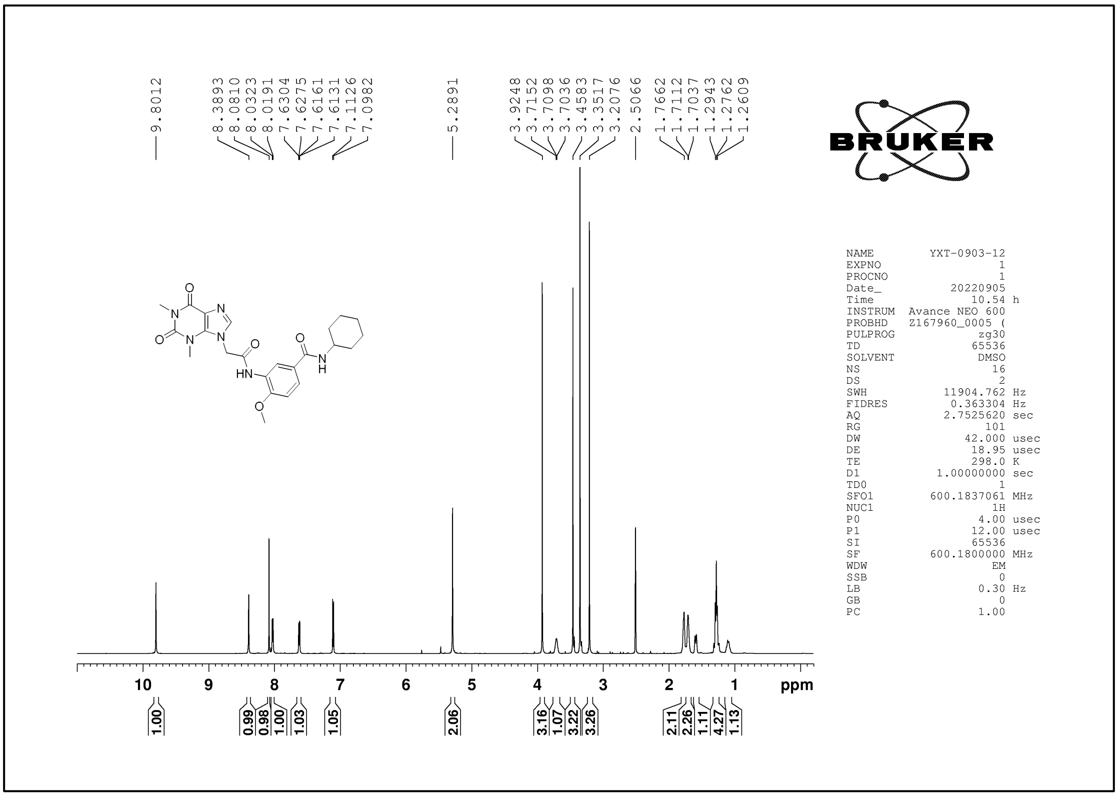

^{1}H-NMR spectrum of compound 19i

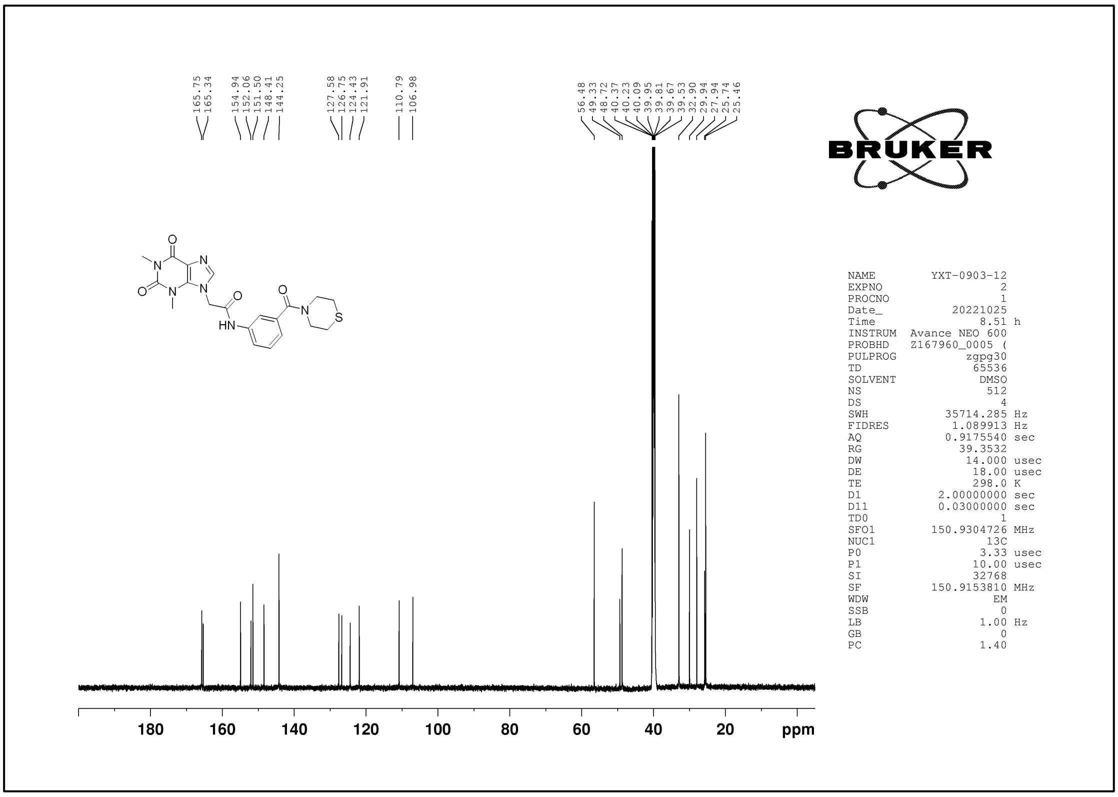

^{13}C-NMR spectrum of compound 19i

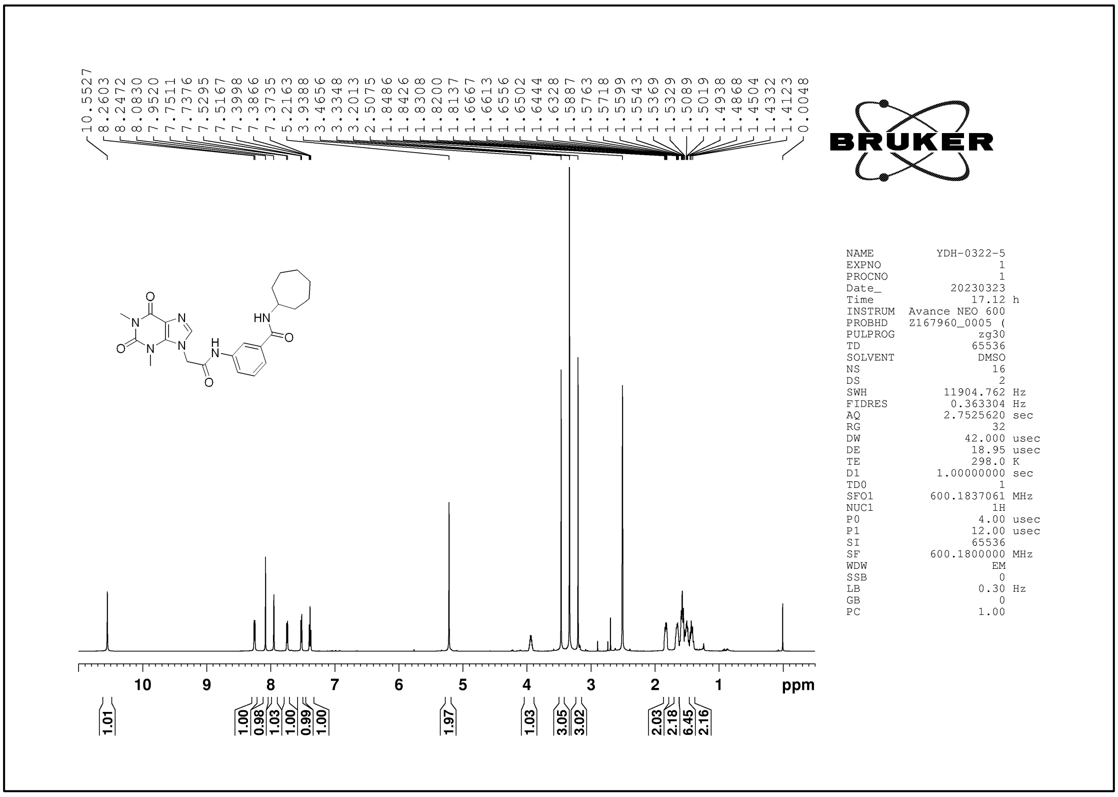

^{1}H-NMR spectrum of compound 19j

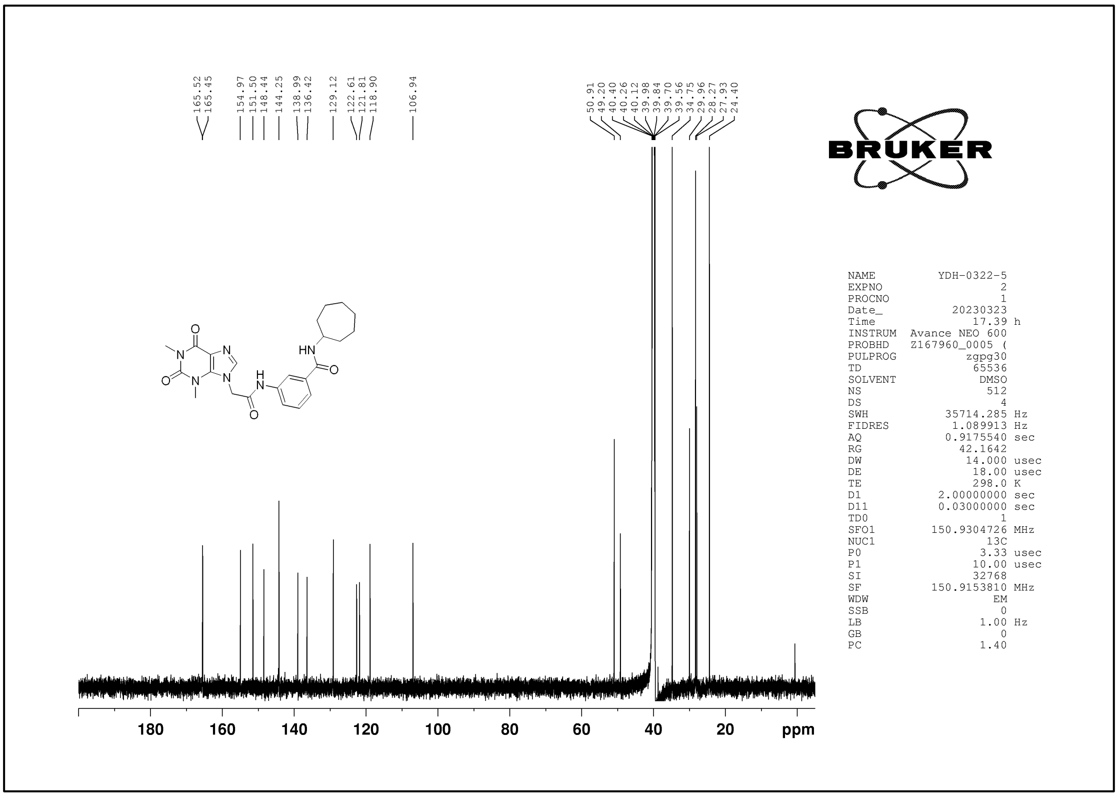

^{13}C-NMR spectrum of compound 19j

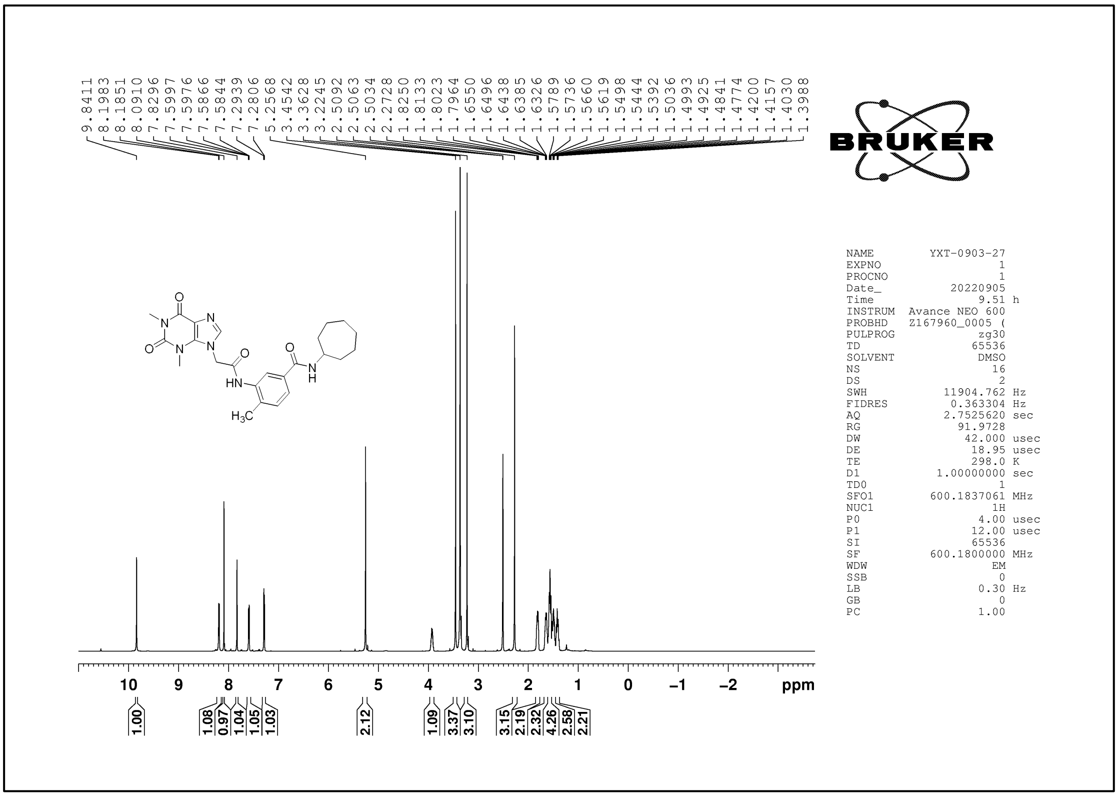

^{1}H-NMR spectrum of compound 19k

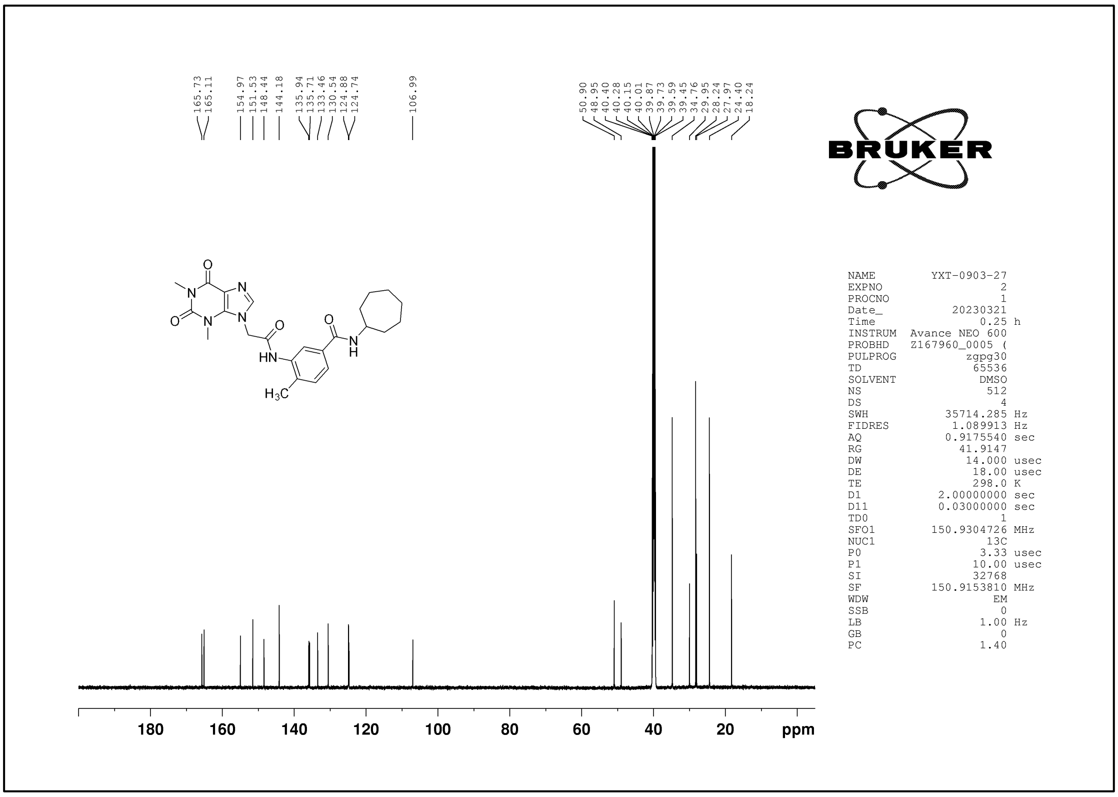

^{13}C-NMR spectrum of compound 19k

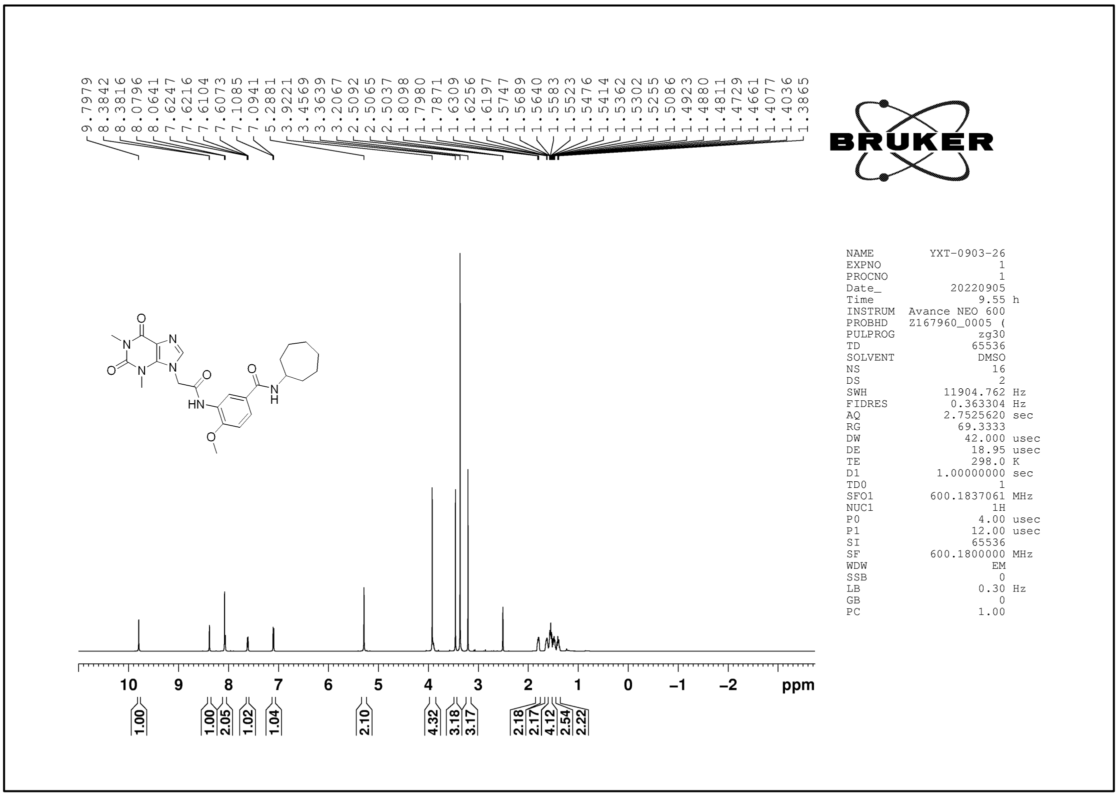

^{1}H-NMR spectrum of compound 19l

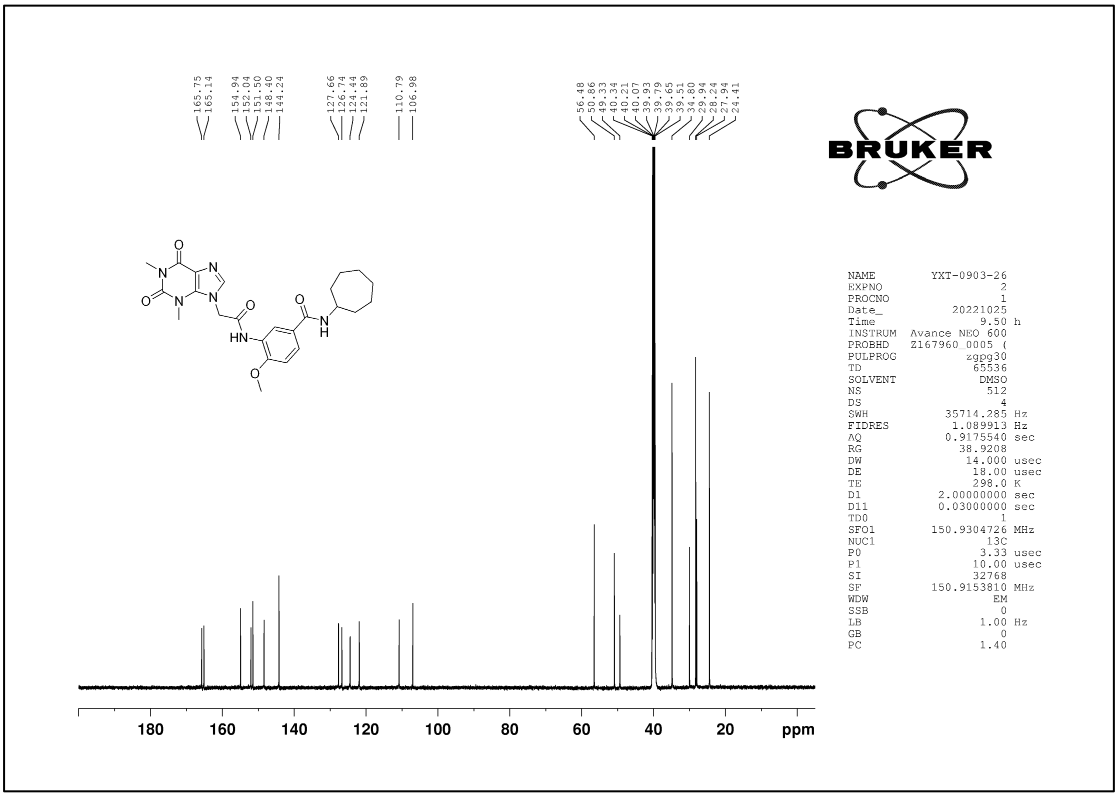

^{13}C-NMR spectrum of compound 19l

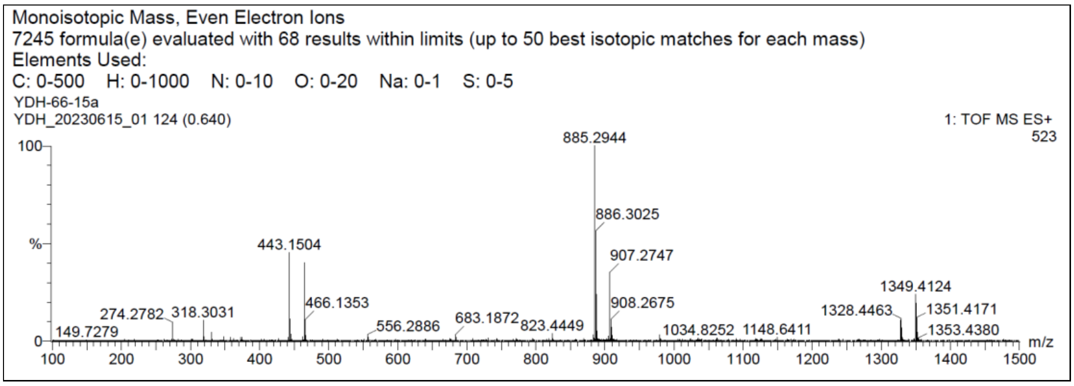

HR-MS spectrum of compound 15a

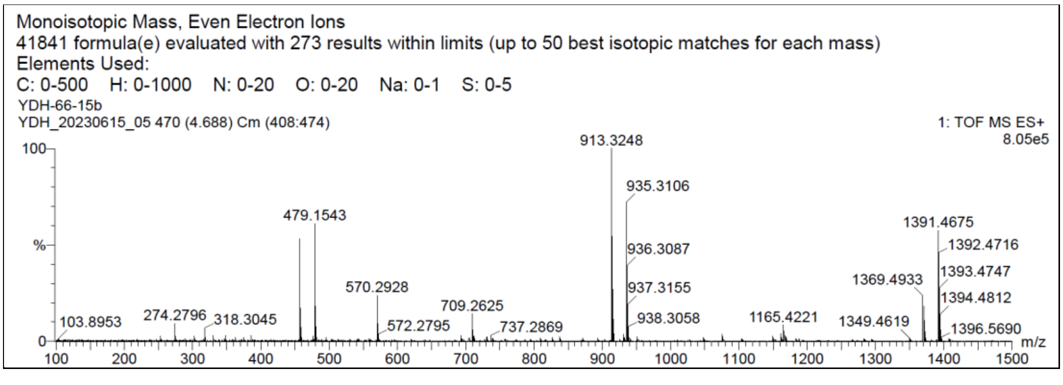

HR-MS spectrum of compound 15b

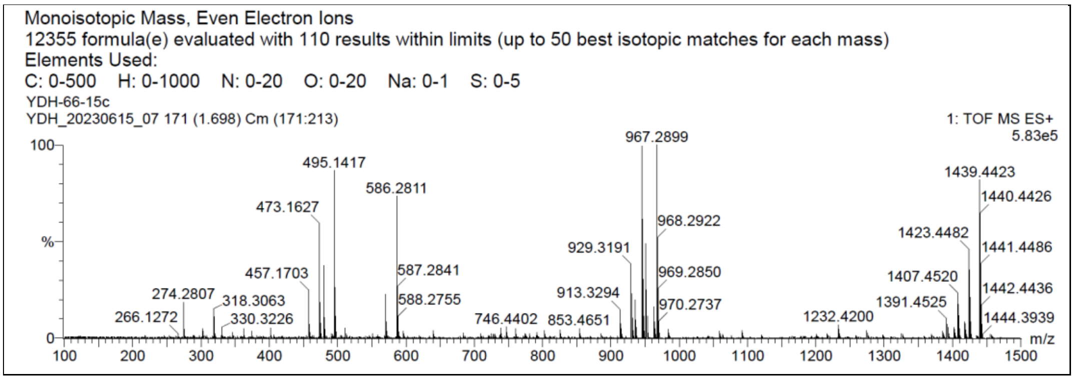

HR-MS spectrum of compound 15c

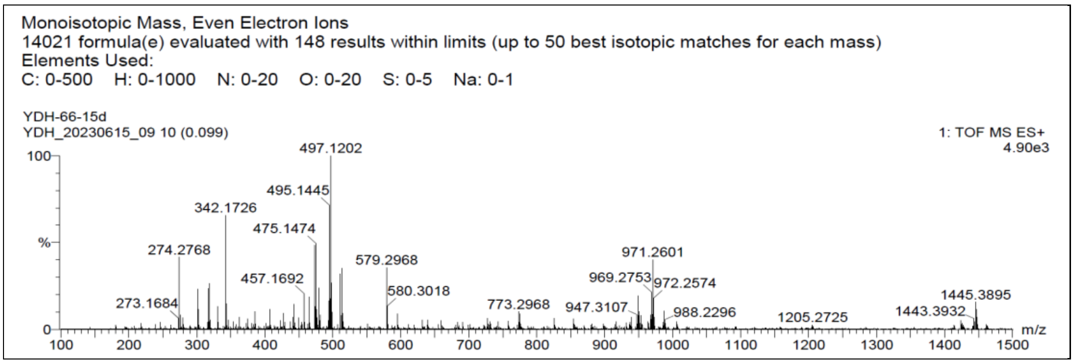

HR-MS spectrum of compound 15d

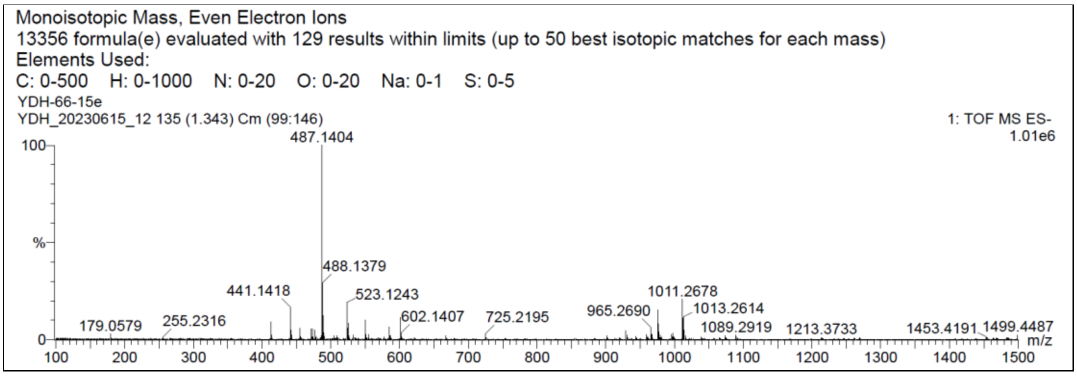

HR-MS spectrum of compound 15e

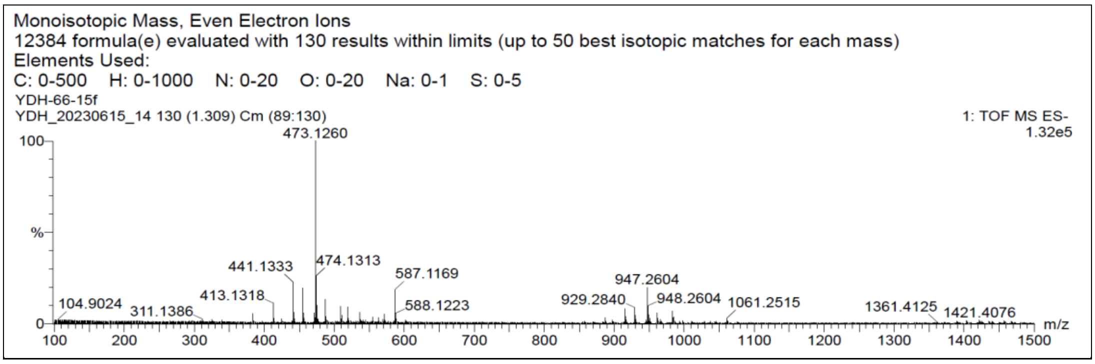

HR-MS spectrum of compound 15f

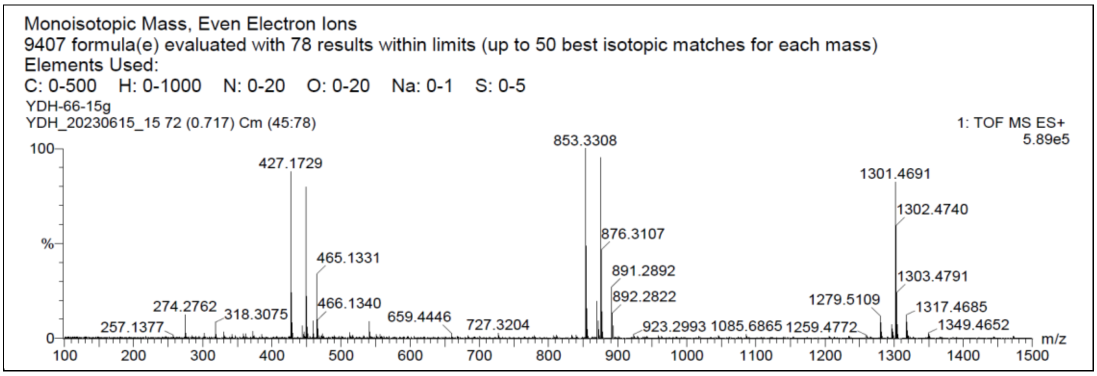

HR-MS spectrum of compound 15g

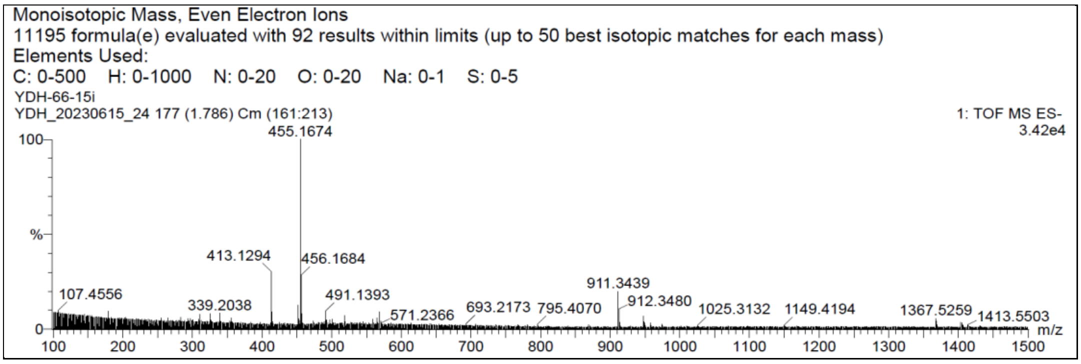

HR-MS spectrum of compound 15i

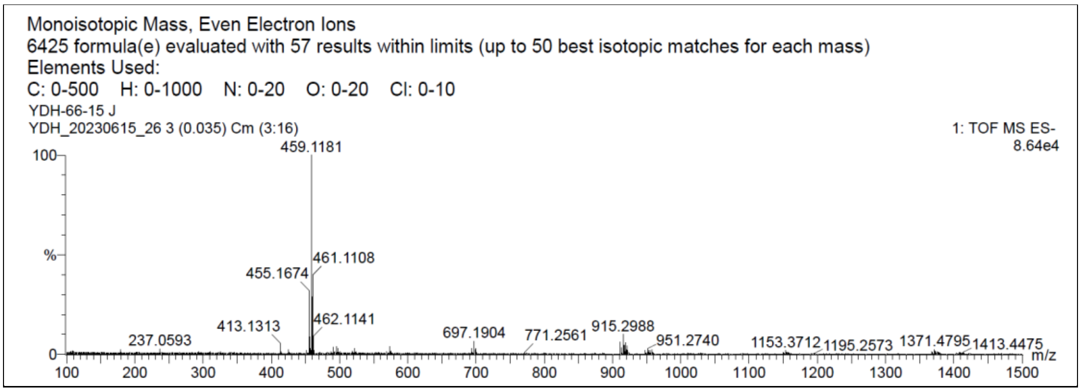

HR-MS spectrum of compound 15j

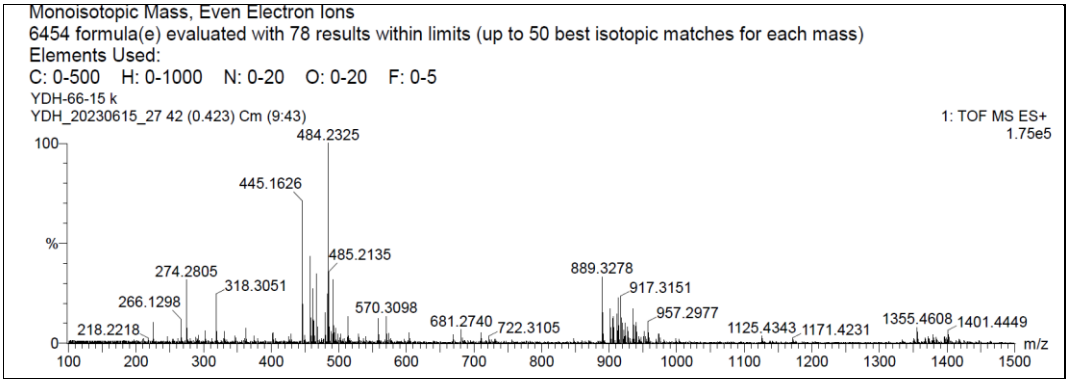

HR-MS spectrum of compound 15k

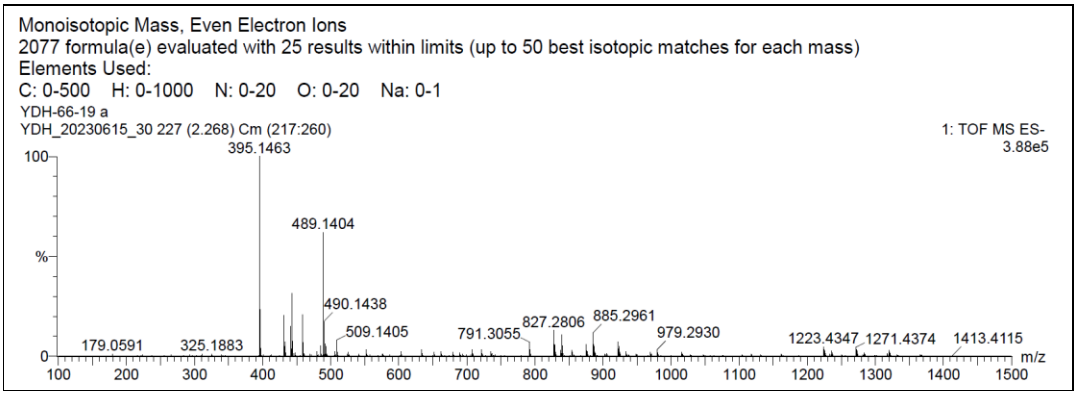

HR-MS spectrum of compound 19a

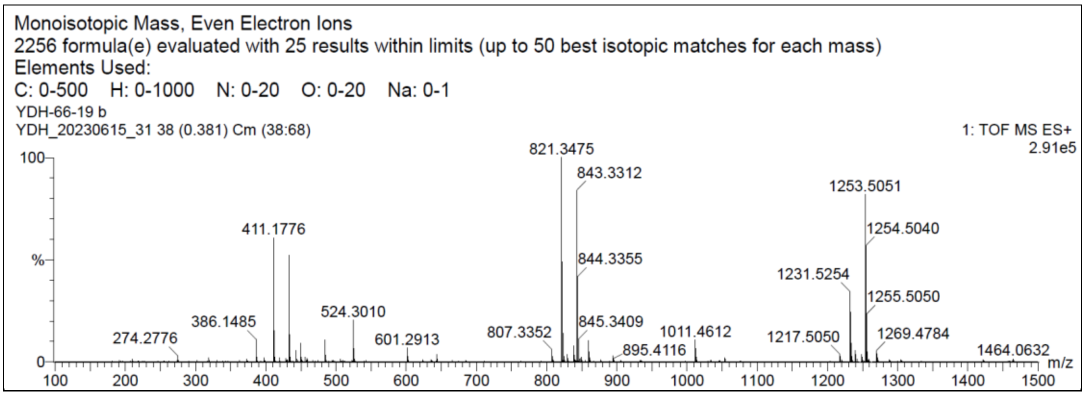

HR-MS spectrum of compound 19b

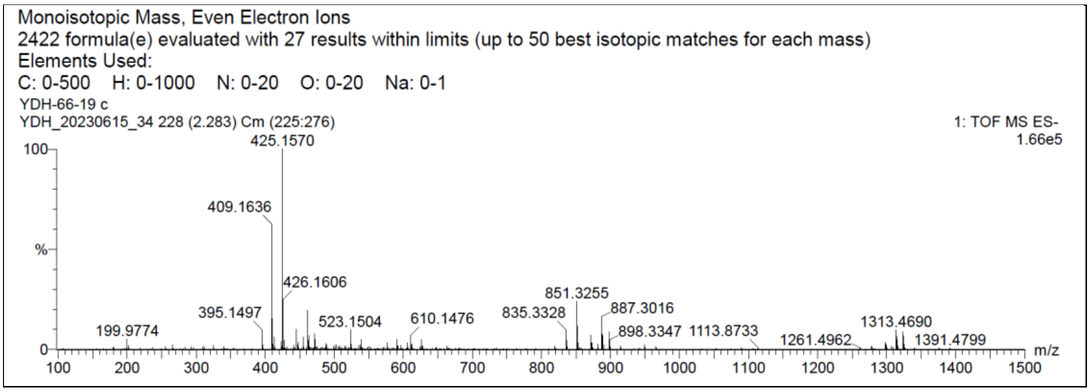

HR-MS spectrum of compound 19c

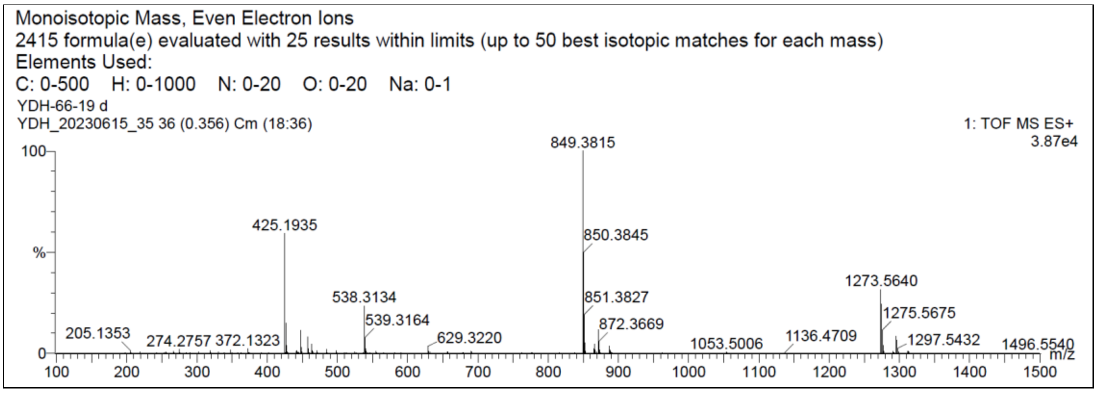

HR-MS spectrum of compound 19d

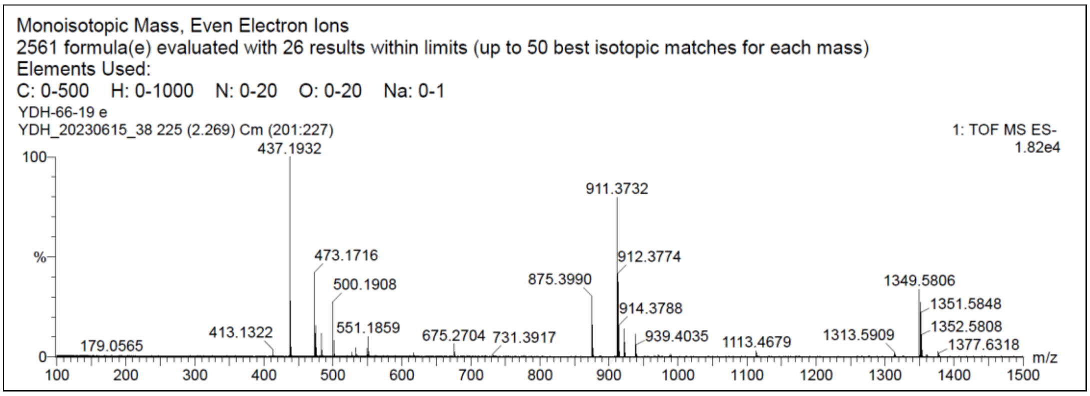

HR-MS spectrum of compound 19e

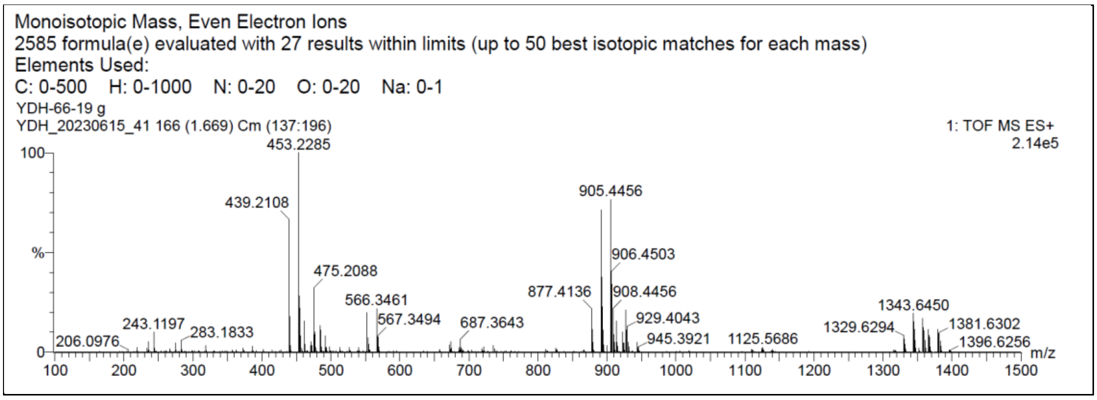

HR-MS spectrum of compound 19g

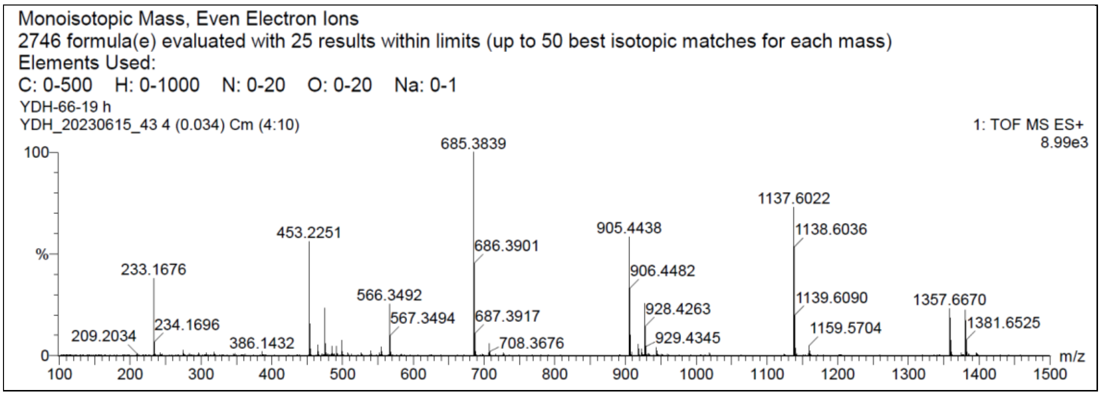

HR-MS spectrum of compound 19h

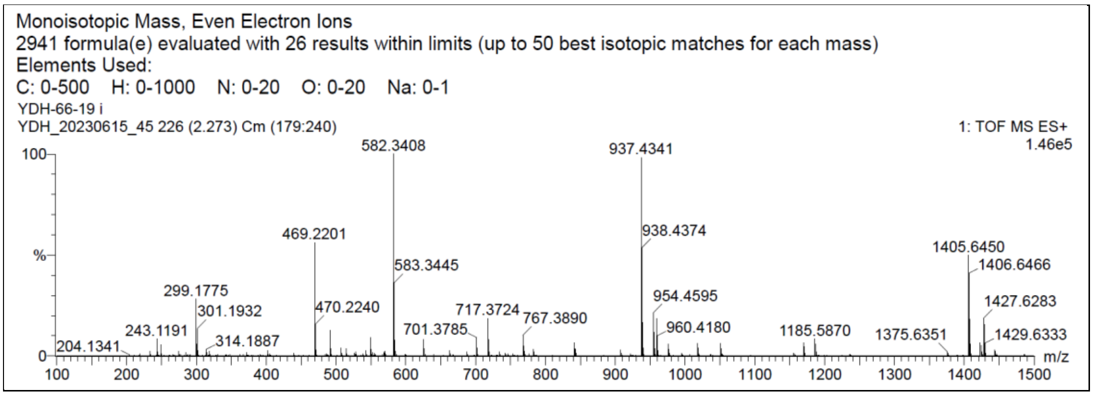

HR-MS spectrum of compound 19i

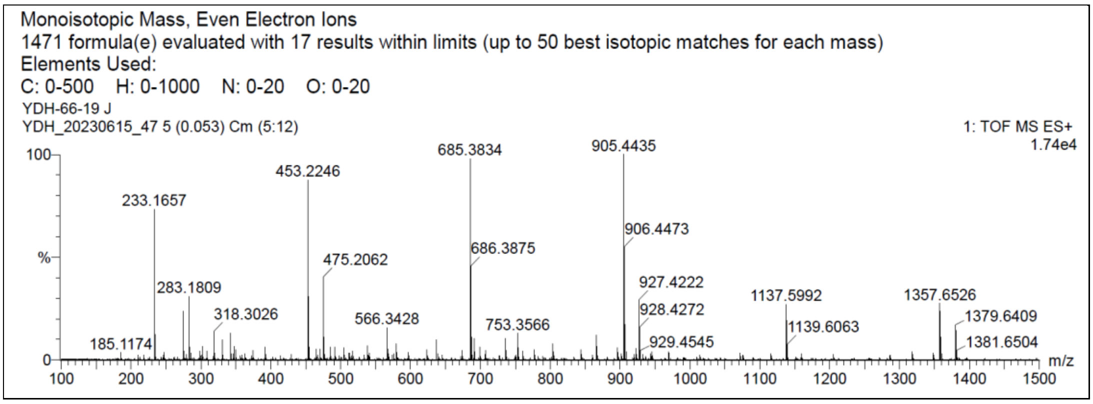

HR-MS spectrum of compound 19j

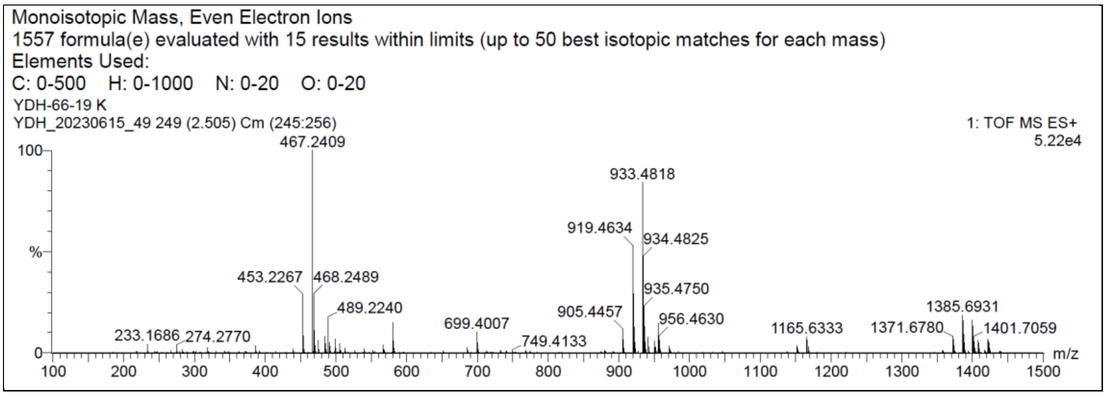

HR-MS spectrum of compound 19k

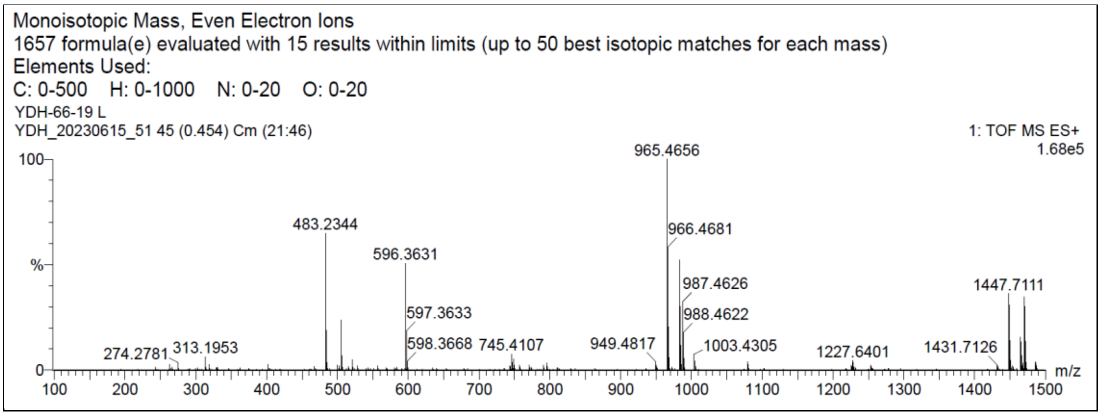

HR-MS spectrum of compound 19l
